# Country-specific key lifestyle factors and health outcomes for resource allocation in the general population: a network analysis across 29 countries

**DOI:** 10.7189/jogh.15.04011

**Published:** 2025-01-10

**Authors:** Jiaying Li, Daniel Yee Tak Fong, Kris Yuet Wan Lok, Janet Yuen Ha Wong, Mandy Man Ho, Edmond Pui Hang Choi, Vinciya Pandian, Patricia M Davidson, Wenjie Duan, Marie Tarrant, Jung Jae Lee, Chia-Chin Lin, Oluwadamilare Akingbade, Khalid M Alabdulwahhab, Mohammad Shakil Ahmad, Mohamed Alboraie, Meshari A Alzahrani, Anil S Bilimale, Sawitree Boonpatcharanon, Samuel Byiringiro, Muhammad Kamil Che Hasan, Luisa Clausi Schettini, Walter Corzo, Josephine M. De Leon, Anjanette S. De Leon, Hiba Deek, Fabio Efficace, Mayssah A El Nayal, Fathiya El-Raey, Eduardo Ensaldo-Carrasco, Pilar Escotorin, Oluwadamilola Agnes Fadodun, Israel Opeyemi Fawole, Yong-Shian Shawn Goh, Devi Irawan, Naimah Ebrahim Khan, Binu Koirala, Ashish Krishna, Cannas Kwok, Tung Thanh Le, Daniela Giambruno Leal, Miguel Ángel Lezana-Fernández, Emery Manirambona, Leandro Cruz Mantoani, Fernando Meneses-González, Iman Elmahdi Mohamed, Madeleine Mukeshimana, Chinh Thi Minh Nguyen, Huong Thi Thanh Nguyen, Khanh Thi Nguyen, Son Truong Nguyen, Mohd Said Nurumal, Aimable Nzabonimana, Nagla Abdelrahim Mohamed Ahmed Omer, Oluwabunmi Ogungbe, Angela Chiu Yin Poon, Areli Reséndiz-Rodriguez, Busayasachee Puang-Ngern, Ceryl G Sagun, Riyaz Ahmed Shaik, Nikhil Gauri Shankar, Kathrin Sommer, Edgardo Toro, Hanh Thi Hong Tran, Elvira L Urgel, Emmanuel Uwiringiyimana, Tita Vanichbuncha, Naglaa Youssef

**Affiliations:** 1 School of Nursing, Li Ka Shing Faculty of Medicine, University of Hong Kong, Hong Kong SAR, China; 2 School of Nursing, Johns Hopkins University, Baltimore, Maryland, USA; 3 School of Nursing and Health Studies, Hong Kong Metropolitan University, Hong Kong SAR, China; 4 Vice-Chancellor and Principal, University of Wollongong, Wollongong, Australia; 5 East China University of Science and Technology, Department of Social Work, Shanghai, China; 6 School of Nursing, The University of British Columbia, Kelowna, British Columbia, Canada; 7 The Nethersole School of Nursing, The Chinese University of Hong Kong, Hong Kong, China; 8 Institute of Nursing Research, Osogbo, Osun State, Nigeria; 9 College of Medicine, Majmaah University, Al Majmaah, Saudi Arabia; 10 Department of Family & Community Medicine, College of Medicine, Majmaah University, Majmaah, Saudi Arabia; 11 Department of Internal Medicine, Al-Azhar University, Cairo, Egypt; 12 Department of Urology, College of Medicine, Majmaah University, Al Majmaah, Saudi Arabia; 13 School of Public Health, JSS Medical College, JSS AHER, Mysuru, India; 14 Department of Statistics, Chulalongkorn Business School, Bangkok, Thailand; 15 Kulliyyah of Nursing, International Islamic University, Kuantan, Malaysia; 16 Italian Association against Leukaemia, Lymphoma and Myeloma, Rome, Italy; 17 Diálogos Guatemala, Guatemala, Guatemala; 18 School of Nursing, Centro Escolar University, Manila, Philippines; 19 Nursing Department, Faculty of Health Science, Beirut Arab University, Lebanon; 20 Data Centre and Health Outcomes Research Unit, Italian Group for Adult Hematologic Disease, Rome, Italy; 21 Department of Psychology, Beirut Arab University, Beirut, Lebanon; 22 Department of hepatogastroenterology and infectious diseases, Damietta faculty of medicine, Al-Azher University, Kairo, Egypt; 23 Ergonomics Research Centre, University of Guadalajara, Jalisco, Mexico; 24 Laboratory of Applied Prosocial Research, Department of Basic, Developmental and Educational Psychology, Autonomous University of Barcelona, Barcelona, Spain; 25 Faculty of Health Sciences, University of Lethbridge, Lethbridge, Alberta, Canada; 26 Faculty of Nursing, Ladoke Akintola University of Technology, Ogbomosho, Nigeria; 27 Alice Lee Centre for Nursing Studies, National University of Singapore, Singapore, Singapore; 28 School of Nursing, Wijaya Husada Health Institute, Bogor, Indonesia; 29 Department of Optometry, University of Kwazulu-Natal, Durban, South Africa; 30 Ecove, Ghaziabad, India; 31 School of Nursing, Paramedicine and Health Care Science, Charles Sturt University, New South Wales, Australia; 32 Nam Dinh University of Nursing, Nam Dinh, Vietnam; 33 Pontificia Universidad Católica de Valparaíso, School of Social Work, Valparaíso, Chile; 34 Research Department, National Commission for Medical Arbitration, Mexico, Mexico; 35 College of Medicine and Health Sciences, University of Rwanda, Kigali, Rwanda; 36 Department of Physiotherapy, Presidente Prudente, Faculty of Science and Technology, São Paulo State University (UNESP), São Paulo, Brazil; 37 Pharmacology and Toxicology Department, Faculty of Pharmacy, Benghazi University, Benghazi, Libya; 38 School of Nursing and Midwifery, College of Medicine and Health Sciences, University of Rwanda, Kigali, Rwanda; 39 Centre for Language Enhancement, College of Arts and Social Sciences, University of Rwanda, Huye, Rwanda; 40 Faculty of Medicine, Alzaiem Alazhari University, Khartoum North, Sudan; 41 Faculty of Health Sciences and Sports, Macao Polytechnic University, Macao, China; 42 National Autonomous University of Mexico, Mexico City, Mexico; 43 Mental Health and Learning division, Wrexham Maelor Hospital, Wrexham, UKyy; 44 Medical-surgical Nursing Department, Faculty of Nursing, Cairo University, Cairo, Egypt

## Abstract

**Background:**

We aimed to identify the central lifestyle, the most impactful among lifestyle factor clusters; the central health outcome, the most impactful among health outcome clusters; and the bridge lifestyle, the most strongly connected to health outcome clusters, across 29 countries to optimise resource allocation for local holistic health improvements.

**Methods:**

From July 2020 to August 2021, we surveyed 16 461 adults across 29 countries who self-reported changes in 18 lifestyle factors and 13 health outcomes due to the pandemic. Three networks were generated by network analysis for each country: lifestyle, health outcome, and bridge networks. We identified the variables with the highest bridge expected influence as central or bridge variables. Network validation included nonparametric and case-dropping subset bootstrapping, and centrality difference tests confirmed that the central or bridge variables had significantly higher expected influence than other variables within the same network.

**Results:**

Among 87 networks, 75 were validated with correlation-stability coefficients above 0.25. Nine central lifestyle types were identified in 28 countries: cooking at home (in 11 countries), food types in daily meals (in one country), less smoking tobacco (in two countries), less alcohol consumption (in two countries), less duration of sitting (in three countries), less consumption of snacks (in five countries), less sugary drinks (in five countries), having a meal at home (in two countries), taking alternative medicine or natural health products (in one country). Six central health outcomes were noted among 28 countries: social support received (in three countries), physical health (in one country), sleep quality (in four countries), quality of life (in seven countries), less mental burden (in three countries), less emotional distress (in 13 countries). Three bridge lifestyles were identified in 19 countries: food types in daily meals (in one country), cooking at home (in one country), overall amount of exercise (in 17 countries). The centrality difference test showed the central and bridge variables had significantly higher centrality indices than others in their networks (*P* < 0.05).

**Conclusions:**

In 29 countries, cooking at home, less emotional distress, and overall amount of exercise emerged as common central lifestyle, health outcome, and bridge lifestyle factors, respectively. However, notable regional variations necessitate tailored interventions and resource allocations to effectively address unique local key variables and promote holistic health in each locale. The study's cross-sectional design and self-reported data may limit generalisability, emphasising the need for cautious interpretation and further longitudinal research.

**Keywords:**

global; across-country comparisons; lifestyle; health outcomes; network analysis

Lifestyle factors – including dietary risks, alcohol and tobacco consumption, sedentary behaviours, and physical inactivity – significantly influence health outcomes both immediately and over the long term. Short-term impacts manifest in changes to physical and psychological quality of life [1], sleep [2], and social well-being [3]. Conversely, persistent unhealthy lifestyle behaviours substantially elevate the risk for non-communicable diseases such as cardiovascular disease [4], diabetes, cancer, and chronic respiratory diseases [5], ultimately affecting life expectancy and mortality [6]. The global prevalence of non-communicable diseases underscores the critical importance of lifestyle in health promotion. However, implementing comprehensive lifestyle interventions that cover all lifestyle aspects at the population level is complex and resource-intensive, necessitating the identification of the most impactful lifestyle for targeted interventions and effective resource allocation for effective holistic health promotion, especially for those low- and middle-income countries with heavier burden and less resources.

Network analysis is a technique that examines the effects of one variable within its network cluster or its connections with another network cluster while considering all other within-network variables as confounders [7]. In network analysis theory, the central lifestyle and health outcome variables are the most impactful in their respective networks, offering the highest predictive value and potential for substantial improvements when modified. The bridge lifestyle, which is the lifestyle factor most strongly connected to the health outcome network, serves a similar function but impacts the entire health network [7]. The presence of most impactful lifestyle is supported by the interconnected relationships identified by previous studies within lifestyles, health outcomes, and between them. For instance, physical inactivity is associated with other lifestyle behaviours, such as smoking, alcohol consumption and an unhealthy diet [8,9] Additionally, physical inactivity affects various interim health outcomes, including mental health and sleep quality [10]. Moreover, behaviours like smoking, drinking, and unhealthy eating also adversely impact other health outcomes, such as mental health and sleep quality [11]. Building on these intertwined relationships, a study utilised network analysis to identify central lifestyle factors, health outcomes, and bridge lifestyles using a sample drawn from various countries [12]. However, global approaches may overlook crucial cross-country variations in lifestyle patterns and cultural norms, limiting the effectiveness of interventions in specific regions. Therefore, identifying central lifestyle factors at the country level is essential to tailor interventions to local contexts.

The assumption that central lifestyle factors vary across countries is supported by multiple drivers, including socioeconomic and cultural influences, which affect the coexistence and strength of associations among lifestyle behaviours. For instance, limited access to healthy foods is more prevalent in low-income countries [13], which may result in insufficient food availability and reduced variety of food options. In contrast, high-income countries often experience higher rates of sedentary behaviours, such as prolonged sitting and increased screen time [14], due to factors like widespread use of digital technology, the prevalence of desk-based and office jobs, and greater reliance on automobiles for transportation. Additionally, cultural factors, such as the American preference for sweet flavours [15,16] and alcohol prohibition in some areas of Saudi Arabia due to religious and legal restrictions [15,16], can increase or decrease frequency of certain lifestyles. Hong Kong’s high-density urban living may limit physical activity and increase stress levels [17]. In contrast, Rwanda’s predominantly rural environment promotes higher levels of physical activity [18]. The connection between these differences and country-specific key variables can be explained by the pronounced presence of specific lifestyle factors and health outcomes. These factors may strengthen their associations with other coexisting lifestyles or health outcomes, thereby increasing their centrality within a network and making each country's key variables distinct. Identifying these diverse key lifestyles is crucial for developing effective, tailored local health interventions, yet remains poorly understood.

Therefore, this study aims to assess the interplay between lifestyle factors and health outcomes across 29 countries, and to identify the key variables (central lifestyle, central health outcome, and bridge lifestyle). We hypothesise that each country has distinct central lifestyle factors, health outcomes, and bridge lifestyles. The findings are expected to provide country-specific guidance for local resource allocation for effective holistic health improvements.

## METHODS

### Study design

This was an international cross-sectional study.

### Study settings

This study spanned six regions defined by the World Health Organization, covering 29 countries after excluding Spain due to a limited sample size of 51. Recruitment was conducted primarily through online platforms to optimise reach and facilitate voluntary participation in preferred languages. The published protocol provides detailed information on the study design [19].

### Participants and sample size

We recruited participants aged 18 and older using convenience and snowball sampling techniques. While these methods enabled efficient participant recruitment across diverse geographical locations, they also introduce potential selection biases, particularly affecting the representativeness of the socioeconomic and rural/urban diversity within the sample populations. Sample sizes for each network – lifestyle (18 nodes), health outcomes (13 nodes), and bridge (31 nodes) – were determined based on the maximum number of edges: 153, 78, and 465, respectively, adhering to the guideline of at least three participants per parameter [20]. Consequently, the required sample sizes for each network were 459, 234, and 1134 participants, respectively. Sample sizes across countries ranged 150–2238, with most meeting these criteria. Notably, centrality measures in network analysis can be considered reliable even if the sample size falls short of initial estimates, provided they pass the stability test via case-dropping subset bootstrap [20]. To ensure reliability, stability tests were conducted for all 87 networks, with only those networks that passed these tests being reported. We recognise the limitations of these sampling methods and the sample size in some countries, and their potential impact on the generalisability of our findings. To mitigate these effects, stability tests via case-dropping subset bootstrap were conducted for all 87 networks, and only those networks that passed these tests were reported.

### Measures

#### Sociodemographics

The sociodemographic variables included in this study were gender, age, country of residence, marital status, highest educational level attained, and employment status.

#### Measurement of lifestyle factors and health outcomes

Item generation for the study questionnaire was developed for assessing a thorough literature review and collaborative discussions with public health professionals, nurses, and nutritionists. The chosen variables for lifestyle and health outcomes reflect dimensions commonly acknowledged in public health research, focusing on key lifestyle dimensions, including diet, physical activity, and substance use, as well as health outcomes across physical, psychological, social, and financial well-being. Specifically, 18 lifestyle factors include:

• dietary (types of food in daily meals, consumption of fruits and vegetables, frozen food/food products, snacks, soft drinks, juices, or other sugary drinks, meals at home, cooking at home, takeout food, traditional Chinese medicine or natural health products, and oral supplements/vitamins)

• exercise (frequency, duration, type, and overall amount)

• sedentary behaviours (sitting and screen time duration)

• addiction behaviours (smoking tobacco and alcohol consumption).

The 13 health-related outcomes include:

• physical well-being (weight, appetite, sleep quality, perceived physical health, and quality of life)

• psychological well-being (mental burden, emotional distress)

• social well-being (family disputes, social support provided, social support received, and social activities)

• financial well-being (income and economic burden).

The initial English version was created and face validity ensured through rigorous reviews that refined the questionnaire by enhancing clarity, eliminating redundancies, and structuring the flow of questions to facilitate ease of completion. To ensure cultural relevance, consultations with domain experts from various countries were conducted, followed by translations into multiple languages using a standard forward-backwards method. Lifestyle factors and health outcomes were assessed using single items, making formal psychometric evaluation inapplicable [19].

Participants rated the impact of COVID19 on 18 lifestyle factors and 13 general health outcomes using a 5-point Likert scale, with 1 indicating 'substantially reduced', 3 'no change', and 5 'substantially increased'. To ensure consistent item alignment reflecting healthier lifestyles and improved health outcomes, certain items were reverse coded by appending a 'less' prefix.

### Data collection

Data were collected via online survey platforms and supplemented with offline electronic PDF forms in areas with limited internet access. Both methods were standardised in content and structure to ensure comparability and maintain data quality across different collection environments. Rigorous pre-testing confirmed the equivalence of the two formats, allowing for consistent data collection across all regions. To encourage participation, one Hong Kong dollar was donated to the Red Cross for each completed questionnaire. The response rate was recorded at 75.2%.

### Validation and rigor

To enhance internal validity, validation questions were incorporated into the questionnaire. Participants were asked 'Where does the sun rise every day?' Additionally, to ensure cultural relevance in Nigeria, the question 'Where is your STATE capital?' was used. Additionally, each country’s questionnaire was pilot-tested with a minimum of 10 participants prior to administration. The results from these pilot tests indicated that participant responses were within expected parameters and no confusion regarding the questions was reported; therefore, no modifications to the questionnaire were deemed necessary. Moreover, to mitigate social desirability bias, the survey was conducted anonymously. Furthermore, we performed data quality checks before analysis to remove any responses indicating misreporting or misunderstanding. These measures collectively aim to reduce potential biases and enhance the accuracy and reliability of our data set.

### Statistical analysis

Data collected for our study were transferred to a Microsoft Excel database to facilitate stringent quality control. This included removing incomplete or duplicate responses to ensure data integrity. We also addressed data inconsistencies by discarding responses that did not align with validation questions, such as incorrect details regarding sunset times or capital cities, thereby enhancing the accuracy of our data set and the validity of our analysis. Subsequent analyses were performed using *R* Statistical Software (v4.1.1; R Core Team, Vienna, Austria). The network analyses encompassed four domains: topological overlap assessment, network estimation, stability testing, and the computation of centrality and bridge centrality indices.

#### Checking topological overlap

We used the ‘networktools’ R package's 'goldbricker' function to identify unique variables to prevent artificial relationships among similar variables in a network. A significance threshold of 0.25 for inclusion and a significance level of 0.01 were applied [21].

#### Network estimation

For each country, three networks were analysed: one comprising all remaining lifestyle factors, another encompassing all remaining health outcomes, and a bridge network connecting the two. Pairwise associations were estimated using partial correlation analysis to control for confounding effects from other nodes. The least absolute shrinkage and selection operator was applied to minimise edges and nullify small correlations, while the extended Bayesian information criterion helped select the tuning parameter, resulting in a more interpretable and sparse network [22]. Network estimation and visualisation were performed using the ‘bootnet’ and ‘qgraph’ R packages, respectively [22]. In visual representations, nodes symbolised network items and edges their relationships, with edge thickness indicating the strength of association – blue for positive and red for negative associations.

#### Network stability

Network stability was evaluated using the ‘bootnet’ package [22]. Edge weight stability was assessed with 95% confidence intervals (CIs) derived from nonparametric bootstrapping; narrower CIs indicated greater network credibility [22]. Centrality stability was measured by the correlation stability coefficient (CS-C), calculated via case-dropping subset bootstrap. A CS-C value above 0.25 was considered acceptable, with values above 0.5 being preferable [22].

#### Central node, centrality, bridge node, and bridge centrality

A central node significantly influences a network through its connections with other nodes, while bridge nodes connect closely to different clusters within another network [23,24]. In network analysis, betweenness centrality measures how frequently a node occurs on the shortest paths between other pairs, underscoring its intermediary role. Closeness centrality calculates a node's average distance to all others, indicating network accessibility. Strength centrality totals the edge weights connected to a node, showing its connectivity. Differently, expected influence centrality sums edge weights without absolute conversions, capturing dominant influences in networks with both positive and negative edges. Given our objective to identify nodes that exert significant cumulative effects, particularly in networks with negative interactions, we focus on expected influence centrality to pinpoint central nodes and to identify bridge nodes effectively [25]. The 'qgraph' package in R calculated the expected influence index, which accounts for both positive and negative edges to identify central nodes with the highest values. Bridge nodes were assessed using the 'networktools' package in R, which utilises the bridge expected influence (one-step) index that aggregates a node's edges to nodes in other networks [23]. Centrality differences between two nodes were evaluated using Wilcoxon tests with 1000 bootstrapped indices, generated through the ‘bootnet’ package in R. Multiple comparisons were adjusted for using Holm-Bonferroni corrections.

## RESULTS

### Demographics and item description

After screening 19 094 responses, 16 461 were eligible for analysis. Exclusions included blank or incomplete responses (1940), duplicates (116), inconsistent entries (450), responses from non-participating countries (126), and records missing age or gender information (1). **Table 1** outlines the sociodemographic details, means, standard deviations, and abbreviations of lifestyle factors and health outcomes, while Table S1 in the **Online Supplementary Document** displays the breakdown by country.

**Table 1:** Sociodemographic of respondents from 29 countries (n = 16 461)

Variables	N/mean	%/SD
Age group		
*18–24*	4845	29.4
*25–29*	2343	14.2
*30–34*	1930	11.7
*35–39*	1850	11.2
*40–44*	1417	8.6
*45–49*	1147	7.0
*50–54*	971	5.9
*55–59*	663	4.0
*60–64*	699	4.2
*≥65*	596	3.6
Gender		
*Female*	10 317	62.7
*Male*	6045	36.7
*Non-binary*	99	0.6
Marital status		
*Married/cohabitation/common-law*	7244	44.0
*Separated/divorced/widowed*	730	4.4
*Single*	8486	51.6
Education		
*Primary or below*	405	2.5
*Secondary*	2618	15.9
*Associate degree*	1573	9.6
*College*	2246	13.6
*Bachelor*	6495	39.5
*Graduate*	2952	17.9
*Missing value*	172	1.0
Employment		
*Job seeking*	883	5.4
*Laid off*	170	1.0
*Not in workforce*	990	6.0
*Retired*	612	3.7
*Self-employed*	1300	7.9
*Student*	4577	27.8
*Working (≥40 h/week)*	5183	31.5
*Working (1–39 h/week)*	2746	16.7
Country stay		
*Australia*	639	3.9
*Brazil*	553	3.4
*Burundi*	369	2.2
*Canada*	368	2.2
*Chile*	342	2.1
*Egypt*	461	2.8
*Guatemala*	229	1.4
*Hong Kong*	2127	12.9
*India*	529	3.2
*Indonesia*	482	2.9
*Italy*	203	1.2
*Lebanon*	440	2.7
*Libya*	645	3.9
*Macau*	250	1.5
*Mainland China*	667	4.1
*Malaysia*	535	3.3
*Mexico*	1016	6.2
*Nigeria*	590	3.6
*Philippines*	457	2.8
*Republic Of Sudan*	538	3.3
*Rwanda*	150	0.9
*Saudi Arabia*	631	3.8
*Singapore*	237	1.4
*South Africa*	198	1.2
*South Korea*	2238	13.6
*Thailand*	723	4.4
*UK*	212	1.3
*USA*	213	1.3
*Vietnam*	419	2.5
Lifestyle factors (abbreviations)		
*Food types in daily meals (L1)*	3.01	0.87
*Consumption of fruits and vegetables (L2)*	3.15	0.90
*Less consumption of frozen food/food products (L3)*	3.01	0.97
*Less consumption of snacks (L4)*	2.96	1.00
*Less soft drinks/juices/other sugary drinks (L5)*	2.80	1.03
*Having a meal at home (L6)*	3.86	0.99
*Cooking at home (L7)*	3.80	0.98
*Less eating takeout food (L8)*	2.95	1.19
*Taking alternative medicine or natural health products (L9)*	2.89	0.91
*Taking oral supplements/vitamins (L10)*	3.06	0.93
*Less smoking tobacco (L11)*	2.63	0.94
*Less alcohol consumption (L12)*	2.62	0.96
*Less duration of sitting (L13)*	3.65	0.98
*Less duration of screen time (L14)*	3.74	0.98
*Frequency of exercise (L15)*	2.81	1.08
*Duration of exercise (L16)*	2.78	1.07
*Type of exercise (L17)*	2.77	1.02
*Overall amount of exercise (L18)*	2.77	1.07
Health outcomes (abbreviations)		
*Lose weight (H1)*	3.21	0.90
*Appetite (H2)*	3.13	0.83
*Physical health (H3)*	2.91	0.80
*Sleep quality (H4)*	2.86	0.96
*Quality of life (H5)*	2.71	0.98
*Less mental burden (H6)*	3.40	1.07
*Less emotional distress (H7)*	3.36	1.04
*Family disputes (H8)*	3.10	0.87
*Social support provided (H9)*	3.09	0.86
*Social support received (H10)*	2.97	0.84
*Social activities (H11)*	2.36	1.06
*Income (H12)*	2.65	0.93
*Less economic burden (H13)*	3.24	1.01

H – health outcome, L – lifestyle, SD – standard deviation

### Non-redundant items remained for network analysis

Redundancy analysis via the Goldbricker method excluded L15 (**Table 1**), L16, and L17 for overlapping with L18, representing exercise habits more accurately. The final network comprised 15 lifestyle factors and 13 health outcomes with no additional redundancies.

### Lifestyle networks across each country

#### Network stability

The bootstrapped 95% CI analysis confirmed the accuracy of edge weights in the lifestyle network, with narrow intervals suggesting reliable estimates (Figure S1 in the **Online Supplementary Document**). Besides, CS-C values for expected influence, derived from a case-dropping subset bootstrap, ranged 0.21–0.75 (**Table 2**; Figure S2 in the **Online Supplementary Document**). Excluding the Unities States, these values surpassed the 0.25 threshold, affirming the interpretability of 28 country-specific lifestyle networks.

**Table 2:** Summary of network measures across 29 countries (n = 16 461)

Country	Lifestyle network						Health outcome network						Bridge network			
	**Nonzero/total edges = 105 (%)**	**Largest edge (PC-C)**	**Second largest edge (PC-C)**	**Third largest edge (PC-C)**	**CS-C value**	**Central lifestyle**	**Nonzero/total edges = 78 (%)**	**Largest edge (PC-C)**	**Second largest edge (PC-C)**	**Third largest edge (PC-C)**	**CS-C value**	**Central health outcome**	**Nonzero/total edges = 378 (%)**	**CS-C value**	**Bridge lifestyle**	**Bridge edge (PC-C)**
Australia	68 (64.8)	L9–L2 (0.37)	L9–L1 (0.24)	L10–L6 (0.24)	0.59	L9	52 (66.7)	H13–H12 (0.64)	H5–H4 (0.34)	H6–H3 (0.26)	0.21	NA	124 (32.8)	0.13	NA	NA
Brazil	50 (47.6)	L7–L6 (0.63)	L14–L13 (0.61)	L5–L4 (0.40)	0.67	L4	21 (26.9)	H10–H9 (0.52)	H7–H6 (0.51)	H2–H1 (0.38)	0.75	H5	60 (15.9)	0.75	L18	L18–H3 (0.34)
Burundi	46 (43.8)	L2–L1 (0.43)	L7–L6 (0.26)	L4–L3 (0.24)	0.28	L1	46 (59.0)	H8–H7 (0.34)	H7–H6 (0.28)	H4–H3 (0.26)	0.52	H3	83 (22.0)	0.28	L18	L18–H4 (0.12)
Canada	35 (33.3)	L14–L13 (0.68)	L7–L6 (0.64)	L2–L1 (0.45)	0.67	L13	40 (51.3)	H7–H6 (0.75)	H10–H9 (0.58)	H5–H4 (0.31)	0.67	H7	64 (16.9)	0.52	L18	L18–H3 (0.25)
Chile	42 (40.0)	L14–L13 (0.67)	L7–L6 (0.48)	L10–L9 (0.36)	0.28	L4	25 (32.1)	H7–H6 (0.70)	H2–H1 (0.47)	H5–H4 (0.40)	0.75	H5	32 (8.5)	0.59	L18	L18–H3 (0.25)
Egypt	64 (61.0)	L7–L6 (0.68)	L12–L11 (0.66)	L14–L13 (0.49)	0.67	L6 = L7	34 (43.6)	H2–H1 (0.40)	H7–H6 (0.39)	H8–H7 (0.37)	0.67	H4	125 (33.1)	0.00	NA	NA
Guatemala	16 (15.2)	L7–L6 (0.58)	L12–L11 (0.47)	L14–L13 (0.42)	0.59	L7	22 (28.2)	H7–H6 (0.47)	H2–H1 (0.38)	H5–H4 (0.30)	0.52	H4	23 (6.1)	0.44	L18	L18–H3 (0.24)
Hong Kong	72 (68.6)	L7–L6 (0.60)	L5–L4 (0.57)	L14–L13 (0.57)	0.75	L5	46 (59.0)	H7–H6 (0.51)	H10–H9 (0.37)	H5–H4 (0.29)	0.75	H7	169 (44.7)	0.67	L18	L18–H11 (0.14)
India	59 (56.2)	L12–L11 (0.76)	L7–L6 (0.60)	L14–L13 (0.58)	0.75	L5	26 (33.3)	H7–H6 (0.63)	H10–H9 (0.44)	H2–H1 (0.40)	0.67	H5 = H7	85 (22.5)	0.59	L18	L18–H3 (0.23)
Indonesia	68 (64.8)	L12–L11 (0.71)	L7–L6 (0.54)	L14–L13 (0.47)	0.67	L11	53 (68.0)	H7–H6 (0.63)	H10–H9 (0.55)	H13–H12 (0.49)	0.75	H10	176 (46.6)	0.44	L18	L18–H5 (0.08)
Italy	25 (23.8)	L14–L13 (0.64)	L7–L6 (0.49)	L5–L4 (0.32)	0.21	NA	17 (21.8)	H5–H4 (0.41)	H7–H6 (0.40)	H2–H1 (0.36)	0.52	H5	37 (9.8)	0.13	NA	NA
Lebanon	59 (56.2)	L14–L13 (0.68)	L7–L6 (0.60)	L10–L9 (0.29)	0.52	L7	42 (53.9)	H7–H6 (0.54)	H2–H1 (0.53)	H8–H7 (0.37)	0.67	H7	127 (33.6)	0.20	NA	NA
Libya	64 (61.0)	L7–L6 (0.71)	L12–L11 (0.59)	L14–L13 (0.53)	0.67	L5 = L6	37 (47.4)	H2–H1 (0.55)	H7–H6 (0.42)	H8–H7 (0.36)	0.75	H7	86 (22.8)	0.36	L1	L1–H2 (0.11)
Macau	43 (41.0)	L7–L6 (0.72)	L12–L11 (0.64)	L14–L13 (0.57)	0.52	L7	14 (18.0)	H7–H6 (0.60)	H2–H1 (0.35)	H5–H4 (0.35)	0.67	H7	23 (6.1)	0.00	NA	NA
Mainland China	52 (49.5)	L7–L6 (0.85)	L12–L11 (0.75)	L14–L13 (0.66)	0.75	L7	40 (51.3)	H7–H6 (0.67)	H5–H4 (0.47)	H2–H1 (0.42)	0.75	H5	144 (38.1)	0.44	L18	L18–H1 (0.15)
Malaysia	59 (56.2)	L12–L11 (0.78)	L7–L6 (0.62)	L14–L13 (0.61)	0.67	L11	46 (59.0)	H7–H6 (0.66)	H10–H9 (0.60)	H2–H1 (0.47)	0.75	H10	117 (31.0)	0.44	L18	L18–H3 (0.16)
Mexico	53 (50.5)	L14–L13 (0.68)	L7-L6 (0.65)	L12–L11 (0.54)	0.75	L4 = L5	44 (56.4)	H7–H6 (0.59)	H2–H1 (0.46)	H5–H4 (0.38)	0.75	H5	107 (28.3)	0.75	L18	L18–H3 (0.34)
Nigeria	50 (47.6)	L12–L11 (0.66)	L7–L6 (0.60)	L14–L13 (0.45)	0.75	L7	39 (50.0)	H7–H6 (0.51)	H10–H9 (0.43)	H5–H4 (0.26)	0.75	H7	140 (37.0)	0.36	L7	L7–H5 (0.07)
Philippines	53 (50.5)	L7–L6 (0.74)	L12–L11 (0.66)	L14–L13 (0.60)	0.75	L7	44 (56.4)	H7–H6 (0.74)	H10–H9 (0.57)	H2–H1 (0.43)	0.75	H10	139 (36.8)	0.44	L18	L18–H3 (0.20)
Republic of Sudan	42 (40.0)	L12–L11 (0.62)	L7–L6 (0.61)	L14–L13 (0.32)	0.67	L7	34 (43.6)	H7–H6 (0.41)	H2–H1 (0.38)	H4–H3 (0.24)	0.67	H7	101 (26.7)	0.21	NA	NA
Rwanda	34 (32.4)	L14–L13 (0.55)	L2–L1 (0.53)	L7–L6 (0.50)	0.36	L12 = L13 = L7	7 (9.0)	H7–H6 (0.38)	H2–H1 (0.18)	H12–H11 (0.15)	0.44	H7 = H6	22 (5.8)	0.00	NA	NA
Saudi Arabia	67 (63.8)	L7–L6 (0.67)	L14–L13 (0.58)	L10–L9 (0.34)	0.59	L7	43 (55.1)	H7–H6 (0.51)	H2–H1 (0.50)	H5–H4 (0.40)	0.75	H4	125 (33.1)	0.21	NA	NA
Singapore	36 (34.3)	L14–L13 (0.61)	L7–L6 (0.54)	L12–L11 (0.46)	0.28	L13	24 (30.8)	H7–H6 (0.70)	H10–H9 (0.45)	H5–H4 (0.33)	0.44	H7 = H6	83 (22.0)	0.13	NA	NA
South Africa	19 (18.1)	L7–L6 (0.53)	L12–L11 (0.39)	L14–L13 (0.39)	0.44	L7	19 (24.4)	H7–H6 (0.58)	H10–H9 (0.43)	H2–H1 (0.41)	0.60	H6	54 (14.3)	0.00	NA	NA
South Korea	71 (67.6)	L7–L6 (0.71)	L5–L4 (0.52)	L14–L13 (0.52)	0.75	L4	39 (50.0)	H7–H6 (0.78)	H10–H9 (0.59)	H4–H3 (0.37)	0.75	H7	133 (35.2)	0.75	L18	L18–H3 (0.28)
Thailand	58 (55.2)	L12–L11 (0.74)	L14–L13 (0.52)	L7–L6 (0.43)	0.67	L5	44 (56.4)	H7–H6 (0.53)	H13–H12 (0.42)	H2–H1 (0.41)	0.75	H4	135 (35.7)	0.28	L18	L18–H11 (0.10)
U K	24 (22.9)	L7–L6 (0.58)	L14–L13 (0.49)	L5–L4 (0.24)	0.36	L4	12 (15.4)	H7–H6 (0.60)	H10–H9 (0.32)	H4–H3 (0.27)	0.44	H7	36 (9.5)	0.28	L18	L18–H3 (0.16)
USA	29 (27.6)	L7–L6 (0.63)	L14–L13 (0.57)	L10–L9 (0.42)	0.21	NA	26 (33.3)	H7–H6 (0.72)	H2–H1 (0.43)	H10–H9 (0.42)	0.60	H5	37 (9.8)	0.44	L18	L18–H3 (0.22)
Vietnam	49 (46.7)	L12–L11 (0.76)	L7–L6 (0.68)	L14–L13 (0.53)	0.75	L12	39 (50.0)	H10–H9 (0.73)	H7–H6 (0.56)	H8–H7 (0.38)	0.75	H7	145 (38.4)	0.52	L18	L18–H11 (0.12)

CS-C – correlation stability coefficient, H – health outcome, L – lifestyle, NA – not applicable, PC-C – partial correlation coefficient

#### Network structure and central lifestyle

**Figure 1:**
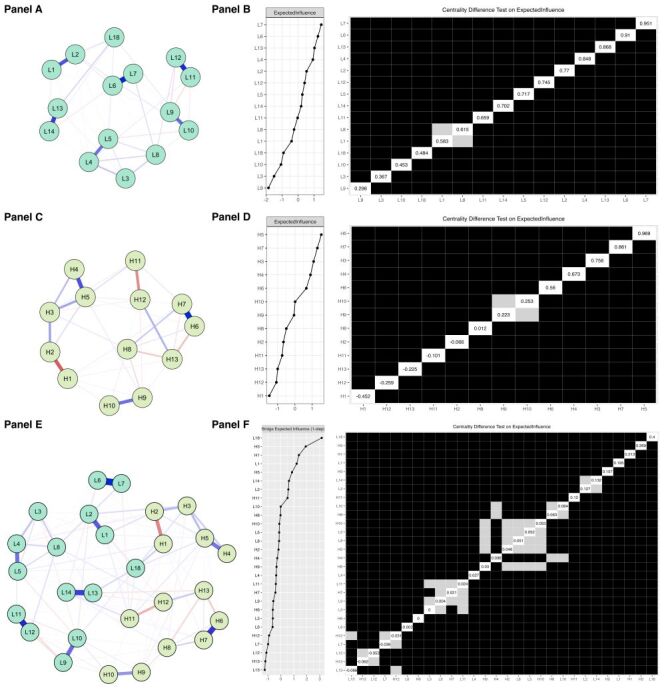
Network structure and centrality difference test of lifestyles (**Panels A–B**), health outcomes (**Panels C–D**), and combined (**Panels E–F**) in mainland China. The abbreviations of nodes in **Panels A**, **C**, and **E** can be found in **Table 1**. In **Panels B**, **D**, and **F**, a grey cell indicates that there is no significant difference between the corresponding two variables. A dark cell indicates that there is a significant difference between the corresponding two variables at 5% level of significance. A white cell displays the value of the expected influence or bridge expected influence.

Using Mainland China as an example, **Figure 1**, Panel A shows the lifestyle network with 49.5% of its 150 edges being nonzero, indicating strong connectivity. The strongest connections were between (the edge connected Lifestyle 7 and Lifestyle 6 (L7–L6), partial correlation coefficient = 0.85), L11–L12 (partial correlation coefficient = 0.75), and L13–L14 (partial correlation coefficient = 0.66) (**Table 2**). The central node, cooking at home (L7), was confirmed by centrality index plots and bootstrapped difference tests (*P*<0.05) (**Figure 1**, Panel B). For the other 27 countries, Figure S3 in the **Online Supplementary Document** presents the network structures, with nonzero edge percentages ranging from 15.2 in Guatemala to 68.6% in Hong Kong (**Table 2**). Figure S4 in the **Online Supplementary Document** contains centrality index plots and results of centrality bootstrapped difference tests. Table S2 in the **Online Supplementary Document** contains the partial correlation coefficients for all edges across all countries.

### Health outcome networks across countries

#### Network stability

Narrow CIs across countries confirmed the precision of estimated edge weights (Figure S5 in the **Online Supplementary Document**). Besides, CS-C values (**Table 2**; Figure S6 in the **Online Supplementary Document**), varied 0.21–0.75. Australia was the only country below the 0.25 threshold, resulting in 28 interpretable health outcome networks.

#### Network structure and central health outcome

For Mainland China (**Figure 1**, Panel C) displays a network structure with 51.3% (40 out of 78) of edges being nonzero. The strongest connections were between H7–H6 (**Table 1**) and H5–H4 (both 0.67), and H2–H1 (0.42) (**Table 2**). Quality of life (H5) was identified as the most influential health outcome, confirmed by centrality index and bootstrapped difference tests (*P*<0.05) (**Figure 1**, Panel D). For the other 27 countries, Figure S3 in the **Online Supplementary Document** illustrates the network structures, with nonzero edge percentages ranging from 15.4 in the UK to 68.0% in Indonesia (**Table 2**). Figure S4 in the **Online Supplementary Document** contains centrality index plots and results of centrality bootstrapped difference tests. Table S3 in the **Online Supplementary Document** provides the partial correlation matrix for all edges across all networks.

### Bridge networks across countries

#### Network stability

Figure S7 in the **Online Supplementary Document** illustrates the precision of estimated edge weights for each country with narrow CIs, indicating high accuracy. Besides, CS-C values for bridge networks varied between 0.00–0.75 (**Table 2**; Figure S8 in the **Online Supplementary Document**). Ten countries, including Australia, Egypt, Italy, Lebanon, Macau, Republic of Sudan, Rwanda, Saudi Arabia, Singapore, and South Africa, fell below the 0.25 threshold, yielding 19 interpretable bridge networks.

#### Network structure and bridge lifestyle

**Figure 1**, Panel E shows Mainland China's network with 144 of 378 edges being nonzero. The overall amount of exercise (L18) emerged as the most influential bridge lifestyle, significantly outperforming other nodes as shown by centrality bootstrapped difference tests (*P*<0.05) (**Figure 1**, Panel F). The corresponding bridge edge is L18–H1 (lose weight). For the other 18 countries, Figure S3 in the **Online Supplementary Document** displays network structures, with nonzero edge percentages ranging from 6.1 in Guatemala to 46.6% in Indonesia (**Table 2**). Figure S4 in the **Online Supplementary Document** contains centrality index plots and results of centrality bootstrapped difference tests. Table S4 in the **Online Supplementary Document** contains the partial correlation matrix for all networks.

### Summary of the central and bridge variables in networks across 29 countries

Nine types of central lifestyle were identified among 28 countries:

• cooking at home (L7) in Guatemala, Lebanon, Macau, Mainland China, Nigeria, Philippines, Republic of Sudan, Rwanda, Saudi Arabia, South Africa, and Egypt

• food types in daily meals (L1) in Burundi

• ess smoking tobacco (L11) in Indonesia and Malaysia

• less alcohol consumption (L12) in Rwanda and Vietnam

• less duration of sitting (L13) in Canada, Rwanda, and Singapore

• less consumption of snacks (L4) in Brazil, Chile, Mexico, South Korea, and UK

• less sugary drinks (L5) in Hong Kong, India, Libya, Mexico, and Thailand

• having a meal at home (L6) in Egypt and Libya

• taking alternative medicine or natural health products (L9) in Australia.

Six types of central health outcome were identified among 28 countries:

• social support received (H9) in Indonesia, Malaysia, and Philippines

• physical health (H3) in Burundi

• sleep quality (H4) in Egypt, Guatemala, Saudi Arabia, and Thailand

• quality of life (H5) in Brazil, Chile, India, Italy, Mainland China, Mexico, and USA

• less mental burden (H6) in Rwanda, Singapore, and South Africa

• less emotional distress (H7) in Canada, Hong Kong, India, Lebanon, Libya, Macau, Nigeria, Republic of Sudan, Rwanda, Singapore, South Korea, UK, and Vietnam.

Three types of bridge lifestyle were identified among 19 countries:

• food types in daily meals (L1) in Libya

• cooking at home (L7) in Nigeria

• overall amount of exercise (L18) in Brazil, Burundi, Canada, Chile, Guatemala, Hong Kong, India, Indonesia, Mainland China, Malaysia, Mexico, Philippines, South Korea, Thailand, United Kingdom, United States, and Vietnam.

**Figure 2:**
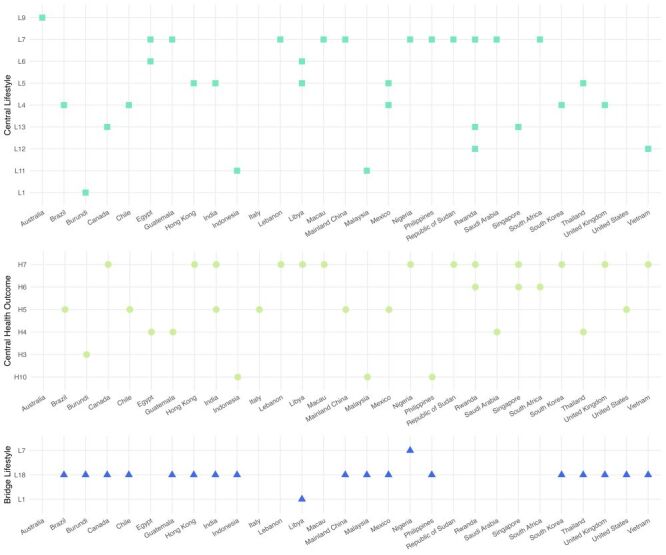
Central lifestyles, central health outcomes, and bridge lifestyles by country.

**Figure 2** visually summarises the distribution of central lifestyles, health outcomes, and bridge lifestyles across all participating countries.

## DISCUSSION

This study employed network analysis to examine complex relationships between lifestyle factors and health outcomes across 29 countries, pinpointing key variables for use as potential predictive markers or intervention targets. We identified four central lifestyle categories: diet-related behaviours, natural health product usage, substance use, and sedentary habits; and four key health outcomes: physical health, emotional distress, social support, and quality of life. Additionally, three bridge lifestyle factors – food types in daily meals, home cooking, and overall exercise amount – were recognised as focal points for targeted resource allocation to enhance network-wide health outcomes, given the established causal relationship between lifestyle and health. Modifying central lifestyle factors can enhance all within-network lifestyles and improve long-term health outcomes. Central health outcomes highlight opportunities for beyond-lifestyle interventions, such as better health care service utilisation. Bridge lifestyles offer specific pathways for optimising overall health through targeted adjustments. Shared central or bridge variables across regions highlight the potential for collaborative strategies that leverage common strengths and address shared challenges.

In 28 lifestyle networks, four central lifestyle categories emerged: diet-related behaviours, natural health product usage, substance use, and sedentary habits, reflecting complex cultural, economic, and public health interactions. Specifically, Australia's focus on natural health products as a central lifestyle, likely driven by strong cultural acceptance and a preference for alternative health practices [26], enhances preventive lifestyles such as diet and exercise. In contrast, central alcohol consumption in Indonesia and Malaysia is influenced by significant binge drinking in Malaysia and rising harm from illicit home-produced alcohol in Indonesia [27,28]. Similarly, tobacco consumption was prominent in Rwanda and Vietnam, likely due to cultural norms and high smoking prevalence [29,30]. Reduced sitting time, significant in Rwanda and Singapore, corresponds with high urbanisation and pandemic-related increases in sedentary behaviour [31,32]. Moreover, diet-related behaviours such as specific food choices, reduced consumption of snacks and sugary drinks, home cooking, and having meals at home are pivotal in Rwanda and 23 other countries. Diet's centrality within the lifestyle network may be explained by its integral role in daily life and its deep connections to cultural and economic conditions [33,34], along with its coexistence with other lifestyle factors through behavioural and psychological mechanisms, such as self-regulation and discipline. These central behaviours not only predict overall lifestyle patterns but also serve as catalysts for broader modifications, suggesting that interventions targeting these areas could significantly influence entire lifestyle networks. Globally, effective strategies to address these dietary challenges could include enhancing nutritional education and awareness, improving food accessibility and affordability, and subsidising healthy foods or taxing unhealthy ones to leverage diet's centrality for holistic improvements in overall healthy lifestyles.

In 28 health outcome networks, we identified four primary categories of central health outcomes: physical health (including sleep quality), emotional distress, social support, and overall quality of life. Social support received was central in Indonesia, Malaysia, and the Philippines, reflecting their collective cultural values [35,36]. Physical health was prominent in Burundi, highlighting direct concerns with bodily well-being, while sleep quality was central in Egypt, Guatemala, Saudi Arabia, and Thailand, possibly due to hot climates affecting sleep [37]. Emotional distress or mental burden emerged as central health concerns in countries including Canada, Hong Kong, India, Lebanon, Libya, Macau, Nigeria, Republic of Sudan, Rwanda, Singapore, South Africa, South Korea, UK, and Vietnam, driven by diverse factors. Specifically, political turmoil and economic instability significantly impacted Lebanon, Libya, Sudan, and Nigeria [38], while rapid urbanisation and economic stress affected Singapore, Hong Kong, South Korea, Macau, and others [39]. Quality of life, a multidimensional outcome encompassing physical health, mental health, and social relationships [40], was central in Brazil, Chile, India, Italy, Mainland China, Mexico, and the USA. This highlights the varied needs for health outcome interventions in each country-targeting physical, psychological, and social factors beyond lifestyle changes-to effectively improve holistic health.

In 19 countries with an interpretable bridge network, our study identified three key bridge lifestyles significantly affecting overall health outcomes: daily food types in Libya, home cooking in Nigeria, and exercise in other nations. In the context of network analysis, bridge lifestyle variables serve as critical connectors between the lifestyle network and the health outcomes network. These variables exhibit strong influence within the lifestyle network while simultaneously linking strongly to health outcome networks. Targeting these bridges in interventions can produce widespread, synergistic benefits by improving both the variables themselves and associated lifestyle behaviours, leading to enhanced overall health outcomes. Libya's reliance on specific meal types, influenced by its desert climate, heavy reliance on food imports, and political instability, highlights the critical role of dietary choices in health outcomes [41,42]. In Nigeria, home cooking may influence health through nutritional control over diets and economic relief via cost-effectiveness [43,44]. All remaining 17 countries or regions had exercise as their bridge lifestyle. The well-documented benefits of exercise on mental and physical health may explain this consistency across various nations [45], suggesting that strategic enhancements in exercise accessibility could lead to substantial health improvements. Globally, effective strategies to improve exercise accessibility include enhancing public infrastructure, implementing workplace wellness programmes, promoting community initiatives, integrating physical activity into urban planning, conducting education campaigns, ensuring inclusivity, partnering with health care providers, leveraging digital platforms, and supporting research and monitoring. Given the well-established causal relationship between lifestyle factors and health outcomes, targeted resource allocation and intensified interventions on bridge lifestyles are crucial for optimising these modifications across diverse settings.

This study identifies target points requiring increased resources and targeted interventions but does not specify which kinds of strategies are most effective. It assumes countries will adopt context-proven strategies. While comprehensive interventions for all lifestyle factors are ideal, they may only be feasible in resource-rich settings. Network theory suggests that prioritising central lifestyles, key health outcomes, and bridging lifestyles achieves greater health improvements than addressing other factors alone, which is particularly useful for resource-limited settings. For example, high-income Macau, middle-income mainland China, and low-income Sudan all identify home cooking as a central lifestyle factor. In resource-rich Macau, allocating substantial resources to home cooking can enhance other lifestyles, while additional resources can directly target other factors. In low-resource Sudan, dedicating a higher proportion of resources to home cooking can maximise impact. Similarly, targeting central health outcomes and bridging lifestyles can holistically improve health outcomes. Policymakers should design cost-effective programmes focused on these key variables and adopt context-specific interventions.

### Limitations

Several limitations were noted. First, our online recruitment might cause selection bias, underrepresenting individuals with low socio-economic status and limited digital literacy, possibly skewing the relationships between lifestyle and health outcomes. Future studies should use stratified sampling and offline data collection to improve representativeness and validate findings. Second, our reliance on self-reported data subject to recall bias, social desirability bias, and inaccurate self-assessment, potentially distorting the observed relationships between variables in the network. Although mitigated by validation questions and strict data quality controls, these limitations necessitate caution when interpreting the network structures and their implications. Future research should incorporate more accurate assessments, such as wearable technology or 24-hour recall, to enhance validity. Third, methodological limitations inherent in network analysis, such as assumptions of sparsity and potential overfitting, may affect the stability and generalisability of the identified network structures. The network estimation techniques rely on statistical models that may not fully capture the complex interplay between variables, and small changes in data can lead to different network configurations. Therefore, our network analysis results should be interpreted with consideration of these methodological constraints. Fourth, partial correlations within networks were based on individual perceptions of lifestyle and health outcome changes driven by the external factor of the COVID-19 pandemic. Although we anticipate these changes will revert in equal magnitude but opposite direction, preserving the relationships between variables post-pandemic, the findings require future validation during non-pandemic periods. Fifth, the cross-sectional design of our study during the COVID-19 pandemic limits our findings to associative insights and precludes the analysis of temporal network dynamics. By measuring outcomes relative to pre-pandemic conditions rather than their current status, the study's applicability to the post-pandemic period may be constrained. However, if we posit a rebound effect of equal magnitude and opposite direction, the findings could retain their relevance in a post-pandemic context. To substantiate and expand upon these insights, we recommend conducting longitudinal studies and randomised controlled trials post-pandemic. Lastly, small sample sizes in several countries limited network interpretability and robust comparisons, especially concerning bridge lifestyles. Increasing sample sizes in future research could address this issue.

## CONCLUSIONS

This study delineated central lifestyle, health outcome, and bridge lifestyle variables across 29 countries. Modifications in these identified variables hold the potential to significantly improve holistic lifestyle and holistic health outcomes, as well as holistic health outcomes via lifestyle, respectively. Their substantial impact supports the need for targeted interventions and increased resource allocation to achieve effective, comprehensive health improvements. The variation in these variables across different countries-shaped by diverse cultural, socio-demographic, political, and economic factors-emphasises the importance of customised strategies that exploit the unique effects of central or bridge lifestyles. Additionally, the similarities observed among some countries suggest opportunities for collaborative initiatives to promote global health.

## Additional material


Online Supplementary Document
