# Supplementary material for: Country-specific key lifestyle factors and health outcomes for resource allocation in the general population: a network analysis across 29 countries
Source: J Glob Health. 2025 Jan 10;15:04011. doi: 10.7189/jogh.15.04011 (PMC11719263; doi:10.7189/jogh.15.04011)
Supplement: Online Supplementary Document [file jogh-15-04011-s001.pdf]

**ONLINE SUPPLEMENTARY DOCUMENT**

**Title:** Country-Specific Key Lifestyle Factors and Health Outcomes for Resource Allocation in the General Population: A Network Analysis Across 29 Countries

**Authors:** Jiaying Li, Daniel Yee Tak Fong, Kris Yuet Wan Lok, Janet Yuen Ha Wong, Mandy Man Ho, Edmond Pui Hang Choi, Vinciya Pandian, Patricia M Davidson, Wenjie Duan, Marie Tarrant, Jung Jae Lee, Chia-Chin Lin, Oluwadamilare Akingbade, Khalid M Alabdulwahhab, Mohammad Shakil Ahmad, Mohamed Alboraie, Meshari A Alzahrani, Anil S Bilimale, Sawitree Boonpatcharanon, Samuel Byiringiro, Muhammad Kamil Che Hasan, Luisa Clausi Schettini, Walter Corzo, Josephine M. De Leon, Anjanette S. De Leon, Hiba Deek, Fabio Efficace, Mayssah A El Nayal, Fathiya El-Raey, Eduardo Ensaldo-Carrasco, Pilar Escotorin, Oluwadamilola Agnes Fadodun, Israel Opeyemi Fawole, Yong-Shian Shawn Goh, Devi Irawan, Naimah Ebrahim Khan, Binu Koirala, Ashish Krishna, Cannas Kwok, Tung Thanh Le, Daniela Giambruno Leal, Miguel Ángel Lezana-Fernández, Emery Manirambona, Leandro Cruz Mantoani, Fernando Meneses-González, Iman Elmahdi Mohamed, Madeleine Mukeshimana, Chinh Thi Minh Nguyen, Huong Thi Thanh Nguyen, Khanh Thi Nguyen, Son Truong Nguyen, Mohd Said Nurumal, Aimable Nzabonimana, Nagla Abdelrahim Mohamed Ahmed Omer, Oluwabunmi Ogungbe, Angela Chiu Yin Poon, Areli Reséndiz-Rodriguez, Busayasachee Puang-Ngern, Ceryl G Sagun, Riyaz Ahmed Shaik, Nikhil Gauri Shankar, Kathrin Sommer, Edgardo Toro, Hanh Thi Hong Tran, Elvira L Urgel, Emmanuel Uwiringiyimana, Tita Vanichbuncha, Naglaa Youssef

**Supplementary materials**


| eTables or eFigures | Page number |
|---|---|
| **Table S1.** Means and standard deviations of lifestyle factors and health outcomes across 29 countries | 2-6 |
| **Table S2.** Partial correlation matrix of variables in lifestyles network across 29 countries. | 7-35 |
| **Table S3.** Partial correlation matrix of variables in health outcomes network across 29 countries. | 36-64 |
| **Table S4**. Partial correlation matrix of variables in bridge network of lifestyles and health outcomes across 29 countries. | 65-93 |
| **Figure S1.** Bootstrapped confidence intervals of edge weights for the lifestyles network across 29 countries. | 94-97 |
| **Figure S2.** The stability of expected influence centrality index in lifestyles network across 29 countries using case-dropping bootstrap. | 98-99 |
| **Figure S3.** Network structure of lifestyle (A), health outcomes (B), and bridge that combined two (C) across 29 countries. | 100-128 |
| **Figure S4.** Centrality difference test of lifestyle, health outcomes, and bridge networks across 29 countries. | 129-157 |
| **Figure S5.** Bootstrapped confidence intervals of edge weights for the health outcome network across 29 countries. | 158-161 |
| **Figure S6.** The stability of expected influence centrality index in health outcomes network across 29 countries using case-dropping bootstrap. | 162-163 |
| **Figure S7.** Bootstrapped confidence intervals of edge weights for the bridge network across 29 countries. | 164-165 |
| **Figure S8.** The stability of bridge expected influence centrality index in bridge network across 29 countries using case-dropping bootstrap. | 166-167 |

**Table S1.** Means and standard deviations of lifestyle factors and health outcomes across 29 countries.

| Lifestyles and health outcomes | Australia | Brazil | Burundi | Canada | Chile | Egypt | Guatemala | Hong Kong | India | Indonesia | Italy | Lebanon | Libya | Macau | Mainland China |
|---|---|---|---|---|---|---|---|---|---|---|---|---|---|---|---|
| Food types in daily meals (L1) | 2.69 (0.75) | 3.22 (0.90) | 2.55 (0.97) | 3.01 (0.85) | 3.47 (0.94) | 2.95 (0.78) | 3.24 (0.95) | 3.15 (0.70) | 3.07 (0.80) | 2.95 (0.98) | 3.20 (0.68) | 2.88 (0.93) | 3.03 (0.86) | 2.94 (0.63) | 2.95 (0.79) |
| Consumption of fruits and vegetables (L2) | 2.29 (0.72) | 3.15 (0.89) | 2.65 (0.92) | 2.92 (0.85) | 3.33 (0.99) | 3.14 (0.89) | 3.30 (1.00) | 3.25 (0.69) | 3.46 (0.82) | 3.05 (1.01) | 3.19 (0.69) | 3.03 (0.92) | 3.37 (0.96) | 3.24 (0.62) | 3.11 (0.89) |
| Less consumption of frozen food/food products (L3) | 2.99 (0.93) | 2.77 (1.01) | 2.49 (0.87) | 3.16 (0.76) | 3.17 (0.90) | 2.34 (0.98) | 2.90 (1.03) | 3.42 (0.75) | 2.53 (1.01) | 2.75 (1.01) | 2.91 (0.87) | 2.55 (0.95) | 2.66 (0.95) | 3.23 (0.78) | 2.65 (0.94) |
| Less consumption of snacks (L4) | 3.43 (0.69) | 2.76 (1.11) | 2.78 (0.89) | 3.43 (0.90) | 3.17 (1.25) | 2.46 (0.98) | 2.95 (1.21) | 3.14 (0.82) | 2.67 (1.01) | 2.84 (0.98) | 2.87 (0.99) | 2.71 (1.04) | 2.58 (1.14) | 3.02 (0.84) | 2.71 (0.93) |
| Less soft drinks/juices/other sugary drinks (L5) | 3.44 (0.79) | 2.85 (1.15) | 2.57 (0.90) | 2.97 (0.76) | 2.96 (1.19) | 2.13 (1.05) | 2.72 (1.19) | 3.13 (0.81) | 2.29 (1.06) | 2.87 (0.98) | 2.78 (0.91) | 2.43 (1.11) | 2.42 (1.11) | 2.89 (0.73) | 2.68 (0.92) |
| Having a meal at home (L6) | 3.99 (0.79) | 4.06 (0.96) | 3.01 (0.93) | 3.84 (0.95) | 4.49 (0.84) | 3.55 (0.98) | 4.23 (1.02) | 3.83 (0.81) | 3.78 (0.97) | 3.33 (0.97) | 3.70 (0.91) | 3.59 (1.01) | 3.63 (1.05) | 3.78 (0.92) | 3.88 (1.05) |
| Cooking at home (L7) | 4.05 (0.61) | 3.98 (1.01) | 3.04 (0.87) | 3.70 (0.93) | 4.28 (0.96) | 3.57 (1.00) | 4.14 (1.01) | 3.81 (0.80) | 3.82 (0.98) | 3.28 (0.96) | 3.86 (0.89) | 3.60 (1.00) | 3.66 (1.05) | 3.69 (0.90) | 3.86 (1.07) |
| Less eating takeout food (L8) | 3.09 (1.07) | 3.28 (1.14) | 2.59 (0.87) | 2.85 (1.16) | 2.82 (1.34) | 2.02 (1.03) | 2.81 (1.25) | 3.41 (0.87) | 2.27 (1.09) | 2.83 (1.03) | 3.01 (1.02) | 2.08 (1.10) | 2.32 (1.10) | 3.27 (0.96) | 2.26 (1.06) |
| Taking alternative medicine or natural health products (L9) | 2.26 (0.50) | 3.27 (0.86) | 2.88 (0.89) | 3.07 (0.57) | 3.31 (0.88) | 2.51 (1.10) | 3.30 (1.10) | 3.01 (0.48) | 2.84 (1.03) | 2.83 (0.89) | 3.07 (0.54) | 2.58 (1.04) | 3.11 (1.17) | 2.92 (0.62) | 2.83 (0.77) |
| Taking oral supplements/vitamins (L10) | 2.91 (1.02) | 3.43 (0.86) | 2.71 (0.90) | 3.30 (0.67) | 3.22 (0.91) | 2.67 (1.20) | 3.45 (1.03) | 3.15 (0.52) | 3.04 (0.98) | 2.99 (1.00) | 3.16 (0.63) | 2.62 (1.10) | 3.00 (1.22) | 3.11 (0.62) | 2.88 (0.77) |
| Less smoking tobacco (L11) | 3.37 (0.73) | 2.70 (0.88) | 3.01 (0.57) | 2.96 (0.58) | 2.82 (1.07) | 2.01 (1.08) | 2.31 (1.01) | 3.01 (0.45) | 2.46 (0.95) | 2.89 (0.88) | 2.81 (0.72) | 2.56 (1.09) | 2.37 (1.08) | 2.81 (0.62) | 2.64 (0.83) |
| Less alcohol consumption (L12) | 3.29 (0.70) | 2.88 (0.95) | 3.13 (0.91) | 3.05 (0.86) | 2.98 (1.07) | 1.87 (1.01) | 2.48 (1.13) | 2.90 (0.57) | 2.46 (0.91) | 2.89 (0.93) | 2.86 (0.78) | 2.19 (1.00) | 2.19 (0.99) | 2.84 (0.70) | 2.63 (0.82) |
| Less duration of sitting (L13) | 4.30 (0.66) | 4.03 (0.99) | 3.08 (0.98) | 4.07 (0.87) | 4.41 (0.87) | 3.08 (1.08) | 4.12 (0.99) | 3.61 (0.74) | 3.51 (1.00) | 3.15 (1.04) | 3.96 (0.84) | 3.52 (1.08) | 3.61 (1.03) | 3.63 (0.87) | 3.64 (0.98) |
| Less duration of screen time (L14) | 4.21 (0.57) | 4.14 (0.96) | 2.90 (1.05) | 4.15 (0.81) | 4.55 (0.80) | 3.11 (1.10) | 4.31 (0.89) | 3.61 (0.74) | 3.58 (0.96) | 3.31 (1.09) | 4.00 (0.79) | 3.45 (1.14) | 3.48 (1.12) | 3.78 (0.80) | 3.84 (0.97) |

| | | | | | | | | | | | | | | | |
|---|---|---|---|---|---|---|---|---|---|---|---|---|---|---|---|
| Frequency of exercise (L15) | 3.27 | 2.59 | 3.01 | 2.44 | 2.30 | 2.41 | 2.74 | 2.76 | 3.01 | 2.94 | 2.54 | 2.59 | 2.80 | 2.94 | 2.77 |
| | (1.04) | (1.32) | (1.03) | (1.14) | (1.27) | (1.05) | (1.30) | (0.79) | (1.02) | (1.07) | (1.14) | (1.12) | (1.06) | (0.90) | (1.07) |
| Duration of exercise (L16) | 3.26 | 2.59 | 2.76 | 2.46 | 2.28 | 2.33 | 2.75 | 2.76 | 3.03 | 2.88 | 2.47 | 2.54 | 2.75 | 2.92 | 2.76 |
| | (0.93) | (1.25) | (1.06) | (1.12) | (1.26) | (1.06) | (1.31) | (0.78) | (1.02) | (1.01) | (1.11) | (1.10) | (1.08) | (0.88) | (1.08) |
| Type of exercise (L17) | 3.12 | 2.64 | 3.00 | 2.49 | 2.32 | 2.38 | 2.75 | 2.74 | 3.02 | 2.87 | 2.55 | 2.58 | 2.71 | 2.89 | 2.67 |
| | (0.93) | (1.22) | (0.99) | (1.08) | (1.26) | (1.06) | (1.32) | (0.72) | (0.95) | (0.95) | (1.11) | (1.09) | (1.02) | (0.83) | (1.02) |
| Overall amount of exercise (L18) | 3.15 | 2.60 | 2.98 | 2.41 | 2.28 | 2.38 | 2.69 | 2.72 | 2.98 | 2.90 | 2.53 | 2.59 | 2.76 | 2.91 | 2.71 |
| | (0.96) | (1.29) | (0.95) | (1.15) | (1.27) | (1.05) | (1.31) | (0.77) | (1.02) | (0.96) | (1.20) | (1.11) | (1.06) | (0.88) | (1.07) |
| Lose weight (H1) | 3.71 | 3.30 | 2.61 | 3.42 | 3.60 | 3.00 | 3.29 | 3.29 | 3.17 | 2.81 | 3.29 | 2.95 | 3.13 | 3.20 | 3.16 |
| | (0.71) | (1.00) | (1.01) | (0.89) | (1.05) | (0.93) | (1.04) | (0.63) | (0.83) | (1.02) | (0.81) | (0.96) | (0.99) | (0.85) | (0.89) |
| Appetite (H2) | 3.36 | 3.41 | 3.13 | 3.10 | 3.63 | 2.85 | 3.32 | 3.01 | 3.09 | 2.90 | 3.18 | 2.99 | 3.08 | 3.04 | 3.03 |
| | (0.59) | (0.93) | (0.88) | (0.84) | (1.00) | (0.89) | (0.97) | (0.54) | (0.73) | (0.94) | (0.73) | (1.07) | (1.04) | (0.59) | (0.78) |
| Physical health (H3) | 2.32 | 2.79 | 3.00 | 2.64 | 2.79 | 2.59 | 2.97 | 2.96 | 3.12 | 2.88 | 2.79 | 2.75 | 2.91 | 2.91 | 2.96 |
| | (0.64) | (0.99) | (0.94) | (0.85) | (1.09) | (0.86) | (0.99) | (0.51) | (0.70) | (0.87) | (0.67) | (0.86) | (0.86) | (0.53) | (0.63) |
| Sleep quality (H4) | 2.76 | 2.54 | 3.04 | 2.44 | 2.60 | 2.55 | 2.70 | 2.90 | 3.16 | 3.06 | 2.61 | 2.68 | 2.88 | 2.82 | 2.96 |
| | (0.88) | (1.02) | (0.90) | (0.97) | (1.25) | (1.01) | (1.17) | (0.65) | (0.91) | (1.05) | (0.92) | (1.13) | (1.13) | (0.73) | (0.78) |
| Quality of life (H5) | 2.17 | 2.54 | 2.82 | 2.12 | 2.75 | 2.49 | 2.92 | 2.78 | 3.04 | 3.01 | 2.31 | 2.28 | 2.55 | 2.81 | 2.91 |
| | (0.69) | (1.03) | (0.87) | (1.08) | (1.18) | (0.96) | (1.10) | (0.68) | (0.89) | (1.00) | (0.97) | (1.05) | (1.04) | (0.65) | (0.72) |
| Less mental burden (H6) | 4.00 | 4.00 | 3.18 | 3.90 | 4.32 | 3.21 | 3.98 | 3.34 | 3.40 | 3.02 | 3.68 | 3.38 | 3.31 | 3.43 | 3.36 |
| | (0.74) | (1.14) | (0.98) | (1.11) | (0.96) | (1.18) | (1.10) | (0.70) | (0.90) | (1.01) | (0.97) | (1.27) | (1.16) | (0.72) | (0.79) |
| Less emotional distress (H7) | 3.98 | 4.17 | 3.38 | 3.85 | 4.32 | 2.92 | 4.10 | 3.26 | 3.34 | 3.02 | 3.71 | 3.11 | 3.21 | 3.33 | 3.39 |
| | (0.87) | (1.00) | (1.10) | (1.02) | (0.90) | (1.15) | (1.00) | (0.64) | (0.91) | (1.05) | (0.80) | (1.16) | (1.14) | (0.66) | (0.77) |
| Family disputes (H8) | 3.94 | 3.29 | 3.43 | 3.31 | 3.29 | 2.79 | 3.37 | 3.13 | 3.12 | 2.91 | 3.19 | 3.12 | 3.18 | 3.13 | 3.19 |
| | (0.76) | (0.81) | (1.04) | (0.81) | (0.97) | (1.05) | (0.95) | (0.51) | (0.83) | (0.83) | (0.74) | (1.08) | (1.06) | (0.52) | (0.71) |
| Social support provided (H9) | 2.66 | 3.12 | 3.17 | 2.57 | 3.44 | 3.26 | 3.41 | 3.02 | 3.09 | 2.97 | 3.11 | 3.34 | 3.30 | 3.00 | 3.08 |
| | (1.12) | (0.89) | (1.04) | (1.04) | (1.01) | (0.99) | (1.03) | (0.40) | (0.73) | (0.90) | (0.83) | (1.06) | (1.07) | (0.43) | (0.64) |
| Social support received (H10) | 2.92 | 2.95 | 3.12 | 2.41 | 2.95 | 2.71 | 2.98 | 3.07 | 2.96 | 2.95 | 2.98 | 2.82 | 2.94 | 3.04 | 3.03 |
| | (1.05) | (0.83) | (1.01) | (0.99) | (0.99) | (0.98) | (0.94) | (0.43) | (0.75) | (0.89) | (0.64) | (0.98) | (1.00) | (0.44) | (0.64) |
| Social activities (H11) | 2.08 | 1.71 | 2.88 | 1.47 | 1.70 | 2.42 | 1.84 | 2.26 | 2.65 | 3.05 | 1.78 | 2.31 | 2.52 | 2.49 | 2.46 |
| | (0.75) | (1.00) | (0.99) | (0.83) | (1.11) | (1.04) | (1.14) | (0.94) | (0.98) | (1.00) | (1.03) | (1.11) | (1.10) | (0.78) | (0.90) |
| Income (H12) | 2.05 | 2.67 | 2.82 | 2.67 | 2.74 | 2.44 | 2.62 | 2.59 | 2.51 | 3.16 | 2.77 | 2.26 | 2.48 | 2.78 | 2.58 |

| | | | | | | | | | | | | | | | |
|---|---|---|---|---|---|---|---|---|---|---|---|---|---|---|---|
| | (0.92) | (0.94) | (1.03) | (0.95) | (1.01) | (0.93) | (1.04) | (0.73) | (0.83) | (0.99) | (0.72) | (1.01) | (0.99) | (0.70) | (0.82) |
| Less economic burden (H13) | 3.85 | 3.51 | 3.05 | 3.28 | 3.34 | 3.05 | 3.37 | 3.27 | 3.29 | 3.12 | 3.03 | 3.29 | 3.06 | 3.35 | 3.44 |
| | (0.92) | (0.91) | (1.06) | (0.95) | (1.02) | (1.15) | (1.08) | (0.72) | (0.88) | (0.97) | (0.77) | (1.36) | (1.14) | (0.68) | (0.90) |

(Continued)

| **Lifestyles and health outcomes** | **Malaysia** | **Mexico** | **Nigeria** | **Philippines** | **Republic Of Sudan** | **Rwanda** | **Saudi Arabia** | **Singapore** | **South Africa** | **South Korea** | **Thailand** | **United Kingdom** | **United States** | **Vietnam** |
|---|---|---|---|---|---|---|---|---|---|---|---|---|---|---|
| Food types in daily meals (L1) | 2.97 (0.81) | 3.19 (1.00) | 2.81 (1.00) | 3.23 (1.11) | 2.93 (0.87) | 2.56 (1.02) | 3.05 (1.00) | 2.96 (0.76) | 3.05 (0.84) | 2.85 (0.78) | 2.95 (0.82) | 3.17 (0.91) | 3.09 (0.83) | 3.16 (0.70) |
| Consumption of fruits and vegetables (L2) | 3.11 (0.87) | 3.44 (0.93) | 3.26 (1.10) | 3.35 (1.10) | 3.31 (0.99) | 2.68 (1.21) | 3.22 (0.98) | 3.13 (0.71) | 3.27 (0.90) | 3.05 (0.78) | 3.07 (0.78) | 3.18 (0.85) | 3.15 (0.77) | 3.37 (0.80) |
| Less consumption of frozen food/food products (L3) | 3.01 (0.91) | 2.80 (0.99) | 2.63 (1.00) | 3.25 (1.09) | 2.75 (0.98) | 2.60 (1.22) | 3.01 (1.00) | 3.34 (0.77) | 3.16 (0.74) | 3.53 (0.76) | 2.93 (0.98) | 3.18 (0.84) | 3.14 (0.79) | 2.99 (0.87) |
| Less consumption of snacks (L4) | 3.02 (0.96) | 2.77 (1.21) | 2.70 (0.99) | 3.05 (1.18) | 2.74 (1.10) | 2.70 (1.09) | 2.91 (1.11) | 3.27 (0.90) | 3.41 (1.08) | 3.18 (0.80) | 2.76 (0.93) | 3.48 (0.97) | 3.33 (0.89) | 2.98 (0.87) |
| Less soft drinks/juices/other sugary drinks (L5) | 2.75 (1.01) | 2.56 (1.17) | 2.68 (1.06) | 2.76 (1.27) | 2.51 (1.13) | 2.47 (1.13) | 2.59 (1.23) | 2.90 (0.86) | 2.95 (1.11) | 3.05 (0.79) | 2.84 (0.98) | 3.01 (1.02) | 2.91 (0.78) | 2.59 (0.96) |
| Having a meal at home (L6) | 3.64 (1.06) | 4.13 (1.01) | 3.54 (1.10) | 3.86 (1.16) | 3.73 (1.16) | 3.52 (1.18) | 3.96 (1.11) | 4.07 (0.97) | 3.92 (0.98) | 4.13 (0.77) | 3.83 (0.92) | 3.93 (1.04) | 4.22 (0.89) | 3.69 (0.95) |
| Cooking at home (L7) | 3.71 (1.05) | 4.04 (1.03) | 3.57 (1.12) | 3.86 (1.15) | 3.76 (1.16) | 3.42 (1.15) | 4.00 (1.07) | 3.84 (0.98) | 3.84 (0.99) | 3.98 (0.79) | 3.49 (1.01) | 3.83 (1.04) | 4.12 (0.93) | 3.66 (1.01) |
| Less eating takeout food (L8) | 2.90 (1.12) | 2.84 (1.29) | 2.44 (1.04) | 2.73 (1.32) | 2.26 (1.13) | 2.42 (1.11) | 2.54 (1.29) | 3.34 (1.18) | 2.36 (1.20) | 3.70 (0.91) | 3.53 (1.00) | 3.09 (1.23) | 2.99 (1.18) | 3.23 (1.06) |
| Taking alternative medicine or natural health products (L9) | 2.72 (0.97) | 3.18 (0.99) | 2.52 (1.09) | 2.76 (1.06) | 3.18 (1.16) | 2.81 (0.96) | 2.88 (1.17) | 2.84 (0.68) | 3.14 (0.99) | 3.02 (0.65) | 2.33 (0.99) | 2.89 (0.84) | 3.10 (0.62) | 2.65 (0.88) |
| Taking oral supplements/vitamins (L10) | 2.75 (0.97) | 3.40 (0.98) | 2.92 (1.03) | 3.22 (1.15) | 2.90 (1.12) | 2.64 (0.90) | 2.90 (1.13) | 3.15 (0.67) | 3.36 (0.99) | 3.28 (0.66) | 2.65 (1.03) | 3.35 (0.86) | 3.23 (0.69) | 2.73 (0.94) |
| Less smoking tobacco (L11) | 2.55 (0.95) | 2.42 (1.06) | 2.30 (1.03) | 2.22 (1.21) | 2.41 (1.01) | 2.55 (0.87) | 2.37 (1.15) | 2.85 (0.54) | 2.66 (0.86) | 2.66 (0.85) | 2.21 (1.00) | 2.76 (0.91) | 2.87 (0.60) | 2.27 (1.04) |
| Less alcohol consumption (L12) | 2.56 | 2.50 | 2.32 | 2.27 | 2.35 (1.01) | 2.61 | 2.33 | 2.81 | 2.68 | 2.57 | 2.31 | 2.78 | 3.21 | 2.28 |

| | | | | | | | | | | | | | | |
|---|---|---|---|---|---|---|---|---|---|---|---|---|---|---|
| | (0.93) | (1.05) | (1.01) | (1.23) | | (1.03) | (1.00) | (0.69) | (0.89) | (0.89) | (0.98) | (1.01) | (0.86) | (1.04) |
| Less duration of sitting (L13) | 3.66 (0.94) | 3.97 (1.06) | 3.24 (1.09) | 3.84 (1.03) | 3.44 (1.11) | 3.66 (1.17) | 3.64 (1.15) | 3.96 (0.93) | 3.88 (1.00) | 3.57 (0.73) | 3.42 (0.90) | 4.16 (0.82) | 4.14 (0.83) | 3.07 (0.95) |
| Less duration of screen time (L14) | 3.80 (0.99) | 4.10 (1.02) | 3.32 (1.08) | 4.00 (1.03) | 3.48 (1.16) | 3.73 (1.15) | 3.66 (1.12) | 4.03 (0.88) | 4.06 (0.98) | 3.77 (0.73) | 3.65 (0.89) | 4.24 (0.76) | 4.15 (0.80) | 3.26 (0.91) |
| Frequency of exercise (L15) | 2.98 (1.02) | 2.85 (1.27) | 3.09 (1.01) | 3.02 (1.27) | 2.89 (1.11) | 3.18 (1.20) | 2.92 (1.17) | 3.01 (1.17) | 2.92 (1.16) | 2.59 (0.97) | 2.85 (0.99) | 2.61 (1.21) | 2.70 (1.14) | 3.38 (0.88) |
| Duration of exercise (L16) | 2.95 (1.00) | 2.79 (1.28) | 3.04 (1.02) | 2.98 (1.24) | 2.86 (1.10) | 3.14 (1.24) | 2.87 (1.15) | 3.00 (1.10) | 2.80 (1.13) | 2.58 (0.96) | 2.81 (0.92) | 2.68 (1.26) | 2.72 (1.11) | 3.35 (0.88) |
| Type of exercise (L17) | 2.96 (0.95) | 2.78 (1.24) | 3.02 (0.96) | 2.95 (1.20) | 2.85 (1.05) | 3.13 (1.13) | 2.82 (1.13) | 3.05 (1.03) | 2.92 (1.09) | 2.54 (0.90) | 2.94 (0.95) | 2.73 (1.22) | 2.76 (1.13) | 3.26 (0.86) |
| Overall amount of exercise (L18) | 2.98 (1.02) | 2.74 (1.28) | 3.07 (1.02) | 3.04 (1.27) | 2.85 (1.09) | 3.10 (1.18) | 2.86 (1.16) | 3.01 (1.15) | 2.90 (1.22) | 2.54 (0.97) | 2.85 (0.93) | 2.63 (1.31) | 2.73 (1.23) | 3.28 (0.84) |
| Lose weight (H1) | 3.12 (0.93) | 3.25 (1.09) | 3.05 (0.90) | 3.31 (1.09) | 2.95 (0.92) | 3.33 (0.95) | 3.14 (1.04) | 3.22 (0.90) | 3.23 (1.02) | 3.24 (0.73) | 3.23 (0.77) | 3.45 (0.94) | 3.27 (0.91) | 3.20 (0.72) |
| Appetite (H2) | 3.25 (0.84) | 3.30 (0.95) | 3.11 (0.85) | 3.23 (1.01) | 2.96 (0.98) | 3.18 (0.84) | 3.14 (1.02) | 3.01 (0.70) | 3.22 (1.00) | 3.09 (0.69) | 3.22 (0.72) | 3.19 (0.91) | 3.26 (0.77) | 3.19 (0.62) |
| Physical health (H3) | 3.06 (0.74) | 2.99 (0.97) | 3.26 (0.88) | 3.27 (1.02) | 2.93 (0.85) | 2.95 (0.72) | 3.05 (0.91) | 2.95 (0.74) | 2.88 (0.85) | 2.76 (0.63) | 3.19 (0.68) | 2.82 (1.00) | 2.80 (0.78) | 3.22 (0.60) |
| Sleep quality (H4) | 3.10 (1.06) | 2.71 (1.19) | 3.25 (0.98) | 3.11 (1.21) | 3.05 (1.09) | 2.82 (0.99) | 2.97 (1.08) | 2.91 (0.90) | 2.84 (1.07) | 2.65 (0.71) | 3.25 (0.81) | 2.60 (1.01) | 2.56 (1.00) | 3.16 (0.73) |
| Quality of life (H5) | 3.08 (0.95) | 2.84 (1.04) | 3.18 (1.00) | 3.11 (1.10) | 2.63 (1.08) | 2.67 (0.86) | 2.90 (1.10) | 2.76 (0.90) | 2.80 (1.01) | 2.22 (0.81) | 3.41 (0.85) | 2.42 (1.05) | 2.51 (0.99) | 3.11 (0.74) |
| Less mental burden (H6) | 3.26 (0.96) | 3.97 (1.04) | 3.16 (1.09) | 3.39 (1.17) | 3.29 (1.20) | 3.37 (1.10) | 3.21 (1.18) | 3.48 (0.87) | 3.68 (1.04) | 2.88 (1.09) | 3.46 (0.89) | 3.74 (1.07) | 3.97 (0.96) | 3.06 (0.87) |
| Less emotional distress (H7) | 3.25 (0.95) | 4.08 (0.97) | 3.16 (1.05) | 3.36 (1.18) | 3.22 (1.15) | 3.56 (1.08) | 3.13 (1.09) | 3.39 (0.84) | 3.64 (1.05) | 2.86 (0.97) | 3.32 (0.92) | 3.64 (1.01) | 3.89 (0.93) | 2.95 (0.90) |
| Family disputes (H8) | 3.00 (0.77) | 3.32 (1.00) | 2.87 (1.02) | 2.79 (1.10) | 3.08 (1.09) | 2.82 (0.85) | 2.99 (1.07) | 3.24 (0.74) | 3.20 (0.87) | 2.92 (0.54) | 2.83 (0.86) | 3.14 (0.75) | 3.30 (0.75) | 2.72 (0.90) |
| Social support provided (H9) | 3.13 (0.80) | 3.31 (0.96) | 2.96 (1.01) | 3.10 (1.08) | 3.24 (1.09) | 3.16 (1.11) | 3.25 (0.99) | 3.09 (0.60) | 3.12 (0.82) | 3.02 (0.63) | 3.17 (0.78) | 2.98 (0.86) | 3.12 (0.91) | 2.83 (0.76) |

| | | | | | | | | | | | | | | |
|---|---|---|---|---|---|---|---|---|---|---|---|---|---|---|
| Social support received (H10) | 3.15 (0.81) | 2.91 (0.92) | 2.77 (1.03) | 3.08 (1.05) | 2.90 (1.01) | 2.80 (1.03) | 2.86 (0.94) | 3.04 (0.60) | 2.95 (0.84) | 3.16 (0.71) | 2.90 (0.81) | 2.79 (0.80) | 2.91 (0.87) | 2.87 (0.74) |
| Social activities (H11) | 2.93 (0.98) | 1.91 (1.15) | 2.69 (1.04) | 2.64 (1.18) | 2.55 (1.12) | 2.10 (1.17) | 2.57 (1.15) | 2.14 (0.86) | 2.09 (1.04) | 2.34 (0.78) | 3.05 (1.19) | 1.88 (1.01) | 1.63 (0.88) | 2.89 (0.79) |
| Income (H12) | 2.84 (0.80) | 2.61 (1.03) | 2.66 (1.00) | 2.69 (1.13) | 2.54 (1.03) | 2.41 (1.09) | 2.82 (0.94) | 2.81 (0.62) | 2.54 (0.87) | 2.65 (0.84) | 3.22 (0.90) | 2.73 (0.87) | 2.85 (0.71) | 2.63 (0.89) |
| Less economic burden (H13) | 3.21 (0.92) | 3.56 (0.99) | 3.46 (1.16) | 3.29 (1.19) | 3.11 (1.26) | 3.18 (1.32) | 3.01 (1.07) | 3.29 (0.63) | 3.43 (1.09) | 2.90 (0.95) | 3.43 (0.96) | 3.13 (0.80) | 3.09 (0.68) | 2.95 (0.98) |

**Table S2.** Partial correlation matrix of variables in lifestyles network across 29 countries.

**(A). Australia**

| | **L1** | **L2** | **L3** | **L4** | **L5** | **L6** | **L7** | **L8** | **L9** | **L10** | **L11** | **L12** | **L13** | **L14** |
|---|---|---|---|---|---|---|---|---|---|---|---|---|---|---|
| L1 | | | | | | | | | | | | | | |
| L2 | 0 | | | | | | | | | | | | | |
| L3 | 0.1765 | -0.1167 | | | | | | | | | | | | |
| L4 | 0 | 0 | 0.0954 | | | | | | | | | | | |
| L5 | -0.1637 | 0.097 | 0 | -0.1023 | | | | | | | | | | |
| L6 | 0 | 0 | 0 | 0.0409 | 0 | | | | | | | | | |
| L7 | -0.0551 | -0.0505 | -0.0313 | -0.0979 | 0 | 0.1217 | | | | | | | | |
| L8 | 0 | 0.1487 | -0.109 | 0.0755 | -0.0881 | 0.2065 | 0.0401 | | | | | | | |
| L9 | 0.2439 | 0.3653 | 0 | 0.0828 | 0.161 | 0.0433 | -0.026 | 0.0131 | | | | | | |
| L10 | -0.1355 | 0 | -0.0347 | -0.0415 | 0.0379 | -0.2394 | 0.1039 | -0.0265 | 0.2144 | | | | | |
| L11 | 0.072 | 0 | 0.0354 | 0 | 0.0185 | 0 | 0 | 0 | 0.1846 | 0.0134 | | | | |
| L12 | 0 | 0.0509 | 0 | 0 | 0 | 0 | 0.0156 | 0.0033 | 0.0443 | 0 | 0.1529 | | | |
| L13 | 0 | 0.1668 | 0 | 0.0606 | 0 | 0.0784 | -0.0857 | 0 | 0.1106 | 0.109 | 0.064 | 0.0403 | | |
| L14 | 0.2187 | 0.0403 | -0.0093 | 0 | 0.1658 | -0.158 | -0.0013 | 0.0513 | 0.0407 | -0.1008 | 0.0115 | 0 | 0.0824 | |
| L18 | 0.0279 | 0 | 0 | 0.004 | -0.0132 | 0 | 0 | -0.0412 | -0.037 | 0 | -0.0157 | 0 | 0 | 0 |

**(B). Brazil**

| | L1 | L2 | L3 | L4 | L5 | L6 | L7 | L8 | L9 | L10 | L11 | L12 | L13 | L14 |
|---|---|---|---|---|---|---|---|---|---|---|---|---|---|---|
| L1 | | | | | | | | | | | | | | |
| L2 | 0.0448 | | | | | | | | | | | | | |
| L3 | 0 | 0.0898 | | | | | | | | | | | | |
| L4 | 0 | 0.1707 | 0.2377 | | | | | | | | | | | |
| L5 | -0.085 | 0.027 | 0.1304 | 0.401 | | | | | | | | | | |
| L6 | 0.0567 | 0.0053 | 0 | 0 | 0 | | | | | | | | | |
| L7 | 0 | 0.0503 | 0.0428 | 0 | 0.0588 | 0.6261 | | | | | | | | |
| L8 | 0 | 0.0182 | 0.1178 | 0.1299 | 0.136 | 0 | 0.0262 | | | | | | | |
| L9 | 0.0446 | 0 | -0.0191 | -0.0368 | 0 | 0.014 | 0 | -0.0544 | | | | | | |
| L10 | 0 | 0.0701 | 0 | 0 | 0 | 0.0388 | 0 | 0 | 0.331 | | | | | |
| L11 | -0.012 | 0 | 0.0849 | 0.0396 | 0.0178 | 0 | 0 | 0.0154 | 0 | -0.017 | | | | |
| L12 | 0 | 0 | 0 | 0 | 0.0726 | 0 | 0 | 0.0642 | 0 | 0 | 0.335 | | | |
| L13 | -0.0075 | 0 | 0 | 0 | 0 | -0.0653 | 0 | 0 | 0 | 0 | 0 | 0 | | |
| L14 | 0 | 0 | 0 | 0.0022 | 0.0548 | -0.0709 | 0 | 0 | -0.0668 | 0 | 0.0477 | 0 | 0.6133 | |
| L18 | 0 | 0.1275 | 0 | 0.0145 | 0.081 | 0 | 0 | 0 | 0 | 0.0457 | 0 | -0.0356 | 0.0503 | 0 |

**(C). Burundi**

| | L1 | L2 | L3 | L4 | L5 | L6 | L7 | L8 | L9 | L10 | L11 | L12 | L13 | L14 |
|---|---|---|---|---|---|---|---|---|---|---|---|---|---|---|
| L1 | | | | | | | | | | | | | | |
| L2 | 0.4313 | | | | | | | | | | | | | |
| L3 | 0 | -0.1022 | | | | | | | | | | | | |
| L4 | 0 | -0.0036 | 0.2442 | | | | | | | | | | | |
| L5 | 0 | -0.0824 | 0 | 0.1767 | | | | | | | | | | |
| L6 | 0 | 0 | 0 | -0.091 | -0.0319 | | | | | | | | | |
| L7 | 0 | 0 | 0 | -0.08 | 0 | 0.2604 | | | | | | | | |
| L8 | -0.046 | 0 | 0 | 0 | 0.0246 | -0.0593 | -0.0314 | | | | | | | |
| L9 | 0 | 0.019 | -0.0096 | -0.1069 | 0 | 0.0834 | 0.0271 | -0.2175 | | | | | | |
| L10 | 0 | 0 | 0 | -0.022 | -0.106 | 0.0899 | 0.1249 | -0.0559 | 0.1023 | | | | | |
| L11 | -0.0839 | 0 | 0 | 0.0166 | 0 | 0 | 0 | 0.0247 | 0 | 0 | | | | |
| L12 | 0 | 0 | 0 | 0 | 0 | 0 | 0 | 0 | 0 | 0 | 0.1826 | | | |
| L13 | 0 | 0 | 0 | 0 | 0 | -0.0667 | -0.1097 | 0.0918 | -0.0126 | -0.0719 | 0 | 0 | | |
| L14 | 0 | 0 | 0.0292 | 0.0676 | 0.001 | -0.0089 | -0.134 | 0.0497 | -0.0418 | -0.1196 | 0 | 0 | 0.1128 | |
| L18 | 0 | 0 | 0 | 0 | 0 | 0 | 0.0452 | 0 | 0 | 0 | 0 | 0 | 0 | -0.0552 |

**(D). Canada**

| | L1 | L2 | L3 | L4 | L5 | L6 | L7 | L8 | L9 | L10 | L11 | L12 | L13 | L14 |
|---|---|---|---|---|---|---|---|---|---|---|---|---|---|---|
| L1 | | | | | | | | | | | | | | |
| L2 | 0.4519 | | | | | | | | | | | | | |
| L3 | 0 | 0.1171 | | | | | | | | | | | | |
| L4 | -0.0168 | 0 | 0.2607 | | | | | | | | | | | |
| L5 | 0 | 0 | 0 | 0.2536 | | | | | | | | | | |
| L6 | 0 | 0 | -0.0158 | 0 | 0 | | | | | | | | | |
| L7 | 0.0378 | 0 | 0 | 0 | 0.0071 | 0.6372 | | | | | | | | |
| L8 | 0 | 0 | 0.0083 | 0.0778 | 0.0265 | 0 | 0.1734 | | | | | | | |
| L9 | 0 | 0.0549 | 0 | 0 | 0 | 0 | 0 | 0 | | | | | | |
| L10 | 0 | 0.0723 | 0 | 0 | 0.0838 | 0.0351 | 0 | 0 | 0.2795 | | | | | |
| L11 | -0.036 | 0 | 0 | 0 | 0 | 0 | 0 | 0 | 0 | 0 | | | | |
| L12 | 0 | 0 | 0 | 0.0604 | 0.0654 | 0 | 0 | 0 | 0 | 0 | 0.2185 | | | |
| L13 | 0 | 0 | 0 | 0.0106 | 0.0125 | 0 | 0 | 0 | 0 | 0 | 0.0206 | 0.0412 | | |
| L14 | 0 | 0 | 0.023 | 0.1012 | 0 | -0.0758 | -0.0065 | 0 | 0 | 0 | 0 | 0 | 0.6828 | |
| L18 | 0 | 0.1107 | 0 | 0 | 0.0071 | 0 | 0 | 0 | 0 | 0 | 0 | 0 | 0.1542 | 0.1402 |

**(E). Chile**

| | L1 | L2 | L3 | L4 | L5 | L6 | L7 | L8 | L9 | L10 | L11 | L12 | L13 | L14 |
|---|---|---|---|---|---|---|---|---|---|---|---|---|---|---|
| L1 | | | | | | | | | | | | | | |
| L2 | 0.1158 | | | | | | | | | | | | | |
| L3 | -0.0626 | 0 | | | | | | | | | | | | |
| L4 | -0.1009 | 0.1699 | 0.1806 | | | | | | | | | | | |
| L5 | 0 | 0.0514 | 0 | 0.3557 | | | | | | | | | | |
| L6 | 0.0705 | 0 | 0 | 0 | 0 | | | | | | | | | |
| L7 | 0.0104 | 0.0265 | 0 | 0 | 0.0713 | 0.4813 | | | | | | | | |
| L8 | 0 | 0.0473 | 0.004 | 0.1558 | 0 | 0 | 0 | | | | | | | |
| L9 | 0 | 0 | 0 | 0 | 0 | 0.0339 | 0 | 0 | | | | | | |
| L10 | 0 | 0 | -0.0293 | 0 | 0 | 0 | 0 | 0 | 0.3612 | | | | | |
| L11 | 0 | 0.0725 | 0.0341 | 0.0268 | 0.0897 | 0 | 0 | 0.0207 | 0 | -0.1151 | | | | |
| L12 | -0.0251 | 0 | 0.0274 | 0.127 | 0 | 0 | 0 | 0.0753 | 0 | 0 | 0.3347 | | | |
| L13 | 0 | 0 | 0 | 0.0224 | 0 | -0.0091 | 0 | 0 | 0 | 0 | 0 | 0 | | |
| L14 | -0.0248 | 0 | 0 | 0.0341 | 0 | -0.0883 | -0.0443 | 0 | -0.0739 | 0 | 0 | 0 | 0.6724 | |
| L18 | 0 | 0.0456 | 0 | 0.0131 | 0.0313 | 0 | 0 | 0 | 0.0378 | 0 | 0 | 0 | 0.149 | 0 |

**(F). Egypt**

| | L1 | L2 | L3 | L4 | L5 | L6 | L7 | L8 | L9 | L10 | L11 | L12 | L13 | L14 |
|---|---|---|---|---|---|---|---|---|---|---|---|---|---|---|
| L1 | | | | | | | | | | | | | | |
| L2 | 0.2081 | | | | | | | | | | | | | |
| L3 | -0.0066 | 0 | | | | | | | | | | | | |
| L4 | -0.0608 | 0 | 0.2162 | | | | | | | | | | | |
| L5 | -0.011 | 0 | 0.228 | 0.1576 | | | | | | | | | | |
| L6 | 0.1347 | 0.0649 | 0.0477 | 0 | 0 | | | | | | | | | |
| L7 | 0 | 0.1141 | 0 | 0 | 0 | 0.6772 | | | | | | | | |
| L8 | -0.033 | 0 | 0.1053 | 0.1913 | 0.2319 | 0.0474 | 0.1232 | | | | | | | |
| L9 | 0.0607 | 0.0192 | 0 | -0.0265 | -0.051 | 0.0191 | 0.0076 | 0 | | | | | | |
| L10 | 0 | 0.1096 | 0 | -0.0027 | 0 | 0.0094 | 0.0254 | 0 | 0.209 | | | | | |
| L11 | 0 | 0 | 0.0032 | 0 | 0.0788 | 0.0021 | 0.0115 | 0.0234 | 0 | 0 | | | | |
| L12 | 0 | 0 | 0.0524 | 0.0034 | 0.011 | 0.0026 | 0.0256 | 0 | -0.0675 | -0.1401 | 0.6648 | | | |
| L13 | -0.0409 | 0 | 0 | 0 | 0 | -0.0361 | -0.0157 | 0 | -0.0387 | -0.0338 | 0.0357 | 0 | | |
| L14 | -0.0898 | 0 | 0.0645 | 0 | 0.0436 | -0.0115 | -0.0154 | 0 | -0.0266 | -0.0445 | 0.039 | 0 | 0.4913 | |
| L18 | 0 | 0.0728 | -0.1164 | -0.0501 | 0 | 0 | 0 | -0.0542 | 0.0941 | 0.0762 | 0 | -0.1391 | 0 | 0 |

**(G). Guatemala**

| | L1 | L2 | L3 | L4 | L5 | L6 | L7 | L8 | L9 | L10 | L11 | L12 | L13 | L14 |
|---|---|---|---|---|---|---|---|---|---|---|---|---|---|---|
| L1 | | | | | | | | | | | | | | |
| L2 | 0 | | | | | | | | | | | | | |
| L3 | 0 | 0 | | | | | | | | | | | | |
| L4 | -0.0191 | 0 | 0.203 | | | | | | | | | | | |
| L5 | 0 | 0.0144 | 0.0182 | 0.4173 | | | | | | | | | | |
| L6 | 0 | 0 | 0 | 0 | 0 | | | | | | | | | |
| L7 | 0 | 0 | 0 | 0 | 0.1004 | 0.58 | | | | | | | | |
| L8 | 0 | 0 | 0 | 0.0759 | 0.0236 | 0 | 0 | | | | | | | |
| L9 | 0 | 0 | 0 | 0 | 0 | 0 | 0 | 0 | | | | | | |
| L10 | 0 | 0 | 0 | 0 | 0 | 0 | 0 | 0 | 0.3747 | | | | | |
| L11 | 0 | 0 | 0 | 0 | 0 | 0 | 0 | 0 | 0 | 0 | | | | |
| L12 | 0 | 0 | 0 | 0 | 0.0547 | 0 | 0 | 0 | 0 | 0 | 0.4686 | | | |
| L13 | 0 | 0 | 0 | 0 | 0 | 0 | 0 | 0 | -0.0107 | 0 | 0 | 0 | | |
| L14 | 0 | 0 | 0 | 0 | 0 | -0.0961 | 0 | 0 | 0 | 0 | 0 | 0 | 0.418 | |
| L18 | 0 | 0.1073 | 0 | 0 | 0 | 0 | 0 | 0 | 0 | 0 | 0 | 0 | 0 | 0 |

**(H). Hong Kong**

| | L1 | L2 | L3 | L4 | L5 | L6 | L7 | L8 | L9 | L10 | L11 | L12 | L13 | L14 |
|---|---|---|---|---|---|---|---|---|---|---|---|---|---|---|
| L1 | | | | | | | | | | | | | | |
| L2 | 0.2202 | | | | | | | | | | | | | |
| L3 | 0 | -0.1107 | | | | | | | | | | | | |
| L4 | -0.1047 | 0.0834 | 0.1456 | | | | | | | | | | | |
| L5 | -0.0573 | 0.0699 | 0.0311 | 0.57 | | | | | | | | | | |
| L6 | -0.0085 | 0.086 | -0.0966 | 0.0238 | 0 | | | | | | | | | |
| L7 | 0 | 0.087 | -0.0017 | 0.0074 | 0.0339 | 0.6007 | | | | | | | | |
| L8 | 0 | 0.0315 | 0.0875 | 0.081 | 0.118 | -0.0199 | -6.00E-04 | | | | | | | |
| L9 | 0.0791 | 0 | 0 | 0 | 0 | 0 | 0 | -0.0439 | | | | | | |
| L10 | 0 | 0.0782 | -0.0282 | 0 | 0.0045 | 0.0086 | 0.0648 | -0.0484 | 0.1684 | | | | | |
| L11 | 0 | 0 | 0 | 0.0077 | 0.0453 | 0 | 0 | 0 | -0.0635 | -0.0629 | | | | |
| L12 | -0.0239 | 0 | -0.017 | 0.0068 | 0.0564 | 0.0386 | 0.021 | 0 | -0.0474 | -0.0202 | 0.4396 | | | |
| L13 | 0 | -0.0148 | 0.0528 | 0.0297 | 0 | -0.0642 | -0.0341 | 0.0649 | 0 | -0.0103 | 0 | 0 | | |
| L14 | 0 | -0.0031 | 0.0648 | 0 | 0.0107 | -0.0642 | -0.0542 | 0.1231 | 0 | -0.0696 | 0 | -0.0021 | 0.5651 | |
| L18 | 0.053 | 0 | 0.0299 | -0.0415 | -0.0334 | -0.0782 | -0.0096 | 0 | 0 | 0.0644 | 0 | -0.0999 | 0.0534 | 0.0802 |

**(I). India**

| | L1 | L2 | L3 | L4 | L5 | L6 | L7 | L8 | L9 | L10 | L11 | L12 | L13 | L14 |
|---|---|---|---|---|---|---|---|---|---|---|---|---|---|---|
| L1 | | | | | | | | | | | | | | |
| L2 | 0.1369 | | | | | | | | | | | | | |
| L3 | -0.0358 | -0.0209 | | | | | | | | | | | | |
| L4 | -0.1023 | 0 | 0.2224 | | | | | | | | | | | |
| L5 | 0 | 0 | 0.3281 | 0.2833 | | | | | | | | | | |
| L6 | 0.0202 | 0.1172 | 0 | 0 | 0.0185 | | | | | | | | | |
| L7 | 0.0562 | 0.0872 | 0 | 0 | 0.0027 | 0.5976 | | | | | | | | |
| L8 | 0 | 0 | 0.0841 | 0.0048 | 0.2525 | 0.1164 | 0.0378 | | | | | | | |
| L9 | 0 | 0 | 0 | 0 | -0.0413 | 0 | 0 | 0 | | | | | | |
| L10 | -0.0328 | 0.0846 | -0.0211 | 0 | -0.0392 | 0 | 0.021 | 0 | 0.4276 | | | | | |
| L11 | 0 | 0 | 0.017 | 0 | 0.1354 | 0 | 0.0245 | 0.0696 | -0.0091 | -0.0859 | | | | |
| L12 | 0.042 | 0.0094 | 0.0657 | 0 | 0.0158 | 0.0261 | 0.034 | 0 | -0.0615 | 0 | 0.7579 | | | |
| L13 | 0 | 0.0218 | 0 | 0.0479 | 0 | -0.1376 | 0 | -0.0864 | -0.033 | 0 | 0 | 0 | | |
| L14 | 0 | 0 | 0 | 0.0599 | -0.0323 | -0.0632 | 0 | -0.0324 | 0 | 0 | 0 | 0 | 0.5787 | |
| L18 | -0.0367 | 0.1615 | 0 | 0.0256 | 0 | 0.0278 | 0.047 | -0.0044 | 0.0319 | 0 | 0 | 0 | 0.0714 | 0.0398 |

**(J). Indonesia**

| | L1 | L2 | L3 | L4 | L5 | L6 | L7 | L8 | L9 | L10 | L11 | L12 | L13 | L14 |
|---|---|---|---|---|---|---|---|---|---|---|---|---|---|---|
| L1 | | | | | | | | | | | | | | |
| L2 | 0.2337 | | | | | | | | | | | | | |
| L3 | -0.108 | -0.0171 | | | | | | | | | | | | |
| L4 | -0.0835 | -0.0231 | 0.1723 | | | | | | | | | | | |
| L5 | 0 | 0 | 0.2324 | 0.364 | | | | | | | | | | |
| L6 | 0.1064 | 0.1653 | 0 | -0.0136 | -0.0159 | | | | | | | | | |
| L7 | 0.0959 | 0 | 0 | -0.0087 | 0 | 0.5397 | | | | | | | | |
| L8 | -0.0072 | -0.0275 | 0.0616 | 0.0626 | 0.0855 | -0.0765 | 0 | | | | | | | |
| L9 | 0.113 | 0 | -0.051 | -0.0675 | -0.0667 | 0.035 | 0.0155 | -0.2114 | | | | | | |
| L10 | 0.0069 | 0.0447 | -0.0931 | 0 | 0 | 0 | 0.0774 | -0.0824 | 0.1892 | | | | | |
| L11 | 0 | -0.0759 | 0.0102 | 0.0399 | 0.045 | -0.006 | 0 | 0 | 0 | -0.022 | | | | |
| L12 | -0.0174 | 0 | 0 | 0 | 0 | 0 | 0 | 0 | 0.0141 | -0.0843 | 0.7064 | | | |
| L13 | 0 | -0.0195 | 0.0412 | 0.0428 | 0 | 0 | 0 | 0.0346 | 0 | -0.0106 | 0.0468 | 0 | | |
| L14 | 0 | 0 | 0.0096 | 0.0917 | 0 | -0.0457 | -0.0235 | 0 | 0 | -0.0442 | 0.0011 | 0 | 0.4739 | |
| L18 | 0.1063 | 0.105 | -0.0135 | 0 | -0.0607 | 0.0502 | 0.0105 | -0.0609 | 0 | 0.115 | 0 | -0.0525 | -0.0785 | -0.0808 |

**(K). Italy**

| | L1 | L2 | L3 | L4 | L5 | L6 | L7 | L8 | L9 | L10 | L11 | L12 | L13 | L14 |
|---|---|---|---|---|---|---|---|---|---|---|---|---|---|---|
| L1 | | | | | | | | | | | | | | |
| L2 | 0 | | | | | | | | | | | | | |
| L3 | 0 | 0.1479 | | | | | | | | | | | | |
| L4 | -0.0518 | 0.0233 | 0.1621 | | | | | | | | | | | |
| L5 | -0.0092 | 0.0911 | 0.0498 | 0.3202 | | | | | | | | | | |
| L6 | 0 | 0 | 0 | 0 | 0 | | | | | | | | | |
| L7 | 0 | 0 | 0 | 0 | 0 | 0.493 | | | | | | | | |
| L8 | 0 | 0 | 0.1163 | 0.1389 | 0 | 0 | 0 | | | | | | | |
| L9 | 0 | 0 | 0 | 0 | 0 | 0 | 0 | 0 | | | | | | |
| L10 | 0 | 0 | 0 | 0 | 0 | 0 | 0 | 0 | 0.273 | | | | | |
| L11 | 0 | 0 | 0 | 0 | 0.0177 | 0 | 0 | 0.1613 | 0 | 0 | | | | |
| L12 | 0 | 0 | 0 | 0 | 0.1709 | 0 | 0 | 0 | 0 | 0 | 0.319 | | | |
| L13 | -0.0605 | 0 | 0 | 0 | 0 | -0.0048 | -0.0649 | 0.0383 | 0 | 0 | 0 | 0 | | |
| L14 | 0 | 0 | 0 | 0 | 0 | -0.0474 | 0 | 0 | 0 | 0 | 0 | 0 | 0.6449 | |
| L18 | 0 | 0.0545 | 0.0464 | 0 | 0 | 0 | 0 | 0 | 0 | 0 | 0 | 0 | 0.0952 | 0 |

**(L). Lebanon**

| | L1 | L2 | L3 | L4 | L5 | L6 | L7 | L8 | L9 | L10 | L11 | L12 | L13 | L14 |
|---|---|---|---|---|---|---|---|---|---|---|---|---|---|---|
| L1 | | | | | | | | | | | | | | |
| L2 | 0.2118 | | | | | | | | | | | | | |
| L3 | -0.1073 | -0.0553 | | | | | | | | | | | | |
| L4 | -0.1055 | -0.0117 | 0.154 | | | | | | | | | | | |
| L5 | -0.0504 | 0 | 0.1142 | 0.0935 | | | | | | | | | | |
| L6 | 0.075 | 0 | 0 | -0.064 | 0 | | | | | | | | | |
| L7 | 0 | 0.1702 | 0 | 0 | 0 | 0.5953 | | | | | | | | |
| L8 | -0.0624 | 0 | 0.1869 | 0.2021 | 0.2154 | 0.0165 | 0.1799 | | | | | | | |
| L9 | 0 | 0 | 0 | 0 | 0 | 0 | 0.0165 | 0 | | | | | | |
| L10 | 0 | 0.071 | -0.0539 | -0.1329 | 0 | 0.0195 | 0 | 0 | 0.2927 | | | | | |
| L11 | 0 | 0 | 0.0031 | 0 | 0.1464 | 0 | 0 | 0 | -0.0176 | -0.0279 | | | | |
| L12 | 0 | 0 | 0.1678 | 0 | 0 | 0.0855 | 0 | 0 | -0.2009 | -0.1406 | 0.292 | | | |
| L13 | 0 | -0.0081 | 0.0206 | 0.0283 | 0 | -0.0934 | 0 | 0 | -0.0142 | -0.0422 | 0.0498 | 0.0228 | | |
| L14 | 0 | 0 | 0.0229 | 0 | 0.0933 | 0 | -0.0736 | 0.0193 | 0 | -0.0363 | 0 | 0.0063 | 0.6805 | |
| L18 | 0.0253 | 0.1023 | 0 | -0.0619 | -0.0073 | 0 | 0.0493 | -0.0231 | 0.0467 | 0.087 | 0 | -0.0516 | 0.0943 | 0 |

**(M). Libya**

| | L1 | L2 | L3 | L4 | L5 | L6 | L7 | L8 | L9 | L10 | L11 | L12 | L13 | L14 |
|---|---|---|---|---|---|---|---|---|---|---|---|---|---|---|
| L1 | | | | | | | | | | | | | | |
| L2 | 0.2277 | | | | | | | | | | | | | |
| L3 | -0.1949 | 0 | | | | | | | | | | | | |
| L4 | -0.0702 | 0 | 0.1417 | | | | | | | | | | | |
| L5 | 0 | 0.0289 | 0.2025 | 0.3521 | | | | | | | | | | |
| L6 | 0.1029 | 0.1096 | 0.0195 | 0.0318 | 0.0071 | | | | | | | | | |
| L7 | 0.0123 | 0.0334 | 0 | 0.0189 | 0.0376 | 0.7107 | | | | | | | | |
| L8 | 0 | 0 | 0.0228 | 0.1167 | 0.0874 | 0 | 0.0208 | | | | | | | |
| L9 | 0.0371 | 0.0833 | 0 | 0.0076 | 0.0238 | 0.01 | 0.0379 | 0 | | | | | | |
| L10 | 0 | 0.091 | 0 | 0 | 0.0231 | 0.0087 | 0 | -0.0501 | 0.2794 | | | | | |
| L11 | 0.0261 | 0.04 | 0.0382 | 0 | 0 | 0 | 0.0446 | 0.0832 | 0 | 0 | | | | |
| L12 | 0 | 0.0077 | 0.0115 | 0 | 0.0716 | 0.0394 | 0.0194 | 0.1633 | -0.055 | -0.0441 | 0.5899 | | | |
| L13 | -0.0082 | 0 | 0 | 0 | 0.0331 | -0.1092 | -0.0253 | 0 | -0.0664 | -0.0353 | 0.0407 | 0 | | |
| L14 | 0 | 0 | 0.0273 | 0 | 0.073 | 0 | 0 | 0 | -0.073 | 0 | 0.0079 | 0.04 | 0.5277 | |
| L18 | 0 | 0.0862 | 0 | 0 | 0 | 0 | 0.0194 | -0.089 | 0 | 0.0753 | 0 | -0.0882 | 0 | 0 |

**(N). Macau**

| | L1 | L2 | L3 | L4 | L5 | L6 | L7 | L8 | L9 | L10 | L11 | L12 | L13 | L14 |
|---|---|---|---|---|---|---|---|---|---|---|---|---|---|---|
| L1 | | | | | | | | | | | | | | |
| L2 | 0.1843 | | | | | | | | | | | | | |
| L3 | 0 | 0 | | | | | | | | | | | | |
| L4 | 0 | 0 | 0.1678 | | | | | | | | | | | |
| L5 | 0 | 0.0949 | 0.0441 | 0.4876 | | | | | | | | | | |
| L6 | -0.0244 | 0 | -0.0259 | 0 | 0 | | | | | | | | | |
| L7 | 0 | 0.1012 | 0 | 0 | 0.033 | 0.7235 | | | | | | | | |
| L8 | 0 | 0 | 0.0832 | 0.0347 | 0.0756 | 0 | 0 | | | | | | | |
| L9 | 0.0141 | 0 | -0.068 | -0.0523 | 0 | 0 | 0 | 0 | | | | | | |
| L10 | 0 | 0 | 0 | 0 | 0 | 0.0548 | 0.0326 | 0 | 0.2298 | | | | | |
| L11 | 0 | 0.0789 | 0 | 0 | 0.0175 | 0 | 0 | 0 | -0.0136 | -0.0699 | | | | |
| L12 | 0.0248 | 0.037 | 0 | 0.0485 | 0.0481 | 0 | 0 | 0 | -0.1006 | 0 | 0.6433 | | | |
| L13 | 0.0623 | 0 | 0.0172 | 0.0622 | 0.0133 | -0.1001 | 0 | 0.1019 | 0 | 0 | 0 | 0.0082 | | |
| L14 | 0 | 0 | 0.0196 | 0.0281 | 0 | -0.0593 | 0 | 0 | 0 | 0 | 0 | 0 | 0.5652 | |
| L18 | 0 | 0 | 0 | 0 | 0 | 0 | 0 | 0 | 0.017 | 0 | 0 | -0.0345 | 0.1124 | 0 |

**(O). Mainland China**

| | L1 | L2 | L3 | L4 | L5 | L6 | L7 | L8 | L9 | L10 | L11 | L12 | L13 | L14 |
|---|---|---|---|---|---|---|---|---|---|---|---|---|---|---|
| L1 | | | | | | | | | | | | | | |
| L2 | 0.5718 | | | | | | | | | | | | | |
| L3 | -0.0326 | -0.0297 | | | | | | | | | | | | |
| L4 | -0.0316 | -0.0366 | 0.1952 | | | | | | | | | | | |
| L5 | 0 | 0 | 0.0901 | 0.4796 | | | | | | | | | | |
| L6 | 0 | 0.0737 | 0 | 0 | 0.0262 | | | | | | | | | |
| L7 | 0 | 0.0039 | 0 | 0 | 0 | 0.8486 | | | | | | | | |
| L8 | 0 | 0 | 0.1601 | 0.1317 | 0.1094 | 0.0206 | 0.1094 | | | | | | | |
| L9 | -0.0233 | 0 | 0 | 0.0195 | 0 | 0 | 0 | -0.0026 | | | | | | |
| L10 | 0 | 0.0783 | -0.062 | 0 | -0.0906 | 0 | 0.0236 | -0.0077 | 0.5112 | | | | | |
| L11 | 0 | 0 | 0 | 0.0621 | 0.0207 | 0 | 0 | 0 | -0.1086 | -0.0731 | | | | |
| L12 | 0 | 0 | 0 | 0 | 0 | 0.0146 | 0 | 0.094 | -0.1087 | 0 | 0.7451 | | | |
| L13 | 0 | 0.0084 | 0.0464 | 0.0042 | 0.0157 | -0.0027 | 0 | 0 | 0 | 0 | 0 | 0 | | |
| L14 | 0.0572 | 0 | 0 | 0.0235 | 0 | -0.0709 | -0.0342 | 0 | 0 | 0 | 0.0123 | 0 | 0.6591 | |
| L18 | 0.042 | 0.1 | 0 | 0 | 0.066 | 0 | 0 | 0 | 0.0107 | 0.0735 | 0 | 0 | 0.1364 | 0.0554 |

**(P). Malaysia**

| | L1 | L2 | L3 | L4 | L5 | L6 | L7 | L8 | L9 | L10 | L11 | L12 | L13 | L14 |
|---|---|---|---|---|---|---|---|---|---|---|---|---|---|---|
| L1 | | | | | | | | | | | | | | |
| L2 | 0.3218 | | | | | | | | | | | | | |
| L3 | -0.0191 | -0.0295 | | | | | | | | | | | | |
| L4 | -0.1136 | 0 | 0.3439 | | | | | | | | | | | |
| L5 | -0.1012 | 0 | 0.1919 | 0.297 | | | | | | | | | | |
| L6 | 0.0391 | 0.1002 | -0.1022 | 0 | 0.0688 | | | | | | | | | |
| L7 | 0 | 0.0791 | -0.0425 | 0 | 0 | 0.6159 | | | | | | | | |
| L8 | 0.0141 | 0.0551 | 0 | 0.1034 | 0.1247 | 0 | 0.0554 | | | | | | | |
| L9 | 0 | 0.0073 | 0 | -0.0207 | -0.0328 | 0 | 0 | -0.0691 | | | | | | |
| L10 | 0 | 0.0247 | -0.0164 | -0.0038 | -0.0215 | 0 | 0.0172 | -0.0802 | 0.547 | | | | | |
| L11 | 0.0359 | 0 | 0 | 0 | 0.0595 | 0 | 0 | 0.0337 | -0.0814 | -0.0415 | | | | |
| L12 | 0.0025 | 0 | 0 | 0 | 0.0584 | 5.00E-04 | 0.0034 | 0 | -0.0786 | -0.0267 | 0.7831 | | | |
| L13 | -0.001 | -0.0313 | 0 | 0.0513 | 0 | -0.0256 | -0.1189 | 0 | 0 | 0 | 0 | 0 | | |
| L14 | -0.0398 | 0 | 0.0193 | 0.0469 | 0.0293 | 0 | -0.0668 | 0 | 0 | 0 | -0.0166 | 0 | 0.6116 | |
| L18 | 0 | 0.1768 | 0 | 0 | 0 | 0 | 0 | -0.0422 | 0.0422 | 0 | -0.0252 | 0 | 0 | 0 |

**(Q). Mexico**

| | L1 | L2 | L3 | L4 | L5 | L6 | L7 | L8 | L9 | L10 | L11 | L12 | L13 | L14 |
|---|---|---|---|---|---|---|---|---|---|---|---|---|---|---|
| L1 | | | | | | | | | | | | | | |
| L2 | 0.2188 | | | | | | | | | | | | | |
| L3 | -0.0355 | 0 | | | | | | | | | | | | |
| L4 | -0.1053 | 0.0397 | 0.2526 | | | | | | | | | | | |
| L5 | -0.0305 | 0.1056 | 0.0355 | 0.5085 | | | | | | | | | | |
| L6 | 0.019 | 0.0638 | 0 | 0 | 0.0017 | | | | | | | | | |
| L7 | 0 | 0.1254 | 0 | 0 | 0 | 0.647 | | | | | | | | |
| L8 | 0 | 0 | 0.1291 | 0.11 | 0.0895 | 0 | 0.0489 | | | | | | | |
| L9 | 0 | 0.0078 | 0 | 0 | 0 | 0 | 0.0164 | -0.0648 | | | | | | |
| L10 | 0 | 0 | -0.0698 | 0 | 0 | 0 | 0 | 0 | 0.4232 | | | | | |
| L11 | 0 | 0.0375 | 0 | 0 | 0.0658 | 0.0655 | 0.0047 | 0.0236 | -0.0242 | 0 | | | | |
| L12 | 0 | 0.001 | 0 | 0.0473 | 0.0734 | 0.0127 | 0 | 0.0721 | 0 | 0 | 0.5438 | | | |
| L13 | 0 | 0 | 0 | 0.0143 | 0 | -0.0349 | 0 | 0 | 0 | 0 | 0 | 0 | | |
| L14 | -0.0677 | 0 | 0.0512 | 0.0209 | 0.0129 | -0.0522 | -0.0224 | 0 | 0 | 0 | 0.0119 | 0.0211 | 0.6808 | |
| L18 | 0 | 0.1433 | 0 | 0.0142 | 0.0293 | 0 | 0.024 | 0 | 0 | 0 | -0.0076 | 0 | 0.1016 | 0.0474 |

**(R). Nigeria**

| | L1 | L2 | L3 | L4 | L5 | L6 | L7 | L8 | L9 | L10 | L11 | L12 | L13 | L14 |
|---|---|---|---|---|---|---|---|---|---|---|---|---|---|---|
| L1 | | | | | | | | | | | | | | |
| L2 | 0.277 | | | | | | | | | | | | | |
| L3 | -0.1417 | 0 | | | | | | | | | | | | |
| L4 | 0 | 0 | 0.1966 | | | | | | | | | | | |
| L5 | -0.0398 | 0 | 0.1551 | 0.3158 | | | | | | | | | | |
| L6 | 0.072 | 0.0941 | 0 | 0 | 0 | | | | | | | | | |
| L7 | 0 | 0.112 | 0 | 0 | 0 | 0.6014 | | | | | | | | |
| L8 | 0 | 0 | 0.0842 | 0.1122 | 0.1055 | 0.0081 | 0.1011 | | | | | | | |
| L9 | 0 | 0 | -0.0263 | -0.0063 | -0.0834 | 0 | 0 | -0.0621 | | | | | | |
| L10 | 0.0604 | 0.0696 | 0 | 0 | 0 | 0 | 0 | -0.0358 | 0.2643 | | | | | |
| L11 | 0 | 0 | 0.0473 | 0 | 0 | 0.0118 | 0 | 0.0725 | -0.0516 | -0.0958 | | | | |
| L12 | 0 | 0 | 0.0186 | 0 | 0 | 0.0052 | 0.0063 | 0 | -0.1138 | 0 | 0.6608 | | | |
| L13 | 0 | 0 | 0.0127 | 0.0018 | 0.0116 | 0 | -0.0382 | 0 | 0 | 0 | 0.0843 | 0.015 | | |
| L14 | 0 | 0 | 0.054 | 0 | 0.0204 | 0 | -0.0164 | 0 | -0.0288 | 0 | 0 | 0.0096 | 0.4466 | |
| L18 | 0.0283 | 0.0706 | 0 | 0 | 0 | 0 | 0.0436 | 0 | 0 | 0.0026 | 0 | -0.0096 | 0 | 0 |

**(S). Philippines**

| | L1 | L2 | L3 | L4 | L5 | L6 | L7 | L8 | L9 | L10 | L11 | L12 | L13 | L14 |
|---|---|---|---|---|---|---|---|---|---|---|---|---|---|---|
| L1 | | | | | | | | | | | | | | |
| L2 | 0.2679 | | | | | | | | | | | | | |
| L3 | -0.05 | 0 | | | | | | | | | | | | |
| L4 | -0.227 | 0 | 0.3183 | | | | | | | | | | | |
| L5 | -0.0225 | 0 | 0.0812 | 0.2795 | | | | | | | | | | |
| L6 | 0.045 | 0.0653 | -0.0062 | -0.0474 | 0 | | | | | | | | | |
| L7 | 0 | 0.0975 | -0.0202 | 0 | 0.0331 | 0.7395 | | | | | | | | |
| L8 | -0.0718 | 0 | 0.0742 | 0 | 0.246 | 0 | 0 | | | | | | | |
| L9 | 0.0175 | 0.0786 | -0.0205 | -0.0092 | -0.0317 | 0 | 0 | -0.1726 | | | | | | |
| L10 | 0 | 0.0535 | -0.0598 | 0 | -0.0496 | 0 | 0.0189 | 0 | 0.4025 | | | | | |
| L11 | 0 | 0 | 0.0402 | 0 | 0 | 0.0035 | 0 | 0 | -0.0437 | -0.0478 | | | | |
| L12 | -0.0113 | 0.0282 | 0 | 0 | 0.0574 | 0.0195 | 0 | 0.0584 | -0.0329 | 0 | 0.6571 | | | |
| L13 | 0 | 0 | 0 | 0 | 0 | -0.057 | 0 | 0 | 0 | -0.0266 | 0 | 0 | | |
| L14 | 0 | 0 | 0.112 | 0.0738 | 0 | -0.0275 | -0.043 | 0 | 0 | -0.0644 | 0 | 0 | 0.595 | |
| L18 | 0.0248 | 0.1082 | 0 | 0.0418 | 0 | 0 | 0 | -0.0733 | 0 | 0 | 0 | -0.075 | 0 | 0 |

**(T). Republic of Sudan**

| | L1 | L2 | L3 | L4 | L5 | L6 | L7 | L8 | L9 | L10 | L11 | L12 | L13 | L14 |
|---|---|---|---|---|---|---|---|---|---|---|---|---|---|---|
| L1 | | | | | | | | | | | | | | |
| L2 | 0.226 | | | | | | | | | | | | | |
| L3 | -0.0787 | 0 | | | | | | | | | | | | |
| L4 | -0.091 | 0 | 0.1987 | | | | | | | | | | | |
| L5 | 0 | 0 | 0.1685 | 0.2267 | | | | | | | | | | |
| L6 | 0 | 0.0862 | 0 | 0 | 0 | | | | | | | | | |
| L7 | 0.0825 | 0.0992 | 0 | 0 | 0 | 0.6094 | | | | | | | | |
| L8 | 0 | 0 | 0 | 0.0941 | 0.2747 | 0.0139 | 0.1158 | | | | | | | |
| L9 | 0 | 0.0733 | 0 | 0 | 0.0211 | 0 | 0.0578 | 0 | | | | | | |
| L10 | 0 | 0.0885 | -0.0436 | 0 | 0 | 0.0493 | 0 | 0 | 0.2892 | | | | | |
| L11 | 0 | 0 | 0 | 0 | 0.0519 | 0 | 0.0193 | 0.058 | 0 | 0 | | | | |
| L12 | 0 | 0 | 0.032 | 0 | 0 | 0 | 0.0289 | 0.0388 | 0 | -0.1417 | 0.6176 | | | |
| L13 | 0 | 0 | 0.0066 | 0.0322 | 0 | 0 | 0 | 0 | 0 | 0 | 0 | 0 | | |
| L14 | 0 | 0 | 0.0878 | 0 | 0 | -0.0761 | -0.0967 | 0 | -0.0135 | -0.0507 | 0 | 0.0043 | 0.3167 | |
| L18 | 0 | 0.0822 | 0 | 0 | 0 | 0 | 0 | 0 | 0 | 0.0499 | -0.0382 | -0.0889 | 0 | 0 |

**(U). Rwanda**

| | L1 | L2 | L3 | L4 | L5 | L6 | L7 | L8 | L9 | L10 | L11 | L12 | L13 | L14 |
|---|---|---|---|---|---|---|---|---|---|---|---|---|---|---|
| L1 | | | | | | | | | | | | | | |
| L2 | 0.5262 | | | | | | | | | | | | | |
| L3 | 0 | -0.0584 | | | | | | | | | | | | |
| L4 | -0.0494 | -0.065 | 0.1109 | | | | | | | | | | | |
| L5 | -0.1064 | -0.0208 | 0.1915 | 0.3151 | | | | | | | | | | |
| L6 | 0.0251 | 0.0149 | -0.0753 | -0.0038 | 0 | | | | | | | | | |
| L7 | 0.0283 | 0 | 0 | 0 | 0 | 0.4994 | | | | | | | | |
| L8 | 0 | 0 | 0 | 0 | 0 | 0.054 | 0 | | | | | | | |
| L9 | 0 | 0 | 0 | 0 | 0 | 0 | 0 | -0.1051 | | | | | | |
| L10 | 0 | 0 | -0.0088 | -0.1793 | 0 | 0 | 0 | 0 | 0.2851 | | | | | |
| L11 | 0 | 0 | 0 | 0 | 0 | 0.0367 | 0 | 0.171 | -0.1312 | -0.2537 | | | | |
| L12 | 0 | 0 | 0 | 0 | 0.1227 | 0 | 0 | 0.0496 | 0 | 0 | 0.3399 | | | |
| L13 | 0 | 0 | 0 | 0 | 0 | -0.0656 | -0.0168 | 0 | 0 | 0 | 0 | 0 | | |
| L14 | 0 | 0 | 0 | 0 | 0 | 0 | -0.0495 | 0 | -0.0673 | 0 | 0.0348 | 0 | 0.5511 | |
| L18 | 0 | 0.0939 | 0 | 0 | 0 | 0 | 0 | 0 | 0 | 0 | 0 | 0 | 0 | 0 |

**(V). Saudi Arabia**

| | L1 | L2 | L3 | L4 | L5 | L6 | L7 | L8 | L9 | L10 | L11 | L12 | L13 | L14 |
|---|---|---|---|---|---|---|---|---|---|---|---|---|---|---|
| L1 | | | | | | | | | | | | | | |
| L2 | 0.261 | | | | | | | | | | | | | |
| L3 | -0.178 | 0 | | | | | | | | | | | | |
| L4 | -0.1278 | 0 | 0.2586 | | | | | | | | | | | |
| L5 | -0.0473 | 0.0725 | 0.1996 | 0.2845 | | | | | | | | | | |
| L6 | 0.0387 | 0.1003 | -0.0388 | 0 | 0 | | | | | | | | | |
| L7 | 0.0048 | 0.0718 | -0.0185 | 0 | 0.0416 | 0.6745 | | | | | | | | |
| L8 | -0.0345 | 0.0923 | 0.044 | 0.1391 | 0.1349 | 0.015 | 0.0858 | | | | | | | |
| L9 | 0 | 0.0888 | 0 | -0.0098 | -0.0365 | 0.004 | 0.0391 | 0 | | | | | | |
| L10 | 0 | 0.0184 | -0.0019 | -0.0346 | 0 | 0 | 0 | -0.1147 | 0.3446 | | | | | |
| L11 | 0 | 0.0358 | 0 | 0 | 0.1916 | 0 | 0.115 | 0 | -0.0369 | -0.0516 | | | | |
| L12 | 0.0443 | 0 | 0 | 0 | 0 | 0.0176 | 0 | 0 | -0.0681 | 0 | 0.0735 | | | |
| L13 | -0.0366 | 0 | 0 | 0.0398 | 0.0408 | -0.1115 | -0.0076 | 0 | -0.0484 | -0.0609 | 0.0116 | 0 | | |
| L14 | -0.0516 | 0 | 0.1145 | 0 | 0.01 | 0 | -0.0765 | 0.081 | -0.0134 | -0.0552 | 0 | 0 | 0.5792 | |
| L18 | 0 | 0.1894 | -0.0288 | 0 | 0.02 | 0.0185 | 0.0507 | -0.0593 | 0 | 0.1648 | -0.0879 | 0 | 0.1616 | 0 |

**(W). Singapore**

| | L1 | L2 | L3 | L4 | L5 | L6 | L7 | L8 | L9 | L10 | L11 | L12 | L13 | L14 |
|---|---|---|---|---|---|---|---|---|---|---|---|---|---|---|
| L1 | | | | | | | | | | | | | | |
| L2 | 0.1947 | | | | | | | | | | | | | |
| L3 | 0 | 0 | | | | | | | | | | | | |
| L4 | 0 | 0 | 0.2569 | | | | | | | | | | | |
| L5 | 0 | 0 | 0 | 0.2113 | | | | | | | | | | |
| L6 | 0 | 0.0557 | -0.0257 | -0.0388 | 0 | | | | | | | | | |
| L7 | 0 | 0.1445 | 0 | 0 | 0 | 0.539 | | | | | | | | |
| L8 | 0 | 0.0159 | 0.0765 | 0 | 0.1073 | 0 | 0 | | | | | | | |
| L9 | 0 | 0.043 | 0 | -0.0684 | 0 | 0.0447 | 0 | 0 | | | | | | |
| L10 | 0 | 0.1157 | 0 | 0 | 0 | 0.0683 | 0 | 0 | 0.2006 | | | | | |
| L11 | 0 | 0 | 0.0716 | 0.109 | 0.1335 | -0.0312 | 0 | 0 | -0.1335 | 0 | | | | |
| L12 | 0 | 0 | 0 | 0 | 0.1017 | 0 | 0 | 0.0452 | -0.0547 | 0 | 0.4592 | | | |
| L13 | 0 | 0 | 0 | 0.1025 | 0 | -0.1323 | 0 | 0.0406 | 0 | 0 | 0.0319 | 0.005 | | |
| L14 | 0 | 0 | 0.0541 | 0.0162 | 0 | -0.0233 | -0.1352 | 0 | 0 | 0 | 0 | 0 | 0.6095 | |
| L18 | 0 | 0 | 0 | 0 | 0 | 0 | 0 | 0 | 0 | 0 | 0 | 0 | 0 | 0 |

**(X). South Africa**

| | L1 | L2 | L3 | L4 | L5 | L6 | L7 | L8 | L9 | L10 | L11 | L12 | L13 | L14 |
|---|---|---|---|---|---|---|---|---|---|---|---|---|---|---|
| L1 | | | | | | | | | | | | | | |
| L2 | 0 | | | | | | | | | | | | | |
| L3 | 0 | 0 | | | | | | | | | | | | |
| L4 | 0 | 0 | 0 | | | | | | | | | | | |
| L5 | 0 | 0 | 0 | 0.2607 | | | | | | | | | | |
| L6 | 0 | 0 | 0 | 0 | 0 | | | | | | | | | |
| L7 | 0 | 0 | 0 | 0 | 0 | 0.53 | | | | | | | | |
| L8 | 0 | 0.0251 | 0 | 0 | 0.0766 | 0 | 0.0333 | | | | | | | |
| L9 | 0 | 0 | 0 | 0 | 0 | 0 | 0 | 0 | | | | | | |
| L10 | 0 | 0 | 0 | 0 | 0 | 0 | 0 | 0 | 0.2816 | | | | | |
| L11 | 0 | 0 | 0 | 0 | 0 | 0 | 0 | 0 | 0 | -0.0535 | | | | |
| L12 | 0 | 0 | 0 | 0 | 0 | 0 | 0 | 0 | 0 | -0.0328 | 0.3948 | | | |
| L13 | 0 | 0 | 0 | 0.0345 | 0 | 0 | 0 | 0 | -0.0612 | 0 | 0 | 0 | | |
| L14 | 0 | 0 | 0 | 0.0871 | 0 | -0.0576 | 0 | 0 | 0 | -0.0736 | 0.0117 | 0 | 0.3899 | |
| L18 | 0 | 0.0864 | 0 | 0 | 0.0731 | 0 | 0 | 0 | 0 | 0 | 0 | 0 | 0.0146 | 0 |

**(Y). South Korea**

| | L1 | L2 | L3 | L4 | L5 | L6 | L7 | L8 | L9 | L10 | L11 | L12 | L13 | L14 |
|---|---|---|---|---|---|---|---|---|---|---|---|---|---|---|
| L1 | | | | | | | | | | | | | | |
| L2 | 0.409 | | | | | | | | | | | | | |
| L3 | 0 | -0.0385 | | | | | | | | | | | | |
| L4 | 0 | 0 | 0.2653 | | | | | | | | | | | |
| L5 | 0 | 0.0039 | 0.0602 | 0.5239 | | | | | | | | | | |
| L6 | 0 | 0 | -0.0509 | 0 | 0 | | | | | | | | | |
| L7 | 0 | 0.0914 | -0.0379 | 0.0121 | 0.0109 | 0.7106 | | | | | | | | |
| L8 | -0.0123 | 0.0441 | 0.204 | 0.0453 | 0.1571 | -0.0931 | 0 | | | | | | | |
| L9 | 0.0209 | 0.036 | 0 | -0.0037 | -0.0255 | 0 | 0 | -0.0881 | | | | | | |
| L10 | 0.0046 | 0.0594 | -0.0278 | 0 | 0.0121 | 0.0243 | 0.0546 | -0.0213 | 0.4301 | | | | | |
| L11 | 0 | 0.009 | 0 | 0.0073 | 0.0391 | 0 | 0.0546 | 0.0677 | -0.0389 | 0.0383 | | | | |
| L12 | -0.0278 | 0 | 0 | 0.0288 | 0.0638 | 0.0642 | 0 | 0 | -0.0351 | 0 | 0.4551 | | | |
| L13 | 0 | 0.019 | 0.0095 | 0 | 0.0317 | -0.0193 | -0.0018 | 0 | -0.0215 | -0.0424 | 0 | 0.0285 | | |
| L14 | 0.021 | 0 | 0.0703 | 0.0417 | 0 | -0.1154 | -0.054 | 0.0752 | 0 | -0.0211 | -0.0177 | 0 | 0.5159 | |
| L18 | 0.0441 | 0.0928 | 0.0523 | 0.0023 | 0.0259 | -0.0284 | 0 | 0 | 0.0485 | 0 | -0.0127 | 0 | 0.0878 | 0.0945 |

**(Z). Thailand**

| | L1 | L2 | L3 | L4 | L5 | L6 | L7 | L8 | L9 | L10 | L11 | L12 | L13 | L14 |
|---|---|---|---|---|---|---|---|---|---|---|---|---|---|---|
| L1 | | | | | | | | | | | | | | |
| L2 | 0.2981 | | | | | | | | | | | | | |
| L3 | -0.1076 | 0 | | | | | | | | | | | | |
| L4 | -0.0055 | 0 | 0.2172 | | | | | | | | | | | |
| L5 | -0.0484 | 0.0532 | 0.1916 | 0.3515 | | | | | | | | | | |
| L6 | 0.0752 | 0.1011 | 0 | 0 | 0 | | | | | | | | | |
| L7 | 0 | 0.0602 | -0.0431 | 0 | 0.0305 | 0.4342 | | | | | | | | |
| L8 | -0.0247 | 0 | 0.1606 | 0.0398 | 0.1013 | -0.1839 | 0 | | | | | | | |
| L9 | 0 | 0 | -0.0249 | -0.0384 | 0 | -0.0063 | 0 | 0 | | | | | | |
| L10 | 0.0325 | 0.06 | -0.0376 | -0.0061 | 0 | 0 | 0 | -0.0174 | 0.3416 | | | | | |
| L11 | 0.0145 | 0.0017 | 0.0217 | 0 | 0.0287 | 0.0298 | 0 | 0 | -0.1685 | -0.0955 | | | | |
| L12 | 0 | 0 | 0.0207 | 0.0226 | 0.0131 | 0.0117 | 0 | 0.0062 | -0.0544 | -0.0109 | 0.7426 | | | |
| L13 | 0 | 0 | 0.0485 | 0.0435 | 0 | 0 | -0.0195 | 0.054 | 0 | 0 | 0 | 0 | | |
| L14 | -0.0186 | 0 | 0 | 0.0616 | 0.0778 | -0.1211 | 0 | 0.0314 | 0 | 0 | 0 | 0 | 0.5238 | |
| L18 | 0 | 0.0796 | 0 | 0 | 0 | 0 | 0.1304 | 0 | 0.1023 | 0 | -0.0307 | -0.0494 | 0 | 0 |

**(AA). United Kingdom**

| | L1 | L2 | L3 | L4 | L5 | L6 | L7 | L8 | L9 | L10 | L11 | L12 | L13 | L14 |
|---|---|---|---|---|---|---|---|---|---|---|---|---|---|---|
| L1 | | | | | | | | | | | | | | |
| L2 | 0.0653 | | | | | | | | | | | | | |
| L3 | 0 | 0 | | | | | | | | | | | | |
| L4 | 0 | 0.0214 | 0.2303 | | | | | | | | | | | |
| L5 | 0 | 0 | 0.1593 | 0.2383 | | | | | | | | | | |
| L6 | 0 | 0 | 0 | 0 | 0 | | | | | | | | | |
| L7 | 0 | 0.0259 | 0 | 0 | 0 | 0.5808 | | | | | | | | |
| L8 | 0 | 0.1221 | 0 | 0.1563 | 0.1246 | 0 | 0 | | | | | | | |
| L9 | 0 | 0 | 0 | 0 | 0 | 0 | 0 | 0 | | | | | | |
| L10 | 0 | 0 | 0 | 0 | 0 | 0 | 0 | 0 | 0.1801 | | | | | |
| L11 | 0 | 0 | 0 | 0 | 0.0127 | 0 | 0 | 0 | 0 | 0 | | | | |
| L12 | 0 | 0 | 0.0519 | 0 | 0 | 0 | 0 | 0.1683 | -0.024 | 0 | 0.2092 | | | |
| L13 | 0 | 0 | 0 | 0 | 0 | 0 | 0 | 0 | 0 | -0.0205 | 0 | 0 | | |
| L14 | 0 | 0 | 0 | 0.0643 | 0 | -0.0253 | -0.0096 | 0 | 0 | 0 | 0 | 0 | 0.4858 | |
| L18 | 0 | 0.0906 | 0 | 0 | 0 | 0 | 0 | 0 | 0 | 0 | 0 | 0 | 0.006 | 0.0421 |

**(AB). United States**

| | L1 | L2 | L3 | L4 | L5 | L6 | L7 | L8 | L9 | L10 | L11 | L12 | L13 | L14 |
|---|---|---|---|---|---|---|---|---|---|---|---|---|---|---|
| L1 | | | | | | | | | | | | | | |
| L2 | 0.1748 | | | | | | | | | | | | | |
| L3 | 0 | 0.1781 | | | | | | | | | | | | |
| L4 | 0 | 0.0567 | 0.0368 | | | | | | | | | | | |
| L5 | 0 | 0.144 | 0.1453 | 0.1425 | | | | | | | | | | |
| L6 | 0 | 0 | 0 | 0 | 0 | | | | | | | | | |
| L7 | 0 | 0 | 0 | 0 | 0 | 0.6344 | | | | | | | | |
| L8 | 0 | 0 | 0.1095 | 0.1048 | 0.0038 | 0 | 0.1039 | | | | | | | |
| L9 | 0 | 0 | 0 | 0 | 0 | 0 | 0 | 0 | | | | | | |
| L10 | 0 | 0 | 0 | 0 | 0.0513 | 0 | 0 | 0 | 0.4174 | | | | | |
| L11 | 0 | 0.0416 | 0 | 0.0205 | 0.027 | 0 | 0.0254 | 0 | -0.1195 | 0 | | | | |
| L12 | 0 | 0 | 0 | 0.0607 | 0.0357 | 0 | 0 | 0 | -0.0371 | 0 | 0.1944 | | | |
| L13 | 0 | 0 | 0 | 0 | 0 | -0.0665 | -0.021 | 0 | 0 | 0 | 0 | 0 | | |
| L14 | 0 | 0 | 0 | 0 | 0 | 0 | 0 | 0 | 0 | 0 | 0 | 0 | 0.5686 | |
| L18 | 0 | 0.0101 | 0.012 | 0 | 0 | 0 | 0 | 0 | 0 | 0 | 0 | 0 | 0.1327 | 0 |

**(AC). Vietman**

| | L1 | L2 | L3 | L4 | L5 | L6 | L7 | L8 | L9 | L10 | L11 | L12 | L13 | L14 |
|---|---|---|---|---|---|---|---|---|---|---|---|---|---|---|
| L1 | | | | | | | | | | | | | | |
| L2 | 0.4283 | | | | | | | | | | | | | |
| L3 | -0.0607 | 0 | | | | | | | | | | | | |
| L4 | -0.1342 | 0 | 0.3005 | | | | | | | | | | | |
| L5 | -0.003 | 0 | 0.1238 | 0.3832 | | | | | | | | | | |
| L6 | 0 | 0.0722 | 0 | 0 | 0.0265 | | | | | | | | | |
| L7 | 0 | 0.0856 | -0.0047 | 0 | 0 | 0.683 | | | | | | | | |
| L8 | 0 | -0.0272 | 0.0416 | 3.00E-04 | 0 | -0.1901 | -0.2053 | | | | | | | |
| L9 | 0 | 0 | 0 | 0 | 0 | 0 | 0 | -0.1026 | | | | | | |
| L10 | 0 | 0 | -0.06 | 0 | -0.0529 | 0 | 0 | -0.0923 | 0.4975 | | | | | |
| L11 | 0 | 0.0147 | 0 | 0 | 0.1033 | 0.0563 | 0 | 0 | -0.1235 | -0.1013 | | | | |
| L12 | 0 | 0.0354 | 0.0218 | 0 | 0.0071 | 0 | 0.0174 | 0 | -0.0438 | 0 | 0.7589 | | | |
| L13 | 0 | 0.031 | 0.0335 | 0.0226 | 0 | 0 | 0 | -0.0392 | 0 | 0 | 0 | 0.1078 | | |
| L14 | 0 | 0 | 0.0516 | 0.014 | 0.0359 | -0.0355 | -0.0149 | 0 | -0.0025 | -0.029 | 0.0467 | 0 | 0.5298 | |
| L18 | 0.0224 | 0.0826 | 0 | 0 | 0 | 0 | 0 | 0 | 0 | 0 | 0 | 0 | 0 | 0 |

**Table S3.** Partial correlation matrix of variables in health outcomes network across 29 countries.

**(A). Australia**

| | **H1** | **H2** | **H3** | **H4** | **H5** | **H6** | **H7** | **H8** | **H9** | **H10** | **H11** | **H12** |
|---|---|---|---|---|---|---|---|---|---|---|---|---|
| H1 | | | | | | | | | | | | |
| H2 | -0.0436 | | | | | | | | | | | |
| H3 | 0.0361 | -0.1255 | | | | | | | | | | |
| H4 | 0 | 0.0113 | 0.0648 | | | | | | | | | |
| H5 | 0.1027 | -0.1237 | 0.1422 | 0.3405 | | | | | | | | |
| H6 | 0 | 0 | 0.259 | -0.0179 | -0.0682 | | | | | | | |
| H7 | 0.0306 | 0.1246 | 0.0996 | 0.0534 | 0.0502 | 0.0543 | | | | | | |
| H8 | 0.0749 | -0.0986 | 0 | -0.0325 | 0.1463 | 0.1635 | 0 | | | | | |
| H9 | 0.0771 | 0 | 0 | -0.0377 | 0.0307 | 0 | 0 | 0.0866 | | | | |
| H10 | -0.0442 | -0.1234 | 0.1286 | -0.0943 | -0.0233 | 0.1195 | -0.0447 | -0.0354 | 0 | | | |
| H11 | 0 | -0.1288 | 0 | -0.0753 | 0.1152 | 0 | -0.0243 | 0.0067 | 0 | 0 | | |
| H12 | -0.0337 | -0.0043 | -0.0175 | 0 | 0 | -0.0783 | -0.0104 | -0.0674 | 0 | 0 | 0 | |
| H13 | 0 | 0 | -0.0383 | 0 | -0.0636 | -0.0249 | -0.0367 | -0.0233 | 0 | 0 | 0 | 0.6386 |

**(B). Brazil**

| | H1 | H2 | H3 | H4 | H5 | H6 | H7 | H8 | H9 | H10 | H11 | H12 |
|---|---|---|---|---|---|---|---|---|---|---|---|---|
| H1 | | | | | | | | | | | | |
| H2 | -0.3792 | | | | | | | | | | | |
| H3 | 0.1215 | 0 | | | | | | | | | | |
| H4 | 0 | 0 | 0.0786 | | | | | | | | | |
| H5 | 0.0145 | 0 | 0.3474 | 0.3287 | | | | | | | | |
| H6 | 0 | 0 | 0 | 0 | 0 | | | | | | | |
| H7 | 0 | -0.0179 | 0 | 0 | 0.0659 | 0.5106 | | | | | | |
| H8 | 0 | -0.0087 | 0 | 0 | 0 | 0 | 0.1383 | | | | | |
| H9 | 0 | 0 | 0 | 0 | 0 | 0 | 0 | 0 | | | | |
| H10 | 0 | 0 | 0 | 0 | 0.0469 | 0 | 0 | 0.0263 | 0.5214 | | | |
| H11 | 0 | 0 | 0 | 0 | 0.1028 | 0 | 0.0498 | 0 | 0 | 0.0756 | | |
| H12 | 0 | 0 | 0 | 0 | 0 | 0 | 0 | 0 | -0.0179 | -0.0173 | 0 | |
| H13 | 0 | 0 | 0 | 0 | -3.00E-04 | 0 | -0.0849 | 0 | 0 | 0 | 0 | 0 |

**(C). Burundi**

| | H1 | H2 | H3 | H4 | H5 | H6 | H7 | H8 | H9 | H10 | H11 | H12 |
|---|---|---|---|---|---|---|---|---|---|---|---|---|
| H1 | | | | | | | | | | | | |
| H2 | -0.1208 | | | | | | | | | | | |
| H3 | 0 | 0.2263 | | | | | | | | | | |
| H4 | -0.0614 | 0.0036 | 0.2636 | | | | | | | | | |
| H5 | 0 | 0.0885 | 0.1813 | 0.1978 | | | | | | | | |
| H6 | 0.062 | -0.0808 | 0 | -0.0942 | 0 | | | | | | | |
| H7 | 0.0205 | 0 | 0 | -0.0185 | 0 | 0.2837 | | | | | | |
| H8 | 0 | -0.093 | 0 | 0 | 0 | 0.0724 | 0.3411 | | | | | |
| H9 | 0 | 0 | 0.1209 | 0.0837 | 0 | 0 | 0 | -0.1873 | | | | |
| H10 | 0 | 0.0405 | 0 | 0.0699 | 0.018 | -0.0316 | -0.0491 | -0.0745 | 0.2333 | | | |
| H11 | 0 | 0 | 0 | 0.0213 | 0.044 | 0 | 0 | -0.1454 | 0.0887 | 0.2082 | | |
| H12 | 0 | -0.0068 | -0.0096 | 0 | -0.2476 | 0 | 0 | 0 | -0.0775 | -0.1635 | -0.0767 | |
| H13 | -0.0586 | 0.018 | 0 | 0 | 0 | -0.0302 | -0.0572 | -0.0771 | 0 | 0.072 | 0.0407 | -0.1928 |

**(D). Canada**

| | H1 | H2 | H3 | H4 | H5 | H6 | H7 | H8 | H9 | H10 | H11 | H12 |
|---|---|---|---|---|---|---|---|---|---|---|---|---|
| H1 | | | | | | | | | | | | |
| H2 | -0.2717 | | | | | | | | | | | |
| H3 | 0.1398 | 0.0485 | | | | | | | | | | |
| H4 | 0 | 0.0171 | 0.2526 | | | | | | | | | |
| H5 | 0 | 0.0357 | 0.2081 | 0.3119 | | | | | | | | |
| H6 | 0.0362 | 0 | 0 | 0 | 0 | | | | | | | |
| H7 | 0 | 0 | 0 | 0 | 0 | 0.7459 | | | | | | |
| H8 | 0 | 0 | 0.0173 | 0.0702 | 0.0082 | 0.0621 | 0.1887 | | | | | |
| H9 | 0.0412 | 0 | 0 | 0.0042 | 0.0335 | 0 | 0 | 0.0205 | | | | |
| H10 | 0.0327 | 0 | 0 | 0 | 0.0706 | 0.0394 | 0 | 0 | 0.5765 | | | |
| H11 | 0 | 0 | 0.0336 | 0.0171 | 0.2294 | 0 | 0.0216 | 0 | 0.0244 | 0.132 | | |
| H12 | 0 | 0 | 0 | -0.1036 | -0.143 | 0 | 0 | 0 | 0 | 0 | -0.0365 | |
| H13 | 0 | -0.0361 | -0.0095 | 0 | -0.0323 | -0.059 | -0.0692 | -0.0068 | 0 | -0.0278 | 0 | 0.1619 |

**(E). Chile**

| | H1 | H2 | H3 | H4 | H5 | H6 | H7 | H8 | H9 | H10 | H11 | H12 |
|---|---|---|---|---|---|---|---|---|---|---|---|---|
| H1 | | | | | | | | | | | | |
| H2 | -0.4707 | | | | | | | | | | | |
| H3 | 0.0693 | -0.0432 | | | | | | | | | | |
| H4 | 0 | 0 | 0.084 | | | | | | | | | |
| H5 | 0.0493 | 0 | 0.2252 | 0.4017 | | | | | | | | |
| H6 | 0 | 0 | 0 | 0 | 0 | | | | | | | |
| H7 | 0 | 0 | 0 | 0 | 0 | 0.6974 | | | | | | |
| H8 | 0 | -0.0494 | 0 | 0 | 0 | 0 | 0.0778 | | | | | |
| H9 | 0 | 0 | 0 | 0 | 0 | 0 | 0 | 0 | | | | |
| H10 | 0.0248 | -0.0215 | 0 | 0 | 0 | 0 | 0 | -0.0069 | 0.3435 | | | |
| H11 | 0 | 0 | 0.0651 | 0.066 | 0.1646 | 0 | 0 | 0 | 0.039 | 0.0411 | | |
| H12 | 0 | 0 | 0 | 0 | 0 | 0 | 0 | 0 | 0 | -0.1069 | -0.0945 | |
| H13 | 0 | 0.0144 | 0 | 0 | 0 | -0.0363 | 0 | 0 | 0.0098 | 0 | 0.0663 | 0 |

**(F). Egypt**

| | H1 | H2 | H3 | H4 | H5 | H6 | H7 | H8 | H9 | H10 | H11 | H12 |
|---|---|---|---|---|---|---|---|---|---|---|---|---|
| H1 | | | | | | | | | | | | |
| H2 | -0.4025 | | | | | | | | | | | |
| H3 | 0 | 0.1384 | | | | | | | | | | |
| H4 | 0 | 0.06 | 0.2493 | | | | | | | | | |
| H5 | 0 | 0 | 0.1614 | 0.3365 | | | | | | | | |
| H6 | 0.0002 | -0.0444 | 0 | 0 | -0.0213 | | | | | | | |
| H7 | 0 | 0 | 0 | 0 | 0 | 0.3891 | | | | | | |
| H8 | 0 | 0 | 0 | 0 | 0 | 0 | 0.3736 | | | | | |
| H9 | 0 | 0 | 0 | 0 | 0 | -0.1211 | -0.0533 | -0.0032 | | | | |
| H10 | 0 | 0 | 0 | 0.0714 | 0.0184 | 0 | -0.0208 | -0.0097 | 0.1958 | | | |
| H11 | 0 | 0 | 0 | 0 | 0.0698 | 0 | 0 | 0 | 0.0389 | 0.1038 | | |
| H12 | 0 | -0.0084 | -0.0261 | 0 | -0.1452 | 0.0295 | 0 | 0 | -0.0136 | -0.0398 | -0.105 | |
| H13 | 0 | 0 | 0 | 0 | 0 | -0.1259 | -0.0432 | 0 | 0.1026 | 0.0115 | 0 | -0.0956 |

**(G). Guatemala**

| | H1 | H2 | H3 | H4 | H5 | H6 | H7 | H8 | H9 | H10 | H11 | H12 |
|---|---|---|---|---|---|---|---|---|---|---|---|---|
| H1 | | | | | | | | | | | | |
| H2 | -0.3849 | | | | | | | | | | | |
| H3 | 0.0179 | 0 | | | | | | | | | | |
| H4 | 0 | 0.076 | 0.1449 | | | | | | | | | |
| H5 | 0 | 0 | 0.283 | 0.2984 | | | | | | | | |
| H6 | 0 | 0 | 0 | 0 | 0 | | | | | | | |
| H7 | 0 | 0 | 0 | 0 | 0 | 0.4695 | | | | | | |
| H8 | 0 | 0 | 0 | 0 | 0 | 0 | 0.1046 | | | | | |
| H9 | -0.0298 | 0 | 0 | 0 | 0 | 0 | 0 | 0 | | | | |
| H10 | 0 | 0 | 0 | 0 | 0 | 0 | 0 | 0 | 0.2823 | | | |
| H11 | 0 | 0 | 0.0031 | 0.1673 | 0.0466 | 0 | 0 | 0 | 0 | 0.0464 | | |
| H12 | 0 | 0 | -0.1062 | 0 | -0.0618 | 0 | 0 | 0 | -0.055 | -0.087 | -0.057 | |
| H13 | 0 | 0 | 0 | 0 | 0 | -0.0502 | -0.0326 | 0 | 0.1019 | 0 | 0 | 0 |

**(H). Hong Kong**

| | H1 | H2 | H3 | H4 | H5 | H6 | H7 | H8 | H9 | H10 | H11 | H12 |
|---|---|---|---|---|---|---|---|---|---|---|---|---|
| H1 | | | | | | | | | | | | |
| H2 | -0.256 | | | | | | | | | | | |
| H3 | 0 | 0.1985 | | | | | | | | | | |
| H4 | 0 | 0.0647 | 0.2072 | | | | | | | | | |
| H5 | 0 | 0 | 0.1682 | 0.2887 | | | | | | | | |
| H6 | 0.0271 | 0 | 0 | 0 | 0 | | | | | | | |
| H7 | 0.0278 | 0 | 0.0206 | 0.0323 | 0.0226 | 0.5069 | | | | | | |
| H8 | 0.0128 | 0 | 0 | 0 | 0 | 0.0429 | 0.2159 | | | | | |
| H9 | 0 | 0.0064 | 0 | 0 | 0.0287 | 0 | 0 | -0.0559 | | | | |
| H10 | 0 | 0 | 0.0238 | 0 | 0 | -0.0098 | 0 | -0.0375 | 0.369 | | | |
| H11 | -0.0131 | 0 | 0 | 0.0137 | 0.2017 | 0.0816 | 0.0379 | 0 | 0.0239 | 0.0185 | | |
| H12 | 0 | 0 | -0.0835 | -0.0182 | -0.0053 | 0 | -0.0155 | -0.0151 | -0.0183 | -0.019 | -0.2064 | |
| H13 | -0.0093 | 0 | -0.0236 | -0.0424 | 0 | -0.1474 | -0.0555 | -0.0415 | 0.0313 | 0 | -0.0185 | 0.2829 |

**(I). India**

| | H1 | H2 | H3 | H4 | H5 | H6 | H7 | H8 | H9 | H10 | H11 | H12 |
|---|---|---|---|---|---|---|---|---|---|---|---|---|
| H1 | | | | | | | | | | | | |
| H2 | -0.4014 | | | | | | | | | | | |
| H3 | 0 | 0.0598 | | | | | | | | | | |
| H4 | 0 | 0.0426 | 0.2186 | | | | | | | | | |
| H5 | 0 | 0 | 0.2447 | 0.2297 | | | | | | | | |
| H6 | 0.027 | 0 | 0.0037 | 0.0027 | 0 | | | | | | | |
| H7 | 0 | 0 | 0.0059 | 0 | 0.0562 | 0.6342 | | | | | | |
| H8 | 0 | 0 | 0 | 0 | 0.1001 | 0.0552 | 0.1879 | | | | | |
| H9 | 0 | 0 | 0.0353 | 0 | 0.0853 | 0 | 0 | 0 | | | | |
| H10 | 0 | 0 | 0 | 0 | 0 | 0 | 0 | 0 | 0.4429 | | | |
| H11 | 0 | 0 | 0.0423 | 0 | 0.1143 | 0 | 0 | 0 | 0.0978 | 0.2064 | | |
| H12 | 0 | 0 | 0 | 0 | 0 | 0 | 0 | 0 | 0 | 0 | -0.1146 | |
| H13 | 0 | 0 | 0 | 0 | 0 | -0.024 | -0.0644 | -0.093 | 0 | 0 | 0 | 0 |

**(J). Indonesia**

| | H1 | H2 | H3 | H4 | H5 | H6 | H7 | H8 | H9 | H10 | H11 | H12 |
|---|---|---|---|---|---|---|---|---|---|---|---|---|
| H1 | | | | | | | | | | | | |
| H2 | -0.3339 | | | | | | | | | | | |
| H3 | -0.1162 | 0.1598 | | | | | | | | | | |
| H4 | -0.0598 | 0.1789 | 0.064 | | | | | | | | | |
| H5 | -0.0808 | 0.1001 | 0.1697 | 0.2577 | | | | | | | | |
| H6 | 0 | -0.0166 | -0.0659 | -0.013 | -0.1746 | | | | | | | |
| H7 | 0.0688 | 0 | -0.0307 | -0.077 | 0 | 0.6334 | | | | | | |
| H8 | 0.0328 | -0.0356 | -0.0091 | -0.0345 | 0 | 0.1014 | 0.1176 | | | | | |
| H9 | -0.0125 | 0 | 0 | 0 | 0.1134 | -0.0215 | -0.0136 | -0.2172 | | | | |
| H10 | -0.0415 | 0 | 0.0412 | 0 | 0 | -0.0298 | -0.0003 | 0 | 0.5532 | | | |
| H11 | 0 | 0 | 0.0231 | 0 | 0.0251 | 0 | -0.0313 | 0 | 0.1416 | 0.2387 | | |
| H12 | 0 | -0.0087 | 0.0181 | -0.1619 | -0.0413 | 0 | 0 | 0.0281 | -0.0104 | 0 | -0.1015 | |
| H13 | -0.05 | 0 | 0 | 0 | 0 | -0.0256 | -0.0739 | -0.0303 | 0 | 0.0406 | 0.0477 | -0.4937 |

**(K). Italy**

| | H1 | H2 | H3 | H4 | H5 | H6 | H7 | H8 | H9 | H10 | H11 | H12 |
|---|---|---|---|---|---|---|---|---|---|---|---|---|
| H1 | | | | | | | | | | | | |
| H2 | -0.3579 | | | | | | | | | | | |
| H3 | 0 | 0 | | | | | | | | | | |
| H4 | 0 | 0 | 0.0837 | | | | | | | | | |
| H5 | 0 | 0 | 0.127 | 0.4105 | | | | | | | | |
| H6 | 0 | -0.0195 | 0 | 0 | 0 | | | | | | | |
| H7 | 0 | -0.075 | 0.1244 | 0 | 0 | 0.3972 | | | | | | |
| H8 | 0.1114 | -0.0953 | 0 | 0 | 0 | 0 | 0.095 | | | | | |
| H9 | 0 | 0 | 0 | 0 | 0 | 0 | 0 | 0 | | | | |
| H10 | 0 | 0 | 0 | 0 | 0 | 0 | 0 | 0 | 0.3438 | | | |
| H11 | 0 | 0 | 0 | 0.0136 | 0.1827 | 0 | 0.0138 | 0 | 0.0468 | 0.062 | | |
| H12 | 0 | 0 | 0 | 0 | 0 | 0 | 0 | 0 | 0 | 0 | 0 | |
| H13 | 0 | 0 | 0 | 0 | 0 | 0 | 0 | 0 | 0 | 0 | 0 | 0 |

**(L). Lebanon**

| | H1 | H2 | H3 | H4 | H5 | H6 | H7 | H8 | H9 | H10 | H11 | H12 |
|---|---|---|---|---|---|---|---|---|---|---|---|---|
| H1 | | | | | | | | | | | | |
| H2 | -0.5255 | | | | | | | | | | | |
| H3 | 0 | 0.157 | | | | | | | | | | |
| H4 | 0 | 0.0496 | 0.2068 | | | | | | | | | |
| H5 | 0 | 0 | 0.1793 | 0.3619 | | | | | | | | |
| H6 | 0 | -0.0366 | 0 | 0 | 0 | | | | | | | |
| H7 | 0.0129 | -0.0514 | 0 | 0 | 0 | 0.5405 | | | | | | |
| H8 | 0.0253 | -0.0507 | 0.0337 | 0 | 0 | 0.0237 | 0.3709 | | | | | |
| H9 | 0 | 0 | 0.0912 | 0.0051 | 0.0251 | -0.0793 | 0 | -0.0325 | | | | |
| H10 | 0 | 0.0298 | 0 | 0 | 0 | 0 | 0 | -0.1314 | 0.1417 | | | |
| H11 | 0 | 0 | 0.1288 | 0 | 0.177 | 0 | 0.0639 | 0 | 0.1022 | 0.1166 | | |
| H12 | 0 | 0 | -0.0144 | -0.0616 | -0.066 | 0 | -0.0163 | 0 | -0.0798 | -0.073 | -0.1518 | |
| H13 | 0 | 0.0653 | 0 | 0 | 0 | -0.1872 | -0.0635 | -0.0871 | 0.0882 | 0.0523 | 0 | -0.0788 |

**(M). Libya**

| | H1 | H2 | H3 | H4 | H5 | H6 | H7 | H8 | H9 | H10 | H11 | H12 |
|---|---|---|---|---|---|---|---|---|---|---|---|---|
| H1 | | | | | | | | | | | | |
| H2 | -0.5486 | | | | | | | | | | | |
| H3 | -0.0032 | 0.1726 | | | | | | | | | | |
| H4 | 0 | 0.0667 | 0.1144 | | | | | | | | | |
| H5 | 0 | 0.009 | 0.2262 | 0.2665 | | | | | | | | |
| H6 | 0.0378 | 0 | 0 | -0.0335 | -0.061 | | | | | | | |
| H7 | 0 | 0 | 0 | -0.0035 | 0 | 0.4172 | | | | | | |
| H8 | 0 | 0 | 0 | 0 | 0.0524 | 0.0438 | 0.3586 | | | | | |
| H9 | 0 | 0.0252 | 0.0484 | 0 | 0 | -0.0748 | -0.0118 | 0 | | | | |
| H10 | 0 | 0 | 0 | 0.0053 | 0 | 0 | 0 | 0 | 0.2987 | | | |
| H11 | 0 | 0 | 0 | 0.0091 | 0.0984 | 0 | 0 | 0 | 0.1151 | 0.2076 | | |
| H12 | 0 | -0.0441 | -0.0248 | 0 | -0.1703 | 0 | 0 | 0 | 0 | -0.0488 | -0.1862 | |
| H13 | 0 | 0.0223 | 0 | 0 | 0 | -0.1005 | -0.0059 | -0.1101 | 0 | 0.0374 | 0 | -0.1269 |

**(N). Macau**

| | H1 | H2 | H3 | H4 | H5 | H6 | H7 | H8 | H9 | H10 | H11 | H12 |
|---|---|---|---|---|---|---|---|---|---|---|---|---|
| H1 | | | | | | | | | | | | |
| H2 | -0.3495 | | | | | | | | | | | |
| H3 | 0 | 0.0553 | | | | | | | | | | |
| H4 | 0 | 0.0875 | 0.0438 | | | | | | | | | |
| H5 | 0 | 0 | 0.2186 | 0.3483 | | | | | | | | |
| H6 | 0 | 0 | 0 | 0 | 0 | | | | | | | |
| H7 | 0 | 0 | 0 | 0 | 0 | 0.5954 | | | | | | |
| H8 | 0 | 0 | 0 | 0 | 0 | 0 | 0.2226 | | | | | |
| H9 | 0 | 0 | 0 | 0 | 0 | 0 | 0 | -5.00E-04 | | | | |
| H10 | 0 | 0 | 0 | 0 | 0 | 0 | 0 | 0 | 0.2748 | | | |
| H11 | 0 | 0 | 0 | 0 | 0.155 | 0 | 0 | 0 | 0 | 0 | | |
| H12 | 0 | 0 | 0 | 0 | -0.0198 | 0 | 0 | 0 | 0 | 0 | 0 | |
| H13 | 0 | 0 | 0 | 0 | 0 | 0 | 0 | -0.1073 | 0 | 0 | 0 | 0.158 |

**(O). Mainland China**

| | H1 | H2 | H3 | H4 | H5 | H6 | H7 | H8 | H9 | H10 | H11 | H12 |
|---|---|---|---|---|---|---|---|---|---|---|---|---|
| H1 | | | | | | | | | | | | |
| H2 | -0.4213 | | | | | | | | | | | |
| H3 | 0.0265 | 0.2483 | | | | | | | | | | |
| H4 | 0 | 0.0193 | 0.1762 | | | | | | | | | |
| H5 | 0 | 0.0943 | 0.2428 | 0.4651 | | | | | | | | |
| H6 | 0 | -0.0067 | 0 | 0.0022 | 0.0312 | | | | | | | |
| H7 | 0.0429 | 0 | 0 | 0 | 0.029 | 0.6699 | | | | | | |
| H8 | 0.0211 | 0 | 0.0622 | 0 | 0 | 0.0695 | 0.1418 | | | | | |
| H9 | -0.0367 | 0 | 0 | 0.0104 | 0.0564 | -0.0368 | 0 | -0.0931 | | | | |
| H10 | -0.0465 | 0 | 0 | 0 | 0 | 0 | -0.0108 | -0.0459 | 0.3776 | | | |
| H11 | 0 | 0 | 0.0471 | 0 | 0.0891 | 0 | 0.0624 | 0 | 0 | 0 | | |
| H12 | 0 | 0 | -0.0451 | 0 | -0.0394 | 0 | 0 | 0 | -0.0544 | -0.0213 | -0.3 | |
| H13 | -0.0383 | 0 | 0 | 0 | 0 | -0.1697 | -0.0742 | -0.1435 | 0 | 0 | 0 | 0.2011 |

**(P). Malaysia**

| | H1 | H2 | H3 | H4 | H5 | H6 | H7 | H8 | H9 | H10 | H11 | H12 |
|---|---|---|---|---|---|---|---|---|---|---|---|---|
| H1 | | | | | | | | | | | | |
| H2 | -0.4726 | | | | | | | | | | | |
| H3 | -0.0138 | 0.1855 | | | | | | | | | | |
| H4 | 0 | 0.0886 | 0.1557 | | | | | | | | | |
| H5 | -0.0164 | 0 | 0.1485 | 0.4233 | | | | | | | | |
| H6 | 0.0592 | 0 | -0.0312 | 0 | 0 | | | | | | | |
| H7 | 0 | 0 | 0 | 0 | 0 | 0.6574 | | | | | | |
| H8 | 0.0329 | -0.0312 | -0.0189 | 0 | 0.0507 | 0.0245 | 0.197 | | | | | |
| H9 | -0.0207 | 0.0141 | 0.0609 | 0 | 0.0877 | 0 | -0.0208 | 0 | | | | |
| H10 | 0 | 0.0073 | 0.0599 | 0 | 0.0208 | 0 | 0 | -0.0331 | 0.6003 | | | |
| H11 | -0.0052 | 0 | 0.0781 | 0 | 0.0979 | -0.0023 | 0 | 0 | 0.1296 | 0.1872 | | |
| H12 | 0 | -0.0241 | 0 | -0.1104 | -0.0147 | 0.0156 | 0 | 0.0116 | 0 | 0 | -0.1586 | |
| H13 | 0 | 0.0521 | 0 | 0 | 0.0413 | -0.1733 | 0 | -0.1342 | 0 | 0.0591 | 0.0589 | -0.1673 |

**(Q). Mexico**

| | H1 | H2 | H3 | H4 | H5 | H6 | H7 | H8 | H9 | H10 | H11 | H12 |
|---|---|---|---|---|---|---|---|---|---|---|---|---|
| H1 | | | | | | | | | | | | |
| H2 | -0.4555 | | | | | | | | | | | |
| H3 | 0.0942 | 0 | | | | | | | | | | |
| H4 | -0.0345 | 0.0377 | 0.0476 | | | | | | | | | |
| H5 | 0 | 0 | 0.3254 | 0.3758 | | | | | | | | |
| H6 | 0.0411 | 0 | 0 | 0 | -0.0094 | | | | | | | |
| H7 | 0 | -0.0394 | 0 | 0.0702 | 0.0069 | 0.5944 | | | | | | |
| H8 | 0.0679 | -0.1131 | -0.0391 | 0 | 0.0898 | 0 | 0.1938 | | | | | |
| H9 | 0 | 0 | 0.0393 | 0 | 0.0643 | -0.0484 | -0.0498 | 0 | | | | |
| H10 | 0 | 0.0164 | 0 | 0 | 0.0694 | 0 | 0 | 0.0353 | 0.3492 | | | |
| H11 | -0.0316 | 0 | 0.0338 | 0.1258 | 0.1251 | 0 | 0.0373 | -0.03 | 0 | 0.1397 | | |
| H12 | 0 | 0 | 0 | 0 | -0.1392 | 0 | 0 | 0 | -0.0164 | -0.117 | -0.1464 | |
| H13 | -0.0262 | 0.0041 | 0 | 0 | -0.032 | -0.0497 | -0.0582 | -0.0899 | 0.0336 | 0 | 0 | 0 |

**(R). Nigeria**

| | H1 | H2 | H3 | H4 | H5 | H6 | H7 | H8 | H9 | H10 | H11 | H12 |
|---|---|---|---|---|---|---|---|---|---|---|---|---|
| H1 | | | | | | | | | | | | |
| H2 | -0.2444 | | | | | | | | | | | |
| H3 | 0 | 0.2066 | | | | | | | | | | |
| H4 | -0.0423 | 0.2089 | 0.0619 | | | | | | | | | |
| H5 | -0.0921 | 0 | 0.1696 | 0.2614 | | | | | | | | |
| H6 | 0.0822 | 0 | 0 | 0 | 0.0503 | | | | | | | |
| H7 | 0 | 0 | 0 | 0 | 0.0458 | 0.5116 | | | | | | |
| H8 | 0.0172 | 0 | 0 | 0 | 0.0386 | 0.1469 | 0.254 | | | | | |
| H9 | -0.0282 | 0.025 | 9.00E-04 | 0 | 0.0348 | 0 | 0 | -0.0741 | | | | |
| H10 | 0 | 0 | 0.0232 | 0.0351 | 0.0573 | 0 | 0 | 0 | 0.4346 | | | |
| H11 | 0 | 0.0084 | 0 | 0 | 0.017 | 0 | 0 | 0 | 0.1433 | 0.1479 | | |
| H12 | 0.0039 | 0 | 0 | 0 | -0.1031 | 0 | 0 | 0 | -0.0745 | -0.1721 | -0.1399 | |
| H13 | 0 | 0.0393 | 0 | 0 | 0 | -0.1181 | -0.0285 | -0.0631 | 0.0606 | 0 | 0 | 0 |

**(S). Philippines**

| | H1 | H2 | H3 | H4 | H5 | H6 | H7 | H8 | H9 | H10 | H11 | H12 |
|---|---|---|---|---|---|---|---|---|---|---|---|---|
| H1 | | | | | | | | | | | | |
| H2 | -0.4298 | | | | | | | | | | | |
| H3 | -0.0572 | 0.2644 | | | | | | | | | | |
| H4 | -0.0653 | 0 | 0.1164 | | | | | | | | | |
| H5 | 0 | 0 | 0.2493 | 0.3336 | | | | | | | | |
| H6 | 0.0395 | -0.0282 | -0.0167 | -0.0198 | 0 | | | | | | | |
| H7 | 0 | 0 | 0 | 0 | 0 | 0.7379 | | | | | | |
| H8 | 0.0548 | -0.0555 | 0 | 0 | 0.0158 | 0.0688 | 0.1677 | | | | | |
| H9 | 0 | 0 | 0.0049 | 0.0042 | 0.1143 | 0 | 0 | -0.1355 | | | | |
| H10 | 0 | 0.1217 | 0 | 0.0309 | 0.0506 | 0 | 0 | 0 | 0.5678 | | | |
| H11 | -0.0243 | 0 | 0.0562 | 0.0059 | 0.0187 | 0 | 0.0482 | -0.0553 | 0.0394 | 0.2348 | | |
| H12 | 0.0629 | -0.0069 | 0 | 0 | -0.1818 | 0 | 0 | 0 | 0 | -0.0401 | -0.2228 | |
| H13 | 0 | 0 | 0 | 0 | 0 | -0.0872 | -0.0791 | -0.0866 | 0 | 0.0396 | 0.0167 | -0.2622 |

**(T). Republic of Sudan**

| | H1 | H2 | H3 | H4 | H5 | H6 | H7 | H8 | H9 | H10 | H11 | H12 |
|---|---|---|---|---|---|---|---|---|---|---|---|---|
| H1 | | | | | | | | | | | | |
| H2 | -0.3759 | | | | | | | | | | | |
| H3 | -0.0996 | 0.2141 | | | | | | | | | | |
| H4 | -0.0495 | 0.1047 | 0.244 | | | | | | | | | |
| H5 | 0 | 0 | 0.168 | 0.2067 | | | | | | | | |
| H6 | 0 | -0.0604 | 0 | -0.0091 | 0 | | | | | | | |
| H7 | 0 | 0 | 0 | 0 | 0 | 0.4146 | | | | | | |
| H8 | 0 | 0 | 0 | 0 | 0.0164 | 0.0688 | 0.2399 | | | | | |
| H9 | 0 | 0 | 0.0491 | 0 | 0 | -0.0567 | 0 | 0 | | | | |
| H10 | 0 | 0 | 0 | 0 | 0 | 0 | 0 | 0 | 0.1911 | | | |
| H11 | 0 | 0 | 0.0078 | 0 | 0.1254 | 0 | 0.042 | 0 | 0.1253 | 0.1287 | | |
| H12 | 0.0132 | 0 | -0.0222 | 0 | -0.1851 | 0 | 0 | 0 | -0.0431 | 0 | -0.0794 | |
| H13 | -0.025 | 0 | 0 | 0 | 0 | -0.1324 | -0.0037 | -0.0502 | 0.0752 | 0.0121 | 0 | -0.1106 |

**(U). Rwanda**

| | H1 | H2 | H3 | H4 | H5 | H6 | H7 | H8 | H9 | H10 | H11 | H12 |
|---|---|---|---|---|---|---|---|---|---|---|---|---|
| H1 | | | | | | | | | | | | |
| H2 | -0.1757 | | | | | | | | | | | |
| H3 | 0 | 0 | | | | | | | | | | |
| H4 | 0 | 0 | 0.028 | | | | | | | | | |
| H5 | 0 | 0 | 0.1471 | 0.1292 | | | | | | | | |
| H6 | 0 | 0 | 0 | 0 | 0 | | | | | | | |
| H7 | 0 | 0 | 0 | 0 | 0 | 0.3847 | | | | | | |
| H8 | 0 | 0 | 0 | 0 | 0 | 0 | 0 | | | | | |
| H9 | 0 | 0 | 0 | 0 | 0 | 0 | 0 | 0 | | | | |
| H10 | 0 | 0 | 0 | 0 | 0 | 0 | 0 | 0 | 0 | | | |
| H11 | 0 | 0 | 0 | 0 | 0 | 0 | 0 | 0 | 0 | 0 | | |
| H12 | 0 | 0 | 0 | 0 | -0.0906 | 0 | 0 | 0 | 0 | 0 | -0.1506 | |
| H13 | 0 | 0 | 0 | 0 | 0 | 0 | 0 | 0 | 0 | 0 | 0 | 0 |

**(V). Saudi Arabia**

| | H1 | H2 | H3 | H4 | H5 | H6 | H7 | H8 | H9 | H10 | H11 | H12 |
|---|---|---|---|---|---|---|---|---|---|---|---|---|
| H1 | | | | | | | | | | | | |
| H2 | -0.5025 | | | | | | | | | | | |
| H3 | 0 | 0.1156 | | | | | | | | | | |
| H4 | 0 | 0.1202 | 0.1655 | | | | | | | | | |
| H5 | 0 | 0 | 0.2274 | 0.3951 | | | | | | | | |
| H6 | 0 | 0 | 0 | 0 | -0.0471 | | | | | | | |
| H7 | 0 | 0 | 0 | -0.0109 | 0 | 0.5099 | | | | | | |
| H8 | 0.0112 | -0.0259 | 0 | 0 | 0 | 0.0599 | 0.319 | | | | | |
| H9 | 0 | 0.0106 | 0.098 | 0.046 | 0.0289 | -0.0084 | -0.0452 | 0 | | | | |
| H10 | 0 | 0 | 0 | 0.0239 | 0 | -0.0105 | 0 | -0.0347 | 0.2703 | | | |
| H11 | 0 | 0 | 0.0437 | 0.0317 | 0.1141 | 0 | 0 | 0 | 0.1897 | 0.2199 | | |
| H12 | 0 | 0 | -0.0496 | -0.0263 | -0.0947 | 0.0315 | 0 | 0 | 0 | -0.0824 | -0.1295 | |
| H13 | -0.0205 | 0.0582 | 0 | 0 | -0.0309 | -0.0495 | -0.0433 | -0.0517 | 0.0373 | 0.0453 | 0 | -0.1669 |

**(W). Singapore**

| | H1 | H2 | H3 | H4 | H5 | H6 | H7 | H8 | H9 | H10 | H11 | H12 |
|---|---|---|---|---|---|---|---|---|---|---|---|---|
| H1 | | | | | | | | | | | | |
| H2 | -0.273 | | | | | | | | | | | |
| H3 | 0 | 0.1793 | | | | | | | | | | |
| H4 | 0 | 0 | 0.1173 | | | | | | | | | |
| H5 | 0 | 0.006 | 0.1349 | 0.333 | | | | | | | | |
| H6 | 0 | 0 | 0 | 0 | 0.0445 | | | | | | | |
| H7 | 0.0457 | 0 | 0 | 0 | 0.0791 | 0.6974 | | | | | | |
| H8 | 0 | 0 | 0 | 0 | 0 | 0.0744 | 0.1019 | | | | | |
| H9 | 0 | 0.008 | 0 | 0 | 0 | 0 | 0 | 0 | | | | |
| H10 | 0 | 0 | 0.0689 | 0.0955 | 0 | 0 | 0 | 0 | 0.4526 | | | |
| H11 | 0 | 0.0353 | 0.0788 | 0 | 0.1415 | 0 | 0 | 0.0945 | 0 | 0 | | |
| H12 | 0 | 0 | 0 | 0 | 0 | 0 | 0 | 0 | 0 | 0 | -0.0261 | |
| H13 | -0.0377 | 0 | 0 | 0 | 0 | 0 | -0.0787 | 0 | 0 | 0 | 0 | 0.3008 |

**(X). South Africa**

| | H1 | H2 | H3 | H4 | H5 | H6 | H7 | H8 | H9 | H10 | H11 | H12 |
|---|---|---|---|---|---|---|---|---|---|---|---|---|
| H1 | | | | | | | | | | | | |
| H2 | -0.4102 | | | | | | | | | | | |
| H3 | 0 | 0 | | | | | | | | | | |
| H4 | 0 | 0 | 0.1241 | | | | | | | | | |
| H5 | 0 | 0 | 0.2096 | 0.2315 | | | | | | | | |
| H6 | 0 | 0 | 0 | 0 | 0 | | | | | | | |
| H7 | 0 | 0 | 0 | 0 | 0 | 0.5787 | | | | | | |
| H8 | 0 | -0.0444 | 0 | 0 | 0 | 0.0963 | 0.2676 | | | | | |
| H9 | 0 | 0 | 0 | 0 | 0 | 0 | 0 | 0 | | | | |
| H10 | 0 | 0 | 0.0261 | 0 | 0.0172 | 0 | 0 | 0 | 0.43 | | | |
| H11 | 0 | 0 | 0 | 0.0889 | 0.1879 | 0 | 0 | 0 | 0 | 0.0655 | | |
| H12 | 0 | 0 | 0 | 0 | 0 | 0 | 0 | 0 | 0 | -0.0163 | -0.1229 | |
| H13 | 0 | 0 | 0 | 0 | 0 | 0 | -0.2091 | -0.026 | 0 | 0 | 0 | -0.0177 |

**(Y). South Korea**

| | H1 | H2 | H3 | H4 | H5 | H6 | H7 | H8 | H9 | H10 | H11 | H12 |
|---|---|---|---|---|---|---|---|---|---|---|---|---|
| H1 | | | | | | | | | | | | |
| H2 | -0.3676 | | | | | | | | | | | |
| H3 | 0.0421 | 0 | | | | | | | | | | |
| H4 | 0.011 | 0.0237 | 0.3687 | | | | | | | | | |
| H5 | 0.0236 | 0 | 0.141 | 0.241 | | | | | | | | |
| H6 | 0 | 0 | 0 | -0.026 | -0.1418 | | | | | | | |
| H7 | 0 | 0 | -0.0047 | 0 | -0.0042 | 0.775 | | | | | | |
| H8 | 0 | 0 | 0 | 0 | 0 | 0 | 0.0807 | | | | | |
| H9 | 0 | 0 | 0.024 | 0.0025 | 0 | 0 | -0.0175 | -0.0889 | | | | |
| H10 | 0 | 0 | 0 | 9.00E-04 | 0 | -3.00E-04 | -0.0071 | -0.0129 | 0.5938 | | | |
| H11 | 0 | 0 | 0.0682 | 0.0438 | 0.2149 | 0 | 0 | 0 | 0.0718 | 0 | | |
| H12 | 0 | 0 | -0.02 | -0.0356 | -0.109 | 0 | 0 | 0 | -0.0255 | -0.0042 | -0.1053 | |
| H13 | -0.0065 | 0 | 0 | 0 | 0 | -0.1217 | -0.0484 | -0.049 | 0.0186 | 0 | 0 | -0.2589 |

**(Z). Thailand**

| | H1 | H2 | H3 | H4 | H5 | H6 | H7 | H8 | H9 | H10 | H11 | H12 |
|---|---|---|---|---|---|---|---|---|---|---|---|---|
| H1 | | | | | | | | | | | | |
| H2 | -0.4107 | | | | | | | | | | | |
| H3 | -0.2561 | 0.1579 | | | | | | | | | | |
| H4 | 0 | 0.0993 | 0.286 | | | | | | | | | |
| H5 | 0 | 0 | 0.1534 | 0.2048 | | | | | | | | |
| H6 | 0 | 0 | 0 | -0.0609 | -0.2545 | | | | | | | |
| H7 | 0 | 0 | -0.0342 | 0 | -0.0654 | 0.5335 | | | | | | |
| H8 | 0 | -0.0251 | 0 | 0 | 0.0239 | 0 | 0.1521 | | | | | |
| H9 | 0 | 0 | 0.0236 | 0.0578 | 0.0738 | 0 | -0.0081 | -0.0434 | | | | |
| H10 | -0.0092 | 0 | 0.0086 | 0.0176 | 0.0037 | 0 | 0 | -0.2087 | 0.3076 | | | |
| H11 | 0 | 0 | 0 | 0 | 0.1333 | 0 | -0.0305 | -0.1553 | 0 | 0.0391 | | |
| H12 | 0 | 0 | 0 | 0 | -0.1032 | 0.0268 | 0 | 0 | -0.0414 | -0.1122 | -0.1937 | |
| H13 | -0.0365 | 0 | 0.0059 | 0.0056 | 0.0116 | -0.1158 | -0.0599 | 0 | 0.0172 | 0 | 0.0362 | -0.4184 |

**(AA). United Kingdom**

| | H1 | H2 | H3 | H4 | H5 | H6 | H7 | H8 | H9 | H10 | H11 | H12 |
|---|---|---|---|---|---|---|---|---|---|---|---|---|
| H1 | | | | | | | | | | | | |
| H2 | -0.2275 | | | | | | | | | | | |
| H3 | 0 | 0 | | | | | | | | | | |
| H4 | 0 | 0 | 0.2684 | | | | | | | | | |
| H5 | 0 | 0 | 0.1609 | 0.1264 | | | | | | | | |
| H6 | 0 | 0 | 0 | 0 | 0 | | | | | | | |
| H7 | 0 | 0 | 0 | 0 | 0 | 0.5964 | | | | | | |
| H8 | 0 | 0 | 0 | 0 | 0 | 0 | 0.0973 | | | | | |
| H9 | 0 | 0 | 0 | 0 | 0 | 0 | 0 | 0 | | | | |
| H10 | 0 | 0 | 0 | 0 | 0 | 0 | 0 | 0 | 0.3233 | | | |
| H11 | 0 | 0 | 0 | 0 | 0.2415 | 0 | 0 | 0 | 0.0279 | 0.1343 | | |
| H12 | 0 | 0 | 0 | 0 | 0 | 0 | 0 | 0 | 0 | -0.0761 | 0 | |
| H13 | 0 | 0 | 0 | 0 | 0 | -0.0604 | 0 | 0 | 0 | 0 | 0 | 0 |

**(AB). United States**

| | H1 | H2 | H3 | H4 | H5 | H6 | H7 | H8 | H9 | H10 | H11 | H12 |
|---|---|---|---|---|---|---|---|---|---|---|---|---|
| H1 | | | | | | | | | | | | |
| H2 | -0.4345 | | | | | | | | | | | |
| H3 | 0.1063 | 0 | | | | | | | | | | |
| H4 | 0 | 0 | 0.1174 | | | | | | | | | |
| H5 | 0.0509 | 0 | 0.1392 | 0.3581 | | | | | | | | |
| H6 | 0 | 0 | 0 | 0 | 0.0559 | | | | | | | |
| H7 | 0 | 0 | 0 | 0 | 0 | 0.7185 | | | | | | |
| H8 | 0.0748 | 0 | 0 | 0 | 0 | 0.0308 | 0.0795 | | | | | |
| H9 | -0.0173 | 0.0522 | 0 | 0 | 0 | 0 | 0 | 0 | | | | |
| H10 | 0 | 0.0447 | 0 | 0 | 0.0821 | 0.0205 | 0 | 0 | 0.4219 | | | |
| H11 | 0 | 0 | 0.0241 | 0 | 0.2408 | 0 | 0 | 0 | 0 | 0.1096 | | |
| H12 | 0 | 0 | 0 | 0 | -0.0366 | 0 | -0.0301 | -0.0249 | 0 | -0.0312 | 0 | |
| H13 | 0 | 0 | 0 | 0 | 0 | 0 | -0.0724 | -0.103 | 0 | 0 | 0 | 0 |

**(AC). Vietnam**

| | H1 | H2 | H3 | H4 | H5 | H6 | H7 | H8 | H9 | H10 | H11 | H12 |
|---|---|---|---|---|---|---|---|---|---|---|---|---|
| H1 | | | | | | | | | | | | |
| H2 | -0.0938 | | | | | | | | | | | |
| H3 | -0.2398 | 0.3061 | | | | | | | | | | |
| H4 | 0 | 0.1745 | 0.1784 | | | | | | | | | |
| H5 | 0 | 0.2126 | 0.2295 | 0.2006 | | | | | | | | |
| H6 | 0.01 | 0 | 0 | 0 | 0 | | | | | | | |
| H7 | 0.0787 | 0 | 0 | 0.0553 | 0.0105 | 0.5566 | | | | | | |
| H8 | 0 | 0.0752 | 0.0344 | 0 | 0.0748 | 0.0052 | 0.3779 | | | | | |
| H9 | 0 | 0 | 0 | 0 | 0 | 0 | 0 | -0.1288 | | | | |
| H10 | 0 | 0 | 0 | 0 | 0 | 0 | -0.025 | -0.0915 | 0.7261 | | | |
| H11 | 0.0202 | 0.0462 | 0.0807 | 0 | 0.0584 | 0.0546 | 0 | 0.0832 | 0.0386 | 0.1913 | | |
| H12 | 0 | -0.019 | 0 | 0 | -0.1885 | 0 | 0 | 0 | -0.0514 | -0.0192 | -0.2649 | |
| H13 | 0 | 0 | 0 | 0 | 0 | -0.0874 | -0.1413 | -0.0525 | 0 | 0 | 0 | -0.0809 |

**Table S4.** Partial correlation matrix of variables in bridge network of lifestyles and health outcomes across 29 countries.

## (A). Australia

| | L1 | L2 | L3 | L4 | L5 | L6 | L7 | L8 | L9 | L10 | L11 | L12 | L13 | L14 | L18 | H1 | H2 | H3 | H4 | H5 | H6 | H7 | H8 | H9 | H10 | H11 | H12 |
|---|---|---|---|---|---|---|---|---|---|---|---|---|---|---|---|---|---|---|---|---|---|---|---|---|---|---|---|
| L1 | | | | | | | | | | | | | | | | | | | | | | | | | | | |
| L2 | 0 | | | | | | | | | | | | | | | | | | | | | | | | | | |
| L3 | 0.147 | -0.0798 | | | | | | | | | | | | | | | | | | | | | | | | | |
| L4 | 0 | 0 | 0.0592 | | | | | | | | | | | | | | | | | | | | | | | | |
| L5 | -0.0971 | 0.0768 | 0 | -0.0617 | | | | | | | | | | | | | | | | | | | | | | | |
| L6 | 0 | 0 | 0 | 0.0202 | 0 | | | | | | | | | | | | | | | | | | | | | | |
| L7 | -0.0492 | -0.021 | -0.0069 | -0.0685 | 0 | 0.0681 | | | | | | | | | | | | | | | | | | | | | |
| L8 | 0 | 0.1256 | -0.0703 | 0.0476 | -0.0351 | 0.184 | 0 | | | | | | | | | | | | | | | | | | | | |
| L9 | 0.1884 | 0.3153 | 0 | 0.0478 | 0.125 | 0 | -0.0057 | 0 | | | | | | | | | | | | | | | | | | | |
| L10 | -0.1011 | 0 | -0.0118 | -0.0128 | 0.0329 | -0.1785 | 0.0432 | 0 | 0.1565 | | | | | | | | | | | | | | | | | | |
| L11 | 0.053 | 0 | 0 | 0 | 0 | 0 | 0 | 0 | 0.1426 | 0 | | | | | | | | | | | | | | | | | |
| L12 | 0 | 0.0334 | 0 | 0 | 0 | 0 | 0 | 0 | 0.0311 | 0 | 0.1247 | | | | | | | | | | | | | | | | |
| L13 | 0 | 0.1374 | 0 | 0.0349 | 0 | 0.0256 | -0.0525 | 0 | 0.081 | 0.0588 | 0.0329 | 0.0167 | | | | | | | | | | | | | | | |
| L14 | 0.185 | 0.0186 | 0 | 0 | 0.1173 | -0.0864 | 0 | 0.0027 | 0 | -0.0348 | 0 | 0 | 0.0177 | | | | | | | | | | | | | | |
| L18 | 0 | 0 | 0 | 0 | 0 | 0 | 0 | -0.0053 | -0.0068 | 0 | 0 | 0 | 0 | 0 | | | | | | | | | | | | | |
| H1 | 0 | 0.0555 | 0 | 0 | 0 | 0 | 0 | 0 | 0.0227 | 0 | 0 | 0 | 0.0645 | 0.0769 | 0 | | | | | | | | | | | | |
| H2 | 0 | 0 | 0 | -0.0361 | 0 | 0 | 0 | 0 | 0 | 0 | 0 | 0 | 0 | 0 | 0 | -0.0065 | | | | | | | | | | | |
| H3 | 0 | 0.0563 | 0 | 0 | 0 | 0 | 0 | 0 | 0.083 | 0 | 0.1039 | 0 | 0.0669 | 0.057 | 0 | 0 | -0.0931 | | | | | | | | | | |
| H4 | 0 | 0 | 0 | 0 | 0 | 0 | 0 | 0 | 0 | 0 | 0 | 0 | 0 | 0 | 0 | 0 | 0 | 0.0223 | | | | | | | | | |
| H5 | 0 | 0 | 0 | 0 | 0 | 0 | 0 | 0 | 0 | 0 | 0 | 0 | 0.0543 | 0.0709 | 0 | 0.0732 | -0.0959 | 0.0972 | 0.2959 | | | | | | | | |
| H6 | 0 | 0 | 0 | 0 | 0 | 0 | 0 | 0 | 0.0637 | 0 | 0 | 0.0463 | 0.0758 | 0.0481 | 0 | 0 | 0 | 0.1875 | 0 | 0 | | | | | | | |
| H7 | 0 | 0.0511 | 0 | 0 | 0 | 0 | 0 | 0 | 0.0502 | 0 | 0 | 0 | 0.065 | 0.0123 | 0 | 0 | 0.0642 | 0.0358 | 0.033 | 0.0075 | 0 | | | | | | |
| H8 | 0.0175 | 0.0466 | 0 | 0 | 0 | 0 | 0 | 0 | 0.2141 | 0 | 0.0582 | 0 | 0.0301 | 0.0454 | 0 | 0.0198 | -0.0633 | 0 | 0 | 0.0861 | 0.0649 | 0 | | | | | |
| H9 | 0 | 0.0182 | 0 | 0 | 0 | 0 | 0 | 0 | 0.0214 | 0 | 0 | 0 | 0.0239 | 0.0292 | 0 | 0.0286 | 0 | 0 | 0 | 0 | 0 | 0 | 0.0308 | | | | |
| H10 | 0 | 0 | 0 | 0 | 0 | 0 | 0 | 0 | 0 | 0 | 0 | 0 | 0 | 0 | 0 | 0 | -0.0833 | 0.0817 | -0.0555 | 0 | 0.0847 | 0 | 0 | 0 | | | |
| H11 | 0 | 0 | 0 | 0 | 0 | 0 | -0.0028 | 0 | 0 | 0 | 0 | 0 | 0 | 0.0243 | 0 | 0 | -0.0981 | 0 | -0.0044 | 0.0545 | 0 | 0 | 0 | 0 | 0 | | |
| H12 | 0 | -0.0171 | 0 | 0 | 0 | 0 | 0 | 0 | -0.0028 | 0 | 0 | 0 | -0.0382 | -0.0405 | 0 | 0 | 0 | 0 | 0 | 0 | -0.061 | 0 | -0.0322 | 0 | 0 | 0 | |
| H13 | 0 | -0.0011 | 0 | 0 | 0 | 0 | 0 | 0 | -0.0919 | 0 | -0.0084 | 0 | 0 | -0.0051 | 0.0052 | 0 | 0 | -0.0128 | 0 | -0.0381 | -0.0023 | -0.0049 | 0 | 0 | 0 | 0 | 0.5911 |

## (B). Brazil

| | L1 | L2 | L3 | L4 | L5 | L6 | L7 | L8 | L9 | L10 | L11 | L12 | L13 | L14 | L18 | H1 | H2 | H3 | H4 | H5 | H6 | H7 | H8 | H9 | H10 | H11 | H12 |
|---|---|---|---|---|---|---|---|---|---|---|---|---|---|---|---|---|---|---|---|---|---|---|---|---|---|---|---|
| L1 | | | | | | | | | | | | | | | | | | | | | | | | | | | |
| L2 | 0 | | | | | | | | | | | | | | | | | | | | | | | | | | |
| L3 | 0 | 0.0569 | | | | | | | | | | | | | | | | | | | | | | | | | |
| L4 | 0 | 0.1395 | 0.2160 | | | | | | | | | | | | | | | | | | | | | | | | |
| L5 | 0 | 0.0147 | 0.1219 | 0.3603 | | | | | | | | | | | | | | | | | | | | | | | |
| L6 | 0 | 0 | 0 | 0 | 0 | | | | | | | | | | | | | | | | | | | | | | |
| L7 | 0 | 0.0102 | 0.0080 | 0 | 0.0180 | 0.5446 | | | | | | | | | | | | | | | | | | | | | |
| L8 | 0 | 0 | 0.0894 | 0.1134 | 0.1161 | 0 | 0 | | | | | | | | | | | | | | | | | | | | |
| L9 | 0 | 0 | 0 | 0 | 0 | 0 | 0 | 0 | | | | | | | | | | | | | | | | | | | |
| L10 | 0 | 0 | 0 | 0 | 0 | 0 | 0 | 0 | 0.2424 | | | | | | | | | | | | | | | | | | |
| L11 | 0 | 0 | 0.0434 | 0.0167 | 0.0083 | 0 | 0 | 0 | 0 | 0 | | | | | | | | | | | | | | | | | |
| L12 | 0 | 0 | 0 | 0 | 0.0329 | 0 | 0 | 0.0160 | 0 | 0 | 0.2585 | | | | | | | | | | | | | | | | |
| L13 | 0 | 0 | 0 | 0 | 0 | -0.0377 | 0 | 0 | 0 | 0 | 0 | 0 | | | | | | | | | | | | | | | |
| L14 | 0 | 0 | 0 | 0 | 0 | -0.0409 | 0 | 0 | -0.0090 | 0 | 0 | 0 | 0.5326 | | | | | | | | | | | | | | |
| L18 | 0 | 0.0511 | 0 | 0 | 0.0055 | 0 | 0 | 0 | 0 | 0 | 0 | 0 | 0 | 0 | | | | | | | | | | | | | |
| H1 | -0.0362 | 0 | 0 | 0.0757 | 0.0324 | 0 | 0 | 0 | 0 | 0 | 0 | 0 | 0 | 0 | 0.1110 | | | | | | | | | | | | |
| H2 | 0.1756 | 0 | 0 | 0 | 0 | 0 | 0 | 0 | 0 | 0 | 0 | 0 | 0 | 0 | 0 | -0.3335 | | | | | | | | | | | |
| H3 | 0 | 0.0003 | 0 | 0.0052 | 0.0108 | 0 | 0 | 0 | 0 | 0 | 0 | 0 | 0.0298 | 0 | 0.3402 | 0.0544 | 0 | | | | | | | | | | |
| H4 | 0 | 0 | 0 | 0 | 0 | 0 | 0 | 0 | 0 | 0 | 0 | 0 | 0.0333 | 0 | 0 | 0 | 0 | 0.0626 | | | | | | | | | |
| H5 | 0 | 0.0213 | 0 | 0 | 0.0279 | 0 | 0 | 0.0053 | 0 | 0 | 0 | 0 | 0 | 0 | 0.0293 | 0 | 0 | 0.2988 | 0.3091 | | | | | | | | |
| H6 | 0 | 0 | 0 | 0 | 0 | 0 | 0 | 0 | 0 | 0 | 0 | 0 | 0 | 0 | 0 | 0 | 0 | 0 | 0 | 0 | | | | | | | |
| H7 | 0 | 0 | 0 | 0 | 0 | 0 | 0 | 0 | 0 | 0 | 0 | 0 | 0 | 0.0039 | 0 | 0 | 0 | 0 | 0 | 0.0494 | 0.4838 | | | | | | |
| H8 | 0 | 0 | 0 | 0 | 0 | 0 | 0 | 0 | 0 | 0 | 0 | 0 | 0 | 0.0568 | 0 | 0 | 0 | 0 | 0 | 0 | 0 | 0.1145 | | | | | |
| H9 | 0 | 0 | 0 | 0 | 0 | 0 | 0 | 0 | 0 | 0 | 0 | 0 | 0 | 0 | 0 | 0 | 0 | 0 | 0 | 0 | 0 | 0 | 0 | | | | |
| H10 | 0 | 0 | 0 | 0 | 0 | 0 | 0 | 0 | 0 | 0 | 0 | 0 | 0 | 0 | 0 | 0 | 0 | 0 | 0 | 0.0297 | 0 | 0 | 0.0014 | 0.4923 | | | |
| H11 | 0 | 0 | 0 | 0 | 0 | 0 | 0 | 0 | 0 | 0 | 0 | 0 | 0 | 0 | 0 | 0 | 0 | 0 | 0 | 0.0839 | 0 | 0.0295 | 0 | 0 | 0.0546 | | |
| H12 | 0 | 0 | 0 | 0 | 0 | 0 | 0 | 0 | 0 | 0 | 0 | 0 | 0 | 0 | 0 | 0 | 0 | 0 | 0 | 0 | 0 | 0 | 0 | -0.0004 | 0 | 0 | |
| H13 | 0 | 0 | 0 | 0 | 0 | 0 | 0 | 0 | 0.0408 | 0 | 0 | 0 | 0 | 0 | 0 | 0 | 0 | 0 | 0 | 0 | 0 | -0.0602 | 0 | 0 | 0 | 0 | 0 |

## (C). Burundi

| | L1 | L2 | L3 | L4 | L5 | L6 | L7 | L8 | L9 | L10 | L11 | L12 | L13 | L14 | L18 | H1 | H2 | H3 | H4 | H5 | H6 | H7 | H8 | H9 | H10 | H11 | H12 |
|---|---|---|---|---|---|---|---|---|---|---|---|---|---|---|---|---|---|---|---|---|---|---|---|---|---|---|---|
| L1 | | | | | | | | | | | | | | | | | | | | | | | | | | | |
| L2 | 0.3763 | | | | | | | | | | | | | | | | | | | | | | | | | | |
| L3 | 0 | -0.0581 | | | | | | | | | | | | | | | | | | | | | | | | | |
| L4 | 0 | 0 | 0.2 | | | | | | | | | | | | | | | | | | | | | | | | |
| L5 | 0 | -0.0386 | 0 | 0.1412 | | | | | | | | | | | | | | | | | | | | | | | |
| L6 | 0 | 0 | 0 | -0.075 | -0.0055 | | | | | | | | | | | | | | | | | | | | | | |
| L7 | 0 | 0 | 0 | -0.0633 | 0 | 0.2364 | | | | | | | | | | | | | | | | | | | | | |
| L8 | -0.0016 | 0 | 0 | 0 | 0 | -0.0428 | -0.0198 | | | | | | | | | | | | | | | | | | | | |
| L9 | 0 | 0 | 0 | -0.0838 | 0 | 0.0678 | 0.0187 | -0.1848 | | | | | | | | | | | | | | | | | | | |
| L10 | 0 | 0 | 0 | -0.0121 | -0.0726 | 0.0785 | 0.111 | -0.0414 | 0.0838 | | | | | | | | | | | | | | | | | | |
| L11 | -0.033 | 0 | 0 | 0 | 0 | 0 | 0 | 0 | 0 | 0 | | | | | | | | | | | | | | | | | |
| L12 | 0 | 0 | 0 | 0 | 0 | 0 | 0 | 0 | 0 | 0 | 0.1222 | | | | | | | | | | | | | | | | |
| L13 | 0 | 0 | 0 | 0 | 0 | -0.0467 | -0.0905 | 0.0643 | 0 | -0.0511 | 0 | 0 | | | | | | | | | | | | | | | |
| L14 | 0 | 0 | 0 | 0.0495 | 0 | 0 | -0.1183 | 0.0292 | -0.0254 | -0.0986 | 0 | 0 | 0.0831 | | | | | | | | | | | | | | |
| L18 | 0 | 0 | 0 | 0 | 0 | 0 | 0 | 0 | 0 | 0 | 0 | 0 | 0 | 0 | | | | | | | | | | | | | |
| H1 | 0 | 0 | 0 | 0 | 0 | 0 | 0 | 0 | 0 | 0 | 0 | 0 | 0.0028 | 0 | -0.0106 | | | | | | | | | | | | |
| H2 | 0 | 0 | 0 | 0 | 0 | 0 | 0 | 0 | 0.007 | 0 | 0 | 0 | 0 | -0.0077 | 0.0189 | -0.0351 | | | | | | | | | | | |
| H3 | 0.0098 | 0 | 0 | 0 | 0 | 0 | 0 | 0 | 0 | 0 | 0 | 0 | -0.0212 | 0 | 0.1135 | 0 | 0.166 | | | | | | | | | | |
| H4 | 0 | 0 | 0 | 0 | 0 | 0 | 0 | 0 | 0 | 0 | 0 | 0 | 0 | -0.0436 | 0.1194 | 0 | 0 | 0.1984 | | | | | | | | | |
| H5 | 0 | 0 | 0 | 0 | 0 | 0 | 0 | 0 | 0 | 0 | 0 | 0 | 0 | 0 | 0 | 0 | 0.05 | 0.1472 | 0.1549 | | | | | | | | |
| H6 | 0 | 0 | 0 | 0 | 0 | 0 | 0 | 0 | 0 | 0 | 0 | 0 | 0.0435 | 0.0125 | 0 | 0 | -0.0312 | 0 | -0.0443 | 0 | | | | | | | |
| H7 | 0 | 0 | 0 | 0 | 0 | 0 | 0 | 0 | 0 | 0 | 0 | 0 | 0 | 0 | 0 | 0 | 0 | 0 | 0 | 0 | 0.2208 | | | | | | |
| H8 | 0 | 0 | 0 | 0 | 0 | 0 | 0 | 0 | 0 | 0 | 0 | 0 | 0 | 0.0698 | 0 | 0 | -0.0542 | 0 | 0 | 0 | 0.0566 | 0.2846 | | | | | |
| H9 | 0 | 0 | 0 | 0 | 0 | 0 | 0 | 0 | 0 | 0 | 0 | 0 | 0 | 0 | 0.0314 | 0 | 0 | 0.0794 | 0.0568 | 0 | 0 | 0 | -0.1508 | | | | |
| H10 | 0 | 0 | 0 | 0 | 0 | 0 | 0 | 0 | 0 | 0 | 0 | 0 | 0 | 0 | 0 | 0 | 0.0114 | 0 | 0.0524 | 0.0119 | -0.0041 | -0.0286 | -0.0793 | 0.2008 | | | |
| H11 | 0 | 0 | 0 | 0 | 0 | 0 | 0 | 0 | 0 | 0 | 0 | 0 | 0 | 0 | 0 | 0 | 0 | 0 | 0 | 0.0091 | 0 | 0 | -0.1071 | 0.0656 | 0.1725 | | |
| H12 | 0 | 0 | 0 | 0 | 0 | 0 | 0 | 0 | 0 | 0 | 0 | 0 | 0 | 0 | 0 | 0 | 0 | 0 | 0 | -0.19 | 0 | 0 | 0 | -0.0517 | -0.1385 | -0.0514 | |
| H13 | 0 | 0 | 0 | 0 | 0 | 0 | 0 | 0 | 0 | 0 | 0 | 0 | 0 | 0 | 0 | 0 | 0 | 0 | 0 | 0 | 0 | -0.0173 | -0.0513 | 0 | 0.0462 | 0.0001 | -0.1279 |

## (D). Canada

| | L1 | L2 | L3 | L4 | L5 | L6 | L7 | L8 | L9 | L10 | L11 | L12 | L13 | L14 | L18 | H1 | H2 | H3 | H4 | H5 | H6 | H7 | H8 | H9 | H10 | H11 | H12 |
|---|---|---|---|---|---|---|---|---|---|---|---|---|---|---|---|---|---|---|---|---|---|---|---|---|---|---|---|
| L1 | | | | | | | | | | | | | | | | | | | | | | | | | | | |
| L2 | 0.3888 | | | | | | | | | | | | | | | | | | | | | | | | | | |
| L3 | 0 | 0.0665 | | | | | | | | | | | | | | | | | | | | | | | | | |
| L4 | 0 | 0 | 0.2096 | | | | | | | | | | | | | | | | | | | | | | | | |
| L5 | 0 | 0 | 0 | 0.2047 | | | | | | | | | | | | | | | | | | | | | | | |
| L6 | 0 | 0 | 0 | 0 | 0 | | | | | | | | | | | | | | | | | | | | | | |
| L7 | 0 | 0 | 0 | 0 | 0 | 0.5786 | | | | | | | | | | | | | | | | | | | | | |
| L8 | 0 | 0 | 0 | 0.0247 | 0 | 0 | 0.1289 | | | | | | | | | | | | | | | | | | | | |
| L9 | 0 | 0.0132 | 0 | 0 | 0 | 0 | 0 | 0 | | | | | | | | | | | | | | | | | | | |
| L10 | 0 | 0.0316 | 0 | 0 | 0.0241 | 0 | 0 | 0 | 0.2192 | | | | | | | | | | | | | | | | | | |
| L11 | 0 | 0 | 0 | 0 | 0 | 0 | 0 | 0 | 0 | 0 | | | | | | | | | | | | | | | | | |
| L12 | 0 | 0 | 0 | 0.0226 | 0.0193 | 0 | 0 | 0 | 0 | 0 | 0.155 | | | | | | | | | | | | | | | | |
| L13 | 0 | 0 | 0 | 0 | 0 | 0 | 0 | 0 | 0 | 0 | 0 | 0.0095 | | | | | | | | | | | | | | | |
| L14 | 0 | 0 | 0 | 0.0829 | 0 | -0.0492 | 0 | 0 | 0 | 0 | 0 | 0 | 0.622 | | | | | | | | | | | | | | |
| L18 | 0 | 0.0608 | 0 | 0 | 0 | 0 | 0 | 0 | 0 | 0 | 0 | 0 | 0.1116 | 0.1095 | | | | | | | | | | | | | |
| H1 | -0.0097 | 0 | 0 | 0.0392 | 0 | 0 | 0 | 0 | 0 | 0 | 0 | 0.0059 | 0.0714 | 0 | 0.095 | | | | | | | | | | | | |
| H2 | 0.126 | 0 | 0 | -0.1159 | 0 | 0 | 0 | 0 | 0 | 0 | 0 | 0 | 0 | 0 | 0 | -0.163 | | | | | | | | | | | |
| H3 | 0 | 0 | 0 | 0 | 0 | 0 | 0 | 0 | 0 | 0 | 0 | 0 | 0.049 | 0.0475 | 0.2453 | 0.0076 | 0 | | | | | | | | | | |
| H4 | 0 | 0 | 0 | 0 | 0 | 0 | 0 | 0 | 0 | 0 | 0 | 0 | 0 | 0 | 0 | 0 | 0 | 0.2042 | | | | | | | | | |
| H5 | 0 | 0 | 0 | 0 | 0 | 0 | 0 | 0 | 0 | 0 | 0 | 0 | 0.0228 | 0 | 0 | 0 | 0 | 0.1698 | 0.2854 | | | | | | | | |
| H6 | 0 | 0 | 0 | 0 | 0 | 0 | 0 | 0 | 0 | 0 | 0 | 0 | 0.0141 | 0.0155 | 0 | 0 | 0 | 0 | 0 | 0 | | | | | | | |
| H7 | 0 | 0 | 0 | 0 | 0 | -0.0065 | 0 | 0 | 0 | 0 | 0 | 0 | 0 | 0 | 0 | 0 | 0 | 0 | 0 | 0 | 0.6715 | | | | | | |
| H8 | 0 | 0 | 0 | 0 | 0 | 0 | 0 | 0 | 0 | 0 | 0 | 0 | 0 | 0 | 0 | 0 | 0 | 0 | 0.0211 | 0 | 0.0629 | 0.1642 | | | | | |
| H9 | 0 | 0 | 0 | 0 | 0 | 0 | 0 | 0 | 0 | 0 | 0 | 0 | 0 | 0 | 0 | 0 | 0 | 0 | 0 | 0.016 | 0 | 0 | 0 | | | | |
| H10 | 0 | 0 | 0 | 0 | 0 | -0.0267 | 0 | 0 | 0 | 0 | 0 | 0 | 0.0147 | 0.0148 | 0 | 0 | 0 | 0 | 0 | 0.0543 | 0.0125 | 0 | 0 | 0.5077 | | | |
| H11 | 0 | 0 | 0 | 0 | 0 | -0.0128 | 0 | 0 | 0 | 0 | 0 | 0 | 0 | 0.0369 | 0 | 0 | 0 | 0 | 0.0003 | 0.2032 | 0 | 0 | 0 | 0.0064 | 0.1029 | | |
| H12 | 0 | 0 | 0 | 0 | 0 | 0 | 0 | 0 | 0.007 | 0 | 0 | 0 | 0 | 0 | 0 | 0 | 0 | 0 | -0.0661 | -0.1181 | 0 | 0 | 0 | 0 | 0 | 0 | |
| H13 | 0 | 0 | 0 | 0 | 0 | 0 | 0 | 0 | 0 | 0 | 0 | 0 | 0 | -0.0054 | 0 | 0 | 0 | 0 | 0 | 0 | -0.041 | -0.0503 | 0 | 0 | 0 | 0 | 0.0978 |

## (E). Chile

| | L1 | L2 | L3 | L4 | L5 | L6 | L7 | L8 | L9 | L10 | L11 | L12 | L13 | L14 | L18 | H1 | H2 | H3 | H4 | H5 | H6 | H7 | H8 | H9 | H10 | H11 | H12 |
|---|---|---|---|---|---|---|---|---|---|---|---|---|---|---|---|---|---|---|---|---|---|---|---|---|---|---|---|
| L1 | | | | | | | | | | | | | | | | | | | | | | | | | | | |
| L2 | 0 | | | | | | | | | | | | | | | | | | | | | | | | | | |
| L3 | 0 | 0 | | | | | | | | | | | | | | | | | | | | | | | | | |
| L4 | 0 | 0.0924 | 0.0925 | | | | | | | | | | | | | | | | | | | | | | | | |
| L5 | 0 | 0 | 0 | 0.271 | | | | | | | | | | | | | | | | | | | | | | | |
| L6 | 0 | 0 | 0 | 0 | 0 | | | | | | | | | | | | | | | | | | | | | | |
| L7 | 0 | 0 | 0 | 0 | 0 | 0.3689 | | | | | | | | | | | | | | | | | | | | | |
| L8 | 0 | 0 | 0 | 0.0771 | 0 | 0 | 0 | | | | | | | | | | | | | | | | | | | | |
| L9 | 0 | 0 | 0 | 0 | 0 | 0 | 0 | 0 | | | | | | | | | | | | | | | | | | | |
| L10 | 0 | 0 | 0 | 0 | 0 | 0 | 0 | 0 | 0.2376 | | | | | | | | | | | | | | | | | | |
| L11 | 0 | 0 | 0 | 0.0127 | 0.02 | 0 | 0 | 0 | 0 | -0.0075 | | | | | | | | | | | | | | | | | |
| L12 | 0 | 0 | 0 | 0.0757 | 0 | 0 | 0 | 0 | 0 | 0 | 0.2358 | | | | | | | | | | | | | | | | |
| L13 | 0 | 0 | 0 | 0 | 0 | 0 | 0 | 0 | 0 | 0 | 0 | 0 | | | | | | | | | | | | | | | |
| L14 | 0 | 0 | 0 | 0 | 0 | -0.0464 | 0 | 0 | 0 | 0 | 0 | 0 | 0.561 | | | | | | | | | | | | | | |
| L18 | 0 | 0 | 0 | 0 | 0 | 0 | 0 | 0 | 0 | 0 | 0 | 0 | 0.0594 | 0 | | | | | | | | | | | | | |
| H1 | 0 | 0 | 0 | 0.0939 | 0 | 0 | 0 | 0 | 0 | 0 | 0 | 0 | 0 | 0 | 0.0454 | | | | | | | | | | | | |
| H2 | 0.0582 | 0 | 0 | -0.0735 | 0 | 0.0028 | 0 | 0 | 0 | 0 | 0 | 0 | -0.0072 | 0 | 0 | -0.3494 | | | | | | | | | | | |
| H3 | 0 | 0 | 0 | 0 | 0 | 0 | 0 | 0 | 0 | 0 | 0 | 0 | 0 | 0 | 0.2519 | 0 | 0 | | | | | | | | | | |
| H4 | 0 | 0 | 0 | 0 | 0 | 0 | 0 | 0 | 0 | 0 | 0 | 0 | 0 | 0 | 0 | 0 | 0 | 0.0361 | | | | | | | | | |
| H5 | 0 | 0 | 0 | 0 | 0 | 0 | 0 | 0 | 0 | 0 | 0 | 0 | 0 | 0 | 0.0237 | 0 | 0 | 0.1601 | 0.3243 | | | | | | | | |
| H6 | 0 | 0 | 0 | 0 | 0 | 0 | 0 | 0 | 0 | 0 | 0 | 0 | 0 | 0 | 0 | 0 | 0 | 0 | 0 | 0 | | | | | | | |
| H7 | 0 | 0 | 0 | 0 | 0 | 0 | 0 | 0 | 0 | 0 | 0 | 0 | 0 | 0 | 0 | 0 | 0 | 0 | 0 | 0 | 0.5814 | | | | | | |
| H8 | 0 | 0 | 0 | 0 | 0 | 0 | 0 | 0 | 0 | 0 | 0 | 0 | 0 | 0 | 0 | 0 | 0 | 0 | 0 | 0 | 0 | 0 | | | | | |
| H9 | 0 | 0 | 0 | 0 | 0 | 0 | 0 | 0 | 0 | 0 | 0 | 0 | 0 | 0 | 0 | 0 | 0 | 0 | 0 | 0 | 0 | 0 | 0 | | | | |
| H10 | 0 | 0 | 0 | 0 | 0 | 0 | 0 | 0 | 0 | 0 | 0 | 0 | 0 | 0 | 0 | 0 | 0 | 0 | 0 | 0 | 0 | 0 | 0 | 0.2303 | | | |
| H11 | 0 | 0 | 0 | 0 | 0 | 0 | 0 | 0 | 0 | 0 | 0 | 0 | 0 | 0 | 0.0314 | 0 | 0 | 0 | 0.014 | 0.1097 | 0 | 0 | 0 | 0 | 0 | | |
| H12 | 0 | 0 | 0 | 0 | 0 | 0 | 0 | 0 | 0 | 0 | 0 | 0 | 0 | 0 | 0 | 0 | 0 | 0 | 0 | 0 | 0 | 0 | 0 | 0 | -0.0022 | 0 | |
| H13 | 0 | 0 | 0 | 0 | 0 | 0 | 0 | 0 | 0 | 0 | 0 | 0 | 0 | 0 | 0 | 0 | 0 | 0 | 0 | 0 | 0 | 0 | 0 | 0 | 0 | 0 | 0 |

## (F). Egypt

| | L1 | L2 | L3 | L4 | L5 | L6 | L7 | L8 | L9 | L10 | L11 | L12 | L13 | L14 | L18 | H1 | H2 | H3 | H4 | H5 | H6 | H7 | H8 | H9 | H10 | H11 | H12 |
|---|---|---|---|---|---|---|---|---|---|---|---|---|---|---|---|---|---|---|---|---|---|---|---|---|---|---|---|
| L1 | | | | | | | | | | | | | | | | | | | | | | | | | | | |
| L2 | 0.1688 | | | | | | | | | | | | | | | | | | | | | | | | | | |
| L3 | 0 | 0 | | | | | | | | | | | | | | | | | | | | | | | | | |
| L4 | -0.0245 | 0 | 0.1983 | | | | | | | | | | | | | | | | | | | | | | | | |
| L5 | 0 | 0 | 0.2107 | 0.1472 | | | | | | | | | | | | | | | | | | | | | | | |
| L6 | 0.1034 | 0.0615 | 0.0166 | 0 | 0 | | | | | | | | | | | | | | | | | | | | | | |
| L7 | 0 | 0.0972 | 0 | 0 | 0 | 0.6309 | | | | | | | | | | | | | | | | | | | | | |
| L8 | 0 | 0 | 0.105 | 0.1717 | 0.2117 | 0.0373 | 0.1073 | | | | | | | | | | | | | | | | | | | | |
| L9 | 0.0406 | 0.0064 | 0 | -0.0056 | -0.0297 | 0 | 0 | 0 | | | | | | | | | | | | | | | | | | | |
| L10 | 0 | 0.0807 | 0 | 0 | 0 | 0 | 0 | 0 | 0.1862 | | | | | | | | | | | | | | | | | | |
| L11 | 0 | 0 | 0.0037 | 0 | 0.0702 | 0 | 0 | 0.0177 | 0 | 0 | | | | | | | | | | | | | | | | | |
| L12 | 0 | 0 | 0.0491 | 0 | 0.0156 | 0 | 0 | 0 | -0.0583 | -0.1229 | 0.6219 | | | | | | | | | | | | | | | | |
| L13 | -0.0134 | 0 | 0 | 0 | 0 | -0.0213 | -4.00E-04 | 0 | -0.0234 | -0.0156 | 0.0165 | 0 | | | | | | | | | | | | | | | |
| L14 | -0.0591 | 0 | 0.0317 | 0 | 0.0212 | 0 | 0 | 0 | -0.0197 | -0.0292 | 0.0307 | 0 | 0.4388 | | | | | | | | | | | | | | |
| L18 | 0 | 0.0229 | -0.098 | -0.0378 | 0 | 0 | 0 | -0.0311 | 0.0749 | 0.0548 | 0 | -0.1293 | 0 | 0 | | | | | | | | | | | | | |
| H1 | -0.0294 | 0 | 0 | 0 | 0 | -0.0208 | -0.0116 | 0 | -0.0111 | 0 | 0 | 0 | 0.0538 | 0.0392 | 0 | | | | | | | | | | | | |
| H2 | 0.1428 | 0 | -0.0052 | 0 | 0 | 0 | 0 | -0.0611 | 0 | 0 | 0 | 0 | -0.0189 | -0.0482 | 0 | -0.3736 | | | | | | | | | | | |
| H3 | 0.0601 | 0 | 0 | 0 | 0 | 0 | 0 | 0 | 0 | 0 | 0 | 0 | 0 | 0 | 0.0532 | 0 | 0.1207 | | | | | | | | | | |
| H4 | 0.0176 | 0.0055 | 0 | 0 | 0 | 0 | 0 | 0 | 0 | 0 | 0 | 0 | 0 | 0 | 0 | 0 | 0.0514 | 0.2439 | | | | | | | | | |
| H5 | 0 | 0 | 0 | 0 | 0 | 0 | 0.0059 | 0 | 0 | 0 | 0 | 0 | 0 | 0 | 0.0077 | 0 | 0 | 0.1578 | 0.3331 | | | | | | | | |
| H6 | -0.011 | 0 | 0 | 0 | 0 | -0.0379 | 0 | 0 | 0 | 0 | 0 | 0 | 0.0041 | 0.0156 | 0 | 0 | -0.0258 | 0 | 0 | -0.0166 | | | | | | | |
| H7 | 0 | 0 | 0 | 0 | 0 | 0 | 0 | 0 | 0 | -0.023 | 0.0026 | 0 | 0 | 0.0347 | 0 | 0 | 0 | 0 | 0 | 0 | 0.3827 | | | | | | |
| H8 | 0 | 0 | 0.0614 | 0 | 0.0369 | 0 | 0 | 0 | 0 | 0 | 0 | 0.0016 | 0.0427 | 0.0204 | 0 | 0 | 0 | 0 | 0 | 0 | 0 | 0.3628 | | | | | |
| H9 | 0.021 | 0 | 0 | 0 | 0 | 0.0594 | 0.0473 | 0 | 0 | 0 | 0 | 0 | -0.0361 | 0 | 0 | 0 | 0 | 0 | 0 | 0 | -0.1086 | -0.0506 | 0 | | | | |
| H10 | 0.0297 | 0.09 | 0 | 0 | 0 | 0 | 0.0037 | 0 | 0 | 0.0615 | 0 | 0 | -0.0547 | 0 | 0.0495 | 0 | 0 | 0 | 0.0608 | 0.014 | 0 | -0.0136 | 0 | 0.178 | | | |
| H11 | 0 | 0 | 0 | 0 | 0 | 0 | 0 | 0 | 0 | 0 | 0 | 0 | 0 | 0 | 0.0489 | 0 | 0 | 0 | 0 | 0.0647 | 0 | 0 | 0 | 0.0341 | 0.0951 | | |
| H12 | -0.0155 | -0.0218 | 0 | 0 | 0 | 0 | 0 | 0.029 | 0 | 0 | 0 | 0 | 0 | 0 | -0.01 | 0 | 0 | -0.0194 | 0 | -0.1423 | 0.0263 | 0 | 0 | -0.0098 | -0.0324 | -0.0997 | |
| H13 | 0.0011 | 0 | 0 | 0 | 0 | 0 | 0 | 0 | 0 | 0.0816 | 0 | 0 | 0 | -0.0183 | 0 | 0 | 0 | 0 | 0 | 0 | -0.122 | -0.0369 | 0 | 0.097 | 0 | 0 | -0.09 |

## (G). Guatemala

| | L1 | L2 | L3 | L4 | L5 | L6 | L7 | L8 | L9 | L10 | L11 | L12 | L13 | L14 | L18 | H1 | H2 | H3 | H4 | H5 | H6 | H7 | H8 | H9 | H10 | H11 | H12 |
|---|---|---|---|---|---|---|---|---|---|---|---|---|---|---|---|---|---|---|---|---|---|---|---|---|---|---|---|
| L1 | | | | | | | | | | | | | | | | | | | | | | | | | | | |
| L2 | 0 | | | | | | | | | | | | | | | | | | | | | | | | | | |
| L3 | 0 | 0 | | | | | | | | | | | | | | | | | | | | | | | | | |
| L4 | 0 | 0 | 0.1474 | | | | | | | | | | | | | | | | | | | | | | | | |
| L5 | 0 | 0 | 0 | 0.3557 | | | | | | | | | | | | | | | | | | | | | | | |
| L6 | 0 | 0 | 0 | 0 | 0 | | | | | | | | | | | | | | | | | | | | | | |
| L7 | 0 | 0 | 0 | 0 | 0.0505 | 0.5112 | | | | | | | | | | | | | | | | | | | | | |
| L8 | 0 | 0 | 0 | 0.0212 | 0 | 0 | 0 | | | | | | | | | | | | | | | | | | | | |
| L9 | 0 | 0 | 0 | 0 | 0 | 0 | 0 | 0 | | | | | | | | | | | | | | | | | | | |
| L10 | 0 | 0 | 0 | 0 | 0 | 0 | 0 | 0 | 0.3004 | | | | | | | | | | | | | | | | | | |
| L11 | 0 | 0 | 0 | 0 | 0 | 0 | 0 | 0 | 0 | 0 | | | | | | | | | | | | | | | | | |
| L12 | 0 | 0 | 0 | 0 | 0 | 0 | 0 | 0 | 0 | 0 | 0.3951 | | | | | | | | | | | | | | | | |
| L13 | 0 | 0 | 0 | 0 | 0 | 0 | 0 | 0 | 0 | 0 | 0 | 0 | | | | | | | | | | | | | | | |
| L14 | 0 | 0 | 0 | 0 | 0 | -0.0448 | 0 | 0 | 0 | 0 | 0 | 0 | 0.3443 | | | | | | | | | | | | | | |
| L18 | 0 | 0 | 0 | 0 | 0 | 0 | 0 | 0 | 0 | 0 | 0 | 0 | 0 | 0 | | | | | | | | | | | | | |
| H1 | 0 | 0 | 0 | 0.1196 | 0 | 0 | 0 | 0 | 0 | 0 | 0 | 0 | 0 | 0 | 0 | | | | | | | | | | | | |
| H2 | 0 | 0 | 0 | 0 | 0 | 0 | 0 | 0 | 0 | 0 | 0 | 0 | 0 | 0 | 0 | -0.2547 | | | | | | | | | | | |
| H3 | 0 | 0.1199 | 0 | 0 | 0 | 0 | 0 | 0 | 0 | 0 | 0 | 0 | 0 | 0 | 0.2422 | 0 | 0 | | | | | | | | | | |
| H4 | 0 | 0 | 0 | 0 | 0 | 0 | 0 | 0 | 0 | 0 | 0 | 0 | 0 | 0 | 0 | 0 | 0 | 0.0801 | | | | | | | | | |
| H5 | 0 | 0.0648 | 0 | 0 | 0 | 0 | 0 | 0 | 0 | 0 | 0 | 0 | 0 | 0 | 0.0105 | 0 | 0 | 0.1884 | 0.2239 | | | | | | | | |
| H6 | 0 | 0 | 0 | 0 | 0 | 0 | 0 | 0 | 0 | 0 | 0 | 0 | 0 | 0.0969 | 0 | 0 | 0 | 0 | 0 | 0 | | | | | | | |
| H7 | 0 | 0 | 0 | 0 | 0 | 0 | 0 | 0 | 0 | 0 | 0 | 0 | 0 | 0 | 0 | 0 | 0 | 0 | 0 | 0 | 0.3435 | | | | | | |
| H8 | 0 | 0 | 0 | 0 | 0 | 0 | 0 | 0 | 0 | 0 | 0 | 0 | 0 | 0 | 0 | 0 | 0 | 0 | 0 | 0 | 0 | 0 | | | | | |
| H9 | 0 | 0 | 0 | 0 | 0 | 0 | 0 | 0 | 0 | 0 | 0 | 0 | 0 | 0 | 0 | 0 | 0 | 0 | 0 | 0 | 0 | 0 | 0 | | | | |
| H10 | 0 | 0 | 0 | 0 | 0 | 0 | 0 | 0 | 0 | 0 | 0 | 0 | 0 | 0 | 0 | 0 | 0 | 0 | 0 | 0 | 0 | 0 | 0 | 0.1619 | | | |
| H11 | 0 | 0 | 0 | 0 | 0 | 0 | 0 | 0 | 0 | 0 | 0 | 0 | 0 | 0 | 0 | 0 | 0 | 0 | 0.0743 | 0 | 0 | 0 | 0 | 0 | 0 | | |
| H12 | 0 | 0 | 0 | 0 | 0 | 0 | 0 | 0 | 0 | 0 | 0 | 0 | 0 | 0 | 0 | 0 | 0 | -0.0147 | 0 | 0 | 0 | 0 | 0 | 0 | 0 | 0 | |
| H13 | 0 | 0 | 0 | 0 | 0 | 0 | 0 | 0 | 0 | 0 | 0 | 0 | 0 | 0 | 0 | 0 | 0 | 0 | 0 | 0 | 0 | 0 | 0 | 0 | 0 | 0 | 0 |

## (H). Hong Kong

| | L1 | L2 | L3 | L4 | L5 | L6 | L7 | L8 | L9 | L10 | L11 | L12 | L13 | L14 | L18 | H1 | H2 | H3 | H4 | H5 | H6 | H7 | H8 | H9 | H10 | H11 | H12 |
|---|---|---|---|---|---|---|---|---|---|---|---|---|---|---|---|---|---|---|---|---|---|---|---|---|---|---|---|
| L1 | | | | | | | | | | | | | | | | | | | | | | | | | | | |
| L2 | 0.1899 | | | | | | | | | | | | | | | | | | | | | | | | | | |
| L3 | 0 | -0.09 | | | | | | | | | | | | | | | | | | | | | | | | | |
| L4 | -0.0863 | 0.0704 | 0.1309 | | | | | | | | | | | | | | | | | | | | | | | | |
| L5 | -0.0351 | 0.061 | 0.024 | 0.5518 | | | | | | | | | | | | | | | | | | | | | | | |
| L6 | 0 | 0.0884 | -0.0862 | 0 | 0 | | | | | | | | | | | | | | | | | | | | | | |
| L7 | 0 | 0.0884 | 0 | 0.0044 | 0.0143 | 0.5676 | | | | | | | | | | | | | | | | | | | | | |
| L8 | 0 | 0.0012 | 0.0801 | 0.074 | 0.1106 | -0.0045 | 0 | | | | | | | | | | | | | | | | | | | | |
| L9 | 0.0632 | 0 | 0 | 0 | 0 | 0 | 0 | -0.0293 | | | | | | | | | | | | | | | | | | | |
| L10 | 0 | 0.0648 | -0.0186 | 0 | 0 | 0.0041 | 0.0574 | -0.0354 | 0.1548 | | | | | | | | | | | | | | | | | | |
| L11 | 0 | 0 | 0 | 0.0033 | 0.0401 | 0 | 0 | 0 | -0.0549 | -0.0533 | | | | | | | | | | | | | | | | | |
| L12 | -0.0048 | 0 | 0 | 0.0018 | 0.0482 | 0.0277 | 0.0127 | 0 | -0.0392 | -0.0082 | 0.4234 | | | | | | | | | | | | | | | | |
| L13 | 0 | -0.0048 | 0.0552 | 0.0151 | 0 | -0.062 | -0.0317 | 0.0642 | 0 | -0.0063 | 0 | 0 | | | | | | | | | | | | | | | |
| L14 | 0 | 0 | 0.0633 | 0 | 0 | -0.0592 | -0.048 | 0.1154 | 0 | -0.0619 | 0 | 0 | 0.5475 | | | | | | | | | | | | | | |
| L18 | 0 | 0 | 0.0065 | -0.0223 | -0.0081 | -0.046 | 0 | 0 | 0 | 0.0327 | 0 | -0.0827 | 0.0404 | 0.0616 | | | | | | | | | | | | | |
| H1 | 0 | 0 | 0.018 | 0.0521 | 0.015 | 0 | 0 | 0.0589 | 0 | -0.0199 | 0 | 0 | 0.0114 | 0.0691 | 0.0001 | | | | | | | | | | | | |
| H2 | 0 | 0 | 0 | -0.0221 | -0.0371 | 0.013 | 0 | 0 | 0 | 0 | 0 | 0 | 0 | -0.0205 | 0.0000 | -0.2344 | | | | | | | | | | | |
| H3 | 0.0555 | 0.0197 | 0 | 0 | -0.0031 | 0 | 0 | -0.017 | 0 | 0.0114 | 0 | 0 | 0 | 0 | 0.0395 | 0 | 0.1915 | | | | | | | | | | |
| H4 | 0 | 0 | 0 | 0 | -0.0079 | 0 | 0 | 0 | 0 | 0 | 0 | 0 | 0 | 0 | 0.0384 | 0 | 0.0610 | 0.2028 | | | | | | | | | |
| H5 | 0.0029 | 0.0372 | 0 | 0 | 0 | -0.0093 | 0 | 0 | 0 | 0 | 0 | 0 | 0.0114 | 0 | 0.0674 | 0 | 0 | 0.1584 | 0.2827 | | | | | | | | |
| H6 | 0 | 0 | 0 | -0.0149 | 0 | -0.0202 | -0.0379 | 0 | 0 | 0 | 0 | 0 | 0.0085 | 0.0249 | 0.0355 | 0.0177 | 0 | 0 | 0 | 0 | | | | | | | |
| H7 | 0 | 0 | 0 | 0 | -0.0237 | -0.0294 | -0.0148 | 0 | 0 | 0 | 0 | 0 | 0 | 0 | 0.0006 | 0.0253 | 0 | 0.0170 | 0.0297 | 0.0183 | 0.4945 | | | | | | |
| H8 | 0 | 0 | 0 | 0.0108 | 0.0484 | 0 | 0 | 0 | 0 | 0 | 0 | 0.03 | 0 | 0 | 0.0000 | 0.0005 | 0 | 0 | 0 | 0 | 0.0446 | 0.2150 | | | | | |
| H9 | 0 | 0 | 0 | 0 | 0 | 0 | 0 | 0 | 0.0119 | 0 | 0 | 0 | 0 | 0 | 0.0145 | 0 | 0.0002 | 0 | 0 | 0.0227 | 0 | 0 | -0.0516 | | | | |
| H10 | 0.0247 | 0 | 0 | 0 | 0 | 0 | 0.0258 | -0.0352 | 0 | 0 | 0 | 0 | 0 | -0.0031 | 0.0356 | 0 | 0 | 0.0136 | 0 | 0 | -0.0023 | 0 | -0.0321 | 0.3621 | | | |
| H11 | 0.1228 | 0 | 0.0261 | -0.036 | -0.0658 | -0.1096 | -0.038 | 0 | 0 | 0 | 0 | -0.0409 | 0.0097 | 0.0352 | 0.1449 | -0.0061 | 0 | 0 | 0.0003 | 0.1701 | 0.0479 | 0.0233 | 0 | 0.0165 | 0.0087 | | |
| H12 | -0.0449 | 0 | 0 | 0 | 0 | 0 | 0 | 0.0196 | 0 | 0 | 0.0073 | 0.0077 | 0 | 0 | 0.0000 | 0 | 0 | -0.0746 | -0.0178 | -0.0046 | 0 | -0.0146 | -0.0153 | -0.0123 | -0.0140 | -0.1847 | |
| H13 | 0 | 0 | 0 | 0 | 0 | 0.0192 | 0.0368 | 0 | 0 | 0 | 0 | 0 | 0 | 0 | -0.0009 | -0.0023 | 0 | -0.0213 | -0.0396 | 0 | -0.1390 | -0.0524 | -0.0404 | 0.0226 | 0 | -0.0035 | 0.2785 |

## (I). India

| | L1 | L2 | L3 | L4 | L5 | L6 | L7 | L8 | L9 | L10 | L11 | L12 | L13 | L14 | L18 | H1 | H2 | H3 | H4 | H5 | H6 | H7 | H8 | H9 | H10 | H11 | H12 |
|---|---|---|---|---|---|---|---|---|---|---|---|---|---|---|---|---|---|---|---|---|---|---|---|---|---|---|---|
| L1 | | | | | | | | | | | | | | | | | | | | | | | | | | | |
| L2 | 0.0695 | | | | | | | | | | | | | | | | | | | | | | | | | | |
| L3 | 0 | 0 | | | | | | | | | | | | | | | | | | | | | | | | | |
| L4 | -0.0219 | 0 | 0.1957 | | | | | | | | | | | | | | | | | | | | | | | | |
| L5 | 0 | 0 | 0.309 | 0.2412 | | | | | | | | | | | | | | | | | | | | | | | |
| L6 | 0 | 0.0853 | 0 | 0 | 0 | | | | | | | | | | | | | | | | | | | | | | |
| L7 | 0.0274 | 0.0747 | 0 | 0 | 0 | 0.5481 | | | | | | | | | | | | | | | | | | | | | |
| L8 | 0 | 0 | 0.0624 | 0 | 0.2335 | 0.1144 | 0.0278 | | | | | | | | | | | | | | | | | | | | |
| L9 | 0 | 0 | 0 | 0 | -0.0183 | 0 | 0 | 0 | | | | | | | | | | | | | | | | | | | |
| L10 | 0 | 0.008 | 0 | 0 | -0.0268 | 0 | 0 | 0 | 0.3646 | | | | | | | | | | | | | | | | | | |
| L11 | 0 | 0 | 0.0273 | 0 | 0.1301 | 0 | 0.0181 | 0.0689 | -0.0129 | -0.0693 | | | | | | | | | | | | | | | | | |
| L12 | 0 | 0 | 0.0526 | 0 | 0.039 | 0.0242 | 0.0296 | 0 | -0.0457 | -0.0013 | 0.6841 | | | | | | | | | | | | | | | | |
| L13 | 0 | 0 | 0 | 0 | 0 | -0.1148 | 0 | -0.0505 | 0 | 0 | 0 | 0 | | | | | | | | | | | | | | | |
| L14 | 0 | 0 | 0 | 0 | 0 | -0.0517 | 0 | -0.0125 | 0 | 0 | 0 | 0 | 0.5176 | | | | | | | | | | | | | | |
| L18 | 0 | 0.0808 | 0 | 0 | 0 | 0 | 0 | 0 | 0 | 0 | 0 | 0 | 0.0082 | 0 | | | | | | | | | | | | | |
| H1 | 0 | 0 | 0 | 0 | 0 | 0 | 0 | 0 | 0 | 0 | 0 | 0 | 0 | 0 | 0.0625 | | | | | | | | | | | | |
| H2 | 0.1075 | 0 | 0 | -0.0572 | 0 | 0 | 0 | 0 | 0 | 0 | 0 | 0 | 0 | 0 | 0 | -0.3615 | | | | | | | | | | | |
| H3 | 0 | 0.001 | 0 | 0 | 0 | 0 | 0 | 0 | 0 | 0 | 0 | 0 | 0 | 0 | 0.2307 | 0 | 0.0405 | | | | | | | | | | |
| H4 | 0 | 0.0414 | 0 | 0 | 0 | 0.008 | 0 | 0 | 0 | 0 | 0 | 0 | 0 | 0 | 0 | 0 | 0.0191 | 0.1933 | | | | | | | | | |
| H5 | 0 | 0.0286 | 0 | 0 | 0 | 0 | 0 | 0 | 0 | 0 | 0 | 0 | 0 | 0 | 0.1113 | 0 | 0 | 0.1971 | 0.207 | | | | | | | | |
| H6 | 0 | 0 | 0 | 0 | 0 | 0 | 0 | 0 | 0 | -0.0064 | 0 | 0 | 0 | 0.038 | 0.0023 | 0 | 0 | 0 | 0 | 0 | | | | | | | |
| H7 | 0 | 0 | 0 | 0 | 0 | 0 | 0 | 0 | 0 | -0.0377 | 0 | 0 | 0.0115 | 0.0017 | 0.0037 | 0 | 0 | 0 | 0 | 0.0426 | 0.6016 | | | | | | |
| H8 | 0 | 0 | 0 | 0 | 0 | 0 | 0 | 0.0028 | 0 | -0.0337 | 0.0515 | 0.0046 | 0 | 0 | 0 | 0 | 0 | 0 | 0 | 0.0782 | 0.0504 | 0.1751 | | | | | |
| H9 | 0 | 0 | 0 | 0 | 0 | 0 | 0 | 0 | 0 | 0 | 0 | 0 | 0 | 0 | 0 | 0 | 0 | 0.0196 | 0 | 0.0712 | 0 | 0 | 0 | | | | |
| H10 | 0 | 0 | 0 | 0 | 0 | 0 | 0 | 0 | 0 | 0 | 0 | 0 | 0 | 0 | 0 | 0 | 0 | 0 | 0 | 0 | 0 | 0 | 0 | 0.4148 | | | |
| H11 | 0 | 0 | 0 | 0 | 0 | 0 | 0 | -0.095 | 0 | 0 | 0 | 0 | 0.043 | 0.0344 | 0 | 0 | 0 | 0.0254 | 0 | 0.0973 | 0 | 0 | 0 | 0.0891 | 0.1861 | | |
| H12 | 0 | 0 | 0.0704 | 0 | 0 | 0 | 0 | 0.0313 | 0 | 0 | 0 | 0 | 0 | 0 | 0 | 0 | 0 | 0 | 0 | 0 | 0 | 0 | 0 | 0 | 0 | -0.0729 | |
| H13 | 0 | 0 | 0 | 0 | 0 | 0 | 0 | 0 | 0 | 0 | -0.018 | 0 | 0 | 0 | 0 | 0 | 0 | 0 | 0 | 0 | -0.0129 | -0.0541 | -0.0663 | 0 | 0 | 0 | 0 |

## (J). Indonesia

| | L1 | L2 | L3 | L4 | L5 | L6 | L7 | L8 | L9 | L10 | L11 | L12 | L13 | L14 | L18 | H1 | H2 | H3 | H4 | H5 | H6 | H7 | H8 | H9 | H10 | H11 | H12 |
|---|---|---|---|---|---|---|---|---|---|---|---|---|---|---|---|---|---|---|---|---|---|---|---|---|---|---|---|
| L1 | | | | | | | | | | | | | | | | | | | | | | | | | | | |
| L2 | 0.2138 | | | | | | | | | | | | | | | | | | | | | | | | | | |
| L3 | -0.0938 | -0.0129 | | | | | | | | | | | | | | | | | | | | | | | | | |
| L4 | -0.0687 | -0.0179 | 0.1688 | | | | | | | | | | | | | | | | | | | | | | | | |
| L5 | 0 | 0 | 0.2274 | 0.3562 | | | | | | | | | | | | | | | | | | | | | | | |
| L6 | 0.0875 | 0.1578 | 0 | -0.0123 | -0.0098 | | | | | | | | | | | | | | | | | | | | | | |
| L7 | 0.0844 | 0 | 0 | -0.0054 | 0 | 0.5242 | | | | | | | | | | | | | | | | | | | | | |
| L8 | 0 | -0.0218 | 0.0576 | 0.0578 | 0.079 | -0.0625 | 0 | | | | | | | | | | | | | | | | | | | | |
| L9 | 0.1005 | 0 | -0.0481 | -0.0612 | -0.061 | 0.0295 | 0.0141 | -0.2041 | | | | | | | | | | | | | | | | | | | |
| L10 | 0 | 0.0315 | -0.0828 | 0 | 0 | 0 | 0.0604 | -0.0647 | 0.175 | | | | | | | | | | | | | | | | | | |
| L11 | 0 | -0.0703 | 0.0065 | 0.0343 | 0.0347 | 0 | 0 | 0 | 0 | -0.0149 | | | | | | | | | | | | | | | | | |
| L12 | -0.0103 | 0 | 0 | 0 | 0 | 0 | 0 | 0 | 0.0106 | -0.0805 | 0.6975 | | | | | | | | | | | | | | | | |
| L13 | 0 | 0 | 0.0267 | 0.032 | 0 | 0 | 0 | 0.0091 | 0 | 0 | 0.032 | 0 | | | | | | | | | | | | | | | |
| L14 | 0 | 0 | 0 | 0.0801 | 0 | -0.0211 | -0.0143 | 0 | 0 | -0.0288 | 0 | 0 | 0.4395 | | | | | | | | | | | | | | |
| L18 | 0.0564 | 0.0819 | 0 | 0 | -0.0422 | 0.0309 | 0.0017 | -0.0388 | 0 | 0.0799 | 0 | -0.0414 | -0.0419 | -0.0555 | | | | | | | | | | | | | |
| H1 | 0 | 0 | 0 | 0 | 0 | -0.009 | -0.0231 | 0.088 | 0 | -0.0699 | 0 | 0 | 0.0596 | 0 | -0.0312 | | | | | | | | | | | | |
| H2 | 0.0284 | 0 | 0 | 0 | 0 | 0.0844 | 0.0258 | 0 | 0 | 0.0532 | 0.0046 | 0 | 0 | 0 | 0.0141 | -0.2993 | | | | | | | | | | | |
| H3 | 0.1029 | 0 | -0.0067 | -0.017 | -0.0188 | 0.0123 | 0.0231 | -0.0238 | 0.0034 | 0 | -0.007 | 0 | 0 | 0 | 0.0625 | -0.0947 | 0.1317 | | | | | | | | | | |
| H4 | 0 | 0 | -0.0508 | 0 | 0 | 0.0446 | 0 | 0 | 0 | 0 | -0.0069 | 0 | -0.0204 | -0.1007 | 0.0216 | -0.0447 | 0.1581 | 0.0439 | | | | | | | | | |
| H5 | 0.0897 | 0.0387 | 0 | 0 | 0 | 0 | 0 | 0 | 0 | 0 | 0 | 0 | -0.0266 | -0.0298 | 0.0831 | -0.0669 | 0.082 | 0.1341 | 0.227 | | | | | | | | |
| H6 | 0 | 0 | 0 | 0 | 0 | 0 | 0 | 0 | 0 | 0 | 0 | -0.0084 | 0 | 0.0069 | 0 | 0 | -0.0171 | -0.0636 | -0.016 | -0.1697 | | | | | | | |
| H7 | 0 | 0 | 0.002 | 0 | 0 | 0 | 0 | 0 | -0.0144 | 0 | 0 | -0.0058 | 0.0515 | 0 | 0 | 0.0642 | 0 | -0.0277 | -0.0693 | 0 | 0.612 | | | | | | |
| H8 | 0 | 0 | 0 | 0 | 0.0663 | 0 | 0 | 0.0378 | -0.0153 | 0 | 0.088 | 0 | 0.0214 | 0.0563 | 0 | 0.0142 | -0.019 | 0 | -0.0069 | 0 | 0.0993 | 0.111 | | | | | |
| H9 | 0.0032 | 0 | -0.0282 | -0.0546 | 0 | 0 | 0 | 0 | 0.0196 | 0 | 0 | 0 | -0.0095 | 0 | 0.0686 | 0 | 0 | 0 | 0 | 0.0877 | -0.0223 | -0.0118 | -0.1904 | | | | |
| H10 | 0 | 0 | 0 | 0 | 0 | 0 | 0.0025 | 0 | 0.0121 | 0.062 | 0 | -0.0155 | 0 | 0.0404 | 0.0428 | -0.025 | 0 | 0.0206 | 0 | 0 | -0.0317 | -0.004 | 0 | 0.5245 | | | |
| H11 | 0.021 | 0.1044 | 0 | 0 | 0 | 0 | 0 | 0 | 0 | 0.0092 | 0 | 0 | -0.0341 | 0 | 0.0264 | 0 | 0 | 0 | 0 | 0 | 0 | -0.0237 | 0 | 0.1308 | 0.2265 | | |
| H12 | 0 | 0 | 0 | 0 | 0 | 0 | 0 | 0 | 0 | -0.0037 | 0 | 0.0243 | 0 | 0 | -0.0026 | 0 | 0 | 0 | -0.1475 | -0.0369 | 0 | 0 | 0.0228 | -0.0081 | 0 | -0.0928 | |
| H13 | 0 | 0 | 0 | 0 | 0 | 0.0039 | 0 | -0.0053 | 0 | 0 | 0 | -0.0042 | 0 | -0.0506 | 0 | -0.0378 | 0 | 0 | 0 | 0 | -0.0257 | -0.0709 | -0.0199 | 0 | 0.0395 | 0.0421 | -0.4756 |

## (K). Italy

| | L1 | L2 | L3 | L4 | L5 | L6 | L7 | L8 | L9 | L10 | L11 | L12 | L13 | L14 | L18 | H1 | H2 | H3 | H4 | H5 | H6 | H7 | H8 | H9 | H10 | H11 | H12 |
|---|---|---|---|---|---|---|---|---|---|---|---|---|---|---|---|---|---|---|---|---|---|---|---|---|---|---|---|
| L1 | | | | | | | | | | | | | | | | | | | | | | | | | | | |
| L2 | 0 | | | | | | | | | | | | | | | | | | | | | | | | | | |
| L3 | 0 | 0.0834 | | | | | | | | | | | | | | | | | | | | | | | | | |
| L4 | 0 | 0 | 0.121 | | | | | | | | | | | | | | | | | | | | | | | | |
| L5 | 0 | 0.0348 | 0.0147 | 0.2591 | | | | | | | | | | | | | | | | | | | | | | | |
| L6 | 0 | 0 | 0 | 0 | 0 | | | | | | | | | | | | | | | | | | | | | | |
| L7 | 0 | 0 | 0 | 0 | 0 | 0.4114 | | | | | | | | | | | | | | | | | | | | | |
| L8 | 0 | 0 | 0.0566 | 0.0836 | 0 | 0 | 0 | | | | | | | | | | | | | | | | | | | | |
| L9 | 0 | 0 | 0 | 0 | 0 | 0 | 0 | 0 | | | | | | | | | | | | | | | | | | | |
| L10 | 0 | 0 | 0 | 0 | 0 | 0 | 0 | 0 | 0.1848 | | | | | | | | | | | | | | | | | | |
| L11 | 0 | 0 | 0 | 0 | 0 | 0 | 0 | 0.0885 | 0 | 0 | | | | | | | | | | | | | | | | | |
| L12 | 0 | 0 | 0 | 0 | 0.108 | 0 | 0 | 0 | 0 | 0 | 0.2439 | | | | | | | | | | | | | | | | |
| L13 | 0 | 0 | 0 | 0 | 0 | 0 | -0.026 | 0 | 0 | 0 | 0 | 0 | | | | | | | | | | | | | | | |
| L14 | 0 | 0 | 0 | 0 | 0 | -0.0092 | 0 | 0 | 0 | 0 | 0 | 0 | 0.5628 | | | | | | | | | | | | | | |
| L18 | 0 | 0 | 0 | 0 | 0 | 0 | 0 | 0 | 0 | 0 | 0 | 0 | 0.0322 | 0 | | | | | | | | | | | | | |
| H1 | -0.0317 | 0 | 0 | 0 | 0 | 0 | 0 | 0 | 0 | 0 | 0 | 0 | 0 | 0 | 0.0036 | | | | | | | | | | | | |
| H2 | 0.1583 | 0 | 0 | -0.0254 | -0.0331 | 0 | 0 | 0 | 0 | 0 | 0 | 0 | 0 | 0 | 0 | -0.2689 | | | | | | | | | | | |
| H3 | 0 | 0 | 0 | 0 | 0 | 0 | 0 | 0 | 0 | 0 | 0 | 0 | 0 | 0 | 0 | 0 | 0 | | | | | | | | | | |
| H4 | 0 | 0 | 0 | 0 | 0 | 0 | 0 | 0 | 0 | 0 | 0 | 0 | 0 | 0 | 0 | 0 | 0 | 0.0318 | | | | | | | | | |
| H5 | 0 | 0 | 0 | 0 | 0 | 0 | 0 | 0 | 0 | 0 | 0 | 0 | 0 | 0 | 0 | 0 | 0 | 0.0726 | 0.3363 | | | | | | | | |
| H6 | 0 | 0 | 0 | 0 | 0 | 0 | 0 | 0 | 0 | 0 | 0 | 0 | 0 | 0 | 0 | 0 | 0 | 0 | 0 | 0 | | | | | | | |
| H7 | 0 | 0 | 0 | 0 | 0 | 0 | 0 | 0 | 0 | 0 | 0 | 0 | 0.0068 | 0.0736 | 0 | 0 | -0.0165 | 0.0431 | 0 | 0 | 0.3098 | | | | | | |
| H8 | 0 | 0 | 0 | 0 | 0 | 0 | 0 | 0 | 0 | 0 | 0 | 0 | 0 | 0 | 0 | 0.0514 | -0.0449 | 0 | 0 | 0 | 0 | 0.0239 | | | | | |
| H9 | 0 | 0 | 0 | 0 | 0 | 0 | 0 | 0 | 0 | 0 | 0 | 0 | 0 | 0 | 0 | 0 | 0 | 0 | 0 | 0 | 0 | 0 | 0 | | | | |
| H10 | 0 | 0 | 0 | 0 | 0 | 0 | 0 | 0 | 0 | 0 | 0 | 0 | 0 | 0 | 0 | 0 | 0 | 0 | 0 | 0 | 0 | 0 | 0 | 0.2532 | | | |
| H11 | 0 | 0 | 0 | 0 | 0 | 0 | 0 | 0 | 0 | 0 | 0 | 0 | 0.0551 | 0.003 | 0 | 0 | 0 | 0 | 0 | 0.1092 | 0 | 0 | 0 | 0 | 0 | | |
| H12 | 0 | 0 | 0 | 0 | 0 | 0 | 0 | 0 | 0 | 0 | 0 | 0 | 0 | 0 | 0 | 0 | 0 | 0 | 0 | 0 | 0 | 0 | 0 | 0 | 0 | 0 | |
| H13 | 0 | 0 | 0 | 0 | 0 | 0 | 0 | 0 | 0 | 0 | 0 | 0 | 0 | 0 | 0 | 0 | 0 | 0 | 0 | 0 | 0 | 0 | 0 | 0 | 0 | 0 | 0 |

## (L). Lebanon

| | L1 | L2 | L3 | L4 | L5 | L6 | L7 | L8 | L9 | L10 | L11 | L12 | L13 | L14 | L18 | H1 | H2 | H3 | H4 | H5 | H6 | H7 | H8 | H9 | H10 | H11 | H12 |
|---|---|---|---|---|---|---|---|---|---|---|---|---|---|---|---|---|---|---|---|---|---|---|---|---|---|---|---|
| L1 | | | | | | | | | | | | | | | | | | | | | | | | | | | |
| L2 | 0.1752 | | | | | | | | | | | | | | | | | | | | | | | | | | |
| L3 | -0.0702 | -0.0367 | | | | | | | | | | | | | | | | | | | | | | | | | |
| L4 | -0.096 | -0.007 | 0.1491 | | | | | | | | | | | | | | | | | | | | | | | | |
| L5 | -0.029 | 0 | 0.1089 | 0.0889 | | | | | | | | | | | | | | | | | | | | | | | |
| L6 | 0.0397 | 0 | 0 | -0.0379 | 0 | | | | | | | | | | | | | | | | | | | | | | |
| L7 | 0 | 0.1513 | 0 | 0 | 0 | 0.5704 | | | | | | | | | | | | | | | | | | | | | |
| L8 | -0.0376 | 0 | 0.1744 | 0.183 | 0.2027 | 0 | 0.1481 | | | | | | | | | | | | | | | | | | | | |
| L9 | 0 | 0 | 0 | 0 | 0 | 0 | 0 | 0 | | | | | | | | | | | | | | | | | | | |
| L10 | 0 | 0.0563 | -0.0499 | -0.1249 | 0 | 0 | 0 | 0 | 0.2729 | | | | | | | | | | | | | | | | | | |
| L11 | 0 | 0 | 0 | 0 | 0.128 | 0 | 0 | 0 | -0.0071 | -0.0212 | | | | | | | | | | | | | | | | | |
| L12 | 0 | 0 | 0.1542 | 0 | 0 | 0.0389 | 0 | 0 | -0.1856 | -0.1315 | 0.2737 | | | | | | | | | | | | | | | | |
| L13 | 0 | 0 | 0.0183 | 0.0227 | 0 | -0.0837 | 0 | 0 | 0 | -0.0396 | 0.0398 | 0.0082 | | | | | | | | | | | | | | | |
| L14 | 0 | 0 | 0.0105 | 0 | 0.0767 | 0 | -0.0513 | 0 | 0 | -0.032 | 0 | 0 | 0.6324 | | | | | | | | | | | | | | |
| L18 | 0 | 0.0727 | 0 | -0.0404 | 0 | 0 | 0 | 0 | 0.0277 | 0.0694 | 0 | -0.0255 | 0.0228 | 0 | | | | | | | | | | | | | |
| H1 | -0.058 | 0 | 0 | 0 | 0 | 0 | 0 | 0 | 0 | 0 | 0 | 0 | 0 | 0 | 0.0692 | | | | | | | | | | | | |
| H2 | 0.1547 | 0 | -0.0603 | 0 | -0.0138 | 0 | 0.0216 | -0.0045 | 0 | 0 | 0 | 0 | 0 | -0.0993 | 0 | -0.4669 | | | | | | | | | | | |
| H3 | 0.1063 | 0.0051 | 0 | 0 | 0 | 0 | 0.0568 | 0 | 0 | 0 | 0 | 0 | 0 | 0 | 0.1684 | 0 | 0.1064 | | | | | | | | | | |
| H4 | 0 | 0.04 | 0 | -0.0115 | 0 | 0 | 0 | 0 | 0 | 0 | 0 | 0 | 0 | 0 | 0 | 0 | 0.0316 | 0.1869 | | | | | | | | | |
| H5 | 0.0572 | 0 | 0 | 0 | 0 | 0 | 0 | 0 | 0 | 0 | 0 | 0 | 0.0485 | 0.0416 | 0.074 | 0 | 0 | 0.1475 | 0.3344 | | | | | | | | |
| H6 | 0 | 0 | 0 | 0 | 0 | 0 | 0 | 0 | 0 | 0 | 0 | 0 | 0.0235 | 0 | 0 | 0 | -0.0287 | 0 | 0 | 0 | | | | | | | |
| H7 | 0 | 0 | 0 | 0.0011 | 0.0303 | 0 | 0 | 0 | 0 | 0 | 0 | 0 | 0.0076 | 0.0272 | 0 | 0.0062 | -0.039 | 0 | 0 | 0 | 0.5088 | | | | | | |
| H8 | 0 | 0 | 0 | 0 | 0 | 0 | 0 | 0 | 0 | 0 | 0 | 0 | 0 | 0.0633 | 0 | 0.0146 | -0.0294 | 0 | 0 | 0 | 0.0346 | 0.3444 | | | | | |
| H9 | 0 | 0 | -0.0117 | 0 | 0 | 0.0302 | 0 | 0 | 0 | 0 | 0 | 0 | 0 | 0 | 0.0293 | 0 | 0 | 0.0625 | 0 | 0.0162 | -0.0619 | 0 | -0.0152 | | | | |
| H10 | 0 | 0.069 | 0 | 0 | 0 | 0 | 0 | 0 | 0 | 0.0522 | -0.0157 | -0.0539 | 0 | 0 | 0 | 0 | 0.005 | 0 | 0 | 0 | 0 | 0 | -0.1057 | 0.1243 | | | |
| H11 | 0 | 0 | 0 | 0 | 0 | 0 | 0 | -0.0111 | 0 | 0 | 0 | 0 | 0.0602 | 0.0091 | 0.0575 | 0 | 0 | 0.1034 | 0 | 0.1463 | 0 | 0.0121 | 0 | 0.0829 | 0.0877 | | |
| H12 | -0.0623 | 0 | 0.0745 | 0 | 0 | 0 | 0 | 0.0292 | 0 | 0 | 0 | 0 | 0 | 0 | 0 | 0 | 0 | 0 | -0.0421 | -0.0518 | 0 | 0 | 0 | -0.0629 | -0.047 | -0.1339 | |
| H13 | 0 | 0 | 0 | 0 | 0 | 0 | 0.0475 | 0 | 0.1015 | 0 | 0 | 0 | -0.0357 | -0.0129 | 0 | 0 | 0.0391 | 0 | 0 | 0 | -0.1729 | -0.0584 | -0.0745 | 0.0755 | 0.0334 | 0 | -0.0414 |

## (M). Libya

| | L1 | L2 | L3 | L4 | L5 | L6 | L7 | L8 | L9 | L10 | L11 | L12 | L13 | L14 | L18 | H1 | H2 | H3 | H4 | H5 | H6 | H7 | H8 | H9 | H10 | H11 | H12 |
|---|---|---|---|---|---|---|---|---|---|---|---|---|---|---|---|---|---|---|---|---|---|---|---|---|---|---|---|
| L1 | | | | | | | | | | | | | | | | | | | | | | | | | | | |
| L2 | 0.1695 | | | | | | | | | | | | | | | | | | | | | | | | | | |
| L3 | -0.1161 | 0 | | | | | | | | | | | | | | | | | | | | | | | | | |
| L4 | 0 | 0 | 0.1192 | | | | | | | | | | | | | | | | | | | | | | | | |
| L5 | 0 | 0 | 0.1679 | 0.3066 | | | | | | | | | | | | | | | | | | | | | | | |
| L6 | 0.0726 | 0.0931 | 0 | 0 | 0 | | | | | | | | | | | | | | | | | | | | | | |
| L7 | 0 | 0.0314 | 0 | 0.0032 | 0.0155 | 0.6457 | | | | | | | | | | | | | | | | | | | | | |
| L8 | 0 | 0 | 0 | 0.0806 | 0.0672 | 0 | 0 | | | | | | | | | | | | | | | | | | | | |
| L9 | 0 | 0.0482 | 0 | 0 | 0 | 0.0021 | 0.0087 | 0 | | | | | | | | | | | | | | | | | | | |
| L10 | 0 | 0.038 | 0 | 0 | 0 | 0 | 0 | 0 | 0.2181 | | | | | | | | | | | | | | | | | | |
| L11 | 0 | 0 | 0.0019 | 0 | 0 | 0 | 0.0275 | 0.0677 | 0 | 0 | | | | | | | | | | | | | | | | | |
| L12 | 0 | 0 | 0 | 0 | 0.0582 | 0.0145 | 0.0141 | 0.151 | 0 | -0.0053 | 0.5304 | | | | | | | | | | | | | | | | |
| L13 | 0 | 0 | 0 | 0 | 0 | -0.082 | -0.0032 | 0 | -0.0533 | 0 | 0 | 0 | | | | | | | | | | | | | | | |
| L14 | 0 | 0 | 0 | 0 | 0.0341 | 0 | 0 | 0 | -0.0353 | 0 | 0 | 0.0119 | 0.4506 | | | | | | | | | | | | | | |
| L18 | 0 | 0.006 | 0 | 0 | 0 | 0 | 0 | -0.0276 | 0 | 0.015 | 0 | -0.0374 | 0 | 0 | | | | | | | | | | | | | |
| H1 | 0 | 0 | 0 | 0 | 0.0521 | 0 | 0 | 0 | 0 | 0 | 0 | 0 | 0.0058 | 0 | 0 | | | | | | | | | | | | |
| H2 | 0.113 | 0 | 0 | 0 | 0 | 0 | 0 | 0 | 0 | 0 | 0 | 0 | 0 | 0 | 0 | -0.4823 | | | | | | | | | | | |
| H3 | 0 | 0.0301 | 0 | 0 | 0 | 0 | 0 | 0 | 0 | 0 | 0 | 0 | 0 | 0 | 0 | 0 | 0.142 | | | | | | | | | | |
| H4 | 0.0332 | 0 | 0 | 0 | 0 | 0 | 0 | 0 | 0 | 0 | 0 | 0 | 0 | 0 | 0 | 0 | 0.0329 | 0.0879 | | | | | | | | | |
| H5 | 0.0375 | 0 | 0 | 0 | 0 | 0 | 0 | 0 | 0 | 0 | 0 | 0 | 0 | 0 | 0 | 0 | 0 | 0.1958 | 0.2258 | | | | | | | | |
| H6 | 0 | -0.0265 | 0 | 0 | 0 | 0 | 0 | 0 | 0 | 0 | 0 | 0 | 0 | 0.0074 | 0 | 0 | 0 | 0 | 0 | -0.0144 | | | | | | | |
| H7 | 0 | 0 | 0 | 0 | 0 | 0 | 0 | 0 | 0 | 0 | 0 | 0 | 0.0262 | 0 | 0 | 0 | 0 | 0 | 0 | 0 | 0.3719 | | | | | | |
| H8 | 0 | 0 | 0 | 0 | 0 | 0 | 0 | 0 | 0 | 0 | 0 | 0.0196 | 0.0667 | 0.0707 | 0 | 0 | 0 | 0 | 0 | 0 | 0.0278 | 0.3071 | | | | | |
| H9 | 0 | 0.107 | 0 | 0 | 0 | 0.0356 | 0.0113 | 0 | 0.0069 | 0 | 0 | 0 | 0 | 0 | 0 | 0 | 0 | 0.0011 | 0 | 0 | -0.0283 | 0 | 0 | | | | |
| H10 | 0 | 0.0088 | 0 | 0 | 0 | 0 | 0 | 0 | 0.0271 | 0.0752 | 0 | 0 | 0 | 0 | 0.0451 | 0 | 0 | 0 | 0 | 0 | 0 | 0 | 0 | 0.2437 | | | |
| H11 | 0 | 0 | 0 | 0 | 0 | 0 | 0 | -0.0603 | 0 | 0 | 0 | 0 | 0 | 0 | 0.0331 | 0 | 0 | 0 | 0 | 0.0663 | 0 | 0 | 0 | 0.0823 | 0.1703 | | |
| H12 | 0 | 0 | 0.0035 | 0 | 0 | 0 | 0 | 0 | 0 | 0 | 0 | 0.0106 | 0 | 0 | -0.0407 | 0 | -0.012 | 0 | 0 | -0.1394 | 0 | 0 | 0 | 0 | -0.0159 | -0.1483 | |
| H13 | 0 | 0 | 0 | 0 | 0 | 0 | 0 | 0 | 0 | 0 | 0 | 0 | 0 | 0 | 0 | 0 | 0 | 0 | 0 | 0 | -0.0645 | 0 | -0.0628 | 0 | 0 | 0 | -0.0712 |

## (N). Macau

| | L1 | L2 | L3 | L4 | L5 | L6 | L7 | L8 | L9 | L10 | L11 | L12 | L13 | L14 | L18 | H1 | H2 | H3 | H4 | H5 | H6 | H7 | H8 | H9 | H10 | H11 | H12 |
|---|---|---|---|---|---|---|---|---|---|---|---|---|---|---|---|---|---|---|---|---|---|---|---|---|---|---|---|
| L1 | | | | | | | | | | | | | | | | | | | | | | | | | | | |
| L2 | 0.0193 | | | | | | | | | | | | | | | | | | | | | | | | | | |
| L3 | 0 | 0 | | | | | | | | | | | | | | | | | | | | | | | | | |
| L4 | 0 | 0 | 0.0713 | | | | | | | | | | | | | | | | | | | | | | | | |
| L5 | 0 | 0 | 0 | 0.3594 | | | | | | | | | | | | | | | | | | | | | | | |
| L6 | 0 | 0 | 0 | 0 | 0 | | | | | | | | | | | | | | | | | | | | | | |
| L7 | 0 | 0 | 0 | 0 | 0 | 0.5702 | | | | | | | | | | | | | | | | | | | | | |
| L8 | 0 | 0 | 0 | 0 | 0 | 0 | 0 | | | | | | | | | | | | | | | | | | | | |
| L9 | 0 | 0 | 0 | 0 | 0 | 0 | 0 | 0 | | | | | | | | | | | | | | | | | | | |
| L10 | 0 | 0 | 0 | 0 | 0 | 0 | 0 | 0 | 0.0771 | | | | | | | | | | | | | | | | | | |
| L11 | 0 | 0 | 0 | 0 | 0 | 0 | 0 | 0 | 0 | 0 | | | | | | | | | | | | | | | | | |
| L12 | 0 | 0 | 0 | 0 | 0 | 0 | 0 | 0 | -0.0058 | 0 | 0.4958 | | | | | | | | | | | | | | | | |
| L13 | 0 | 0 | 0 | 0 | 0 | -0.0511 | 0 | 0 | 0 | 0 | 0 | 0 | | | | | | | | | | | | | | | |
| L14 | 0 | 0 | 0 | 0 | 0 | -0.0116 | 0 | 0 | 0 | 0 | 0 | 0 | 0.4308 | | | | | | | | | | | | | | |
| L18 | 0 | 0 | 0 | 0 | 0 | 0 | 0 | 0 | 0 | 0 | 0 | 0 | 0 | 0 | | | | | | | | | | | | | |
| H1 | 0 | 0 | 0 | 0 | 0 | 0 | 0 | 0 | 0 | 0 | 0 | 0 | 0 | 0 | 0 | | | | | | | | | | | | |
| H2 | 0 | 0 | 0 | 0 | 0 | 0 | 0 | 0 | 0 | 0 | 0 | 0 | 0 | 0 | 0 | -0.2699 | | | | | | | | | | | |
| H3 | 0 | 0 | 0 | 0 | 0 | 0 | 0 | 0 | 0 | 0 | 0 | 0 | 0 | 0 | 0 | 0 | 0 | | | | | | | | | | |
| H4 | 0 | 0 | 0 | 0 | 0 | 0 | 0 | 0 | 0 | 0 | 0 | 0 | 0 | 0 | 0 | 0 | 0.0249 | 0.008 | | | | | | | | | |
| H5 | 0 | 0 | 0 | 0 | 0 | 0 | 0 | 0 | 0 | 0 | 0 | 0 | 0 | 0 | 0 | 0 | 0 | 0.164 | 0.2866 | | | | | | | | |
| H6 | 0 | 0 | 0 | 0 | 0 | 0 | 0 | 0 | 0 | 0 | 0 | 0 | 0 | 0 | 0 | 0 | 0 | 0 | 0 | 0 | | | | | | | |
| H7 | 0 | 0 | 0 | 0 | 0 | 0 | 0 | 0 | 0 | 0 | 0 | 0 | 0 | 0 | 0 | 0 | 0 | 0 | 0 | 0 | 0.5214 | | | | | | |
| H8 | 0 | 0 | 0 | 0 | 0 | 0 | 0 | 0 | 0 | 0 | 0 | 0 | 0 | 0 | 0 | 0 | 0 | 0 | 0 | 0 | 0 | 0.1678 | | | | | |
| H9 | 0 | 0 | 0 | 0 | 0 | 0 | 0 | 0 | 0 | 0 | 0 | -0.012 | 0 | 0 | 0 | 0 | 0 | 0 | 0 | 0 | 0 | 0 | 0 | | | | |
| H10 | 0 | 0 | 0 | 0 | 0 | 0 | 0 | 0 | 0 | 0 | 0 | 0 | 0 | 0 | 0 | 0 | 0 | 0 | 0 | 0 | 0 | 0 | 0 | 0.1928 | | | |
| H11 | 0 | 0 | 0 | 0 | 0 | -0.0454 | 0 | 0 | 0 | 0 | 0 | 0 | 0 | 0 | 0 | 0 | 0 | 0 | 0 | 0.0839 | 0 | 0 | 0 | 0 | 0 | | |
| H12 | 0 | 0 | 0 | 0 | 0 | 0 | 0 | 0 | 0 | 0 | 0 | 0 | 0 | 0 | 0 | 0 | 0 | 0 | 0 | 0 | 0 | 0 | 0 | 0 | 0 | 0 | |
| H13 | 0 | 0 | 0 | 0 | 0 | 0 | 0 | 0 | 0 | 0 | 0 | 0 | 0 | 0 | 0 | 0 | 0 | 0 | 0 | 0 | 0 | 0 | -0.0305 | 0 | 0 | 0 | 0.0771 |

## (O). Mainland China

| | L1 | L2 | L3 | L4 | L5 | L6 | L7 | L8 | L9 | L10 | L11 | L12 | L13 | L14 | L18 | H1 | H2 | H3 | H4 | H5 | H6 | H7 | H8 | H9 | H10 | H11 | H12 |
|---|---|---|---|---|---|---|---|---|---|---|---|---|---|---|---|---|---|---|---|---|---|---|---|---|---|---|---|
| L1 | | | | | | | | | | | | | | | | | | | | | | | | | | | |
| L2 | 0.5361 | | | | | | | | | | | | | | | | | | | | | | | | | | |
| L3 | -0.0231 | -0.0262 | | | | | | | | | | | | | | | | | | | | | | | | | |
| L4 | -0.0159 | -0.0347 | 0.1932 | | | | | | | | | | | | | | | | | | | | | | | | |
| L5 | 0 | 0 | 0.0895 | 0.4653 | | | | | | | | | | | | | | | | | | | | | | | |
| L6 | 0 | 0.0695 | 0 | 0 | 0.0207 | | | | | | | | | | | | | | | | | | | | | | |
| L7 | 0 | 0.0106 | 0 | 0 | 0 | 0.8325 | | | | | | | | | | | | | | | | | | | | | |
| L8 | 0 | 0 | 0.1537 | 0.1288 | 0.1092 | 0.0239 | 0.0997 | | | | | | | | | | | | | | | | | | | | |
| L9 | -0.0077 | 0 | 0 | 0 | 0 | 0 | 0 | 0 | | | | | | | | | | | | | | | | | | | |
| L10 | 0 | 0.0665 | -0.0567 | 0 | -0.0748 | 0 | 0.014 | 0 | 0.4909 | | | | | | | | | | | | | | | | | | |
| L11 | 0 | 0 | 0 | 0.0549 | 0.0218 | 0 | 0 | 0 | -0.1097 | -0.0694 | | | | | | | | | | | | | | | | | |
| L12 | 0 | 0 | 0 | 0 | 0 | 0.0106 | 0 | 0.0933 | -0.1022 | 0 | 0.7304 | | | | | | | | | | | | | | | | |
| L13 | 0 | 0 | 0.04 | 0.0012 | 0.015 | 0 | 0 | 0 | 0 | 0 | 0 | 0 | | | | | | | | | | | | | | | |
| L14 | 0.0264 | 0 | 0 | 0.0163 | 0 | -0.0635 | -0.0211 | 0 | 0 | 0 | 0.0057 | 0 | 0.6327 | | | | | | | | | | | | | | |
| L18 | 0.0206 | 0.0696 | 0 | 0 | 0.0415 | 0 | 0 | 0 | 0.0041 | 0.0667 | 0 | 0 | 0.1229 | 0.0302 | | | | | | | | | | | | | |
| H1 | 0 | 0 | 0 | 0.014 | 0.0599 | -0.0055 | 0 | 0 | 0 | -0.025 | 0 | 0 | 0 | 0.0175 | 0.1525 | | | | | | | | | | | | |
| H2 | 0.0983 | 0 | 0 | -0.0468 | -0.0051 | 0 | 0 | 0 | 0 | 0 | 0 | 0 | 0 | 0 | 0 | -0.4001 | | | | | | | | | | | |
| H3 | 0 | 0.1121 | 0 | 0.0409 | 0 | 0 | 0 | 0 | 0 | 0 | 0.0072 | 0 | 0.0138 | 0.0006 | 0.0946 | 0 | 0.2265 | | | | | | | | | | |
| H4 | 0 | 0 | 0 | 0 | 0 | 0.004 | 0.0078 | 0 | 0 | 0 | 0 | -0.0004 | 0 | 0 | 0.0245 | 0 | 0.0189 | 0.1679 | | | | | | | | | |
| H5 | 0.041 | 0.0265 | 0 | 0 | 0 | 0 | 0 | 0 | 0 | 0.0051 | 0 | 0 | 0 | 0 | 0.0842 | 0 | 0.0852 | 0.2173 | 0.4522 | | | | | | | | |
| H6 | 0 | 0 | 0 | 0 | 0 | 0 | 0 | 0 | 0 | 0 | 0 | 0 | 0 | 0 | 0 | 0 | 0 | 0 | 0 | 0.0263 | | | | | | | |
| H7 | 0 | 0 | 0 | 0 | 0 | -0.0026 | 0 | 0 | 0 | 0 | 0 | 0 | 0 | 0.0233 | 0 | 0.0365 | 0 | 0 | 0 | 0.0254 | 0.6601 | | | | | | |
| H8 | 0.0299 | 0.0018 | 0 | 0.0191 | 0.0159 | -0.0124 | -0.005 | 0 | -0.0325 | -0.0228 | 0 | 0.0178 | 0 | 0.051 | 0 | 0.0052 | 0 | 0.0412 | 0 | 0 | 0.0704 | 0.1357 | | | | | |
| H9 | 0 | 0 | 0 | 0 | 0 | 0 | 0 | 0 | 0 | 0.053 | 0 | -0.0228 | 0 | 0 | 0 | -0.0307 | 0 | 0 | 0.0045 | 0.0476 | -0.0313 | 0 | -0.0807 | | | | |
| H10 | 0 | 0 | 0 | 0 | 0 | 0 | 0 | 0 | 0.0361 | 0.0647 | -0.0003 | -0.0477 | 0 | 0 | 0 | -0.0331 | 0 | 0 | 0 | 0 | 0 | -0.0071 | -0.0282 | 0.35 | | | |
| H11 | 0.0638 | 0 | 0 | 0 | -0.0233 | -0.0078 | -0.0586 | -0.0198 | 0 | 0 | 0 | 0 | 0.004 | 0.1014 | 0.0605 | 0 | 0 | 0.0259 | 0 | 0.0705 | 0 | 0.0415 | 0 | 0 | 0 | | |
| H12 | -0.0379 | -0.0135 | 0 | 0 | 0.0043 | 0 | 0.0168 | 0.0708 | 0 | -0.0106 | 0.0171 | 0 | 0 | -0.062 | -0.0161 | 0 | 0 | -0.0295 | 0 | -0.029 | 0 | 0 | 0 | -0.0426 | -0.0034 | -0.2453 | |
| H13 | 0 | 0 | 0 | 0 | 0 | 0.0223 | 0 | 0 | 0 | 0 | 0 | 0 | -0.0842 | 0 | 0 | -0.0244 | 0 | 0 | 0 | 0 | -0.1661 | -0.0716 | -0.1262 | 0 | 0 | 0 | 0.1709 |

## (P). Malaysia

| | L1 | L2 | L3 | L4 | L5 | L6 | L7 | L8 | L9 | L10 | L11 | L12 | L13 | L14 | L18 | H1 | H2 | H3 | H4 | H5 | H6 | H7 | H8 | H9 | H10 | H11 | H12 |
|---|---|---|---|---|---|---|---|---|---|---|---|---|---|---|---|---|---|---|---|---|---|---|---|---|---|---|---|
| L1 | | | | | | | | | | | | | | | | | | | | | | | | | | | |
| L2 | 0.2724 | | | | | | | | | | | | | | | | | | | | | | | | | | |
| L3 | -0.0151 | -0.0101 | | | | | | | | | | | | | | | | | | | | | | | | | |
| L4 | -0.0875 | 0 | 0.3157 | | | | | | | | | | | | | | | | | | | | | | | | |
| L5 | -0.0474 | 0 | 0.162 | 0.2855 | | | | | | | | | | | | | | | | | | | | | | | |
| L6 | 0.0106 | 0.0916 | -0.0636 | 0 | 0 | | | | | | | | | | | | | | | | | | | | | | |
| L7 | 0 | 0.07 | -0.0278 | 0 | 0 | 0.5635 | | | | | | | | | | | | | | | | | | | | | |
| L8 | 0 | 0 | 0 | 0.063 | 0.1038 | 0 | 0.0074 | | | | | | | | | | | | | | | | | | | | |
| L9 | 0 | 0 | 0 | -0.0078 | -0.033 | 0 | 0 | -0.0573 | | | | | | | | | | | | | | | | | | | |
| L10 | 0 | 0 | 0 | 0 | -0.0199 | 0 | 0 | -0.0568 | 0.4935 | | | | | | | | | | | | | | | | | | |
| L11 | 0 | 0 | 0 | 0 | 0.0509 | 0 | 0 | 0.0251 | -0.0869 | -0.0416 | | | | | | | | | | | | | | | | | |
| L12 | 0 | 0 | 0 | 0 | 0.0546 | 0 | 0 | 0 | -0.0826 | -0.0302 | 0.7162 | | | | | | | | | | | | | | | | |
| L13 | 0 | -0.0094 | 0 | 0.0275 | 0 | -0.0226 | -0.1061 | 0 | 0 | 0 | 0 | 0 | | | | | | | | | | | | | | | |
| L14 | -0.0122 | 0 | 0.0114 | 0.0415 | 0 | 0 | -0.0576 | 0 | 0 | 0 | 0 | 0 | 0.5473 | | | | | | | | | | | | | | |
| L18 | 0 | 0.0761 | 0 | 0 | 0 | 0 | 0 | 0 | 0.0076 | 0 | 0 | 0 | 0 | 0 | | | | | | | | | | | | | |
| H1 | 0 | 0 | 0 | 0 | 0.01 | 0 | 0 | 0 | 0 | -0.0232 | 0 | 0 | 0.0115 | 0.0563 | 0 | | | | | | | | | | | | |
| H2 | 0.1182 | 0 | -0.0444 | -0.0374 | -0.0082 | 0.0057 | 0.0493 | 0 | 0 | 0 | 0 | 0 | -0.071 | -0.0262 | 0 | -0.3973 | | | | | | | | | | | |
| H3 | 0 | 0.0538 | 0 | 0 | 0 | 0.0062 | 0 | 0 | 0 | 0 | 0 | 0 | 0 | 0 | 0.1649 | -0.0077 | 0.1556 | | | | | | | | | | |
| H4 | 0 | 0.0366 | 0 | 0 | 0 | 0 | 0 | 0 | 0 | 0 | 0 | 0 | 0 | 0 | 0.0012 | 0 | 0.0667 | 0.1343 | | | | | | | | | |
| H5 | 0 | 0.0177 | 0 | 0 | 0 | 0 | 0 | 0 | 0 | 0 | 0 | 0 | 0 | 0 | 0.1537 | 0 | 0 | 0.1053 | 0.3739 | | | | | | | | |
| H6 | 0 | 0 | 0 | 0 | 0 | 0 | 0 | 0.0365 | -0.0035 | 0 | 0 | 0 | 0.017 | 0 | 0 | 0.035 | 0 | -0.0104 | 0 | 0 | | | | | | | |
| H7 | 0 | 0 | 0 | 0 | 0 | 0 | -0.0125 | 0 | 0 | -0.0076 | 0 | 0 | 0 | 8.00E-04 | 0 | 0 | 0 | 0 | 0 | 0 | 0.5955 | | | | | | |
| H8 | 0 | 0 | 0 | 0 | 0 | 0 | -0.0373 | 0 | -0.0272 | -0.0499 | 0 | 0 | 0 | 0 | -0.0298 | 0.0029 | 0 | 0 | 0 | 0 | 0.0291 | 0.166 | | | | | |
| H9 | 0 | 0.0328 | 0 | 0 | 0 | 0 | 0 | 0 | 0 | 0 | 0 | 0 | 0 | 0 | 0.0632 | -0.0041 | 0.0078 | 0.0446 | 0 | 0.0654 | 0 | -0.0018 | 0 | | | | |
| H10 | 0 | 0 | 0 | 0 | 0 | 0 | 0.0153 | 0 | 0 | 0 | 0 | 0 | -0.0065 | -0.0096 | 0 | 0 | 0 | 0.0545 | 0 | 0.0198 | 0 | 0 | 0 | 0.5439 | | | |
| H11 | 0 | 0 | 0 | 0 | 0 | 0 | 0 | -0.0153 | 0 | 0 | 0 | 0 | 0 | 0 | 0.0186 | 0 | 0 | 0.0609 | 0 | 0.0823 | 0 | 0 | 0 | 0.1254 | 0.1754 | | |
| H12 | 0 | 0 | 0 | 0 | 0 | 0 | 0 | 0 | 0 | -0.0584 | 0.0192 | 0 | 0 | 0 | 0 | 0 | 0 | 0 | -0.0817 | -0.0045 | 0 | 0 | 0 | 0 | 0 | -0.1245 | |
| H13 | 0 | 0 | 0 | 0 | 0 | 0 | 0 | -0.056 | 0.0167 | 0 | 0 | 0 | 0 | 0 | 0 | 0 | 0.0338 | 0 | 0 | 0.0129 | -0.1539 | 0 | -0.0971 | 0 | 0.0506 | 0.0443 | -0.1305 |

## (Q). Mexico

| | L1 | L2 | L3 | L4 | L5 | L6 | L7 | L8 | L9 | L10 | L11 | L12 | L13 | L14 | L18 | H1 | H2 | H3 | H4 | H5 | H6 | H7 | H8 | H9 | H10 | H11 | H12 |
|---|---|---|---|---|---|---|---|---|---|---|---|---|---|---|---|---|---|---|---|---|---|---|---|---|---|---|---|
| L1 | | | | | | | | | | | | | | | | | | | | | | | | | | | |
| L2 | 0.1735 | | | | | | | | | | | | | | | | | | | | | | | | | | |
| L3 | -0.0192 | 0 | | | | | | | | | | | | | | | | | | | | | | | | | |
| L4 | -0.0467 | 0.0217 | 0.2384 | | | | | | | | | | | | | | | | | | | | | | | | |
| L5 | 0 | 0.0918 | 0.0357 | 0.4738 | | | | | | | | | | | | | | | | | | | | | | | |
| L6 | 0.005 | 0.0613 | 0 | 0 | 0 | | | | | | | | | | | | | | | | | | | | | | |
| L7 | 0 | 0.1186 | 0 | 0 | 0 | 0.6208 | | | | | | | | | | | | | | | | | | | | | |
| L8 | 0 | 0 | 0.1141 | 0.104 | 0.0848 | 0 | 0.0294 | | | | | | | | | | | | | | | | | | | | |
| L9 | 0 | 0 | 0 | 0 | 0 | 0 | 0 | -0.0402 | | | | | | | | | | | | | | | | | | | |
| L10 | 0 | 0 | -0.0439 | 0 | 0 | 0 | 0 | 0 | 0.3893 | | | | | | | | | | | | | | | | | | |
| L11 | 0 | 0.0262 | 0 | 0 | 0.0624 | 0.0584 | 0.0001 | 0.0196 | -0.0008 | 0 | | | | | | | | | | | | | | | | | |
| L12 | 0 | 0 | 0 | 0.044 | 0.0714 | 0.0019 | 0 | 0.0637 | 0 | 0 | 0.5189 | | | | | | | | | | | | | | | | |
| L13 | 0 | 0 | 0 | 0 | 0 | -0.025 | 0 | 0 | 0 | 0 | 0 | 0 | | | | | | | | | | | | | | | |
| L14 | -0.0309 | 0 | 0.0394 | 0.0136 | 0.0028 | -0.0486 | -0.0101 | 0 | 0 | 0 | 0 | 0.0088 | 0.6439 | | | | | | | | | | | | | | |
| L18 | 0 | 0.052 | 0 | 0 | 0.0173 | 0 | 0 | 0 | 0 | 0 | 0 | 0 | 0.0759 | 0.0285 | | | | | | | | | | | | | |
| H1 | -0.129 | 0 | 0 | 0.0682 | 0.0634 | 0 | 0 | 0 | 0 | 0 | 0 | 0 | 0 | 0.0515 | 0.0335 | | | | | | | | | | | | |
| H2 | 0.1091 | 0 | 0 | -0.1335 | 0 | 0 | 0 | 0 | 0 | 0 | 0 | 0 | -0.0381 | 0 | 0 | -0.3551 | | | | | | | | | | | |
| H3 | 0 | 0.0901 | 0 | 0 | 0 | 0 | 0 | 0 | 0 | 0 | 0 | 0 | 0 | 0 | 0.3384 | 0.015 | 0 | | | | | | | | | | |
| H4 | 0 | 0 | 0 | 0 | 0 | 0 | 0 | 0 | -4.00E-04 | 0 | 0 | 0 | 0 | 0 | 0 | 0 | 0 | 0.029 | | | | | | | | | |
| H5 | 0 | 0.1077 | 0 | 0 | 0 | 0 | 0 | 0 | 0 | 0 | 0 | 0 | 0.0161 | 0 | 0.0525 | 0 | 0 | 0.2462 | 0.3493 | | | | | | | | |
| H6 | -0.0591 | 0 | 0 | 0 | 0 | 0 | 0 | 0 | 0 | 0 | 0 | 0 | 0 | 0.028 | 0 | 0 | 0 | 0 | 0 | 0 | | | | | | | |
| H7 | 0 | 0 | 0 | 0.0132 | 0 | 0 | 0 | 0 | 0 | -0.0482 | 0 | 0 | 0.0037 | 0.0721 | 0.0017 | 0 | -0.0035 | 0 | 0.0393 | 0 | 0.546 | | | | | | |
| H8 | 0 | 0 | 0 | 0 | 0.0128 | 0 | 0 | 0 | 0 | 0 | 0 | 0 | 0.052 | 0 | 0 | 0.0396 | -0.0834 | 0 | 0 | 0.039 | 0 | 0.1669 | | | | | |
| H9 | 0 | 0.04 | 0 | 0 | 0 | 0 | 0 | 0 | 0 | 0 | 0 | 0 | 0 | 0 | 0.0418 | 0 | 0 | 0 | 0 | 0.0368 | -0.0291 | -0.0192 | 0 | | | | |
| H10 | 0 | 0 | 0 | 0 | 0 | 0 | 0 | -0.0273 | 0 | 0.0228 | 0 | 0 | 0 | 0 | 0 | 0 | 0 | 0 | 0 | 0.065 | 0 | 0 | 0 | 0.3107 | | | |
| H11 | 0 | 0 | 0 | 0 | -0.0205 | -0.0132 | -0.0094 | 0 | 0 | 0 | 0 | -0.0033 | 0 | 0.0075 | 0.0254 | 0 | 0 | 0.0036 | 0.1101 | 0.1203 | 0 | 0 | 0 | 0 | 0.1142 | | |
| H12 | 0 | 0 | 0 | 0 | 0.0056 | 0 | 0 | 0.0329 | 0 | 0 | 0 | 0 | 0 | -0.0163 | 0 | 0 | 0 | 0 | 0 | -0.1193 | 0 | 0 | 0 | 0 | -0.0964 | -0.1204 | |
| H13 | 0 | 0 | 0 | -0.0449 | 0 | 0 | 0 | 0 | 0 | 0 | 0 | 0 | 0 | 0 | 0 | 0 | 0 | 0 | 0 | 0 | -0.0309 | -0.0473 | -0.0593 | 0 | 0 | 0 | 0 |

## (R). Nigeria

| | L1 | L2 | L3 | L4 | L5 | L6 | L7 | L8 | L9 | L10 | L11 | L12 | L13 | L14 | L18 | H1 | H2 | H3 | H4 | H5 | H6 | H7 | H8 | H9 | H10 | H11 | H12 |
|---|---|---|---|---|---|---|---|---|---|---|---|---|---|---|---|---|---|---|---|---|---|---|---|---|---|---|---|
| L1 | | | | | | | | | | | | | | | | | | | | | | | | | | | |
| L2 | 0.257 | | | | | | | | | | | | | | | | | | | | | | | | | | |
| L3 | -0.1328 | 0 | | | | | | | | | | | | | | | | | | | | | | | | | |
| L4 | 0 | 0 | 0.1919 | | | | | | | | | | | | | | | | | | | | | | | | |
| L5 | -0.0326 | 0 | 0.1557 | 0.3123 | | | | | | | | | | | | | | | | | | | | | | | |
| L6 | 0.059 | 0.086 | 0 | 0 | 0 | | | | | | | | | | | | | | | | | | | | | | |
| L7 | 0 | 0.0934 | 0 | 0 | 0 | 0.5801 | | | | | | | | | | | | | | | | | | | | | |
| L8 | 0 | 0 | 0.0817 | 0.107 | 0.1033 | 0.0085 | 0.1021 | | | | | | | | | | | | | | | | | | | | |
| L9 | 0 | 0 | -0.0263 | -0.003 | -0.0809 | 0 | 0 | -0.0521 | | | | | | | | | | | | | | | | | | | |
| L10 | 0.0513 | 0.0537 | 0 | 0 | 0 | 0 | 0 | -0.0262 | 0.2524 | | | | | | | | | | | | | | | | | | |
| L11 | 0 | 0 | 0.0463 | 0 | 0 | 0.0162 | 0 | 0.0615 | -0.05 | -0.0792 | | | | | | | | | | | | | | | | | |
| L12 | 0 | 0 | 0.0152 | 0 | 0 | 0.0058 | 0.0089 | 0 | -0.1061 | 0 | 0.6467 | | | | | | | | | | | | | | | | |
| L13 | 0 | 0 | 0.0102 | 0 | 0.0081 | 0 | -0.018 | 0 | 0 | 0 | 0.0789 | 0.0146 | | | | | | | | | | | | | | | |
| L14 | 0 | 0 | 0.0488 | 0 | 0.0147 | 0 | 0 | 0 | -0.0071 | 0 | 0 | 0.0027 | 0.4134 | | | | | | | | | | | | | | |
| L18 | 0.0142 | 0.053 | 0 | 0 | 0 | 0 | 0.0153 | 0 | 0 | 0 | 0 | 0 | 0 | 0 | | | | | | | | | | | | | |
| H1 | -0.025 | 0 | 0 | 0 | 0.0207 | 0 | -0.0062 | 0 | -0.0388 | 0 | 0 | 0 | 0.0348 | 0.0841 | 0 | | | | | | | | | | | | |
| H2 | 0 | 0 | 0 | -0.0072 | 0 | 0 | 0.0422 | 0 | 0 | 0 | 0 | 0 | -0.0193 | -0.0073 | 0.0082 | -0.233 | | | | | | | | | | | |
| H3 | 0 | 0.0263 | -0.02 | -0.0444 | 0 | 0.055 | 0.0517 | 0 | 0 | 0 | 0 | 0 | 0 | 0 | 0.1658 | 0 | 0.1864 | | | | | | | | | | |
| H4 | 0 | 0.0045 | 0 | 0 | 0 | 0 | 0.0596 | 0 | 0 | 0 | 0 | 0 | -0.0929 | -0.1002 | 0 | -0.0152 | 0.1903 | 0.0477 | | | | | | | | | |
| H5 | 0.0706 | 0.0834 | 0 | 0 | 0 | 0.0293 | 0.0655 | 0 | 0 | 0 | 0 | 0 | 0 | 0 | 0.0163 | -0.0778 | 0 | 0.1308 | 0.2388 | | | | | | | | |
| H6 | 0 | 0 | 0 | 0 | 0 | 0 | 0 | 0 | 0 | -0.0178 | 0 | 0 | 6.00E-04 | 0.0551 | 0 | 0.0618 | 0 | 0 | 0 | 0.0492 | | | | | | | |
| H7 | 0 | 0 | 0 | 0 | 0 | 0 | 0 | 0 | 0 | -0.0059 | 0 | 0 | 0 | 0.0578 | 0 | 0 | 0 | 0 | 0 | 0.0478 | 0.4995 | | | | | | |
| H8 | 0 | 0 | 0 | 0 | 0 | 0.0121 | 0 | 0.0605 | -0.1034 | -0.0265 | 0.0233 | 0.0182 | 0.0047 | 0.0372 | 0 | 0 | 0 | 0 | 0 | 0.0348 | 0.1392 | 0.2426 | | | | | |
| H9 | 0 | 0.0131 | 0 | 0 | 0 | 0 | 0 | 0 | 0 | 0.0397 | 0 | -0.0393 | 0 | 0 | 0 | -0.0205 | 0.0224 | 0 | 0 | 0.0283 | 0 | 0 | -0.0475 | | | | |
| H10 | 0 | 0.0585 | 0 | 0 | 0 | 0.0185 | 0 | 0 | 0 | 0.0393 | -0.0767 | 0 | 0 | 0 | 0 | 0 | 0 | 0.0075 | 0.0241 | 0.0468 | 0 | 0 | 0 | 0.4164 | | | |
| H11 | 0.0122 | 0.0194 | 0 | -0.0565 | 0 | 0 | 0 | -0.0519 | 0 | 0 | 0 | 0 | 0 | 0 | 0.0192 | 0 | 0 | 0 | 0 | 0.0087 | 0 | 0 | 0 | 0.1385 | 0.1405 | | |
| H12 | -0.0633 | 0 | 0 | 0 | 0 | 0 | 0 | 0.0445 | 0 | -0.0193 | 8.00E-04 | 0.0688 | 0 | 0 | -0.036 | 0 | 0 | 0 | 0 | -0.0875 | 0 | 0 | 0 | -0.0635 | -0.1548 | -0.1275 | |
| H13 | 0 | 0 | 0 | 0 | 0 | 0 | 0.0324 | 0 | 0 | 0.0026 | 0 | 0 | 0 | 0 | 0 | 0 | 0.0265 | 0 | 0 | 0 | -0.1149 | -0.0267 | -0.0603 | 0.0535 | 0 | 0 | 0 |

## (S). Philippines

| | L1 | L2 | L3 | L4 | L5 | L6 | L7 | L8 | L9 | L10 | L11 | L12 | L13 | L14 | L18 | H1 | H2 | H3 | H4 | H5 | H6 | H7 | H8 | H9 | H10 | H11 | H12 |
|---|---|---|---|---|---|---|---|---|---|---|---|---|---|---|---|---|---|---|---|---|---|---|---|---|---|---|---|
| L1 | | | | | | | | | | | | | | | | | | | | | | | | | | | |
| L2 | 0.2275 | | | | | | | | | | | | | | | | | | | | | | | | | | |
| L3 | -0.0445 | 0 | | | | | | | | | | | | | | | | | | | | | | | | | |
| L4 | -0.187 | 0 | 0.3074 | | | | | | | | | | | | | | | | | | | | | | | | |
| L5 | 0 | 0 | 0.0769 | 0.249 | | | | | | | | | | | | | | | | | | | | | | | |
| L6 | 0.0323 | 0.0606 | -0.0074 | -0.0292 | 0 | | | | | | | | | | | | | | | | | | | | | | |
| L7 | 0 | 0.0876 | -0.0082 | 0 | 0 | 0.7079 | | | | | | | | | | | | | | | | | | | | | |
| L8 | -0.0375 | 0 | 0.0616 | 0 | 0.2123 | 0 | 0 | | | | | | | | | | | | | | | | | | | | |
| L9 | 0.0105 | 0.0621 | -0.0213 | -0.0025 | -0.0314 | 0 | 0 | -0.154 | | | | | | | | | | | | | | | | | | | |
| L10 | 0 | 0.0364 | -0.0557 | 0 | -0.0309 | 0 | 0.0054 | 0 | 0.3788 | | | | | | | | | | | | | | | | | | |
| L11 | 0 | 0 | 0.0235 | 0 | 0 | 0 | 0 | 0 | -0.0402 | -0.0297 | | | | | | | | | | | | | | | | | |
| L12 | 0 | 0 | 0 | 0 | 0.0441 | 0.0079 | 0 | 0.0427 | -0.0238 | 0 | 0.6246 | | | | | | | | | | | | | | | | |
| L13 | 0 | 0 | 0 | 0 | 0 | -0.0516 | 0 | 0 | 0 | -0.0162 | 0 | 0 | | | | | | | | | | | | | | | |
| L14 | 0 | 0 | 0.101 | 0.0403 | 0 | -0.025 | -0.0344 | 0 | 0 | -0.0451 | 0 | 0 | 0.5542 | | | | | | | | | | | | | | |
| L18 | 0 | 0.0287 | 0 | 0.0238 | 0 | 0 | 0 | -0.0099 | 0 | 0 | 0 | -0.038 | 0 | 0 | | | | | | | | | | | | | |
| H1 | -0.0243 | 0 | 0 | 0.0204 | 0.0578 | 0 | 0 | 0.0443 | 0 | 0 | 0 | 0 | 0 | 0.0522 | 0 | | | | | | | | | | | | |
| H2 | 0.0785 | 0.0345 | 0 | -0.1198 | 0 | 0.0236 | 8.00E-04 | 0 | 0 | 0.0262 | 0 | 0 | 0 | -0.0544 | 0 | -0.3732 | | | | | | | | | | | |
| H3 | 0.0227 | 0.0729 | 0 | 0 | 0 | 0.0154 | 0.0159 | 0 | 0 | 0 | 0 | 0 | 0 | 0 | 0.1993 | -0.0532 | 0.2231 | | | | | | | | | | |
| H4 | 0 | 0 | 0 | 0 | 0 | 0 | 0 | 0 | 0 | 0 | 0 | 0 | 0 | 0 | 0.0287 | -0.0547 | 0 | 0.1018 | | | | | | | | | |
| H5 | 0 | 0.0039 | 0 | 0 | 0 | 0.0049 | 0.0298 | 0 | 0.0132 | 0 | 0 | 0 | 0 | 0 | 0.1239 | 0 | 0 | 0.1969 | 0.307 | | | | | | | | |
| H6 | 0 | 0 | 0 | 0.0086 | 0 | 0 | 0 | 0 | 0 | 0 | 0.027 | 0 | 0 | 0.0532 | 0 | 0.0248 | -0.0092 | -0.0026 | -0.0054 | 0 | | | | | | | |
| H7 | 0 | 0 | 0 | 0 | 0 | 0 | -0.0173 | 0 | 0 | -0.0061 | 0 | 0 | 0.0082 | 0.034 | 0 | 0 | 0 | 0 | 0 | 0 | 0.692 | | | | | | |
| H8 | 0 | 0 | 0 | 0.0068 | 0 | 0 | 0 | 0 | 0 | 0 | 0 | 0.0107 | 0.0425 | 0 | 0 | 0.0439 | -0.0424 | 0 | 0 | 0 | 0.076 | 0.1513 | | | | | |
| H9 | 0.0031 | 0.0185 | 0 | 0 | 0 | 0 | 0 | 0 | 0 | 0 | 0 | 0 | 0 | 0 | 0 | 0 | 0 | 0.0036 | 0.0017 | 0.1048 | 0 | 0 | -0.124 | | | | |
| H10 | 0.0287 | 0 | 0 | 0 | 0 | 0 | 0 | 0 | 0 | 0 | 0 | 0 | 0 | 0 | 0 | 0 | 0.1033 | 0 | 0.0285 | 0.0541 | 0 | 0 | 0 | 0.539 | | | |
| H11 | 0.1039 | 0 | 0 | 0 | -0.075 | 0 | -0.0213 | -0.0668 | 0 | 0 | 0 | 0 | 0 | 0 | 0.0604 | 0 | 0 | 0.0189 | 0 | 0.0103 | 0 | 0 | -0.0163 | 0.0426 | 0.2139 | | |
| H12 | 0 | 0 | 0 | 0 | 0.0114 | 0 | 0 | 0.105 | 0 | -0.0424 | 0.0269 | 0.0437 | 0 | 0 | -0.0436 | 0.0343 | 0 | 0 | 0 | -0.1535 | 0 | 0 | 0 | 0 | -0.0419 | -0.1806 | |
| H13 | 0.0161 | 0 | -0.0042 | 0 | 0 | 0 | 0.0015 | 0 | 0.0266 | 0.0559 | -0.0079 | 0 | 0 | -0.0317 | 0 | 0 | 0 | 0 | 0 | 0 | -0.0798 | -0.0667 | -0.0765 | 0 | 0.0285 | 0 | -0.2242 |

## (T). Republic of Sudan

| | L1 | L2 | L3 | L4 | L5 | L6 | L7 | L8 | L9 | L10 | L11 | L12 | L13 | L14 | L18 | H1 | H2 | H3 | H4 | H5 | H6 | H7 | H8 | H9 | H10 | H11 | H12 |
|---|---|---|---|---|---|---|---|---|---|---|---|---|---|---|---|---|---|---|---|---|---|---|---|---|---|---|---|
| L1 | | | | | | | | | | | | | | | | | | | | | | | | | | | |
| L2 | 0.1828 | | | | | | | | | | | | | | | | | | | | | | | | | | |
| L3 | -0.0575 | 0 | | | | | | | | | | | | | | | | | | | | | | | | | |
| L4 | -0.0659 | 0 | 0.1842 | | | | | | | | | | | | | | | | | | | | | | | | |
| L5 | 0 | 0 | 0.1515 | 0.2129 | | | | | | | | | | | | | | | | | | | | | | | |
| L6 | 0 | 0.076 | 0 | 0 | 0 | | | | | | | | | | | | | | | | | | | | | | |
| L7 | 0.0648 | 0.094 | 0 | 0 | 0 | 0.5869 | | | | | | | | | | | | | | | | | | | | | |
| L8 | 0 | 0 | 0 | 0.077 | 0.2571 | 0.0032 | 0.1051 | | | | | | | | | | | | | | | | | | | | |
| L9 | 0 | 0.0575 | 0 | 0 | 0 | 0 | 0.0472 | 0 | | | | | | | | | | | | | | | | | | | |
| L10 | 0 | 0.0767 | -0.0241 | 0 | 0 | 0.0355 | 0 | 0 | 0.2647 | | | | | | | | | | | | | | | | | | |
| L11 | 0 | 0 | 0 | 0 | 0.0401 | 0 | 0.0103 | 0.0527 | 0 | 0 | | | | | | | | | | | | | | | | | |
| L12 | 0 | 0 | 0.0181 | 0 | 0 | 0 | 0.0126 | 0.0316 | 0 | -0.1226 | 0.5917 | | | | | | | | | | | | | | | | |
| L13 | 0 | 0 | 0 | 0.0047 | 0 | 0 | 0 | 0 | 0 | 0 | 0 | 0 | | | | | | | | | | | | | | | |
| L14 | 0 | 0 | 0.0647 | 0 | 0 | -0.0666 | -0.0853 | 0 | 0 | -0.0345 | 0 | 0 | 0.2769 | | | | | | | | | | | | | | |
| L18 | 0 | 0.0447 | 0 | 0 | 0 | 0 | 0 | 0 | 0 | 0.0324 | -0.0257 | -0.0773 | 0 | 0 | | | | | | | | | | | | | |
| H1 | 0 | 0 | 0 | 0 | 0 | -0.0204 | 0 | 0 | 0 | 0 | 0 | 0 | 0.0671 | 0 | 0 | | | | | | | | | | | | |
| H2 | 0.1095 | 0.0778 | 0 | 0 | 0 | 0 | 0.0028 | 0 | 0 | 0 | 0 | 0 | 0 | -0.0307 | 0 | -0.3505 | | | | | | | | | | | |
| H3 | 0 | 0.0451 | 0 | 0 | 0 | 0.0088 | 0 | 0 | 0 | 0 | 0 | 0 | 0 | 0 | 0.0711 | -0.0926 | 0.1937 | | | | | | | | | | |
| H4 | 0 | 0 | 0 | 0 | 0 | 0 | 0 | 0 | 0 | 0 | 0 | 0 | 0 | 0 | 0 | -0.042 | 0.0949 | 0.2346 | | | | | | | | | |
| H5 | 0.045 | 0.0284 | 0 | 0 | 0 | 0 | 0 | 0 | 0 | 0 | 0 | 0 | 0 | 0 | 0 | 0 | 0 | 0.1543 | 0.1907 | | | | | | | | |
| H6 | -0.0222 | 0 | 0 | 0 | 0.0082 | 0 | 0 | 0 | 0 | 0 | 0 | 0 | 0.0493 | 0.0125 | 0 | 0 | -0.0351 | 0 | 0 | 0 | | | | | | | |
| H7 | 0 | 0 | 0 | 0.0139 | 0.0123 | 0 | 0 | 0 | 0 | 0 | 0 | 0 | 0 | 0.0329 | 0 | 0 | 0 | 0 | 0 | 0 | 0.3924 | | | | | | |
| H8 | 0 | 0 | 0 | 0 | 0 | 0 | 0 | 0 | 0 | -0.0166 | 0 | 0 | 0.0094 | 0.1086 | 0 | 0 | 0 | 0 | 0 | 0 | 0.0564 | 0.2171 | | | | | |
| H9 | 0 | 0.0406 | 0 | 0 | 0 | 0.0231 | 0 | 0 | 0.0149 | 0 | 0 | 0 | 0 | 0 | 0 | 0 | 0 | 0.027 | 0 | 0 | -0.0354 | 0 | 0 | | | | |
| H10 | 0 | 0 | 0 | 0 | 0 | 0 | 0 | 0 | 0.0071 | 0.0141 | 0 | 0 | 0 | 0 | 0 | 0 | 0 | 0 | 0 | 0 | 0 | 0 | 0 | 0.17 | | | |
| H11 | 0 | 0 | 0 | 0 | -0.0081 | 0 | 0 | 0 | 0 | 0 | 0 | 0 | 0 | 0 | 0.0026 | 0 | 0 | 0 | 0 | 0.1097 | 0 | 0.0152 | 0 | 0.1088 | 0.1089 | | |
| H12 | -0.0321 | -0.0072 | 0 | 0 | 0 | 0 | 0 | 0.0051 | 0 | 0 | 0 | 0.013 | 0 | 0 | -0.0328 | 0 | 0 | -0.0089 | 0 | -0.1666 | 0 | 0 | 0 | -0.0277 | 0 | -0.0641 | |
| H13 | 0 | 0 | 0 | 0 | 0 | 0 | 0 | 0 | 0.0236 | 0 | 0 | -0.0022 | 0 | 0 | 0 | -0.0044 | 0 | 0 | 0 | 0 | -0.1183 | 0 | -0.0304 | 0.0592 | 0 | 0 | -0.0876 |

## (U). Rwanda

| | L1 | L2 | L3 | L4 | L5 | L6 | L7 | L8 | L9 | L10 | L11 | L12 | L13 | L14 | L18 | H1 | H2 | H3 | H4 | H5 | H6 | H7 | H8 | H9 | H10 | H11 | H12 |
|---|---|---|---|---|---|---|---|---|---|---|---|---|---|---|---|---|---|---|---|---|---|---|---|---|---|---|---|
| L1 | | | | | | | | | | | | | | | | | | | | | | | | | | | |
| L2 | 0.3625 | | | | | | | | | | | | | | | | | | | | | | | | | | |
| L3 | 0 | 0 | | | | | | | | | | | | | | | | | | | | | | | | | |
| L4 | 0 | 0 | 0.0096 | | | | | | | | | | | | | | | | | | | | | | | | |
| L5 | -0.0142 | 0 | 0.0731 | 0.1951 | | | | | | | | | | | | | | | | | | | | | | | |
| L6 | 0 | 0 | 0 | 0 | 0 | | | | | | | | | | | | | | | | | | | | | | |
| L7 | 0 | 0 | 0 | 0 | 0 | 0.3221 | | | | | | | | | | | | | | | | | | | | | |
| L8 | 0 | 0 | 0 | 0 | 0 | 0 | 0 | | | | | | | | | | | | | | | | | | | | |
| L9 | 0 | 0 | 0 | 0 | 0 | 0 | 0 | 0 | | | | | | | | | | | | | | | | | | | |
| L10 | 0 | 0 | 0 | -0.0355 | 0 | 0 | 0 | 0 | 0.1606 | | | | | | | | | | | | | | | | | | |
| L11 | 0 | 0 | 0 | 0 | 0 | 0 | 0 | 0.0566 | -0.0588 | -0.1507 | | | | | | | | | | | | | | | | | |
| L12 | 0 | 0 | 0 | 0 | 0 | 0 | 0 | 0 | 0 | 0 | 0.1957 | | | | | | | | | | | | | | | | |
| L13 | 0 | 0 | 0 | 0 | 0 | 0 | 0 | 0 | 0 | 0 | 0 | 0 | | | | | | | | | | | | | | | |
| L14 | 0 | 0 | 0 | 0 | 0 | 0 | 0 | 0 | 0 | 0 | 0 | 0 | 0.3695 | | | | | | | | | | | | | | |
| L18 | 0 | 0 | 0 | 0 | 0 | 0 | 0 | 0 | 0 | 0 | 0 | 0 | 0 | 0 | | | | | | | | | | | | | |
| H1 | 0 | 0 | 0 | 0 | 0 | 0 | 0 | 0 | 0 | 0 | 0 | 0 | 0.0311 | 0.0023 | 0 | | | | | | | | | | | | |
| H2 | 0 | 0 | 0 | 0 | 0 | 0 | 0 | 0 | 0 | 0 | 0 | 0 | 0 | 0 | 0 | -0.1346 | | | | | | | | | | | |
| H3 | 0 | 0 | 0 | 0 | 0 | 0 | 0 | 0 | 0 | 0 | 0 | 0 | 0 | 0 | 0 | 0 | 0 | | | | | | | | | | |
| H4 | 0 | 0 | 0 | 0 | 0 | 0 | 0 | 0 | 0 | 0 | 0 | 0 | 0 | 0 | 0 | 0 | 0 | 0 | | | | | | | | | |
| H5 | 0.053 | 0 | 0 | 0 | 0 | 0 | 0 | 0 | 0 | 0 | 0 | 0 | 0 | 0 | 0 | 0 | 0 | 0.111 | 0.0936 | | | | | | | | |
| H6 | 0 | 0 | 0 | 0 | 0 | 0 | 0 | 0 | 0 | 0 | 0 | 0 | 0 | 0 | 0 | 0 | 0 | 0 | 0 | 0 | | | | | | | |
| H7 | 0 | 0 | 0 | 0 | 0 | 0 | 0 | 0 | 0 | 0 | 0 | 0 | 0 | 0 | 0 | 0 | 0 | 0 | 0 | 0 | 0.3438 | | | | | | |
| H8 | 0 | 0 | 0 | 0 | 0 | 0 | 0 | 0 | 0 | 0 | 0 | 0 | 0 | 0 | 0 | 0 | 0 | 0 | 0 | 0 | 0 | 0 | | | | | |
| H9 | 0 | 0 | 0 | 0 | 0 | 0 | 0 | 0 | 0 | 0 | 0 | 0 | 0 | 0 | 0 | 0 | 0 | 0 | 0 | 0 | 0 | 0 | 0 | | | | |
| H10 | 0 | 0 | 0 | 0 | 0 | 0 | 0 | 0 | 0 | 0 | 0 | 0 | 0 | 0 | 0 | 0 | 0 | 0 | 0 | 0 | 0 | 0 | 0 | 0 | | | |
| H11 | 0 | 0 | 0 | 0 | 0 | 0 | 0 | 0 | 0 | 0 | 0 | 0 | 0 | 0 | 0 | 0 | 0 | 0 | 0 | 0 | 0 | 0 | 0 | 0 | 0 | | |
| H12 | 0 | 0 | 0 | 0 | 0 | 0 | 0 | 0 | 0 | 0 | 0 | 0 | 0 | 0 | 0 | 0 | 0 | 0 | 0 | -0.0516 | 0 | 0 | 0 | 0 | 0 | -0.1101 | |
| H13 | 0 | 0 | 0 | 0 | 0 | 0 | 0 | 0 | 0 | 0 | 0 | 0 | 0 | 0 | 0 | 0 | 0 | 0 | 0 | 0 | 0 | 0 | 0 | 0 | 0 | 0 | 0 |

## (V). Saudi Arabia

| | L1 | L2 | L3 | L4 | L5 | L6 | L7 | L8 | L9 | L10 | L11 | L12 | L13 | L14 | L18 | H1 | H2 | H3 | H4 | H5 | H6 | H7 | H8 | H9 | H10 | H11 | H12 |
|---|---|---|---|---|---|---|---|---|---|---|---|---|---|---|---|---|---|---|---|---|---|---|---|---|---|---|---|
| L1 | | | | | | | | | | | | | | | | | | | | | | | | | | | |
| L2 | 0.1829 | | | | | | | | | | | | | | | | | | | | | | | | | | |
| L3 | -0.1583 | 0 | | | | | | | | | | | | | | | | | | | | | | | | | |
| L4 | -0.1023 | 0 | 0.2399 | | | | | | | | | | | | | | | | | | | | | | | | |
| L5 | 0 | 0.008 | 0.1761 | 0.2629 | | | | | | | | | | | | | | | | | | | | | | | |
| L6 | 0.0305 | 0.0897 | -0.0076 | 0 | 0 | | | | | | | | | | | | | | | | | | | | | | |
| L7 | 0 | 0.0835 | 0 | 0 | 0 | 0.624 | | | | | | | | | | | | | | | | | | | | | |
| L8 | 0 | 0.0181 | 0.0236 | 0.122 | 0.1345 | 0 | 0.0395 | | | | | | | | | | | | | | | | | | | | |
| L9 | 0 | 0.0439 | 0 | 0 | 0 | 0 | 0.0088 | 0 | | | | | | | | | | | | | | | | | | | |
| L10 | 0 | 0 | 0 | -0.0237 | 0 | 0 | 0 | -0.0785 | 0.2979 | | | | | | | | | | | | | | | | | | |
| L11 | 0 | 0 | 0 | 0 | 0.1508 | 0 | 0.0725 | 0 | 0 | -0.0178 | | | | | | | | | | | | | | | | | |
| L12 | 0 | 0 | 0 | 0 | 0 | 0 | 0 | 0 | 0 | 0 | 0.0084 | | | | | | | | | | | | | | | | |
| L13 | -0.0192 | 0 | 0 | 0.0331 | 0.0201 | -0.0973 | 0 | 0 | -0.0347 | -0.0301 | 0 | 0 | | | | | | | | | | | | | | | |
| L14 | -0.0357 | 0 | 0.1061 | 0 | 0 | 0 | -0.0578 | 0.0368 | -0.0036 | -0.0499 | 0 | 0 | 0.5343 | | | | | | | | | | | | | | |
| L18 | 0 | 0.1158 | 0 | 0 | 0 | 0 | 0 | 0 | 0 | 0.1015 | -0.0002 | 0 | 0.059 | 0 | | | | | | | | | | | | | |
| H1 | -0.1276 | 0 | 0 | 0 | 0.0367 | 0 | 0 | 0 | 0 | 0 | 0 | 0 | 0.0126 | 0.0372 | 0.0124 | | | | | | | | | | | | |
| H2 | 0.1142 | 0 | 0 | -0.0271 | -0.0236 | 0 | 0 | 0 | 0 | 0 | 0 | 0 | -0.0234 | -0.016 | 0 | -0.4255 | | | | | | | | | | | |
| H3 | 0.0009 | 0.0408 | 0 | 0 | 0 | 0 | 0 | 0 | 0 | 0 | 0 | 0 | 0 | 0 | 0.0782 | 0 | 0.0935 | | | | | | | | | | |
| H4 | 0.0185 | 0 | -0.0123 | 0 | 0 | 0.0015 | 0.0164 | 0 | 0.0134 | 0 | 0 | 0 | 0 | 0 | 0.0454 | 0 | 0.0955 | 0.1547 | | | | | | | | | |
| H5 | 0 | 0.0425 | 0 | 0 | 0 | 0 | 0 | 0 | 0 | 0 | 0 | 0 | 0 | 0 | 0.0217 | 0 | 0 | 0.209 | 0.3689 | | | | | | | | |
| H6 | 0 | 0 | 0.0365 | 0 | 0 | 0 | 0 | 0 | 0 | -0.0024 | 0 | 0 | 0 | 0 | 0 | 0 | 0 | 0 | 0 | -0.027 | | | | | | | |
| H7 | 0 | 0 | 0 | 0 | 0 | 0 | 0 | 0 | 0 | 0 | 0 | 0 | 0 | 0.0628 | 0 | 0 | 0 | 0 | 0 | 0 | 0.4753 | | | | | | |
| H8 | 0 | 0 | 0.0714 | 0.0029 | 0 | 0 | 0 | 0.0117 | -0.1055 | -0.0142 | 0.0237 | 0 | 0.0159 | 0.0113 | 0 | 0 | 0 | 0 | 0 | 0 | 0.0516 | 0.2864 | | | | | |
| H9 | 0.0151 | 0 | -0.0489 | 0 | 0 | 0.0543 | 0.001 | 0 | 0 | 0 | 0 | 0 | 0 | 0 | 0.0094 | 0 | 0 | 0.0799 | 0.0382 | 0.0229 | 0 | -0.0285 | 0 | | | | |
| H10 | 0 | 0.0293 | -0.0039 | 0 | 0 | 0.0056 | 0 | 0 | 0.0399 | 0.007 | 0 | 0 | 0 | 0 | 0.0672 | 0 | 0 | 0 | 0.0047 | 0 | -6.00E-04 | 0 | -0.0029 | 0.2444 | | | |
| H11 | 0 | 0 | 0 | 0 | 0 | 0 | 0 | 0 | 0 | 0 | 0 | 0 | 0 | 0 | 0.068 | 0 | 0 | 0.0288 | 0.0265 | 0.1024 | 0 | 0 | 0 | 0.1747 | 0.1953 | | |
| H12 | 0 | -0.0745 | 0 | 0 | 0 | 0 | 0 | 0 | 0 | 0 | 0 | 0 | 0 | 0 | 0 | 0 | 0 | -0.0313 | -0.0175 | -0.0766 | 0.0146 | 0 | 0 | 0 | -0.0648 | -0.113 | |
| H13 | 0 | 0 | -0.0487 | 0 | 0 | 0 | 0 | 0 | 0 | 0 | 0 | 0 | 0 | 0 | 0 | 0 | 0.0311 | 0 | 0 | 0 | -0.0329 | -0.0335 | -0.026 | 0.0126 | 0.0269 | 0 | -0.1315 |

## (W). Singapore

| | L1 | L2 | L3 | L4 | L5 | L6 | L7 | L8 | L9 | L10 | L11 | L12 | L13 | L14 | L18 | H1 | H2 | H3 | H4 | H5 | H6 | H7 | H8 | H9 | H10 | H11 | H12 |
|---|---|---|---|---|---|---|---|---|---|---|---|---|---|---|---|---|---|---|---|---|---|---|---|---|---|---|---|
| L1 | | | | | | | | | | | | | | | | | | | | | | | | | | | |
| L2 | 0.1692 | | | | | | | | | | | | | | | | | | | | | | | | | | |
| L3 | 0 | 0 | | | | | | | | | | | | | | | | | | | | | | | | | |
| L4 | 0 | 0 | 0.2333 | | | | | | | | | | | | | | | | | | | | | | | | |
| L5 | 0 | 0 | 0 | 0.181 | | | | | | | | | | | | | | | | | | | | | | | |
| L6 | 0 | 0.052 | 0 | -0.0335 | 0 | | | | | | | | | | | | | | | | | | | | | | |
| L7 | 0 | 0.1341 | 0 | 0 | 0 | 0.5181 | | | | | | | | | | | | | | | | | | | | | |
| L8 | 0 | 0 | 0.0582 | 0 | 0.0863 | 0 | 0 | | | | | | | | | | | | | | | | | | | | |
| L9 | 0 | 0.0291 | 0 | -0.0143 | 0 | 0.0374 | 0 | 0 | | | | | | | | | | | | | | | | | | | |
| L10 | 0 | 0.1005 | 0 | 0 | 0 | 0.0535 | 0 | 0 | 0.1775 | | | | | | | | | | | | | | | | | | |
| L11 | 0 | 0 | 0.0618 | 0.1024 | 0.1259 | -0.0262 | 0 | 0 | -0.1174 | 0 | | | | | | | | | | | | | | | | | |
| L12 | 0 | 0 | 0 | 0 | 0.0926 | 0 | 0 | 0.0296 | -0.0438 | 0 | 0.4431 | | | | | | | | | | | | | | | | |
| L13 | 0 | 0 | 0 | 0.0965 | 0 | -0.1296 | 0 | 0.0248 | 0 | 0 | 0.0293 | 0 | | | | | | | | | | | | | | | |
| L14 | 0 | 0 | 0.0419 | 0.0134 | 0 | -0.0225 | -0.1303 | 0 | 0 | 0 | 0 | 0 | 0.5871 | | | | | | | | | | | | | | |
| L18 | 0 | 0 | 0 | 0 | 0 | 0 | 0 | 0 | 0 | 0 | 0 | 0 | 0 | 0 | | | | | | | | | | | | | |
| H1 | 0 | 0 | 0 | 0.0271 | 0.0469 | 0 | 0 | 0.067 | 0 | 0 | 0 | 0.0253 | 0.0212 | 0 | 0.0636 | | | | | | | | | | | | |
| H2 | 0.0546 | 0 | 0 | 0 | 0 | 0 | 0 | -0.0304 | 0 | 0 | 0 | 0 | 0 | 0 | 0 | -0.2419 | | | | | | | | | | | |
| H3 | 0 | 0 | 0 | 0 | 0 | 0 | 0 | 0 | 0 | 0 | 0 | 0 | 0 | 0 | 0.2314 | 0 | 0.1565 | | | | | | | | | | |
| H4 | 0 | 0 | 0 | 0 | 0 | 0 | 0 | 0 | 0 | 0 | -0.0006 | 0 | 0 | 0 | 0 | 0 | 0 | 0.1021 | | | | | | | | | |
| H5 | 0 | 0 | 0 | 0 | 0 | 0 | 0 | 0 | 0 | 0 | 0 | 0 | 0 | 0 | 0.1082 | 0 | 0 | 0.0953 | 0.3107 | | | | | | | | |
| H6 | 0 | 0 | 0 | 0.0313 | 0 | 0 | 0 | 0 | -0.0336 | 0 | 0 | 0 | 0 | 0 | 0 | 0 | 0 | 0 | 0 | 0.0377 | | | | | | | |
| H7 | 0 | 0 | 0 | 0.0795 | 0.0473 | 0 | 0 | 0 | -0.0547 | 0 | 0 | 0 | 0 | 0 | 0 | 0.0148 | 0 | 0 | 0 | 0.0689 | 0.6605 | | | | | | |
| H8 | 0 | 0 | 0.0084 | 0.116 | 0 | 0 | 0 | 0 | -0.1477 | 0 | 0.0312 | 0 | 0 | 0 | 0 | 0 | 0 | 0 | 0 | 0 | 0.0549 | 0.0658 | | | | | |
| H9 | 0 | 0 | 0 | 0 | 0 | 0 | 0 | 0 | 0.0052 | 0 | 0 | 0 | 0 | 0 | 0 | 0 | 0 | 0 | 0 | 0 | 0 | 0 | 0 | | | | |
| H10 | 0 | 0 | 0 | 0 | 0 | 0 | 0 | 0 | 0 | 0.0305 | -0.0064 | 0 | 0 | 0 | 0 | 0 | 0 | 0.0496 | 0.0773 | 0 | 0 | 0 | 0 | 0.4232 | | | |
| H11 | 0 | 0 | 0.1501 | 0 | 0 | -0.1197 | -0.0009 | 0 | 0 | -0.0293 | 0 | 0 | 0 | 0.0311 | 0 | 0 | 0.0168 | 0.0596 | 0 | 0.1174 | 0 | 0 | 0.0477 | 0 | 0 | | |
| H12 | 0 | 0 | 0 | 0 | 0 | 0 | 0 | 0 | 0 | 0 | 0 | 0 | 0 | 0 | 0 | 0 | 0 | 0 | 0 | 0 | 0 | 0 | 0 | 0 | 0 | 0 | |
| H13 | 0 | 0 | 0 | 0 | 0 | 0 | 0 | 0 | 0 | 0 | 0 | 0 | 0 | -0.0694 | 0 | -0.0108 | 0 | 0 | 0 | 0 | 0 | -0.0601 | 0 | 0 | 0 | 0 | 0.2699 |

## (X). South Africa

| | L1 | L2 | L3 | L4 | L5 | L6 | L7 | L8 | L9 | L10 | L11 | L12 | L13 | L14 | L18 | H1 | H2 | H3 | H4 | H5 | H6 | H7 | H8 | H9 | H10 | H11 | H12 |
|---|---|---|---|---|---|---|---|---|---|---|---|---|---|---|---|---|---|---|---|---|---|---|---|---|---|---|---|
| L1 | | | | | | | | | | | | | | | | | | | | | | | | | | | |
| L2 | 0 | | | | | | | | | | | | | | | | | | | | | | | | | | |
| L3 | 0 | 0 | | | | | | | | | | | | | | | | | | | | | | | | | |
| L4 | 0 | 0 | 0 | | | | | | | | | | | | | | | | | | | | | | | | |
| L5 | 0 | 0 | 0 | 0.2717 | | | | | | | | | | | | | | | | | | | | | | | |
| L6 | 0 | 0 | 0 | 0 | 0 | | | | | | | | | | | | | | | | | | | | | | |
| L7 | 0 | 0 | 0 | 0 | 0 | 0.5444 | | | | | | | | | | | | | | | | | | | | | |
| L8 | 0 | 0.0412 | 0 | 0 | 0.092 | 0 | 0.0468 | | | | | | | | | | | | | | | | | | | | |
| L9 | 0 | 0 | 0 | 0 | 0 | 0 | 0 | 0 | | | | | | | | | | | | | | | | | | | |
| L10 | 0 | 0 | 0 | 0 | 0 | 0 | 0 | 0 | 0.295 | | | | | | | | | | | | | | | | | | |
| L11 | 0 | 0 | 0 | 0 | 0 | 0 | 0 | 0 | 0 | -0.0614 | | | | | | | | | | | | | | | | | |
| L12 | 0 | 0 | 0 | 0 | 0 | 0 | 0 | 0 | 0 | -0.0417 | 0.4099 | | | | | | | | | | | | | | | | |
| L13 | 0 | 0 | 0.009 | 0.0396 | 0 | 0 | 0 | 0 | -0.0716 | -0.0036 | 0 | 0 | | | | | | | | | | | | | | | |
| L14 | 0 | 0 | 0 | 0.0934 | 0 | -0.0659 | 0 | 0 | 0 | -0.0785 | 0.0224 | 0 | 0.3991 | | | | | | | | | | | | | | |
| L18 | 0 | 0.0948 | 0 | 0 | 0.0807 | 0 | 0 | 0 | 0 | 0 | 0 | 0 | 0.0243 | 0 | | | | | | | | | | | | | |
| H1 | 0 | 0 | 0 | 0.0192 | 0 | 0 | 0 | 0 | 0 | 0 | 0 | 0 | 0.0327 | 0 | 0.017 | | | | | | | | | | | | |
| H2 | 0 | 0 | 0 | -0.1161 | 0 | 0 | 0 | 0 | 0 | 0 | 0 | 0 | 0 | 0 | -0.0912 | -0.3503 | | | | | | | | | | | |
| H3 | 0 | 0.0507 | 0 | 0.0383 | 0 | 0 | 0 | 0 | 0 | 0 | 0 | 0 | 0 | 0 | 0.1154 | 0 | 0 | | | | | | | | | | |
| H4 | 0 | 0 | 0 | 0 | 0 | 0 | 0 | 0 | 0 | 0 | 0 | 0 | 0 | 0 | 0 | 0 | 0 | 0.0934 | | | | | | | | | |
| H5 | 0 | 0 | 0 | 0 | 0 | 0 | 0 | 0 | 0 | 0 | 0 | 0 | 0 | 0.0574 | 0 | 0 | 0 | 0.1746 | 0.2045 | | | | | | | | |
| H6 | 0 | 0 | 0 | 0 | 0 | 0 | 0 | 0 | 0 | 0 | 0 | 0 | 0 | 0 | 0 | 0 | 0 | 0 | 0 | 0 | | | | | | | |
| H7 | 0 | 0 | 0 | 0 | 0 | 0 | 0 | 0 | 0 | -0.0022 | 0 | 0 | 0 | 0.0617 | 0 | 0 | 0 | 0 | 0 | 0 | 0.54 | | | | | | |
| H8 | 0 | 0 | 0 | 0.0197 | 0 | 0 | 0 | 0 | 0 | 0 | 0 | 0.0262 | 0 | 0 | 0 | 0 | 0 | 0 | 0 | 0 | 0.0905 | 0.2499 | | | | | |
| H9 | 0 | 0 | 0 | 0 | 0 | 0 | 0 | 0 | 0.0728 | 0 | 0 | 0 | 0 | 0 | 0 | 0 | 0 | 0 | 0 | 0 | 0 | 0 | 0 | | | | |
| H10 | 0 | 0 | 0 | 0 | 0.0087 | 0 | 0 | 0 | 0 | 0 | 0 | 0 | 0 | 0 | 0.0576 | 0 | 0 | 0 | 0 | 0 | 0 | 0 | 0 | 0.3787 | | | |
| H11 | 0 | 0 | 0 | 0 | 0 | 0 | -0.0683 | 0 | 0 | -0.0372 | 0 | 0 | 0 | 0.056 | 0 | 0 | 0 | 0 | 0.0593 | 0.1509 | 0 | 0 | 0 | 0 | 0.0302 | | |
| H12 | 0 | 0 | 0 | 0 | 0 | 0 | 0 | 0 | 0 | 0 | 0 | 0 | 0 | 0 | 0 | 0 | 0 | 0 | 0 | 0 | 0 | 0 | 0 | 0 | 0 | -0.0757 | |
| H13 | 0 | 0 | 0 | 0 | 0 | 0 | 0 | 0 | 0 | 0 | 0 | 0 | 0 | 0 | 0 | 0 | 0 | 0 | 0 | 0 | 0 | -0.1863 | -0.006 | 0 | 0 | 0 | 0 |

## (Y). South Korea

| | L1 | L2 | L3 | L4 | L5 | L6 | L7 | L8 | L9 | L10 | L11 | L12 | L13 | L14 | L18 | H1 | H2 | H3 | H4 | H5 | H6 | H7 | H8 | H9 | H10 | H11 | H12 |
|---|---|---|---|---|---|---|---|---|---|---|---|---|---|---|---|---|---|---|---|---|---|---|---|---|---|---|---|
| L1 | | | | | | | | | | | | | | | | | | | | | | | | | | | |
| L2 | 0.3811 | | | | | | | | | | | | | | | | | | | | | | | | | | |
| L3 | 0 | -0.0029 | | | | | | | | | | | | | | | | | | | | | | | | | |
| L4 | 0 | 0 | 0.2506 | | | | | | | | | | | | | | | | | | | | | | | | |
| L5 | 0 | 0 | 0.0596 | 0.5 | | | | | | | | | | | | | | | | | | | | | | | |
| L6 | 0 | 0 | -0.0507 | 0 | 0 | | | | | | | | | | | | | | | | | | | | | | |
| L7 | 0 | 0.0744 | -0.0274 | 0 | 0 | 0.6811 | | | | | | | | | | | | | | | | | | | | | |
| L8 | 0 | 0 | 0.1958 | 0.0437 | 0.1491 | -0.0762 | 0 | | | | | | | | | | | | | | | | | | | | |
| L9 | 0.0079 | 0.0248 | 0 | 0 | -0.0123 | 0 | 0 | -0.0824 | | | | | | | | | | | | | | | | | | | |
| L10 | 0 | 0.0479 | -0.0196 | 0 | 0 | 0.0248 | 0.0533 | -0.0075 | 0.4046 | | | | | | | | | | | | | | | | | | |
| L11 | 0 | 0 | 0 | 0 | 0.0358 | 0 | 0.0453 | 0.0412 | -0.0047 | 0 | | | | | | | | | | | | | | | | | |
| L12 | 0 | 0 | 0 | 0.021 | 0.058 | 0.0433 | 0 | 0 | -0.0214 | 0 | 0.4295 | | | | | | | | | | | | | | | | |
| L13 | 0 | 0 | 0.0057 | 0 | 0.0236 | -0.0133 | 0 | 0 | -0.0063 | -0.0343 | 0 | 0 | | | | | | | | | | | | | | | |
| L14 | 0 | 0 | 0.0704 | 0.0295 | 0 | -0.1064 | -0.0488 | 0.0685 | 0 | -0.0164 | 0 | 0 | 0.486 | | | | | | | | | | | | | | |
| L18 | 0.0298 | 0.0458 | 0.007 | 0 | 9.00E-04 | 0 | 0 | 0 | 0.0088 | 0 | 0 | 0 | 0.0529 | 0.0478 | | | | | | | | | | | | | |
| H1 | 0 | 0 | 0.0213 | 0 | 0 | -0.006 | 0 | 0 | 0 | -0.0023 | 0 | 0.0067 | 0.0621 | 0.002 | 0.0748 | | | | | | | | | | | | |
| H2 | 0.0385 | 0 | 0 | -0.0904 | -0.014 | 0 | 0 | -0.0339 | 0 | 0 | 0 | -0.0068 | -0.0177 | 0 | 0 | -0.3383 | | | | | | | | | | | |
| H3 | 0 | 0.0288 | 0.0434 | 0 | 0.0173 | 0 | 0 | 0.0097 | 0 | 0 | 0 | 0 | 0.0192 | 0.0208 | 0.2815 | 0 | 0 | | | | | | | | | | |
| H4 | 0 | 0.0262 | 0.0152 | 0 | 0 | 0 | 0 | 0 | 0 | 0 | 0 | 0 | 0 | 0.0102 | 0.0661 | 0 | 0.0136 | 0.3187 | | | | | | | | | |
| H5 | 0 | 0.0042 | 0.0028 | 0 | 0 | -0.0451 | 0 | 0.0207 | 0 | 0 | 0 | 0 | 0.0185 | 0.0256 | 0.0703 | 0 | 0 | 0.1049 | 0.228 | | | | | | | | |
| H6 | 0 | 0 | 0 | 0 | 0 | 0 | 0 | 0 | 0 | 0 | 0 | 0 | 0 | 0 | 0 | 0 | 0 | 0 | -0.0247 | -0.1382 | | | | | | | |
| H7 | 0 | 0 | 0 | 0 | 0 | 0 | 0 | 0 | 0 | 0 | 0 | 0 | 0 | 0 | 0 | 0 | 0 | -0.0021 | 0 | -0.0078 | 0.7599 | | | | | | |
| H8 | 0 | 0 | 0 | 0.0046 | 0 | 0 | 0 | 0 | 0 | 0 | 0.0669 | 1.00E-04 | 0 | 0 | 0 | 0 | 0 | 0 | 0 | 0 | 0 | 0.0766 | | | | | |
| H9 | 0 | 0.0153 | -0.0037 | 0 | 0 | 0 | 0 | 0 | 0.0199 | 0 | -0.024 | 0 | 0 | 0 | 0 | 0 | 0 | 0.0183 | 0 | 0 | 0 | -0.0174 | -0.0806 | | | | |
| H10 | 0 | 0 | -0.002 | 0 | 0 | 0 | 0 | -0.0036 | 0 | 0 | -0.0435 | 0 | 0 | 0 | 0 | 0 | 0 | 0 | 0 | 0 | 0 | -0.0043 | -0.0043 | 0.5772 | | | |
| H11 | 0 | 0 | 0 | 0 | 0 | -0.0493 | -0.0249 | 0 | 0 | 0 | -0.0054 | -0.0058 | 0.0053 | 0.1062 | 0.0641 | 0 | 0 | 0.0279 | 0.0329 | 0.1799 | 0 | 0 | 0 | 0.0639 | 0 | | |
| H12 | 0 | 0 | 0 | 0 | 0 | 0 | 0 | 0 | 0 | 0 | 0 | 0 | -0.0005 | -0.0558 | 0 | 0 | 0 | -0.0101 | -0.0307 | -0.1002 | 0 | 0 | 0 | -0.0232 | 0 | -0.085 | |
| H13 | 0 | 0 | 0 | 0 | 0 | 0 | 0 | 0 | 0.0155 | 0 | 0 | 0 | 0 | 0 | 0 | 0 | 0 | 0 | 0 | 0 | -0.1199 | -0.0502 | -0.0404 | 0.0138 | 0 | 0 | -0.2477 |

## (Z). Thailand

| | L1 | L2 | L3 | L4 | L5 | L6 | L7 | L8 | L9 | L10 | L11 | L12 | L13 | L14 | L18 | H1 | H2 | H3 | H4 | H5 | H6 | H7 | H8 | H9 | H10 | H11 | H12 |
|---|---|---|---|---|---|---|---|---|---|---|---|---|---|---|---|---|---|---|---|---|---|---|---|---|---|---|---|
| L1 | | | | | | | | | | | | | | | | | | | | | | | | | | | |
| L2 | 0.2561 | | | | | | | | | | | | | | | | | | | | | | | | | | |
| L3 | -0.0851 | 0 | | | | | | | | | | | | | | | | | | | | | | | | | |
| L4 | 0 | 0 | 0.2066 | | | | | | | | | | | | | | | | | | | | | | | | |
| L5 | -0.009 | 0 | 0.1821 | 0.3301 | | | | | | | | | | | | | | | | | | | | | | | |
| L6 | 0.0601 | 0.0806 | 0 | 0 | 0 | | | | | | | | | | | | | | | | | | | | | | |
| L7 | 0 | 0.0491 | -0.0108 | 0 | 0 | 0.4035 | | | | | | | | | | | | | | | | | | | | | |
| L8 | -0.009 | 0 | 0.15 | 0.0331 | 0.0857 | -0.1588 | 0 | | | | | | | | | | | | | | | | | | | | |
| L9 | 0 | 0 | -0.0131 | -0.0239 | 0 | 0 | 0 | 0 | | | | | | | | | | | | | | | | | | | |
| L10 | 0.005 | 0.0296 | -0.0308 | 0 | 0 | 0 | 0 | 0 | 0.3093 | | | | | | | | | | | | | | | | | | |
| L11 | 0 | 0 | 0.0164 | 0 | 0.0202 | 0.0081 | 0 | 0 | -0.1592 | -0.0893 | | | | | | | | | | | | | | | | | |
| L12 | 0 | 0 | 0.0162 | 0.0174 | 0.0074 | 0 | 0 | 0 | -0.059 | -0.0126 | 0.7 | | | | | | | | | | | | | | | | |
| L13 | 0 | 0 | 0.0353 | 0.0328 | 0 | 0 | 0 | 0.0423 | 0 | 0 | 0 | 0 | | | | | | | | | | | | | | | |
| L14 | 0 | 0 | 0 | 0.0532 | 0.061 | -0.0971 | 0 | 0.0284 | 0 | 0 | 0 | 0 | 0.483 | | | | | | | | | | | | | | |
| L18 | 0 | 0.0435 | 0 | 0 | 0 | 0 | 0.0919 | 0 | 0.0622 | 0 | -0.019 | -0.0329 | 0 | 0 | | | | | | | | | | | | | |
| H1 | -0.0868 | 0 | 0.0347 | 0 | 0 | 0 | 0 | 0.0284 | 0 | 0 | 0 | 0 | 0 | 0.0305 | 0 | | | | | | | | | | | | |
| H2 | 0.039 | 0 | -0.0089 | -0.0343 | -0.0178 | 0 | 0 | 0 | 0 | 0 | 0 | 0 | -0.0325 | 0 | 0 | -0.37 | | | | | | | | | | | |
| H3 | 0.0021 | 0 | -0.0059 | 0 | 0 | 0.0379 | 0 | 0 | 0 | 0 | 0 | 0 | -0.0137 | -0.0431 | 0.0568 | -0.232 | 0.1487 | | | | | | | | | | |
| H4 | 0.0164 | 0.0472 | 0 | -0.0086 | 0 | 0.0129 | 0 | 0 | 0 | 0 | 0 | 0 | -0.0354 | 0 | 0 | 0 | 0.081 | 0.2662 | | | | | | | | | |
| H5 | 0 | 0 | 0 | 0 | 0 | 0.0308 | 0 | 0 | 0 | 0 | 0 | 0 | 0 | -0.0226 | 0 | 0 | 0 | 0.1413 | 0.1932 | | | | | | | | |
| H6 | 0 | 0 | 0 | 0 | 0 | 0 | 0 | 0 | 0 | 0 | 0 | 0 | 0.0012 | 0.0091 | 0 | 0 | 0 | 0 | -0.0574 | -0.2432 | | | | | | | |
| H7 | 0 | 0 | 0 | 0 | 0.0298 | 0 | 0 | 0 | 0 | 0 | 0 | 0 | 0 | 0.0059 | 0 | 0 | 0 | -0.0263 | 0 | -0.0696 | 0.5033 | | | | | | |
| H8 | 0 | 0 | 0 | 0.0054 | 0.0344 | 0 | 0.0062 | 0 | -0.0192 | -0.0242 | 0 | 0.0233 | 0 | 0 | 0 | 0 | 0 | 0 | 0 | 0 | 0 | 0.1292 | | | | | |
| H9 | 0 | 0 | 0 | 0 | 0 | 0.0039 | 0 | -0.0032 | 0.0158 | 0.0328 | 0 | 0 | 0 | 0 | 0.0041 | 0 | 0 | 0.0129 | 0.0473 | 0.0674 | 0 | -0.0031 | -0.0255 | | | | |
| H10 | 0.0168 | 0 | -0.0087 | 0 | 0 | 0 | 0 | 0 | 0.0704 | 0.0148 | -0.0144 | 0 | 0 | 0 | 0.0297 | 0 | 0 | 0 | 0.0070 | 0.0003 | 0 | 0 | -0.1808 | 0.277 | | | |
| H11 | 0 | 0 | -0.0172 | 0 | 0 | 0 | 0 | 0 | 0.0842 | 0 | -0.0282 | -0.031 | 0 | 0 | 0.1037 | 0 | 0 | 0 | 0 | 0.1126 | 0 | -0.0266 | -0.1183 | 0 | 0.0098 | | |
| H12 | 0 | 0 | 0 | 7.00E-04 | 0 | 0 | 0 | 0 | 0 | 0 | 0 | 0 | 0 | 0 | -0.041 | 0 | 0 | 0 | 0 | -0.098 | 0.0288 | 0 | 0 | -0.0347 | -0.0996 | -0.1717 | |
| H13 | 0 | 0 | 0 | 0 | 0 | 0 | 0 | 0 | 0 | 0 | 0 | 0 | 0 | 0 | 0 | -0.0172 | 0 | 0.0024 | 0 | 0.0176 | -0.1101 | -0.0577 | 0 | 0.0075 | 0 | 0.0298 | -0.392 |

## (AA). United Kingdom

| | L1 | L2 | L3 | L4 | L5 | L6 | L7 | L8 | L9 | L10 | L11 | L12 | L13 | L14 | L18 | H1 | H2 | H3 | H4 | H5 | H6 | H7 | H8 | H9 | H10 | H11 | H12 |
|---|---|---|---|---|---|---|---|---|---|---|---|---|---|---|---|---|---|---|---|---|---|---|---|---|---|---|---|
| L1 | | | | | | | | | | | | | | | | | | | | | | | | | | | |
| L2 | 0.0195 | | | | | | | | | | | | | | | | | | | | | | | | | | |
| L3 | 0 | 0 | | | | | | | | | | | | | | | | | | | | | | | | | |
| L4 | 0 | 0 | 0.2031 | | | | | | | | | | | | | | | | | | | | | | | | |
| L5 | 0 | 0 | 0.1349 | 0.2164 | | | | | | | | | | | | | | | | | | | | | | | |
| L6 | 0 | 0 | 0 | 0 | 0 | | | | | | | | | | | | | | | | | | | | | | |
| L7 | 0 | 0 | 0 | 0 | 0 | 0.5349 | | | | | | | | | | | | | | | | | | | | | |
| L8 | 0 | 0.0848 | 0 | 0.1342 | 0.1003 | 0 | 0 | | | | | | | | | | | | | | | | | | | | |
| L9 | 0 | 0 | 0 | 0 | 0 | 0 | 0 | 0 | | | | | | | | | | | | | | | | | | | |
| L10 | 0 | 0 | 0 | 0 | 0 | 0 | 0 | 0 | 0.1336 | | | | | | | | | | | | | | | | | | |
| L11 | 0 | 0 | 0 | 0 | 0 | 0 | 0 | 0 | 0 | 0 | | | | | | | | | | | | | | | | | |
| L12 | 0 | 0 | 0.0172 | 0 | 0 | 0 | 0 | 0.1317 | 0 | 0 | 0.1655 | | | | | | | | | | | | | | | | |
| L13 | 0 | 0 | 0 | 0 | 0 | 0 | 0 | 0 | 0 | 0 | 0 | 0 | | | | | | | | | | | | | | | |
| L14 | 0 | 0 | 0 | 0.0289 | 0 | 0 | 0 | 0 | 0 | 0 | 0 | 0 | 0.4402 | | | | | | | | | | | | | | |
| L18 | 0 | 0.0444 | 0 | 0 | 0 | 0 | 0 | 0 | 0 | 0 | 0 | 0 | 0 | 0.0005 | | | | | | | | | | | | | |
| H1 | 0 | 0 | 0 | 0.0193 | 0 | 0 | 0 | 0 | 0 | 0 | 0 | 0 | 0 | 0 | 0.0399 | | | | | | | | | | | | |
| H2 | 0 | 0 | 0 | -0.0553 | 0 | 0 | 0 | 0 | 0 | 0 | 0 | 0 | 0 | 0 | 0 | -0.1853 | | | | | | | | | | | |
| H3 | 0 | 0 | 0 | 0 | 0 | 0 | 0 | 0 | 0 | 0 | 0 | 0 | 0 | 0 | 0.1645 | 0 | 0 | | | | | | | | | | |
| H4 | 0 | 0 | 0 | 0 | 0 | 0 | 0 | 0 | 0 | 0 | 0 | 0 | 0 | 0 | 0 | 0 | 0 | 0.2344 | | | | | | | | | |
| H5 | 0 | 0 | 0 | 0 | 0 | 0 | 0 | 0 | 0 | 0 | 0 | 0 | 0 | 0.0605 | 0.034 | 0 | 0 | 0.1283 | 0.1025 | | | | | | | | |
| H6 | 0 | 0 | 0 | 0 | 0 | 0 | 0 | 0 | 0 | 0 | 0 | 0 | 0 | 0 | 0 | 0 | 0 | 0 | 0 | 0 | | | | | | | |
| H7 | 0 | 0 | 0 | 0 | 0 | 0 | 0 | 0 | 0 | 0 | 0 | 0 | 0 | 0 | 0 | 0 | 0 | 0 | 0 | 0 | 0.5584 | | | | | | |
| H8 | 0 | 0 | 0 | 0 | 0 | 0 | 0 | 0 | 0 | 0 | 0 | 0 | 0 | 0 | 0 | 0 | 0 | 0 | 0 | 0 | 0 | 0.0671 | | | | | |
| H9 | 0 | 0 | 0 | 0 | 0 | 0 | 0 | 0 | 0 | 0 | 0 | 0 | 0 | 0 | 0 | 0 | 0 | 0 | 0 | 0 | 0 | 0 | 0 | | | | |
| H10 | 0 | 0 | 0 | 0 | 0 | 0 | 0 | 0 | 0 | 0 | 0 | 0 | 0 | 0.0088 | 0 | 0 | 0 | 0 | 0 | 0 | 0 | 0 | 0 | 0.2886 | | | |
| H11 | 0 | 0 | 0 | 0 | 0 | 0 | 0 | 0 | 0 | 0 | 0 | 0 | 0 | 0.0258 | 0 | 0 | 0 | 0 | 0 | 0.2032 | 0 | 0 | 0 | 0.0047 | 0.1071 | | |
| H12 | 0 | 0 | 0 | 0 | 0 | 0 | 0 | 0 | 0 | 0 | 0 | 0 | 0 | 0 | 0 | 0 | 0 | 0 | 0 | 0 | 0 | 0 | 0 | 0 | -0.039 | 0 | |
| H13 | 0 | 0 | 0 | 0 | 0 | 0 | 0 | 0 | 0 | 0 | 0 | 0 | 0 | 0 | 0 | 0 | 0 | 0 | 0 | 0 | -0.029 | 0 | 0 | 0 | 0 | 0 | 0 |

## (AB). United States

| | L1 | L2 | L3 | L4 | L5 | L6 | L7 | L8 | L9 | L10 | L11 | L12 | L13 | L14 | L18 | H1 | H2 | H3 | H4 | H5 | H6 | H7 | H8 | H9 | H10 | H11 | H12 |
|---|---|---|---|---|---|---|---|---|---|---|---|---|---|---|---|---|---|---|---|---|---|---|---|---|---|---|---|
| L1 | | | | | | | | | | | | | | | | | | | | | | | | | | | |
| L2 | 0.1121 | | | | | | | | | | | | | | | | | | | | | | | | | | |
| L3 | 0 | 0.1336 | | | | | | | | | | | | | | | | | | | | | | | | | |
| L4 | 0 | 0.0155 | 0.0062 | | | | | | | | | | | | | | | | | | | | | | | | |
| L5 | 0 | 0.1067 | 0.1057 | 0.0969 | | | | | | | | | | | | | | | | | | | | | | | |
| L6 | 0 | 0 | 0 | 0 | 0 | | | | | | | | | | | | | | | | | | | | | | |
| L7 | 0 | 0 | 0 | 0 | 0 | 0.5735 | | | | | | | | | | | | | | | | | | | | | |
| L8 | 0 | 0 | 0.0549 | 0.0478 | 0 | 0 | 0.0569 | | | | | | | | | | | | | | | | | | | | |
| L9 | 0 | 0 | 0 | 0 | 0 | 0 | 0 | 0 | | | | | | | | | | | | | | | | | | | |
| L10 | 0 | 0 | 0 | 0 | 0 | 0 | 0 | 0 | 0.3531 | | | | | | | | | | | | | | | | | | |
| L11 | 0 | 0 | 0 | 0 | 0 | 0 | 0 | 0 | -0.0688 | 0 | | | | | | | | | | | | | | | | | |
| L12 | 0 | 0 | 0 | 0.0017 | 0 | 0 | 0 | 0 | 0 | 0 | 0.1381 | | | | | | | | | | | | | | | | |
| L13 | 0 | 0 | 0 | 0 | 0 | -0.0425 | 0 | 0 | 0 | 0 | 0 | 0 | | | | | | | | | | | | | | | |
| L14 | 0 | 0 | 0 | 0 | 0 | 0 | 0 | 0 | 0 | 0 | 0 | 0 | 0.507 | | | | | | | | | | | | | | |
| L18 | 0 | 0 | 0 | 0 | 0 | 0 | 0 | 0 | 0 | 0 | 0 | 0 | 0.0797 | 0 | | | | | | | | | | | | | |
| H1 | 0 | 0.0318 | 0 | 0 | 0 | 0 | 0 | 0 | 0 | 0 | 0 | 0 | 0 | 0 | 0.0419 | | | | | | | | | | | | |
| H2 | 0 | 0 | 0 | -0.2369 | 0 | 0 | 0 | 0 | 0 | 0 | 0 | -0.025 | 0 | 0 | 0 | -0.3188 | | | | | | | | | | | |
| H3 | 0 | 0 | 0 | 0 | 0 | 0 | 0 | 0 | 0 | 0 | 0 | 0 | 0 | 0 | 0.2202 | 0.0108 | 0 | | | | | | | | | | |
| H4 | 0 | 0 | 0 | 0 | 0 | 0 | 0 | 0 | 0 | 0 | 0 | 0 | 0 | 0 | 0 | 0 | 0 | 0.0513 | | | | | | | | | |
| H5 | 0 | 0 | 0 | 0 | 0 | 0 | 0 | 0 | 0 | 0 | 0 | 0 | 0 | 0 | 0 | 0 | 0 | 0.0853 | 0.2777 | | | | | | | | |
| H6 | 0 | 0 | 0 | 0 | 0 | 0 | 0 | 0 | 0 | 0 | 0 | 0 | 0 | 0 | 0 | 0 | 0 | 0 | 0 | 0 | | | | | | | |
| H7 | 0 | 0 | 0 | 0 | 0 | 0 | 0 | 0 | 0 | 0 | 0 | 0 | 0 | 0 | 0 | 0 | 0 | 0 | 0 | 0 | 0.615 | | | | | | |
| H8 | 0 | 0 | 0 | 0 | 0 | 0 | 0 | 0 | 0 | 0 | 0 | 0 | 0 | 0 | 0 | 0 | 0 | 0 | 0 | 0 | 0 | 0.0381 | | | | | |
| H9 | 0 | 0 | 0 | 0 | 0 | 0 | 0 | 0 | 0 | 0 | 0 | 0 | 0 | 0 | 0 | 0 | 0 | 0 | 0 | 0 | 0 | 0 | 0 | | | | |
| H10 | 0 | 0 | 0 | 0 | 0 | 0 | 0 | 0 | 0 | 0 | 0 | 0 | 0 | 0 | 0 | 0 | 0 | 0 | 0 | 0.0178 | 0 | 0 | 0 | 0.3192 | | | |
| H11 | 0 | 0 | 0 | 0 | 0 | 0 | 0 | 0 | 0 | 0 | 0 | 0 | 0.0186 | 0 | 0 | 0 | 0 | 0 | 0 | 0.1644 | 0 | 0 | 0 | 0 | 0.0359 | | |
| H12 | 0 | 0 | 0 | 0 | 0 | 0 | 0 | 0 | 0 | 0 | 0 | 0 | 0 | 0 | 0 | 0 | 0 | 0 | 0 | 0 | 0 | 0 | 0 | 0 | 0 | 0 | |
| H13 | 0 | 0 | 0 | 0 | 0 | 0 | 0 | 0 | 0 | 0 | 0 | 0 | 0 | 0 | 0 | 0 | 0 | 0 | 0 | 0 | 0 | -0.0066 | -0.0074 | 0 | 0 | 0 | 0 |

## (AC). Vietnam

| | L1 | L2 | L3 | L4 | L5 | L6 | L7 | L8 | L9 | L10 | L11 | L12 | L13 | L14 | L18 | H1 | H2 | H3 | H4 | H5 | H6 | H7 | H8 | H9 | H10 | H11 | H12 |
|---|---|---|---|---|---|---|---|---|---|---|---|---|---|---|---|---|---|---|---|---|---|---|---|---|---|---|---|
| L1 | | | | | | | | | | | | | | | | | | | | | | | | | | | |
| L2 | 0.3659 | | | | | | | | | | | | | | | | | | | | | | | | | | |
| L3 | -0.0752 | 0 | | | | | | | | | | | | | | | | | | | | | | | | | |
| L4 | -0.1194 | 0 | 0.2807 | | | | | | | | | | | | | | | | | | | | | | | | |
| L5 | 0 | 0 | 0.1202 | 0.371 | | | | | | | | | | | | | | | | | | | | | | | |
| L6 | 0 | 0.0674 | 0 | 0 | 0.0271 | | | | | | | | | | | | | | | | | | | | | | |
| L7 | 0 | 0.0891 | -0.0063 | 0 | 0 | 0.6795 | | | | | | | | | | | | | | | | | | | | | |
| L8 | 0 | -0.0272 | 0.0406 | 0 | 0 | -0.1907 | -0.2052 | | | | | | | | | | | | | | | | | | | | |
| L9 | 0 | 0 | 0 | 0 | 0 | 0 | 0 | -0.1044 | | | | | | | | | | | | | | | | | | | |
| L10 | 0 | 0 | -0.0514 | 0 | -0.0491 | 0 | 0 | -0.0943 | 0.4899 | | | | | | | | | | | | | | | | | | |
| L11 | 0 | 0.0113 | 0 | 0 | 0.1018 | 0.0531 | 0 | 0 | -0.1196 | -0.099 | | | | | | | | | | | | | | | | | |
| L12 | 0 | 0.0231 | 0 | 0 | 0.0039 | 0 | 0.0158 | 0 | -0.0332 | 0 | 0.7518 | | | | | | | | | | | | | | | | |
| L13 | 0 | 0.0178 | 0.0218 | 0 | 0 | 0 | 0 | -0.0407 | 0 | 0 | 0 | 0.0989 | | | | | | | | | | | | | | | |
| L14 | 0 | 0 | 0.0273 | 0 | 0.034 | -0.0371 | -0.0125 | 0 | 0 | -0.024 | 0.0339 | 0 | 0.5014 | | | | | | | | | | | | | | |
| L18 | 0 | 0.0082 | 0 | 0 | 0 | 0 | 0 | 0 | 0.0037 | 0 | 0 | 0 | 0 | 0 | | | | | | | | | | | | | |
| H1 | -0.0258 | 0 | 0.0384 | 0.1155 | 0.0068 | 0 | 0 | 0 | 0 | 0 | 0 | 0 | 0.121 | 0.0769 | 0.0016 | | | | | | | | | | | | |
| H2 | 0.1036 | 0.0859 | 0 | 0 | 0 | 0 | 0 | 0 | 0 | -0.0111 | 0 | 0 | 0 | 0.0254 | 0.0849 | -0.0921 | | | | | | | | | | | |
| H3 | 0.001 | 0.0333 | 0 | 0 | 0 | 0.0043 | 0 | 0 | 0 | 0 | 0.0071 | 0 | 0 | 0 | 0.0347 | -0.235 | 0.2893 | | | | | | | | | | |
| H4 | 0 | 0.025 | 0 | 0 | 0 | 0.0201 | 0.0226 | 0 | 0 | 0 | 0 | 0 | 0 | -0.0373 | 0 | 0 | 0.1653 | 0.1731 | | | | | | | | | |
| H5 | 0.0409 | 0.0702 | 0 | 0 | -0.0532 | 0 | 0 | 0 | 0 | 0 | 0.0012 | 0 | 0.0375 | 0 | 0.037 | 0 | 0.1837 | 0.2215 | 0.197 | | | | | | | | |
| H6 | 0 | 0 | 0 | 0 | 0 | 0 | 0 | 0 | 0 | 0 | 0 | 0 | 0 | 0.0332 | 0 | 0.003 | 0 | 0 | 0 | 0 | | | | | | | |
| H7 | 0 | 0.0053 | 0 | 0.0699 | 0 | 0 | 0 | 0 | 0 | 0 | 0 | 0 | 0.028 | 0 | 0 | 0.0343 | 0 | 0 | 0.0583 | 0.008 | 0.5555 | | | | | | |
| H8 | 0 | 0.0437 | 0.1263 | 0 | 0.014 | 0 | 0 | 0 | -0.0315 | -0.0064 | 0.016 | 0.0848 | 0.0129 | 0.0824 | 0.097 | 0 | 0.0554 | 0.0356 | 0 | 0.0653 | 0 | 0.3432 | | | | | |
| H9 | 0 | 0 | 0 | 0 | 0 | 0 | 0 | 0 | 0.0386 | 0 | -0.002 | -0.0097 | 0 | -0.0014 | 0 | 0 | 0 | 0 | 0 | 0 | 0 | 0 | -0.1118 | | | | |
| H10 | 0 | 0.0089 | 0 | 0 | -0.0183 | -0.0202 | 0 | 0 | 0 | 0.0211 | -0.0073 | 0 | 0 | 0 | 0 | 0 | 0 | 0 | 0 | 0 | 0 | -0.0227 | -0.0807 | 0.7245 | | | |
| H11 | 0 | 0.0546 | 0.0018 | 0.0054 | 0 | 0 | 0 | 0 | 0 | 0 | 0 | 0 | 0 | 0.0119 | 0.1244 | 0.0233 | 0.0262 | 0.0753 | 0 | 0.0457 | 0.0537 | 0 | 0.0584 | 0.0383 | 0.1905 | | |
| H12 | -0.0998 | -0.019 | -0.0501 | 0 | 0 | 0 | 0.0627 | 0 | -0.0283 | 0 | 0 | 0 | 0 | -0.0388 | 0 | 0 | -0.0042 | 0 | 0 | -0.1774 | 0 | 0 | 0 | -0.052 | -0.0176 | -0.2551 | |
| H13 | 0 | 0 | 0 | 0 | 0 | 0 | 0 | 0 | 0.0458 | 0.0053 | 0 | 0 | 0 | -0.011 | 0 | 0 | 0 | 0 | 0 | 0 | -0.0874 | -0.1403 | -0.0396 | 0 | 0 | 0 | -0.084 |

**Figure S1.** Bootstrapped confidence intervals of edge weights for the lifestyles network across 29 countries.

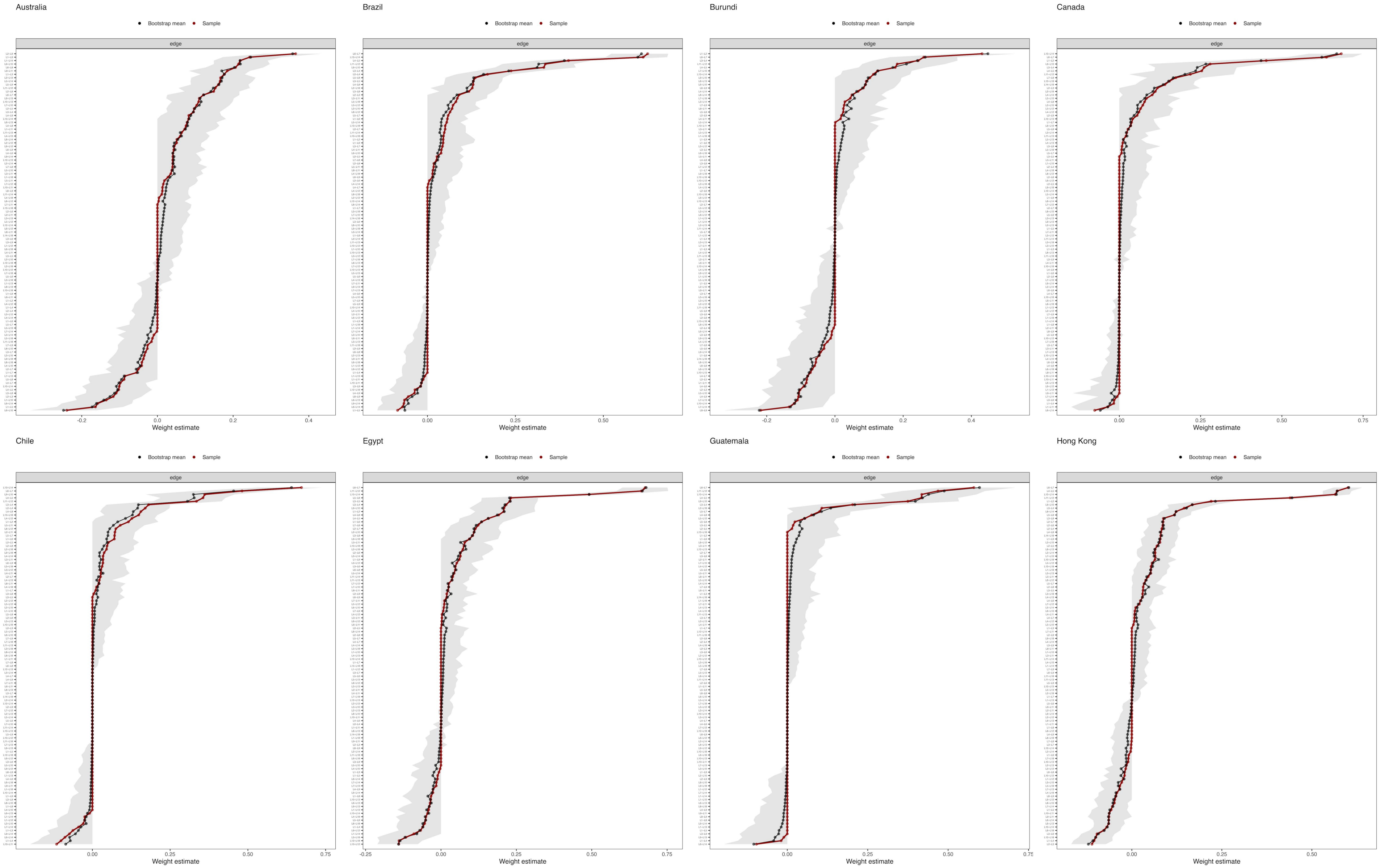

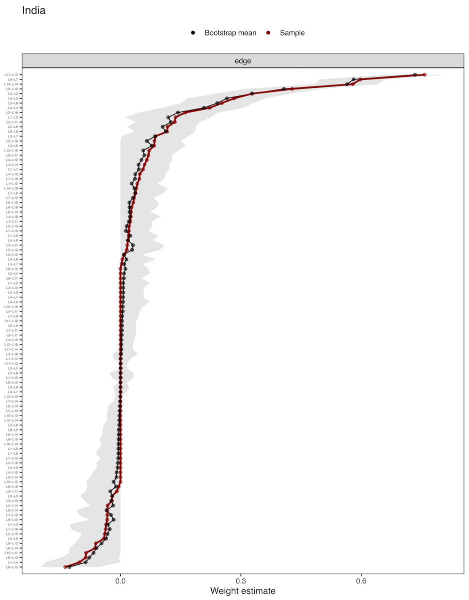
India
Bootstrap mean
Sample
edge
0.0
0.3
0.6
Weight estimate

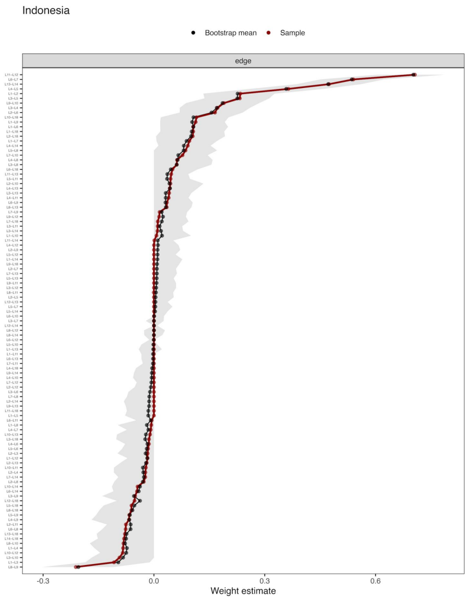
Indonesia
Bootstrap mean
Sample
edge
-0.3
0.0
0.3
0.6
Weight estimate

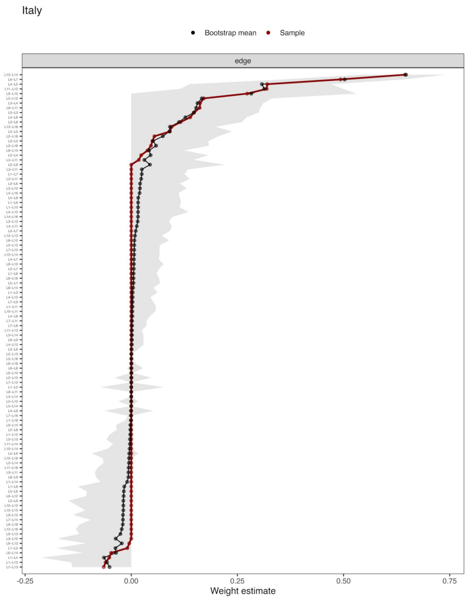
Italy
Bootstrap mean
Sample
edge
-0.25
0.00
0.25
0.50
0.75
Weight estimate

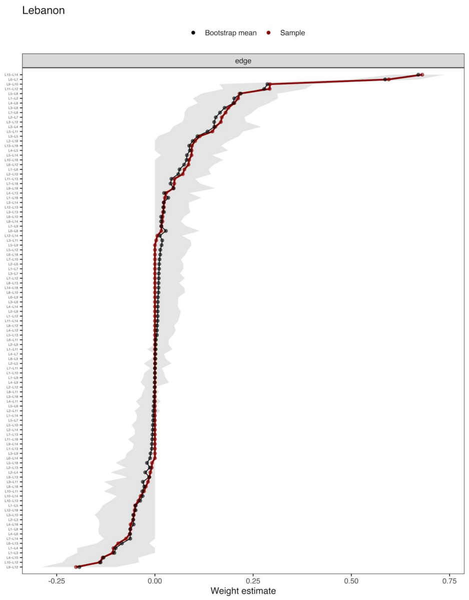
Lebanon
Bootstrap mean
Sample
edge
-0.25
0.00
0.25
0.50
0.75
Weight estimate

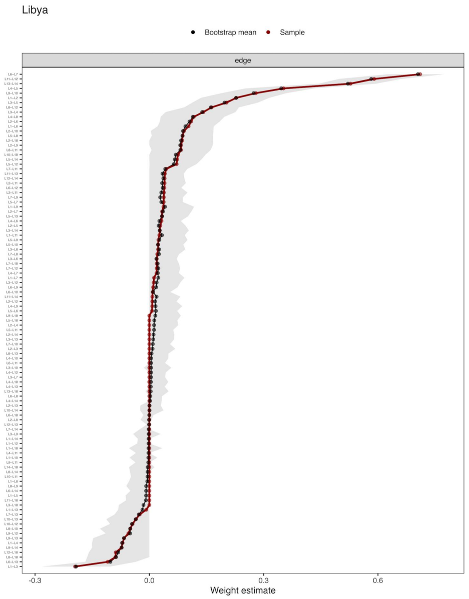
Libya
Bootstrap mean
Sample
edge
-0.3
0.0
0.3
0.6
Weight estimate

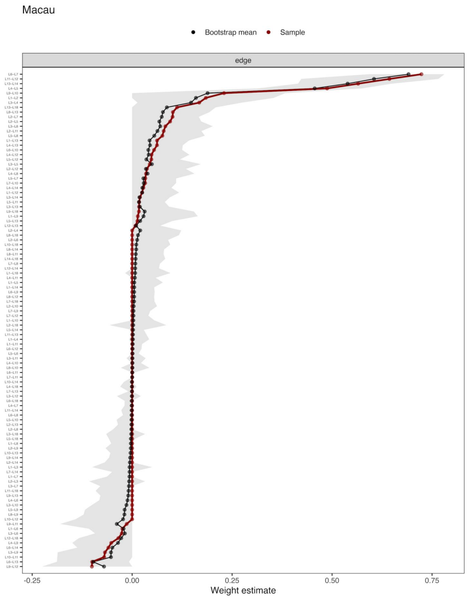
Macau
Bootstrap mean
Sample
edge
-0.25
0.00
0.25
0.50
0.75
Weight estimate

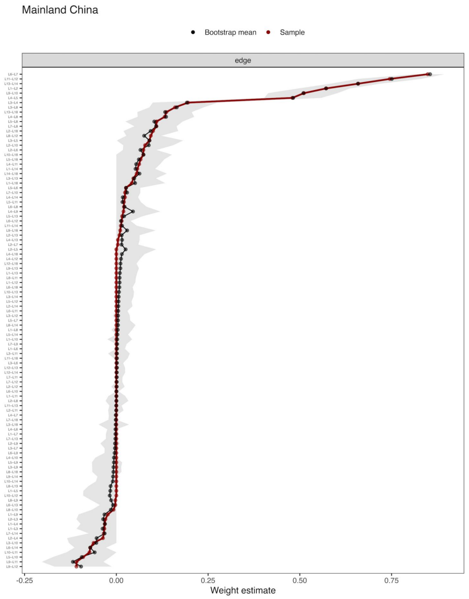
Mainland China
Bootstrap mean
Sample
edge
-0.25
0.00
0.25
0.50
0.75
Weight estimate

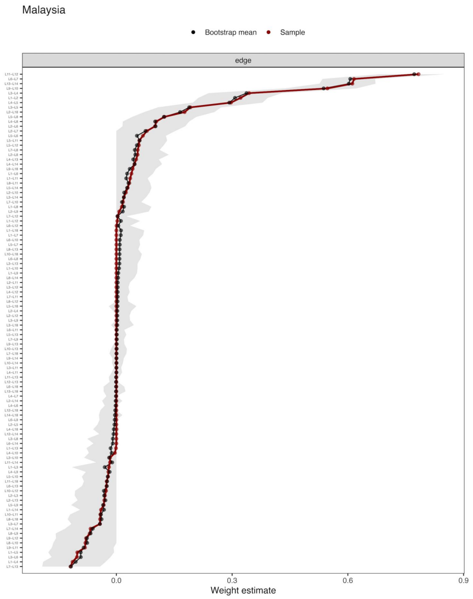
Malaysia
Bootstrap mean
Sample
edge
0.0
0.3
0.6
0.9
Weight estimate

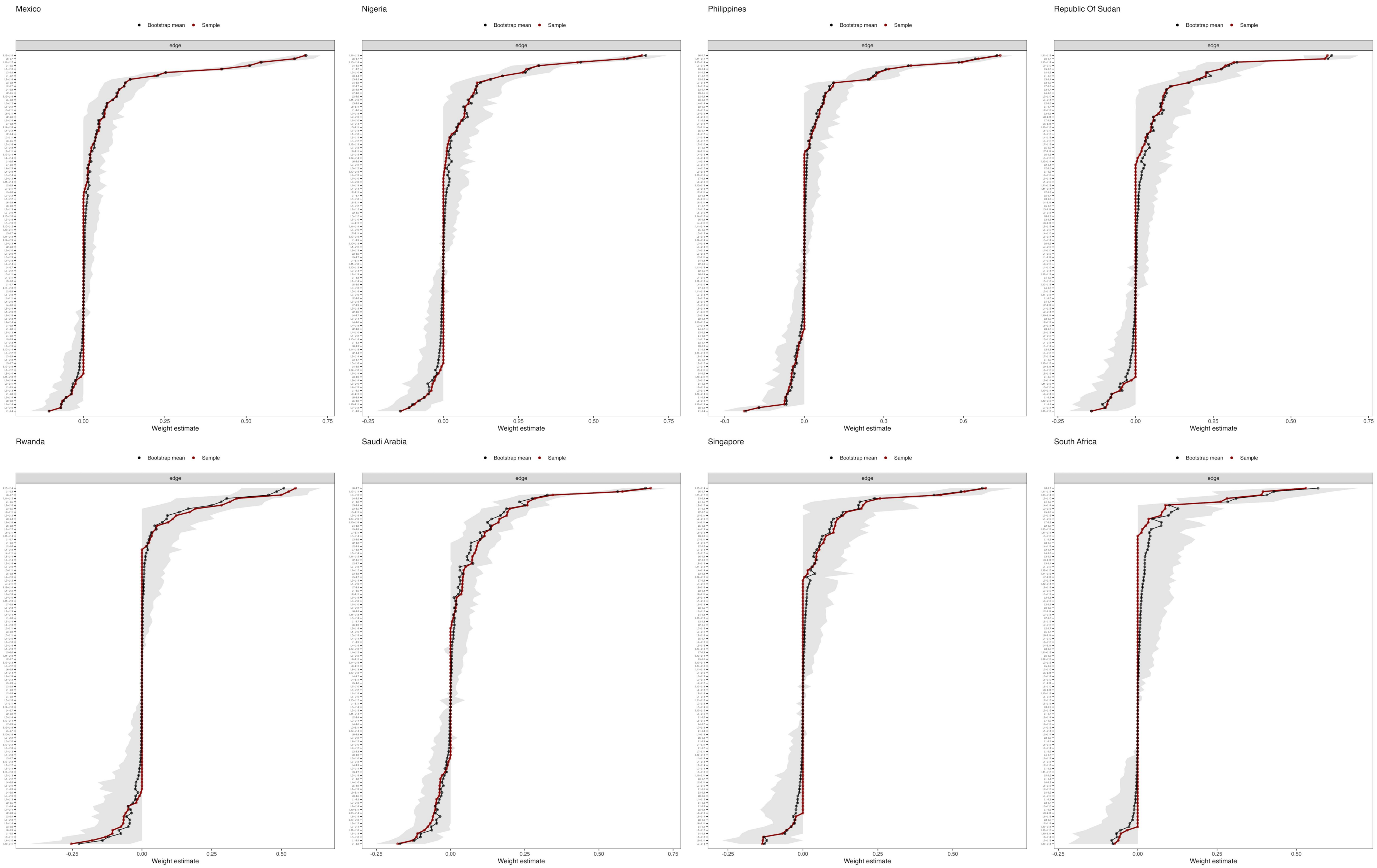
Mexico
Bootstrap mean
Sample
edge
0.00
0.25
0.50
0.75
Weight estimate
Nigeria
Bootstrap mean
Sample
edge
-0.25
0.00
0.25
0.50
0.75
Weight estimate
Philippines
Bootstrap mean
Sample
edge
-0.3
0.0
0.3
0.6
Weight estimate
Republic Of Sudan
Bootstrap mean
Sample
edge
-0.25
0.00
0.25
0.50
0.75
Weight estimate
Rwanda
Bootstrap mean
Sample
edge
-0.25
0.00
0.25
0.50
Weight estimate
Saudi Arabia
Bootstrap mean
Sample
edge
-0.25
0.00
0.25
0.50
0.75
Weight estimate
Singapore
Bootstrap mean
Sample
edge
-0.25
0.00
0.25
0.50
Weight estimate
South Africa
Bootstrap mean
Sample
edge
-0.25
0.00
0.25
0.50
Weight estimate

South Korea

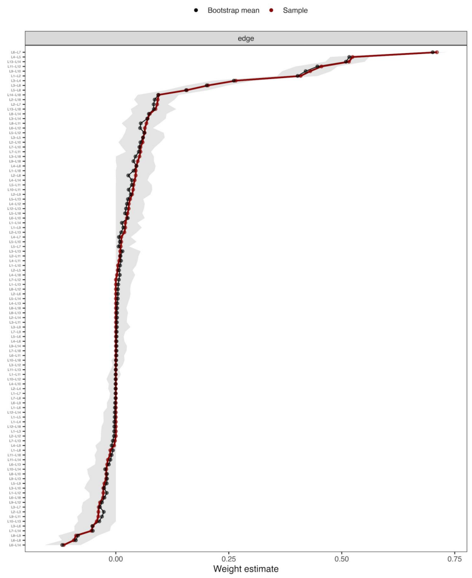


Thailand

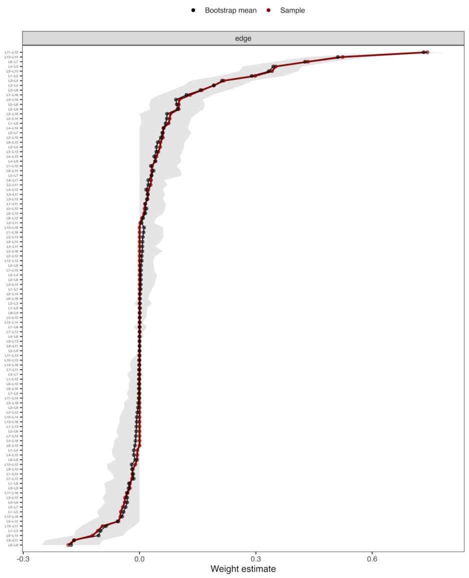


United Kingdom

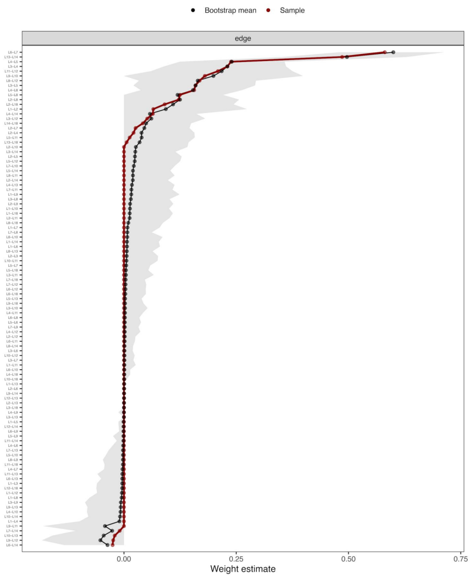


United States

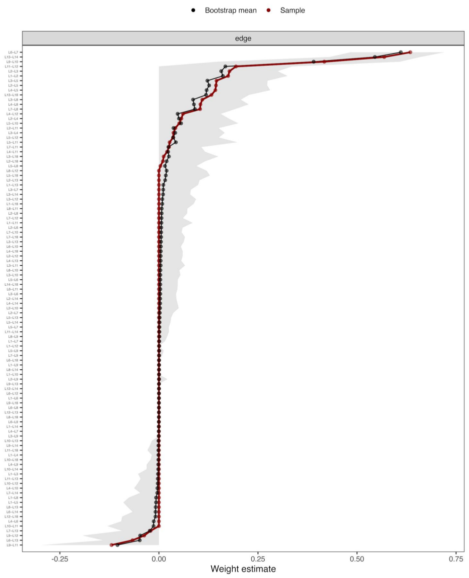


Vietnam

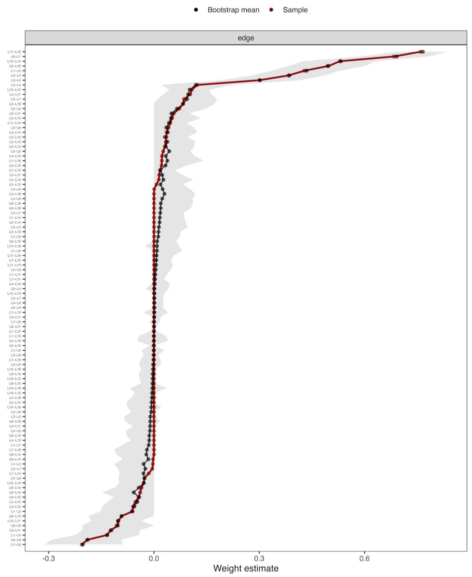

**Figure S2.** The stability of expected influence centrality index in lifestyles network across 29 countries using case-dropping bootstrap

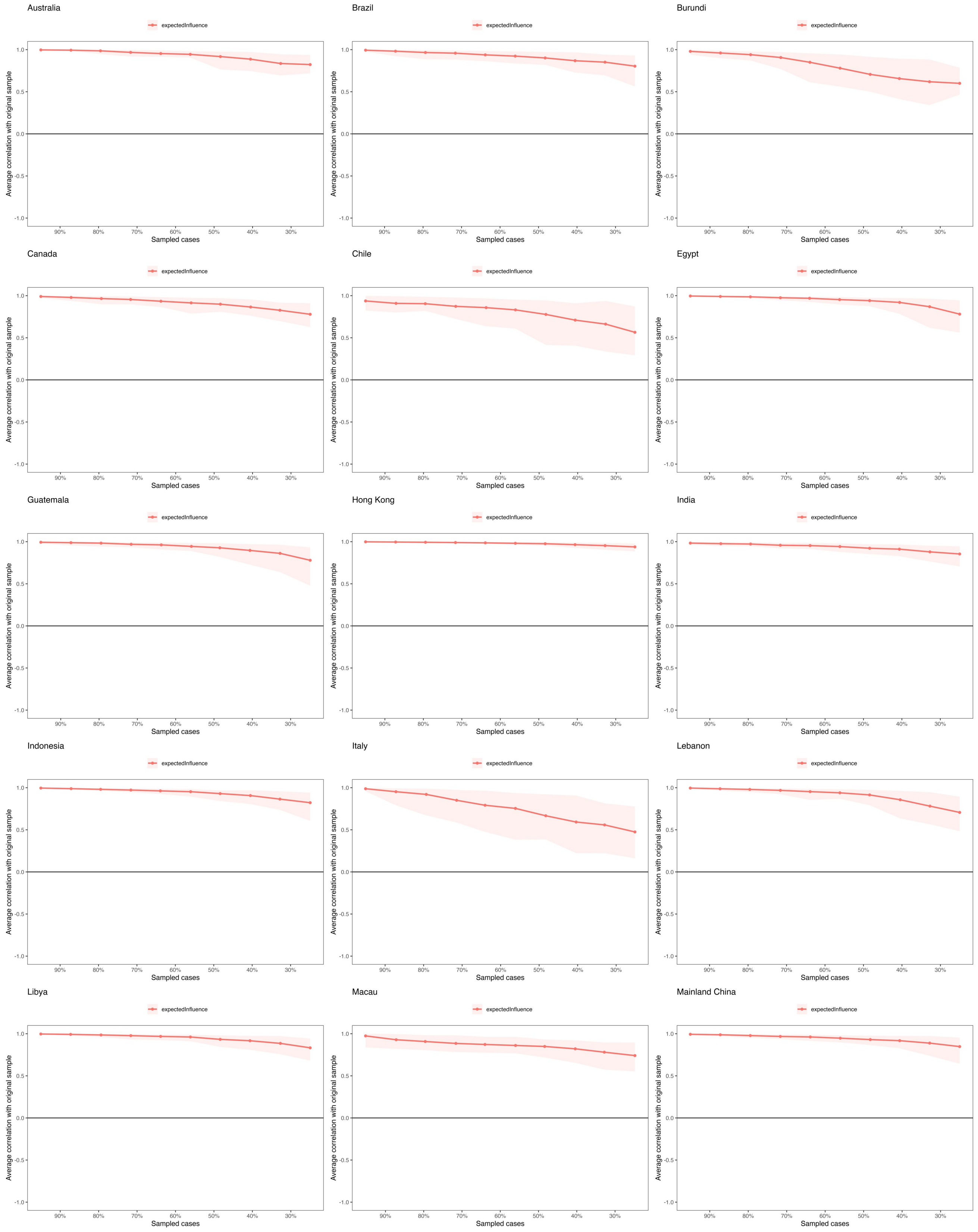

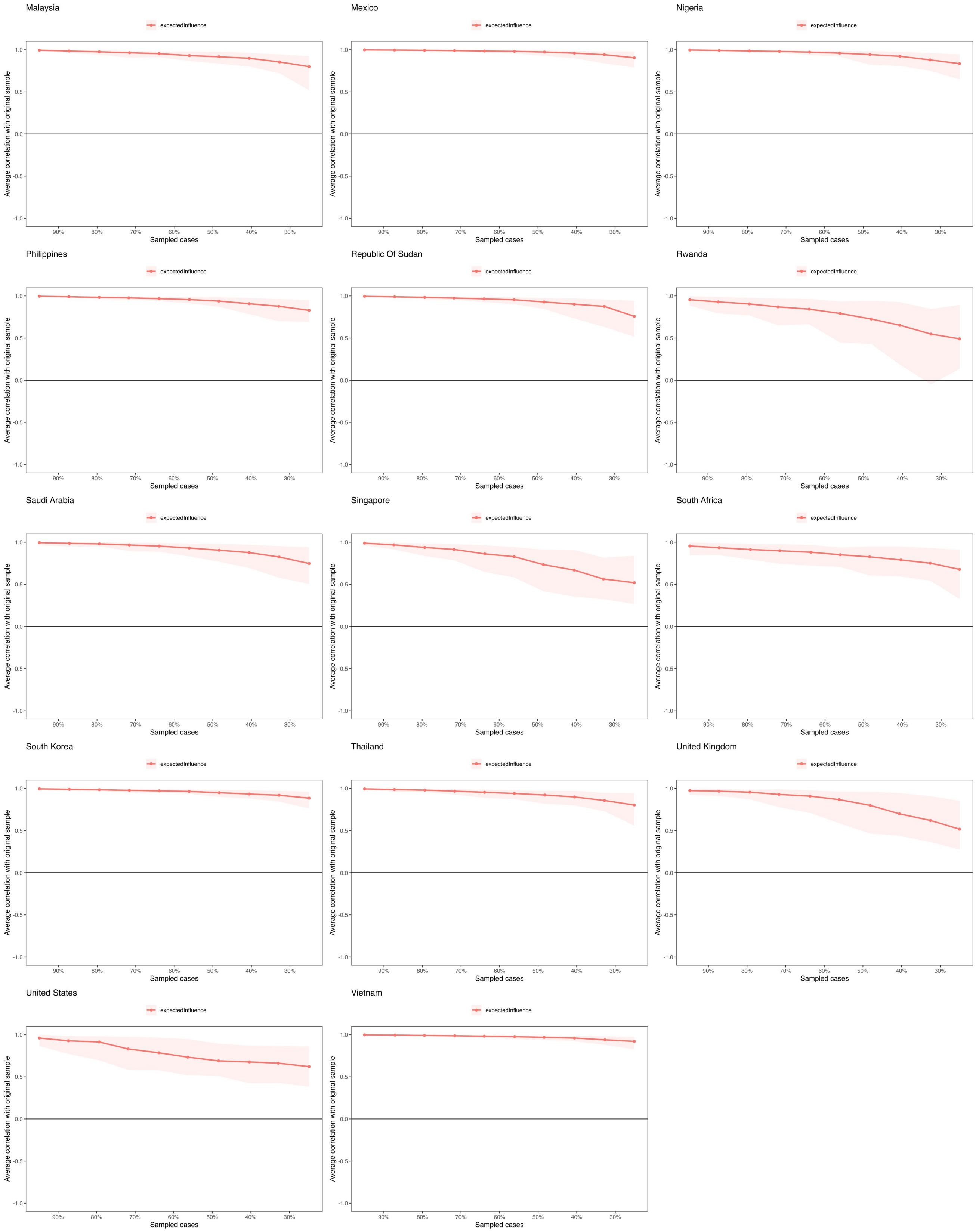
Malaysia
Mexico
Nigeria
Philippines
Republic Of Sudan
Rwanda
Saudi Arabia
Singapore
South Africa
South Korea
Thailand
United Kingdom
United States
Vietnam
expectedInfluence
Average correlation with original sample
Sampled cases
1.0
0.5
0.0
-0.5
-1.0
90%
80%
70%
60%
50%
40%
30%

**Figure S3.** Network structure of lifestyle (A), health outcomes (B), and bridge that combined two (C) across 29 countries

**(A). Australia**

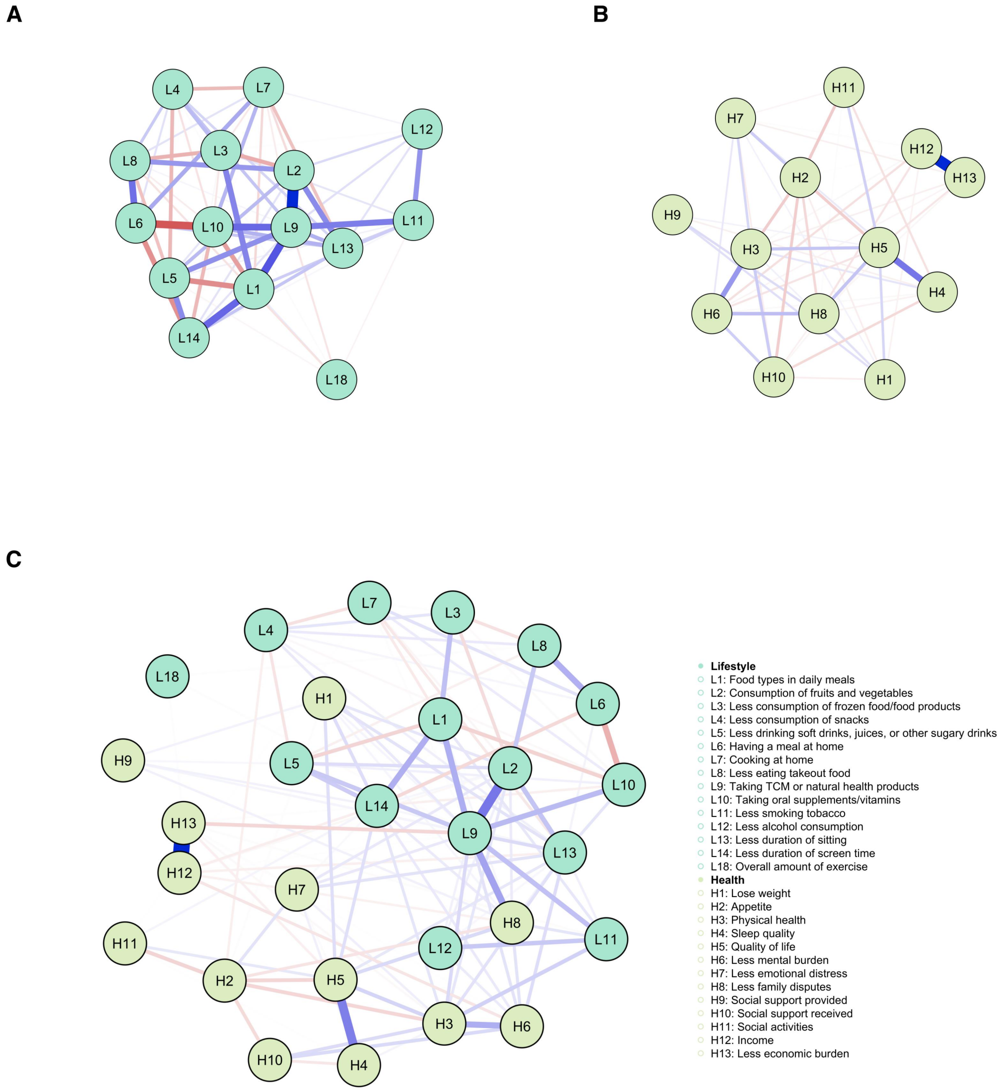

## (B). Brazil

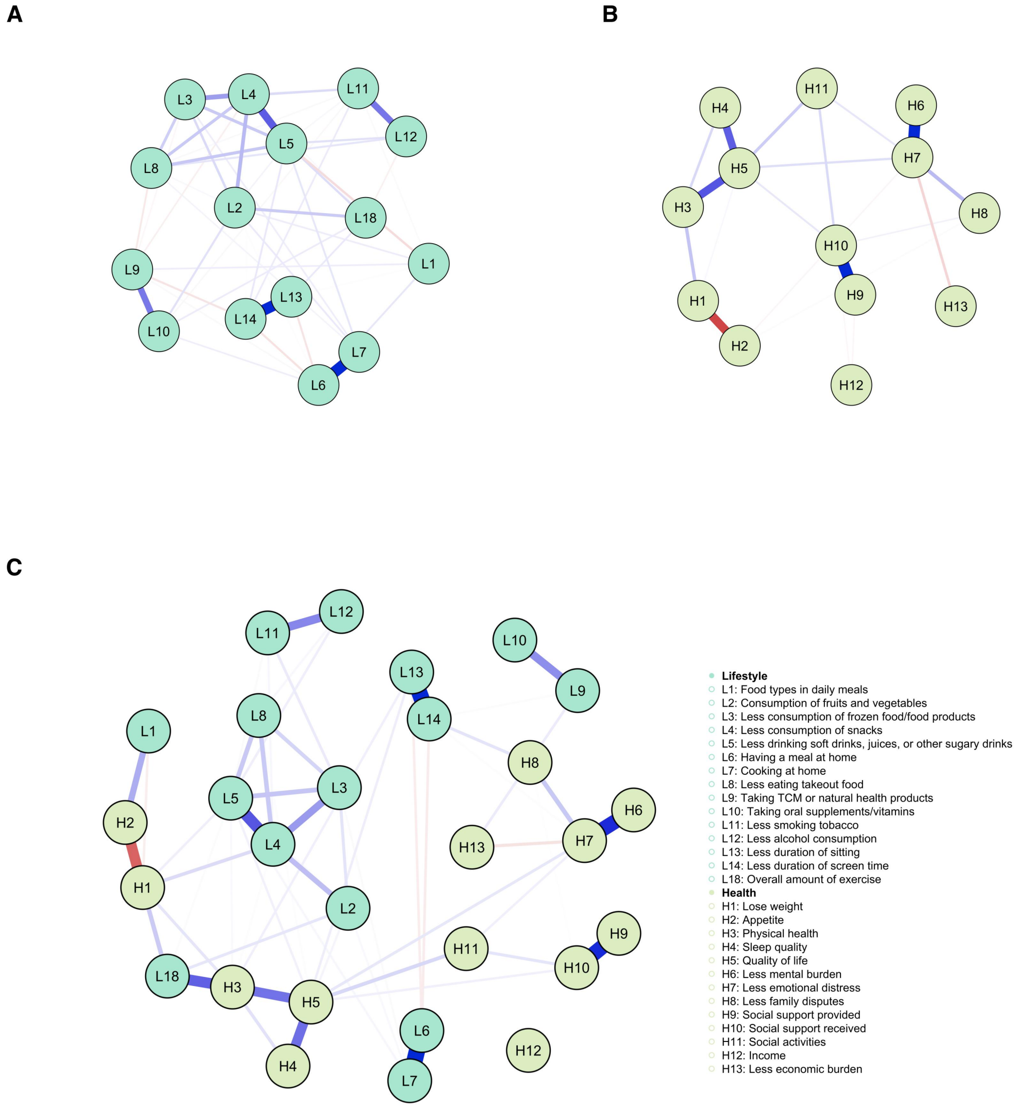
A
B
C
Lifestyle
L1: Food types in daily meals
L2: Consumption of fruits and vegetables
L3: Less consumption of frozen food/food products
L4: Less consumption of snacks
L5: Less drinking soft drinks, juices, or other sugary drinks
L6: Having a meal at home
L7: Cooking at home
L8: Less eating takeout food
L9: Taking TCM or natural health products
L10: Taking oral supplements/vitamins
L11: Less smoking tobacco
L12: Less alcohol consumption
L13: Less duration of sitting
L14: Less duration of screen time
L18: Overall amount of exercise
Health
H1: Lose weight
H2: Appetite
H3: Physical health
H4: Sleep quality
H5: Quality of life
H6: Less mental burden
H7: Less emotional distress
H8: Less family disputes
H9: Social support provided
H10: Social support received
H11: Social activities
H12: Income
H13: Less economic burden

## (C). Burundi

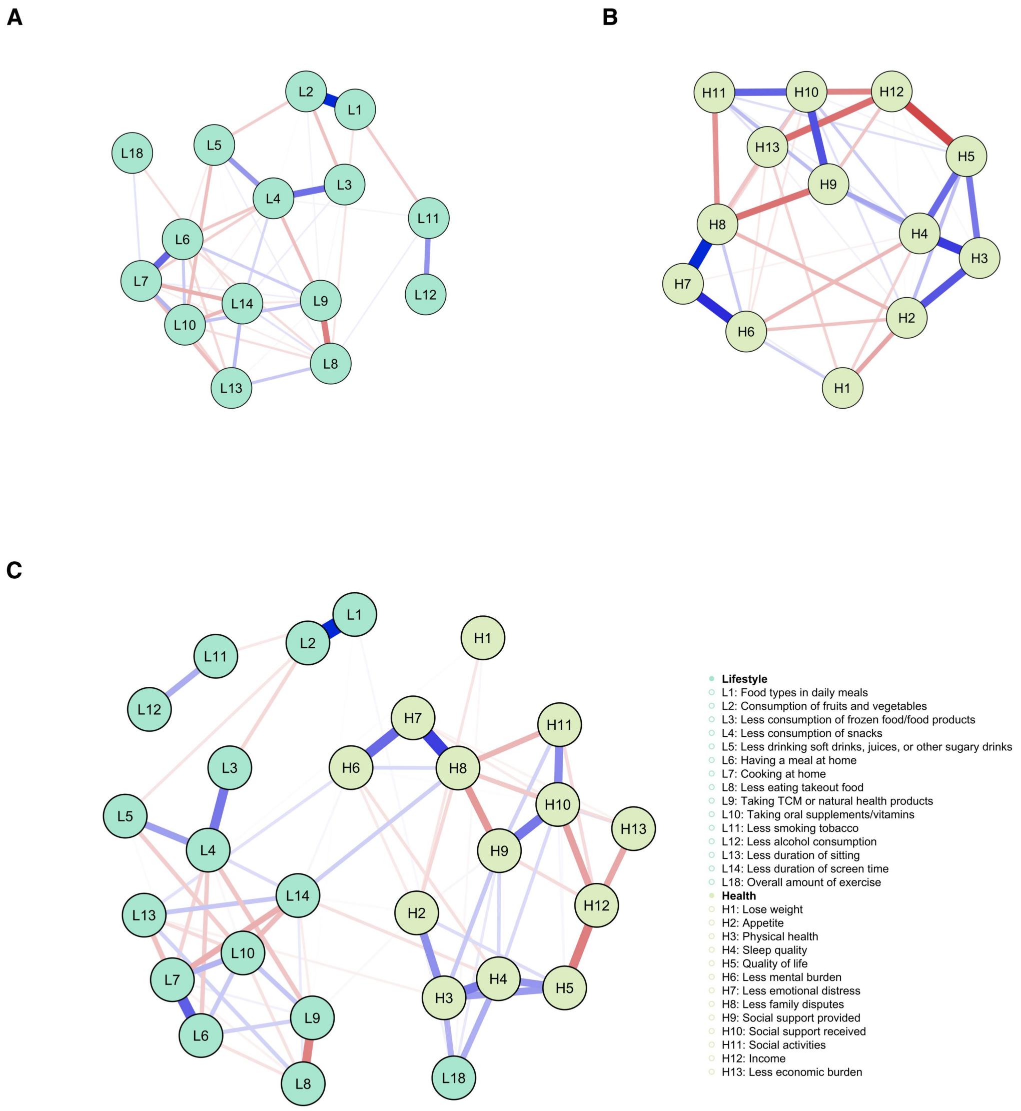

A
B
C
Lifestyle
L1: Food types in daily meals
L2: Consumption of fruits and vegetables
L3: Less consumption of frozen food/food products
L4: Less consumption of snacks
L5: Less drinking soft drinks, juices, or other sugary drinks
L6: Having a meal at home
L7: Cooking at home
L8: Less eating takeout food
L9: Taking TCM or natural health products
L10: Taking oral supplements/vitamins
L11: Less smoking tobacco
L12: Less alcohol consumption
L13: Less duration of sitting
L14: Less duration of screen time
L18: Overall amount of exercise
Health
H1: Lose weight
H2: Appetite
H3: Physical health
H4: Sleep quality
H5: Quality of life
H6: Less mental burden
H7: Less emotional distress
H8: Less family disputes
H9: Social support provided
H10: Social support received
H11: Social activities
H12: Income
H13: Less economic burden

## (D). Canada

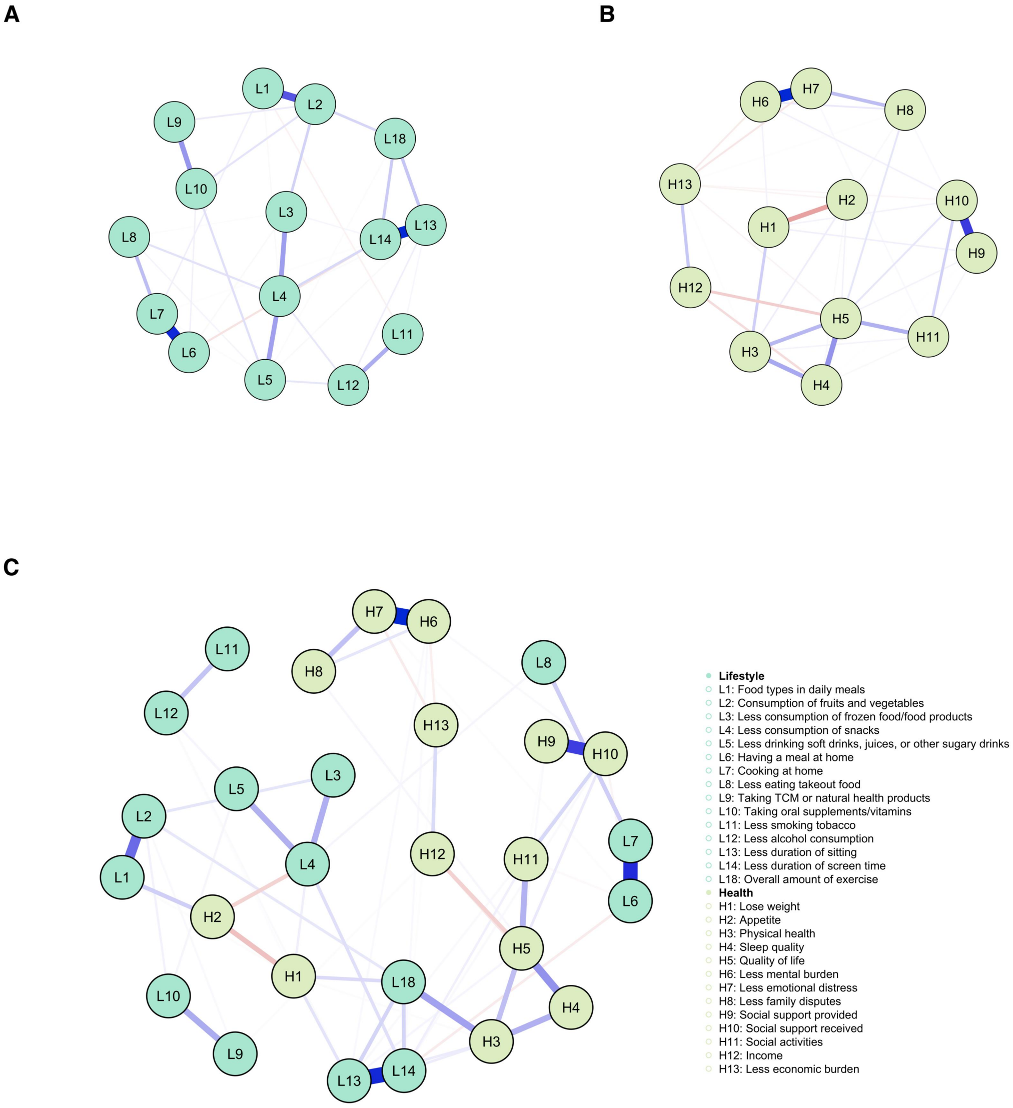
A
B
C
Lifestyle
L1: Food types in daily meals
L2: Consumption of fruits and vegetables
L3: Less consumption of frozen food/food products
L4: Less consumption of snacks
L5: Less drinking soft drinks, juices, or other sugary drinks
L6: Having a meal at home
L7: Cooking at home
L8: Less eating takeout food
L9: Taking TCM or natural health products
L10: Taking oral supplements/vitamins
L11: Less smoking tobacco
L12: Less alcohol consumption
L13: Less duration of sitting
L14: Less duration of screen time
L18: Overall amount of exercise
Health
H1: Lose weight
H2: Appetite
H3: Physical health
H4: Sleep quality
H5: Quality of life
H6: Less mental burden
H7: Less emotional distress
H8: Less family disputes
H9: Social support provided
H10: Social support received
H11: Social activities
H12: Income
H13: Less economic burden

## (E). Chile

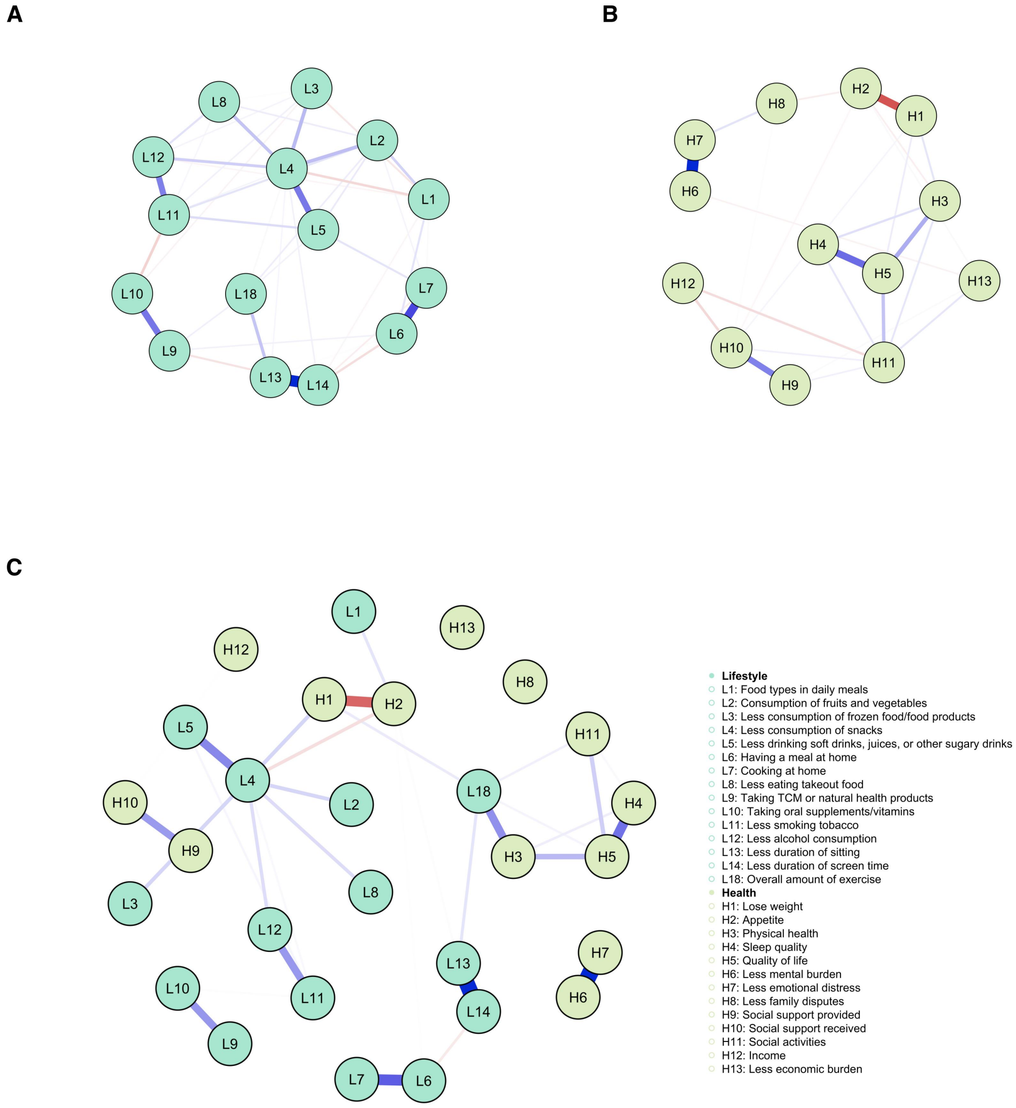
A
B
C
Lifestyle
L1: Food types in daily meals
L2: Consumption of fruits and vegetables
L3: Less consumption of frozen food/food products
L4: Less consumption of snacks
L5: Less drinking soft drinks, juices, or other sugary drinks
L6: Having a meal at home
L7: Cooking at home
L8: Less eating takeout food
L9: Taking TCM or natural health products
L10: Taking oral supplements/vitamins
L11: Less smoking tobacco
L12: Less alcohol consumption
L13: Less duration of sitting
L14: Less duration of screen time
L18: Overall amount of exercise
Health
H1: Lose weight
H2: Appetite
H3: Physical health
H4: Sleep quality
H5: Quality of life
H6: Less mental burden
H7: Less emotional distress
H8: Less family disputes
H9: Social support provided
H10: Social support received
H11: Social activities
H12: Income
H13: Less economic burden

## (F). Egypt

**A**

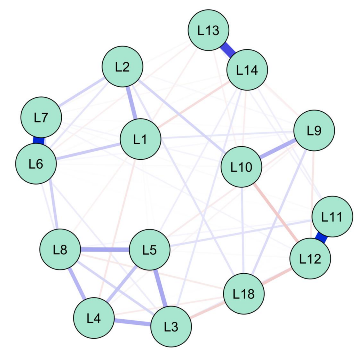

**B**

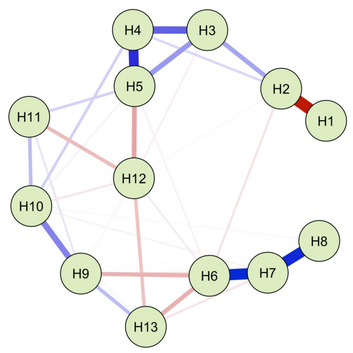

**C**

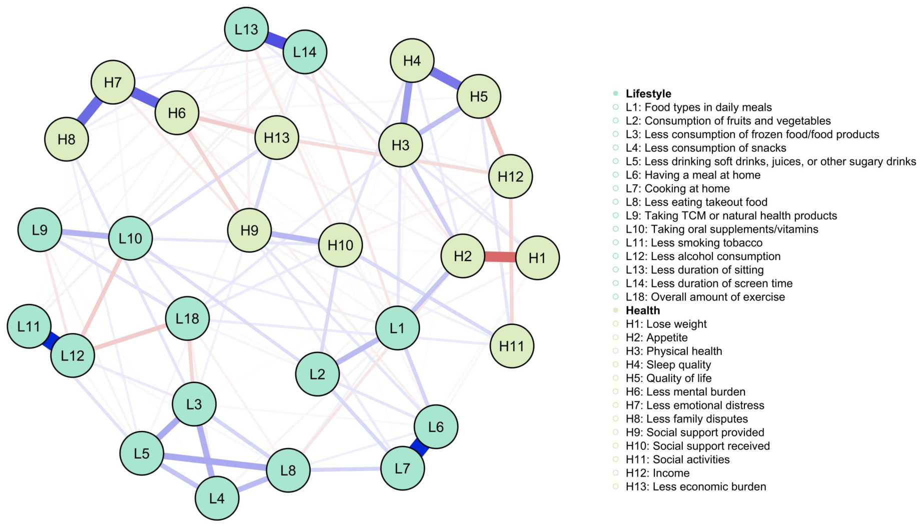
Lifestyle
L1: Food types in daily meals
L2: Consumption of fruits and vegetables
L3: Less consumption of frozen food/food products
L4: Less consumption of snacks
L5: Less drinking soft drinks, juices, or other sugary drinks
L6: Having a meal at home
L7: Cooking at home
L8: Less eating takeout food
L9: Taking TCM or natural health products
L10: Taking oral supplements/vitamins
L11: Less smoking tobacco
L12: Less alcohol consumption
L13: Less duration of sitting
L14: Less duration of screen time
L18: Overall amount of exercise
Health
H1: Lose weight
H2: Appetite
H3: Physical health
H4: Sleep quality
H5: Quality of life
H6: Less mental burden
H7: Less emotional distress
H8: Less family disputes
H9: Social support provided
H10: Social support received
H11: Social activities
H12: Income
H13: Less economic burden

## (G). Guatemala

A

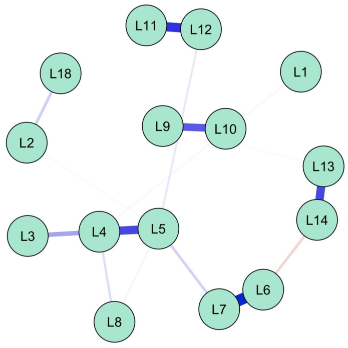


B

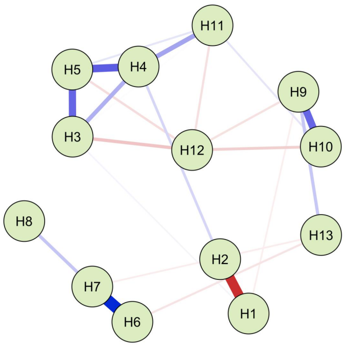


C

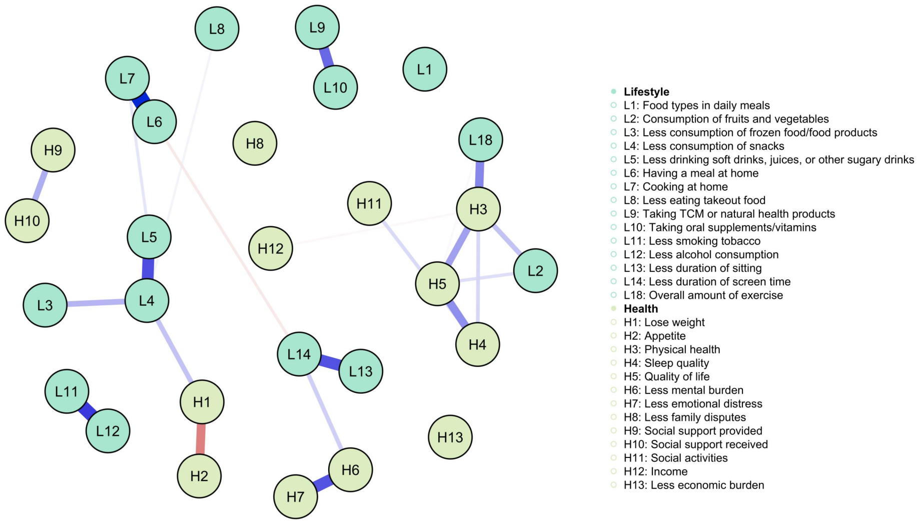

## (H). Hong Kong

**A**

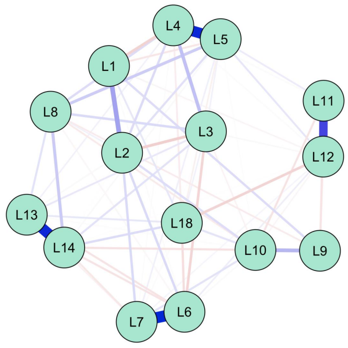

**B**

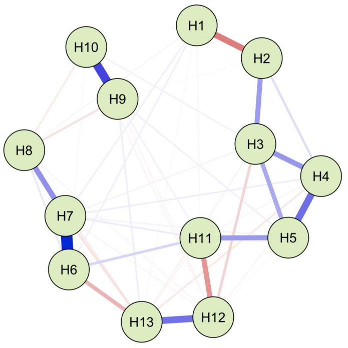

**C**

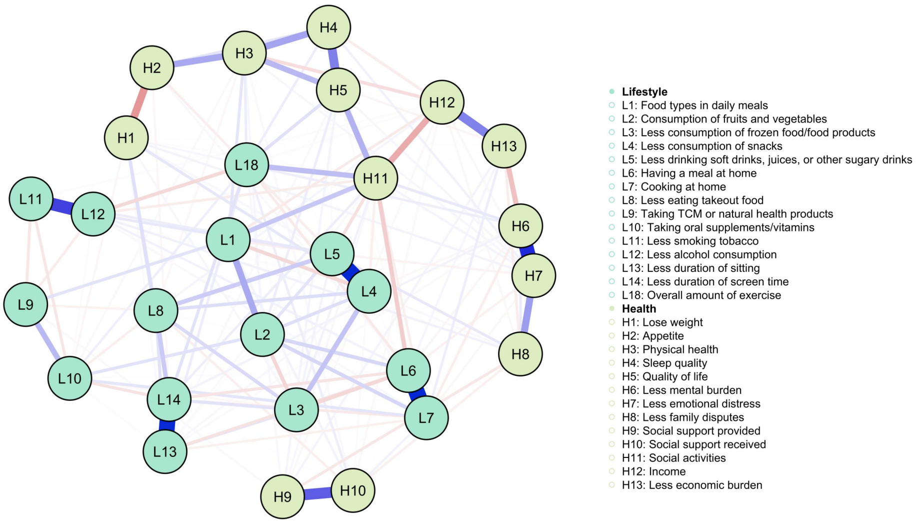

## (I). India

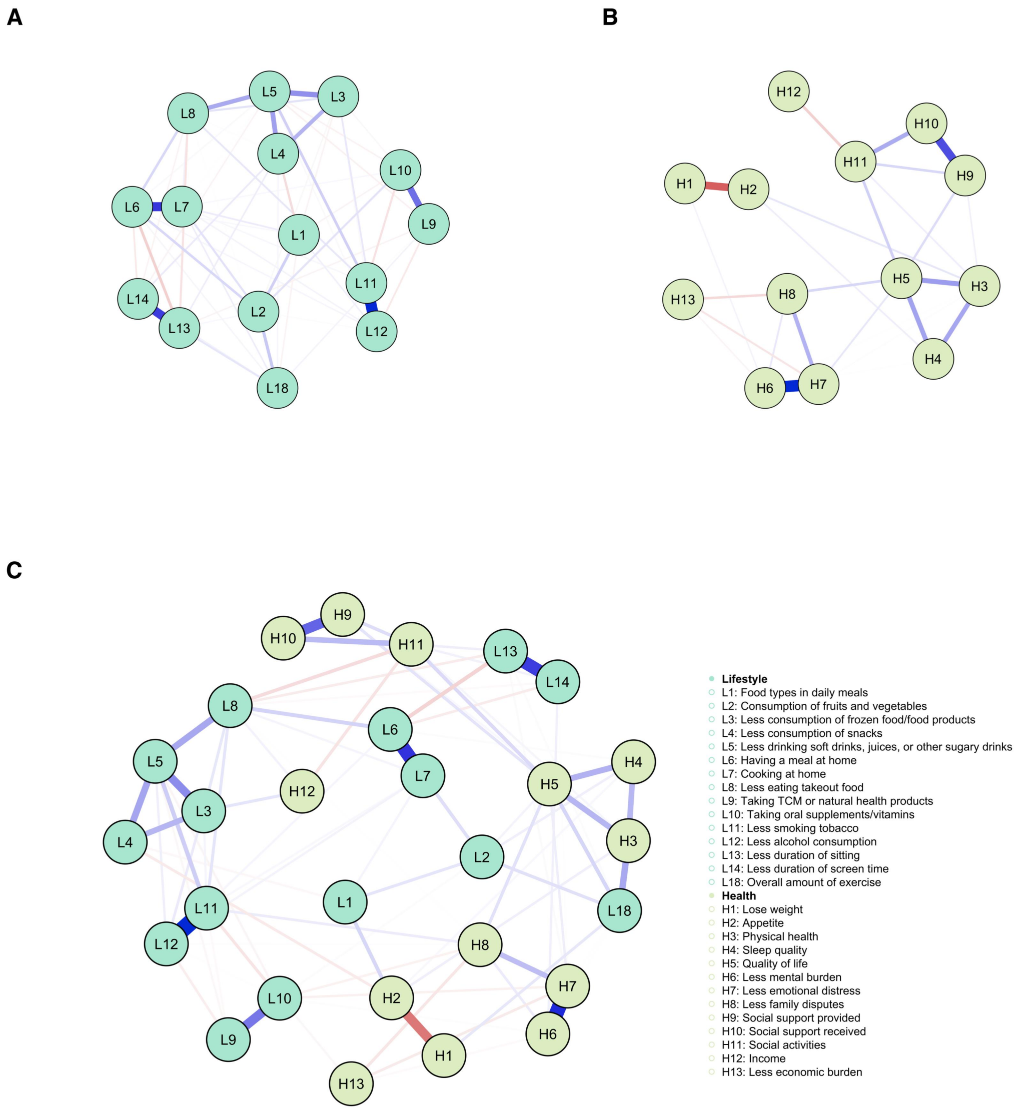
A
L5
L3
L8
L4
L10
L6
L7
L9
L1
L11
L14
L2
L13
L12
L18
B
H12
H10
H11
H9
H1
H2
H5
H3
H13
H8
H4
H6
H7
C
H9
H10
H11
L13
L14
L8
L6
L5
L7
H5
H4
H12
L3
L4
H3
L2
L1
L11
L18
L12
H8
H7
L10
H2
L9
H6
H1
H13
Lifestyle
L1: Food types in daily meals
L2: Consumption of fruits and vegetables
L3: Less consumption of frozen food/food products
L4: Less consumption of snacks
L5: Less drinking soft drinks, juices, or other sugary drinks
L6: Having a meal at home
L7: Cooking at home
L8: Less eating takeout food
L9: Taking TCM or natural health products
L10: Taking oral supplements/vitamins
L11: Less smoking tobacco
L12: Less alcohol consumption
L13: Less duration of sitting
L14: Less duration of screen time
L18: Overall amount of exercise
Health
H1: Lose weight
H2: Appetite
H3: Physical health
H4: Sleep quality
H5: Quality of life
H6: Less mental burden
H7: Less emotional distress
H8: Less family disputes
H9: Social support provided
H10: Social support received
H11: Social activities
H12: Income
H13: Less economic burden

## (J). Indonesia

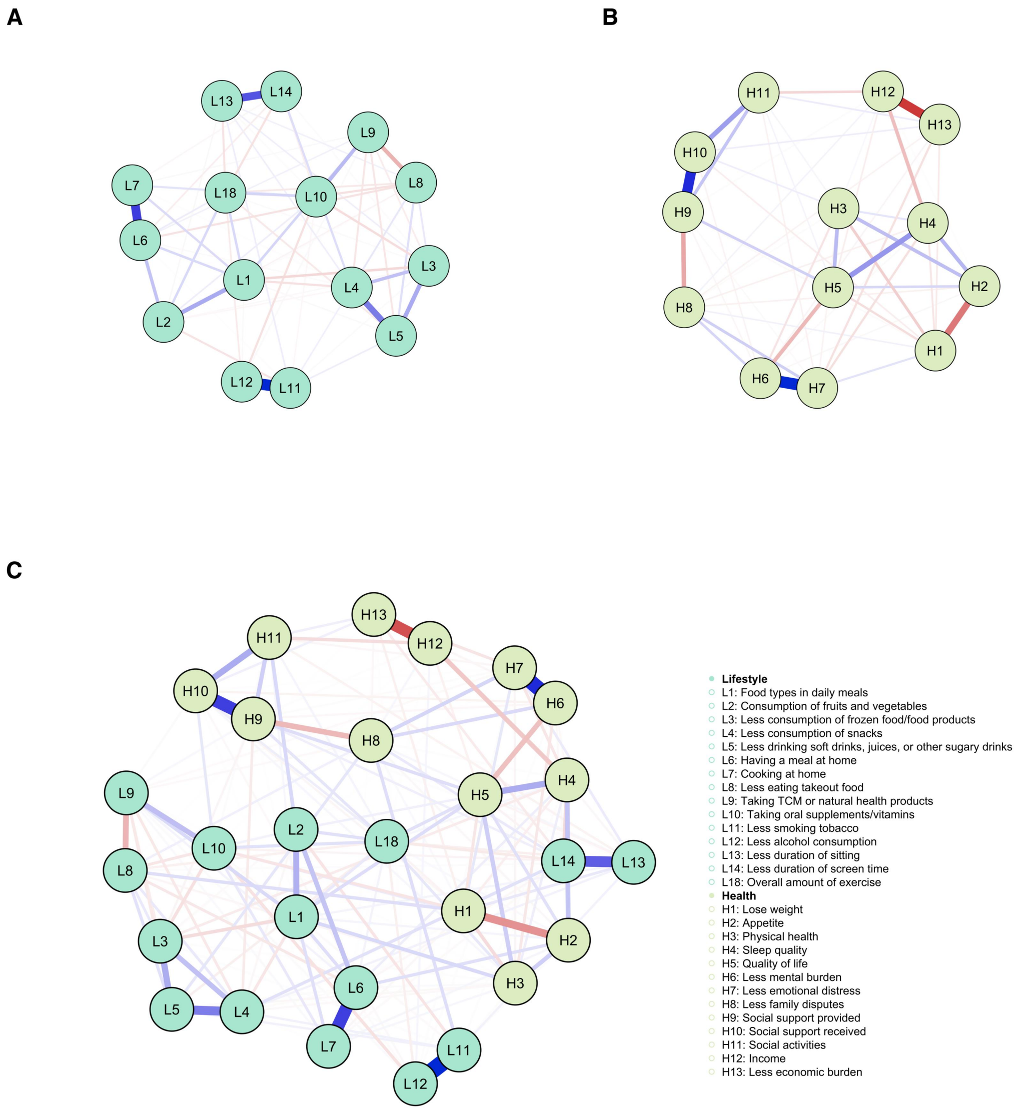
A
B
C
Lifestyle
L1: Food types in daily meals
L2: Consumption of fruits and vegetables
L3: Less consumption of frozen food/food products
L4: Less consumption of snacks
L5: Less drinking soft drinks, juices, or other sugary drinks
L6: Having a meal at home
L7: Cooking at home
L8: Less eating takeout food
L9: Taking TCM or natural health products
L10: Taking oral supplements/vitamins
L11: Less smoking tobacco
L12: Less alcohol consumption
L13: Less duration of sitting
L14: Less duration of screen time
L18: Overall amount of exercise
Health
H1: Lose weight
H2: Appetite
H3: Physical health
H4: Sleep quality
H5: Quality of life
H6: Less mental burden
H7: Less emotional distress
H8: Less family disputes
H9: Social support provided
H10: Social support received
H11: Social activities
H12: Income
H13: Less economic burden

## (K). Italy

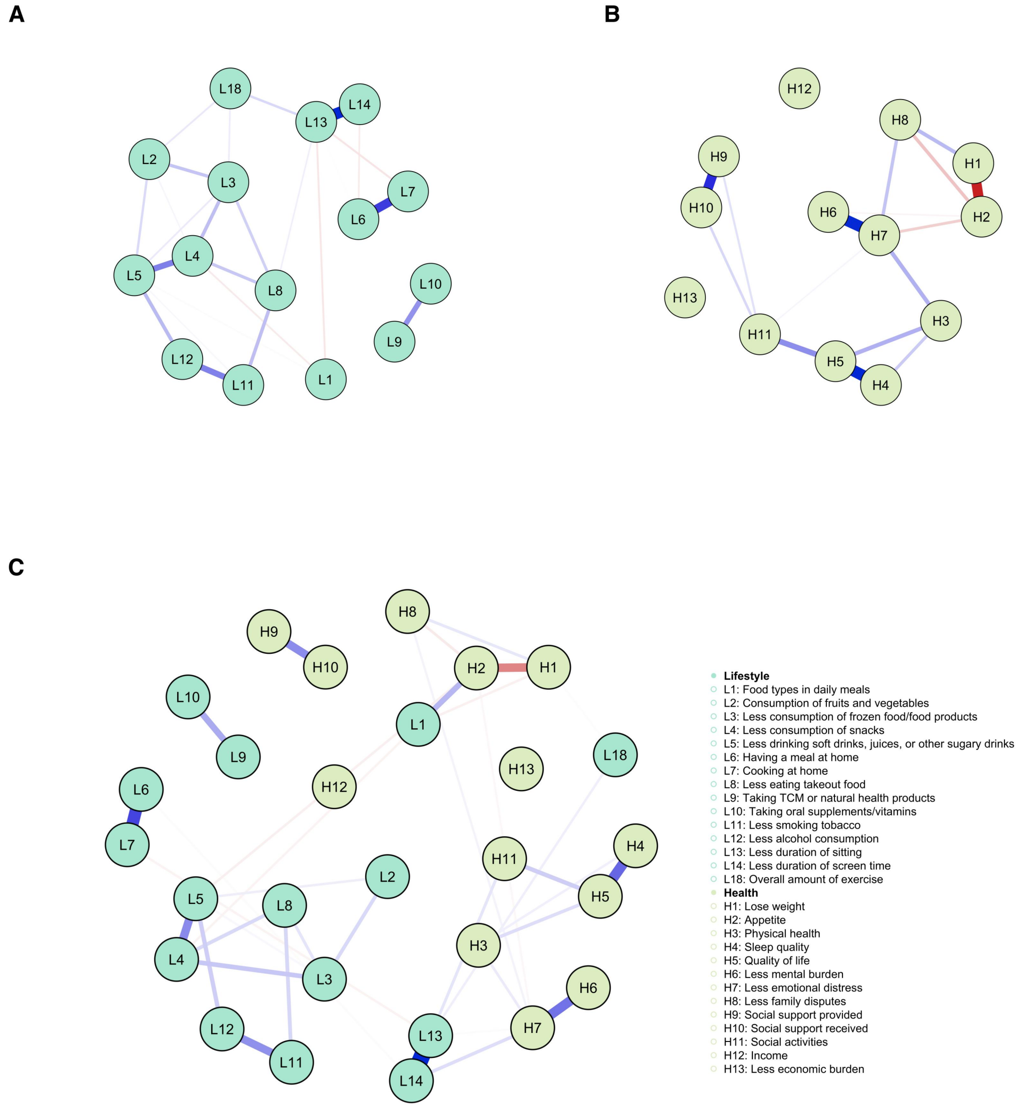

A
B
C
Lifestyle
L1: Food types in daily meals
L2: Consumption of fruits and vegetables
L3: Less consumption of frozen food/food products
L4: Less consumption of snacks
L5: Less drinking soft drinks, juices, or other sugary drinks
L6: Having a meal at home
L7: Cooking at home
L8: Less eating takeout food
L9: Taking TCM or natural health products
L10: Taking oral supplements/vitamins
L11: Less smoking tobacco
L12: Less alcohol consumption
L13: Less duration of sitting
L14: Less duration of screen time
L18: Overall amount of exercise
Health
H1: Lose weight
H2: Appetite
H3: Physical health
H4: Sleep quality
H5: Quality of life
H6: Less mental burden
H7: Less emotional distress
H8: Less family disputes
H9: Social support provided
H10: Social support received
H11: Social activities
H12: Income
H13: Less economic burden

## (L). Lebanon

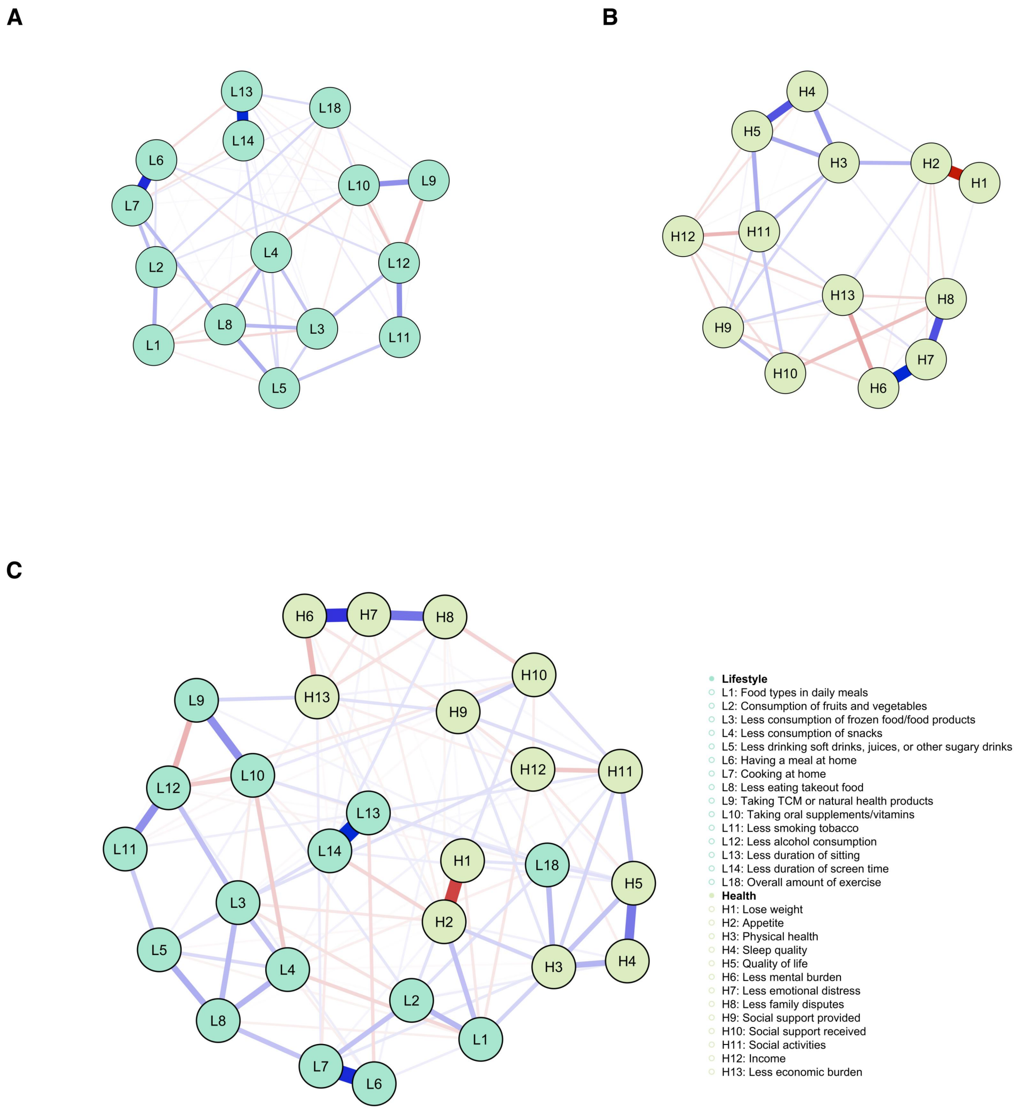
A
B
C
L1
L2
L3
L4
L5
L6
L7
L8
L9
L10
L11
L12
L13
L14
L18
H1
H2
H3
H4
H5
H6
H7
H8
H9
H10
H11
H12
H13
Lifestyle
L1: Food types in daily meals
L2: Consumption of fruits and vegetables
L3: Less consumption of frozen food/food products
L4: Less consumption of snacks
L5: Less drinking soft drinks, juices, or other sugary drinks
L6: Having a meal at home
L7: Cooking at home
L8: Less eating takeout food
L9: Taking TCM or natural health products
L10: Taking oral supplements/vitamins
L11: Less smoking tobacco
L12: Less alcohol consumption
L13: Less duration of sitting
L14: Less duration of screen time
L18: Overall amount of exercise
Health
H1: Lose weight
H2: Appetite
H3: Physical health
H4: Sleep quality
H5: Quality of life
H6: Less mental burden
H7: Less emotional distress
H8: Less family disputes
H9: Social support provided
H10: Social support received
H11: Social activities
H12: Income
H13: Less economic burden

## (M). Libya

A

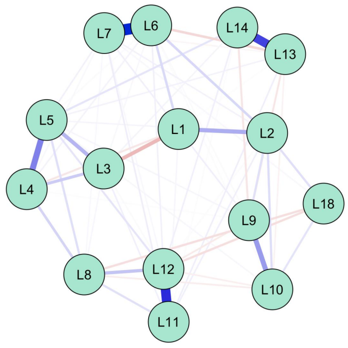

L7
L6
L14
L13
L5
L1
L2
L3
L4
L18
L9
L8
L12
L10
L11


B

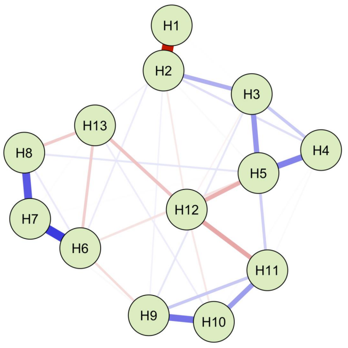

H1
H2
H3
H13
H4
H8
H5
H12
H7
H6
H11
H9
H10


C

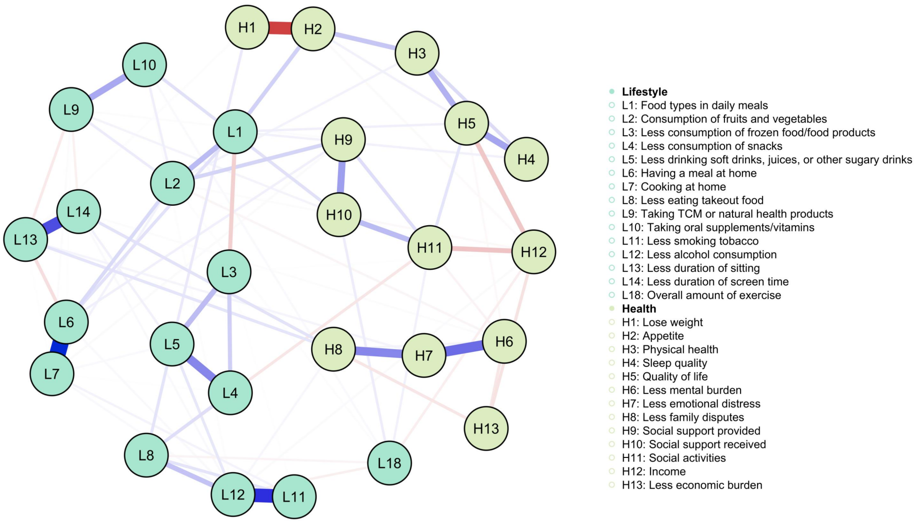

Lifestyle
L1: Food types in daily meals
L2: Consumption of fruits and vegetables
L3: Less consumption of frozen food/food products
L4: Less consumption of snacks
L5: Less drinking soft drinks, juices, or other sugary drinks
L6: Having a meal at home
L7: Cooking at home
L8: Less eating takeout food
L9: Taking TCM or natural health products
L10: Taking oral supplements/vitamins
L11: Less smoking tobacco
L12: Less alcohol consumption
L13: Less duration of sitting
L14: Less duration of screen time
L18: Overall amount of exercise
Health
H1: Lose weight
H2: Appetite
H3: Physical health
H4: Sleep quality
H5: Quality of life
H6: Less mental burden
H7: Less emotional distress
H8: Less family disputes
H9: Social support provided
H10: Social support received
H11: Social activities
H12: Income
H13: Less economic burden

## (N). Macau

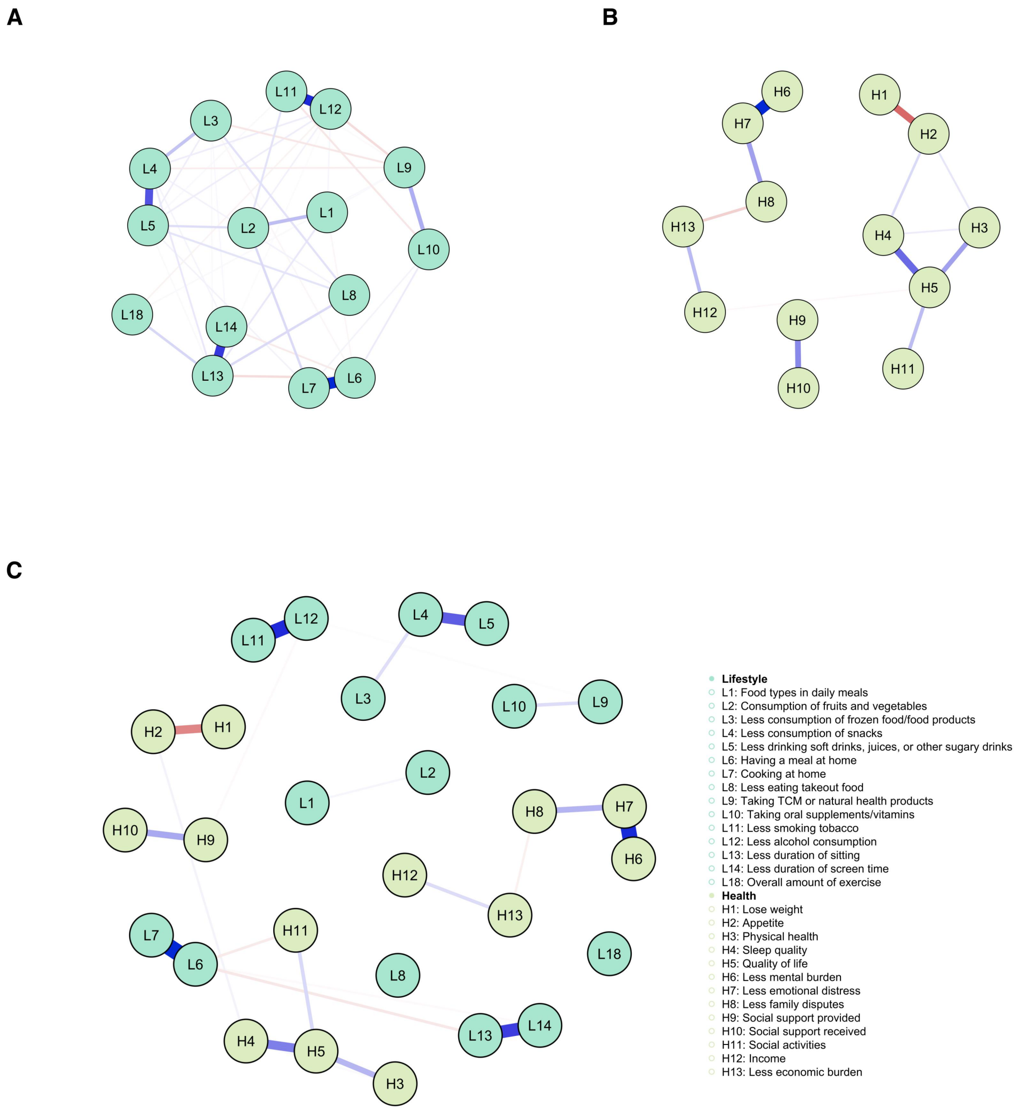

A
B
C
Lifestyle
L1: Food types in daily meals
L2: Consumption of fruits and vegetables
L3: Less consumption of frozen food/food products
L4: Less consumption of snacks
L5: Less drinking soft drinks, juices, or other sugary drinks
L6: Having a meal at home
L7: Cooking at home
L8: Less eating takeout food
L9: Taking TCM or natural health products
L10: Taking oral supplements/vitamins
L11: Less smoking tobacco
L12: Less alcohol consumption
L13: Less duration of sitting
L14: Less duration of screen time
L18: Overall amount of exercise
Health
H1: Lose weight
H2: Appetite
H3: Physical health
H4: Sleep quality
H5: Quality of life
H6: Less mental burden
H7: Less emotional distress
H8: Less family disputes
H9: Social support provided
H10: Social support received
H11: Social activities
H12: Income
H13: Less economic burden

## (O). Mainland China

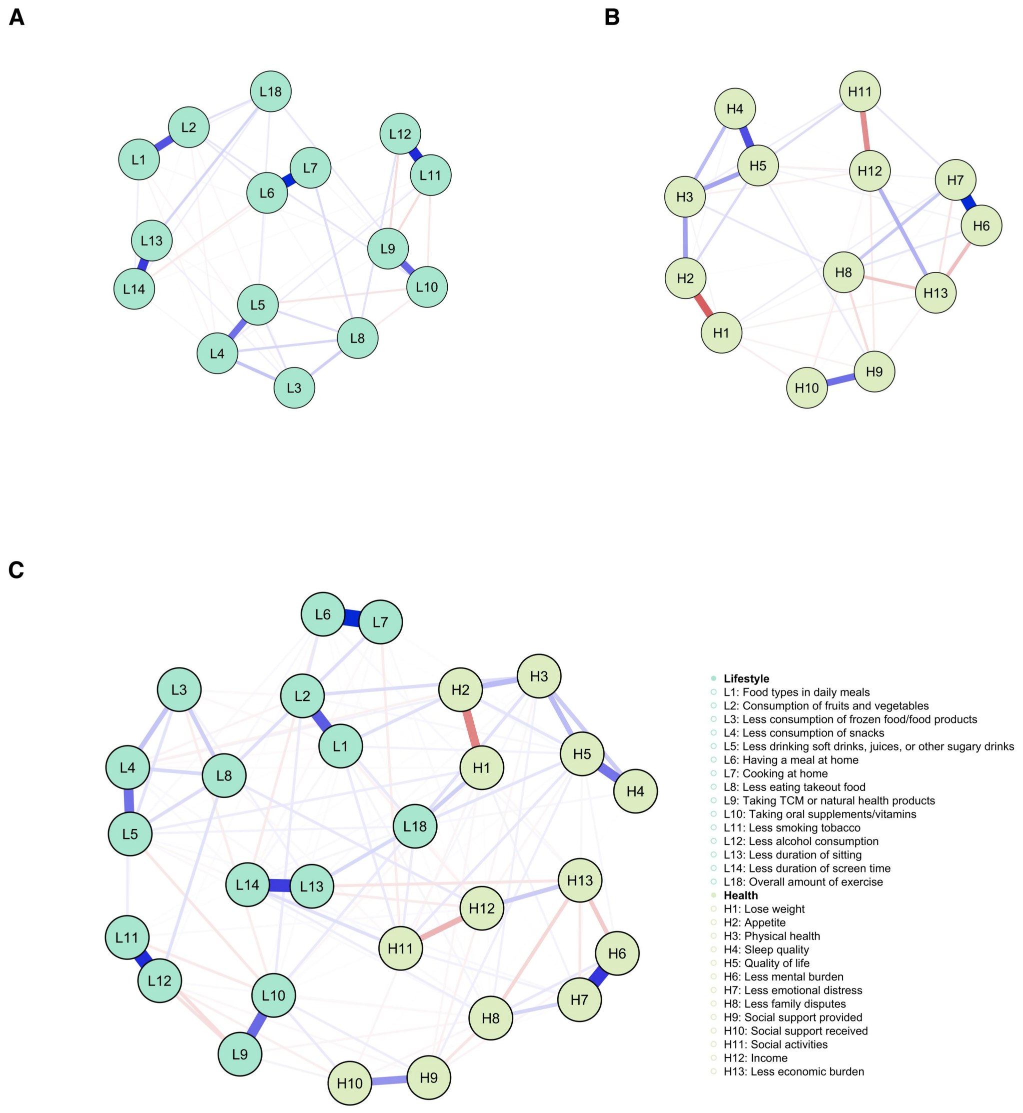

## (P). Malaysia

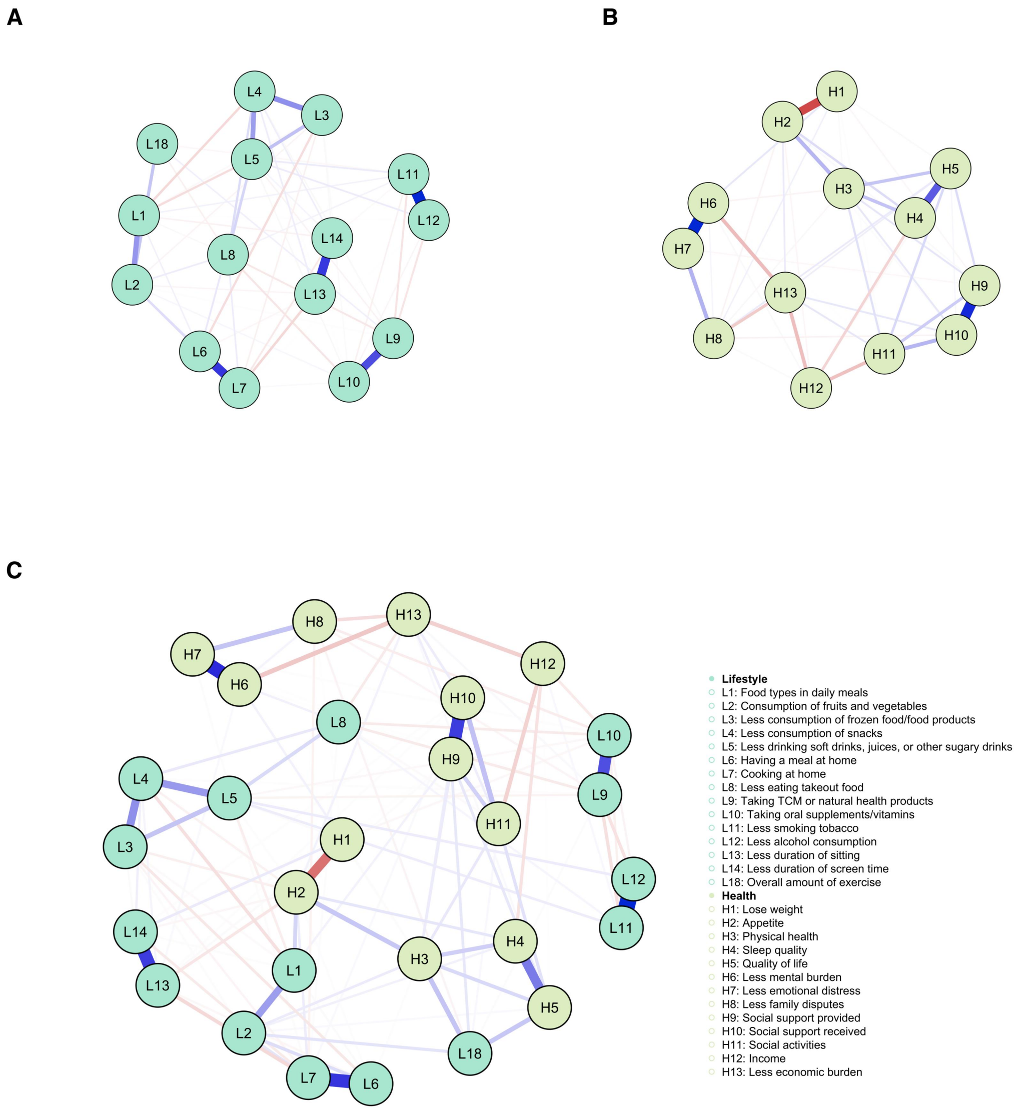
A
B
C
Lifestyle
L1: Food types in daily meals
L2: Consumption of fruits and vegetables
L3: Less consumption of frozen food/food products
L4: Less consumption of snacks
L5: Less drinking soft drinks, juices, or other sugary drinks
L6: Having a meal at home
L7: Cooking at home
L8: Less eating takeout food
L9: Taking TCM or natural health products
L10: Taking oral supplements/vitamins
L11: Less smoking tobacco
L12: Less alcohol consumption
L13: Less duration of sitting
L14: Less duration of screen time
L18: Overall amount of exercise
Health
H1: Lose weight
H2: Appetite
H3: Physical health
H4: Sleep quality
H5: Quality of life
H6: Less mental burden
H7: Less emotional distress
H8: Less family disputes
H9: Social support provided
H10: Social support received
H11: Social activities
H12: Income
H13: Less economic burden

## (Q). Mexico

A

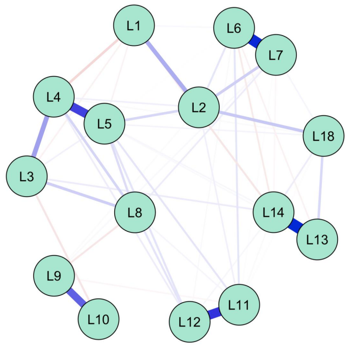
L1
L6
L7
L4
L5
L2
L18
L3
L8
L14
L13
L9
L10
L12
L11

B

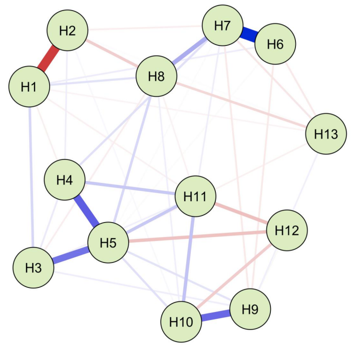
H2
H7
H6
H1
H8
H13
H4
H11
H12
H5
H3
H10
H9

C

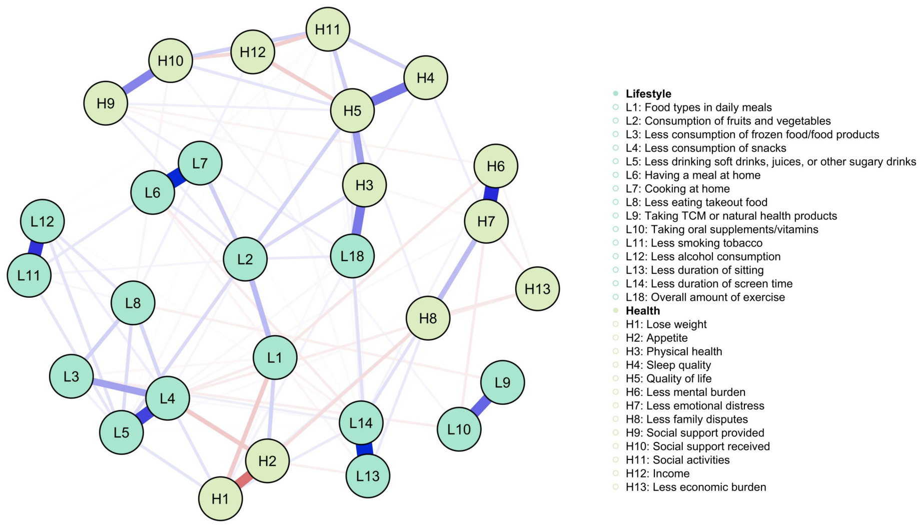
Lifestyle
L1: Food types in daily meals
L2: Consumption of fruits and vegetables
L3: Less consumption of frozen food/food products
L4: Less consumption of snacks
L5: Less drinking soft drinks, juices, or other sugary drinks
L6: Having a meal at home
L7: Cooking at home
L8: Less eating takeout food
L9: Taking TCM or natural health products
L10: Taking oral supplements/vitamins
L11: Less smoking tobacco
L12: Less alcohol consumption
L13: Less duration of sitting
L14: Less duration of screen time
L18: Overall amount of exercise
Health
H1: Lose weight
H2: Appetite
H3: Physical health
H4: Sleep quality
H5: Quality of life
H6: Less mental burden
H7: Less emotional distress
H8: Less family disputes
H9: Social support provided
H10: Social support received
H11: Social activities
H12: Income
H13: Less economic burden

## (R). Nigeria

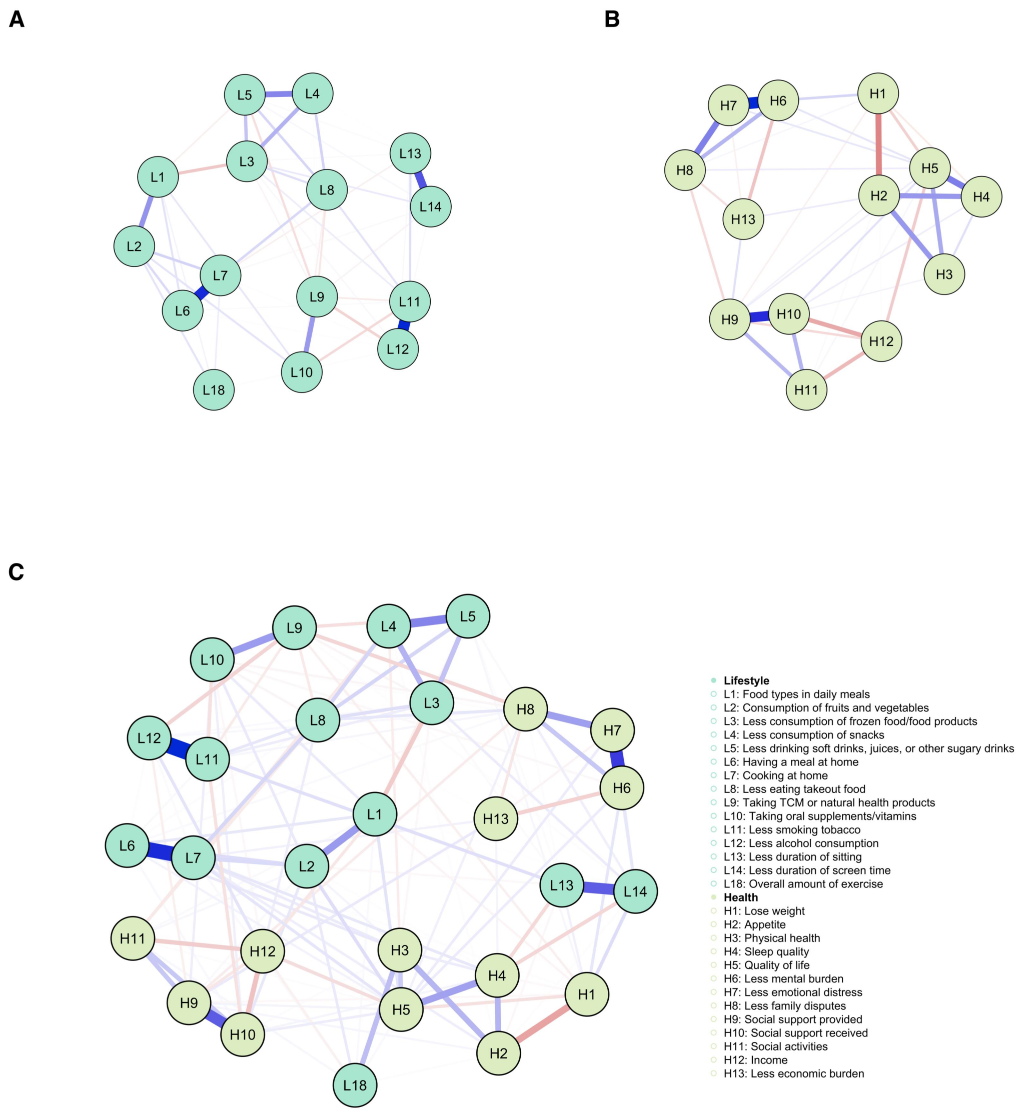
A
B
C
Lifestyle
L1: Food types in daily meals
L2: Consumption of fruits and vegetables
L3: Less consumption of frozen food/food products
L4: Less consumption of snacks
L5: Less drinking soft drinks, juices, or other sugary drinks
L6: Having a meal at home
L7: Cooking at home
L8: Less eating takeout food
L9: Taking TCM or natural health products
L10: Taking oral supplements/vitamins
L11: Less smoking tobacco
L12: Less alcohol consumption
L13: Less duration of sitting
L14: Less duration of screen time
L18: Overall amount of exercise
Health
H1: Lose weight
H2: Appetite
H3: Physical health
H4: Sleep quality
H5: Quality of life
H6: Less mental burden
H7: Less emotional distress
H8: Less family disputes
H9: Social support provided
H10: Social support received
H11: Social activities
H12: Income
H13: Less economic burden

## (S).Philippines

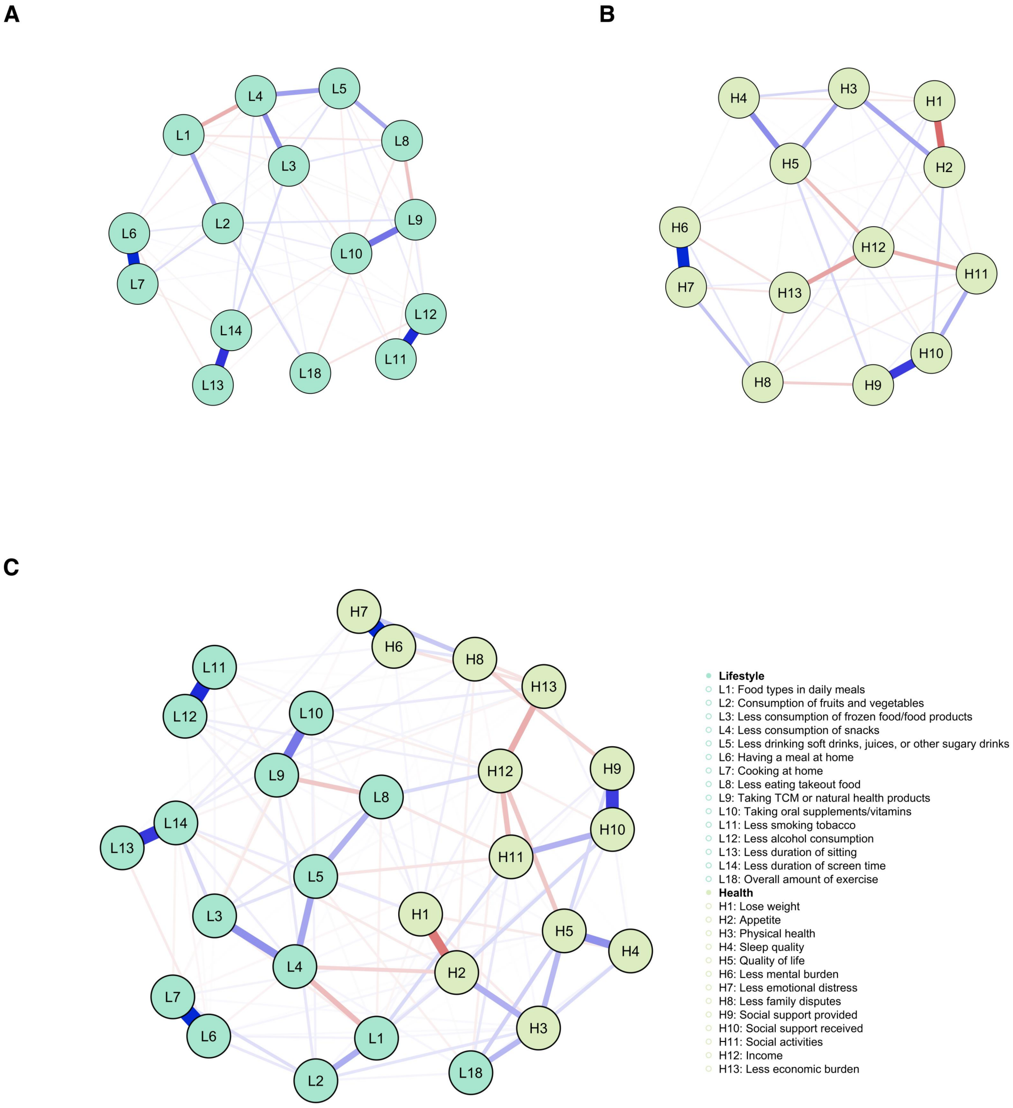

## (T). Republic of Sudan

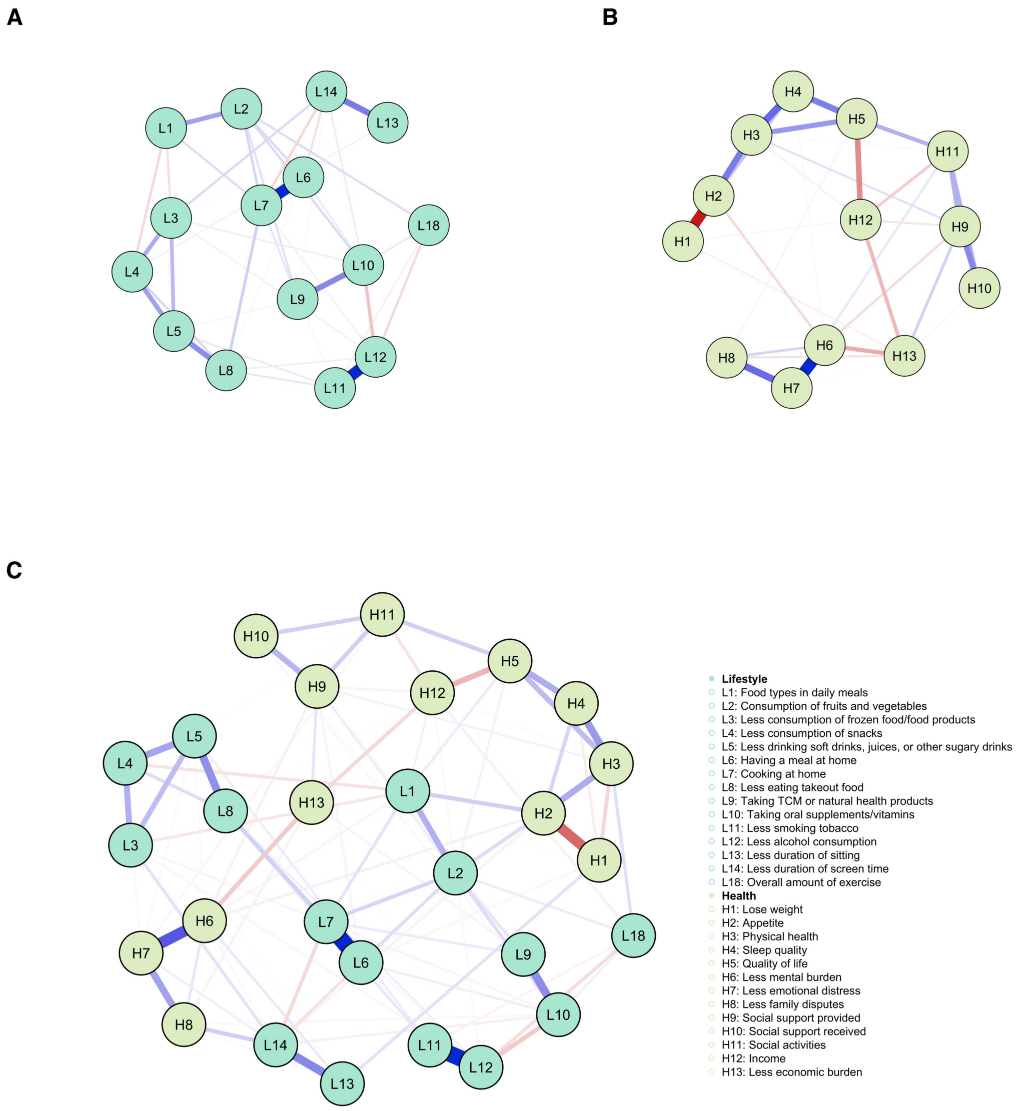

## (U). Rwanda

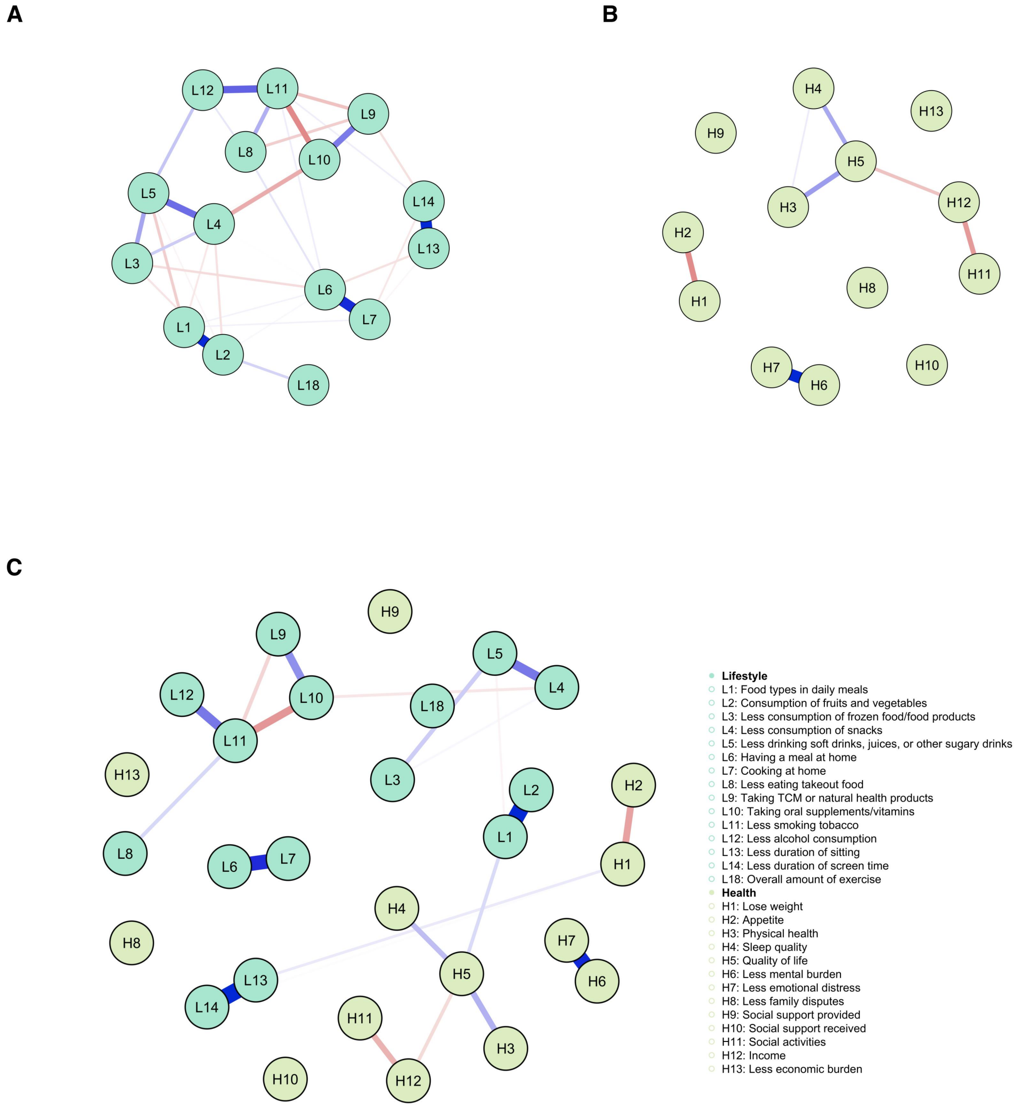

A
B
C
Lifestyle
L1: Food types in daily meals
L2: Consumption of fruits and vegetables
L3: Less consumption of frozen food/food products
L4: Less consumption of snacks
L5: Less drinking soft drinks, juices, or other sugary drinks
L6: Having a meal at home
L7: Cooking at home
L8: Less eating takeout food
L9: Taking TCM or natural health products
L10: Taking oral supplements/vitamins
L11: Less smoking tobacco
L12: Less alcohol consumption
L13: Less duration of sitting
L14: Less duration of screen time
L18: Overall amount of exercise
Health
H1: Lose weight
H2: Appetite
H3: Physical health
H4: Sleep quality
H5: Quality of life
H6: Less mental burden
H7: Less emotional distress
H8: Less family disputes
H9: Social support provided
H10: Social support received
H11: Social activities
H12: Income
H13: Less economic burden

## (V). Saudi Arabia

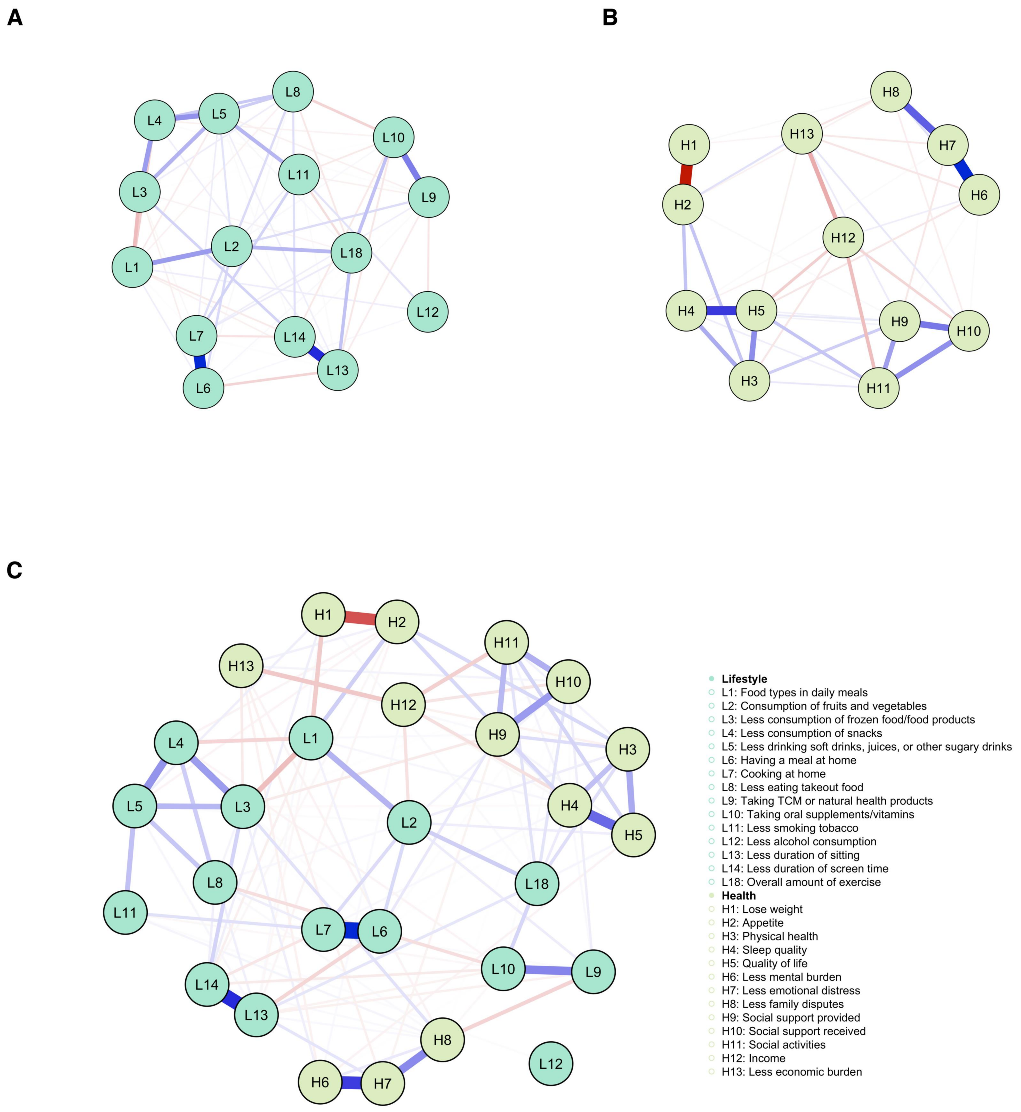

## (W). Singapore

**A**

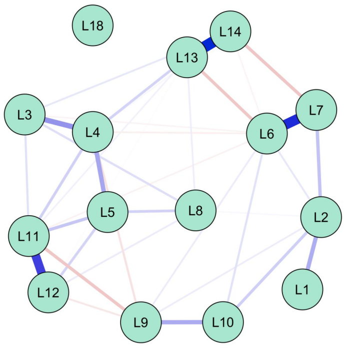


**B**

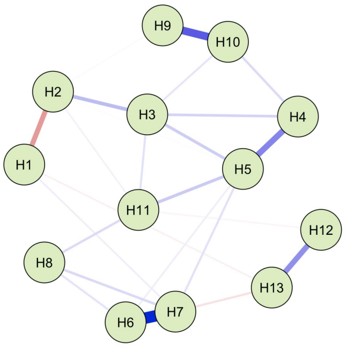


**C**

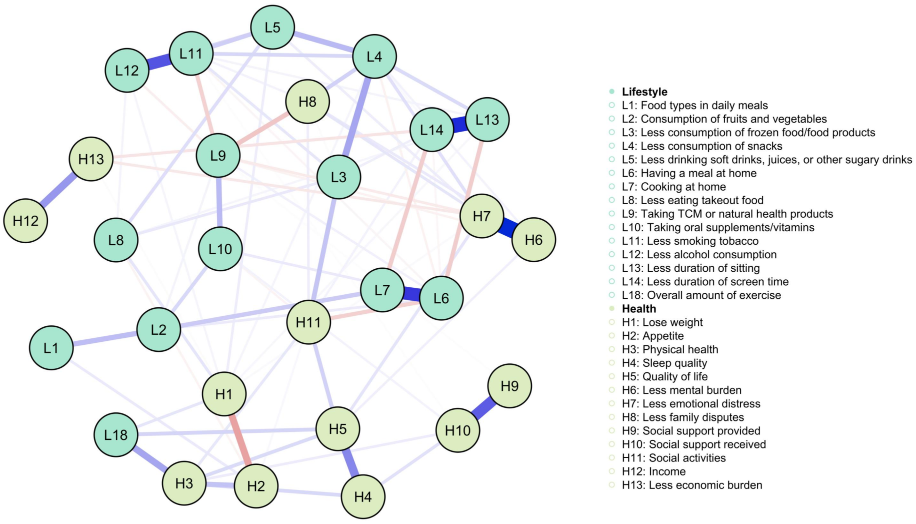

## (X). South Africa

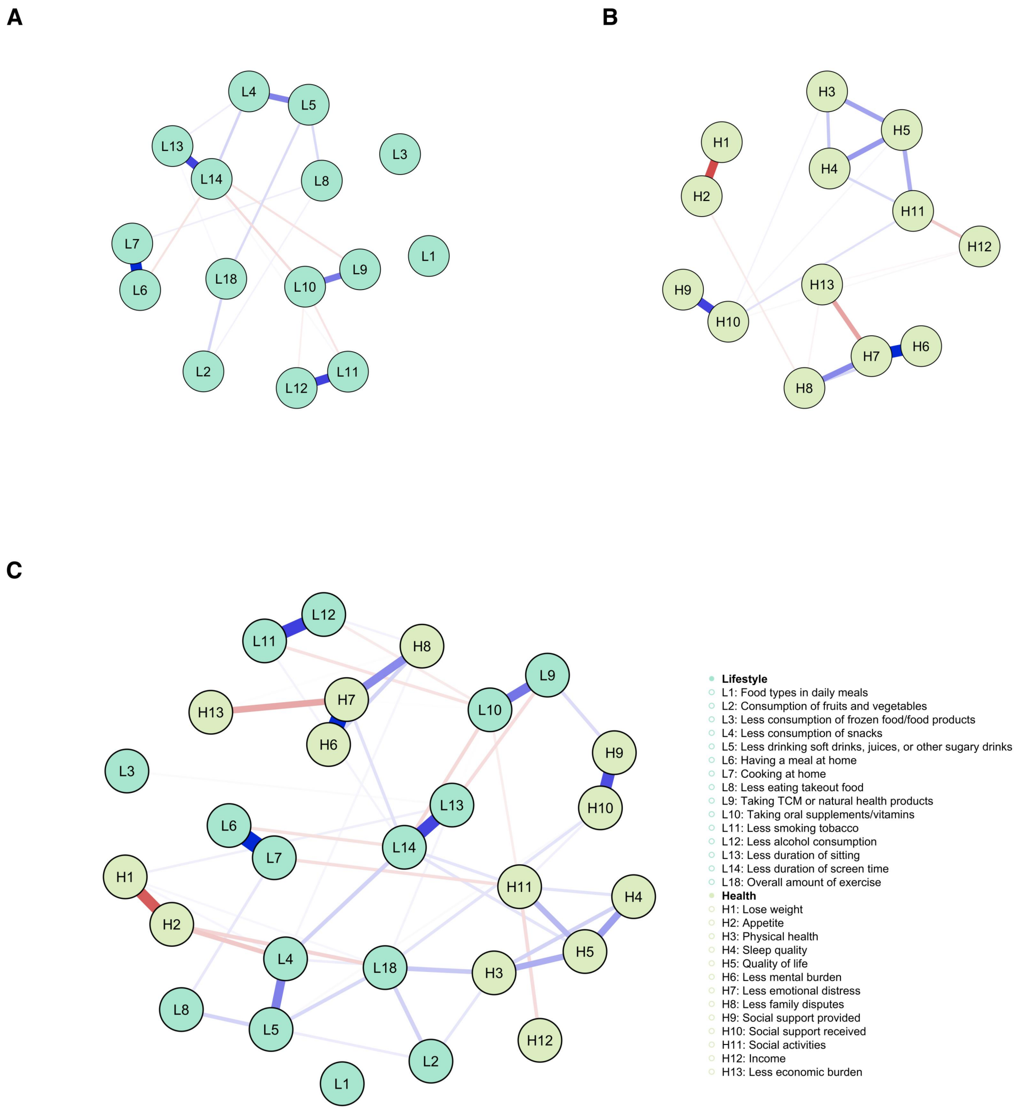

## (Y). South Korea

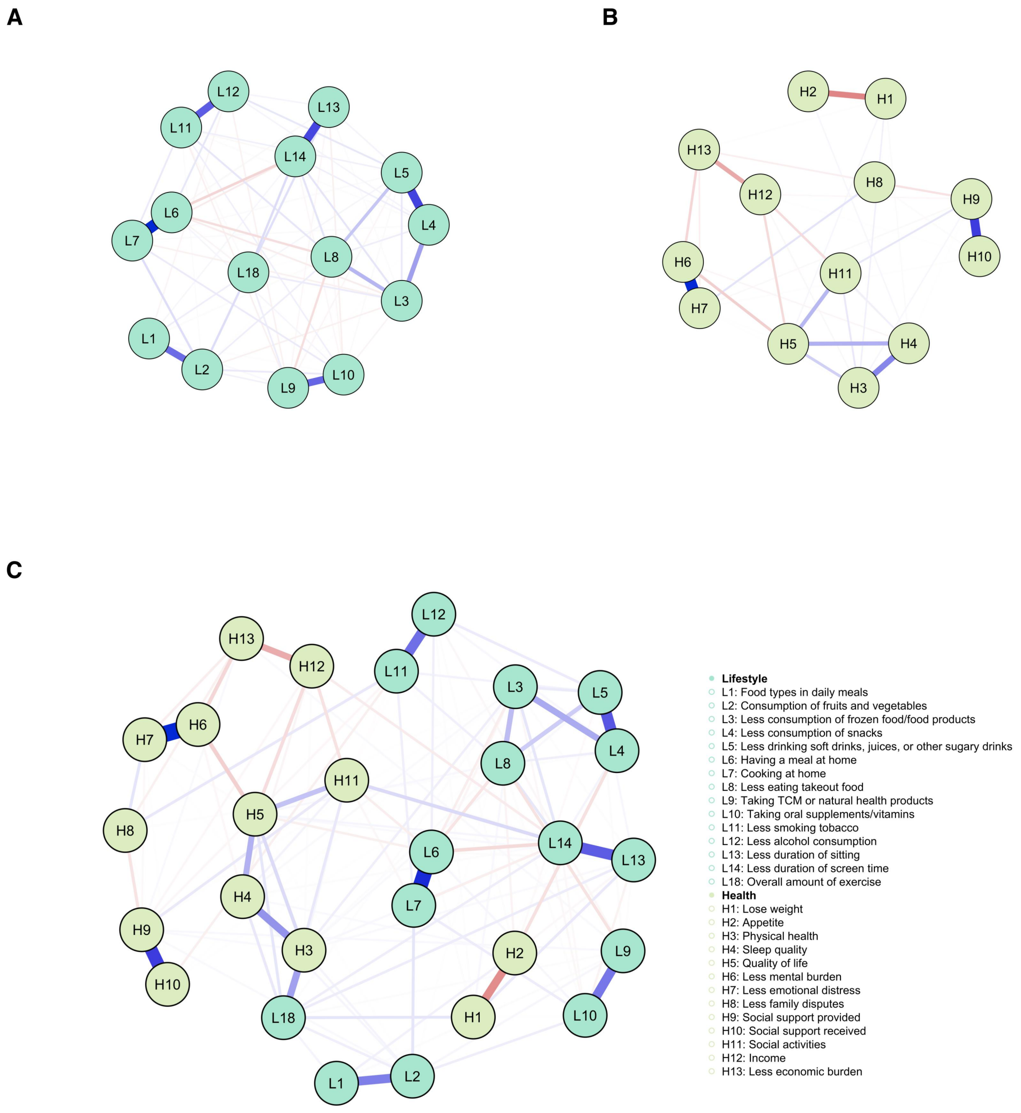

## (Z). Thailand

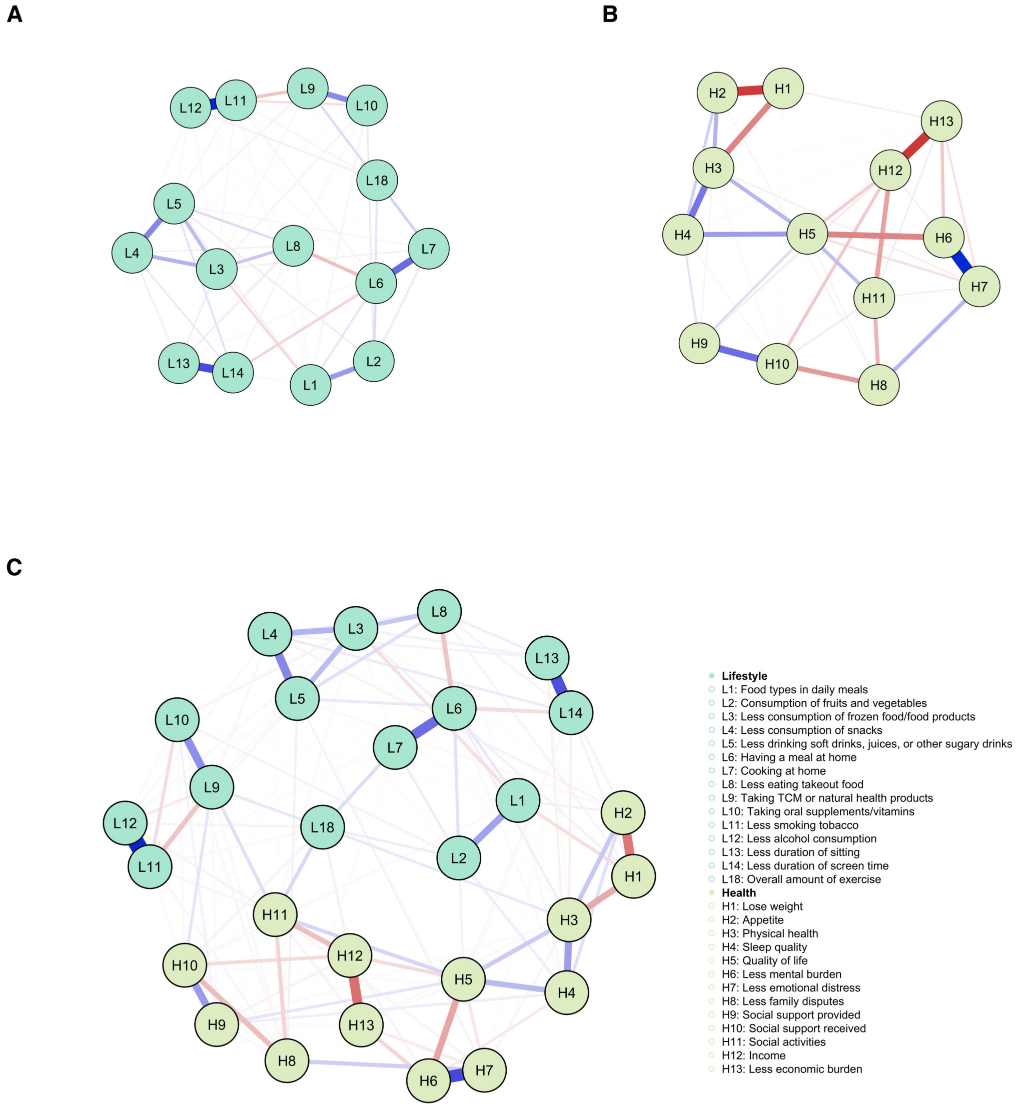
A
B
C
Lifestyle
L1: Food types in daily meals
L2: Consumption of fruits and vegetables
L3: Less consumption of frozen food/food products
L4: Less consumption of snacks
L5: Less drinking soft drinks, juices, or other sugary drinks
L6: Having a meal at home
L7: Cooking at home
L8: Less eating takeout food
L9: Taking TCM or natural health products
L10: Taking oral supplements/vitamins
L11: Less smoking tobacco
L12: Less alcohol consumption
L13: Less duration of sitting
L14: Less duration of screen time
L18: Overall amount of exercise
Health
H1: Lose weight
H2: Appetite
H3: Physical health
H4: Sleep quality
H5: Quality of life
H6: Less mental burden
H7: Less emotional distress
H8: Less family disputes
H9: Social support provided
H10: Social support received
H11: Social activities
H12: Income
H13: Less economic burden

## (AA). United Kingdom

A

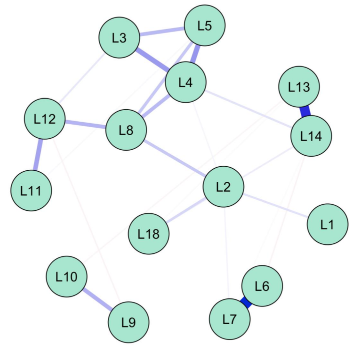


B

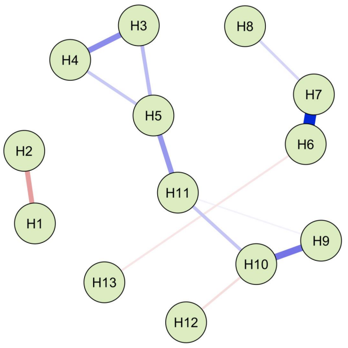


C

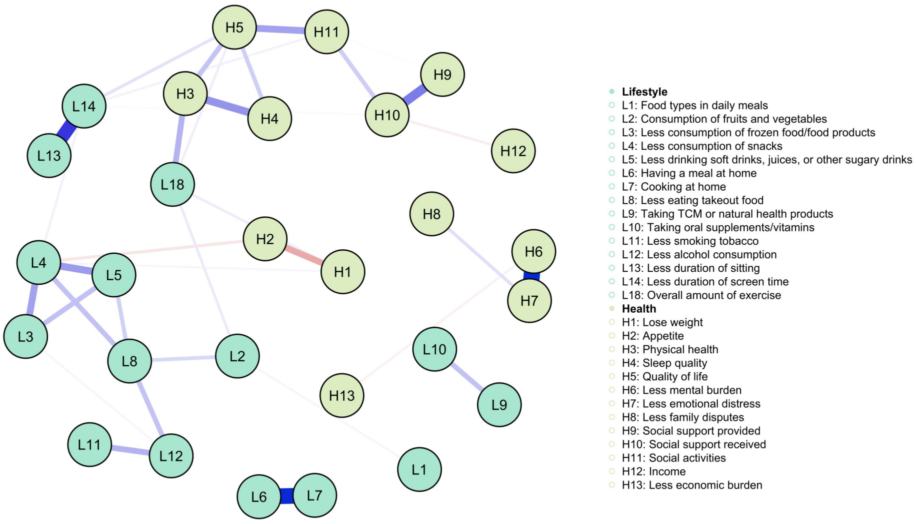

# (AB). United States

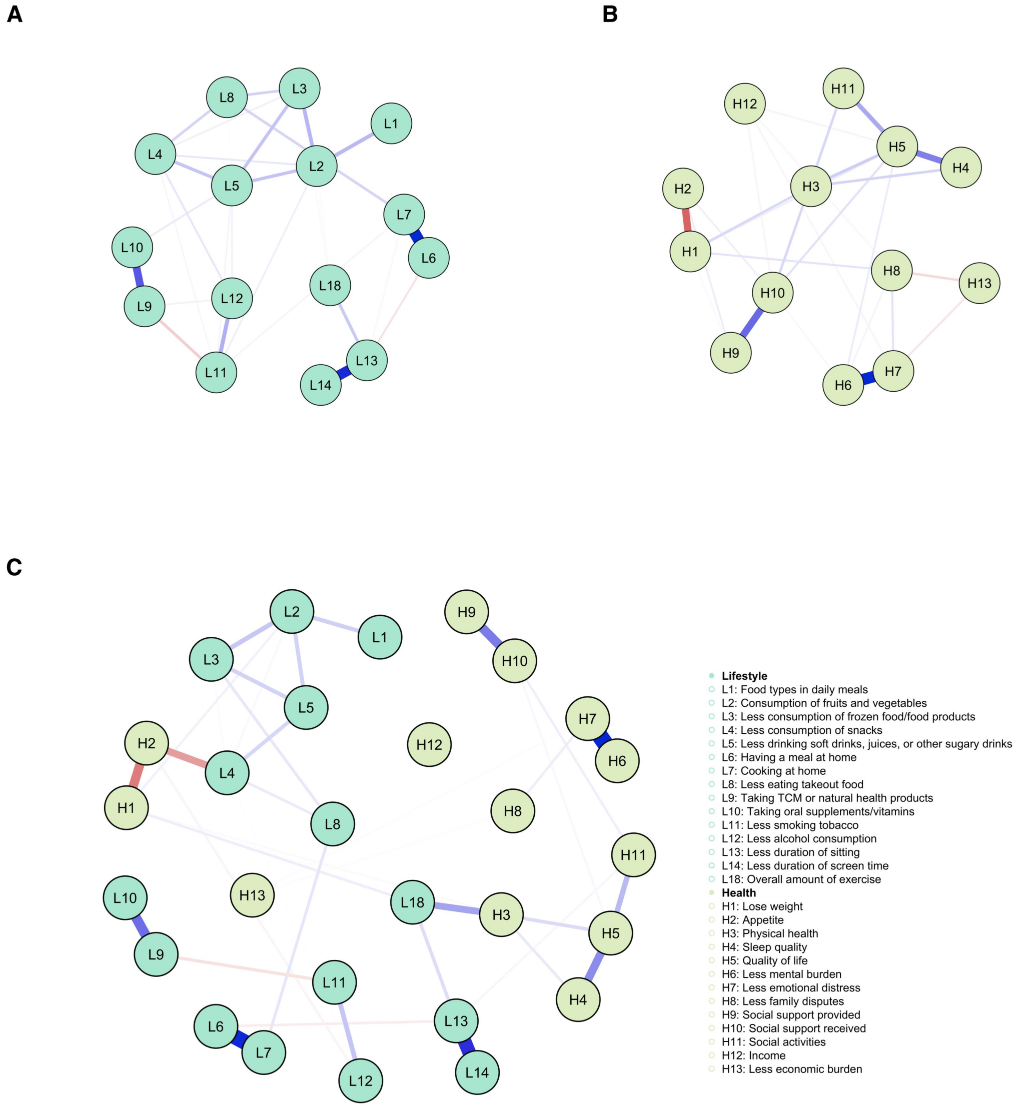

## (AC). Vietnam

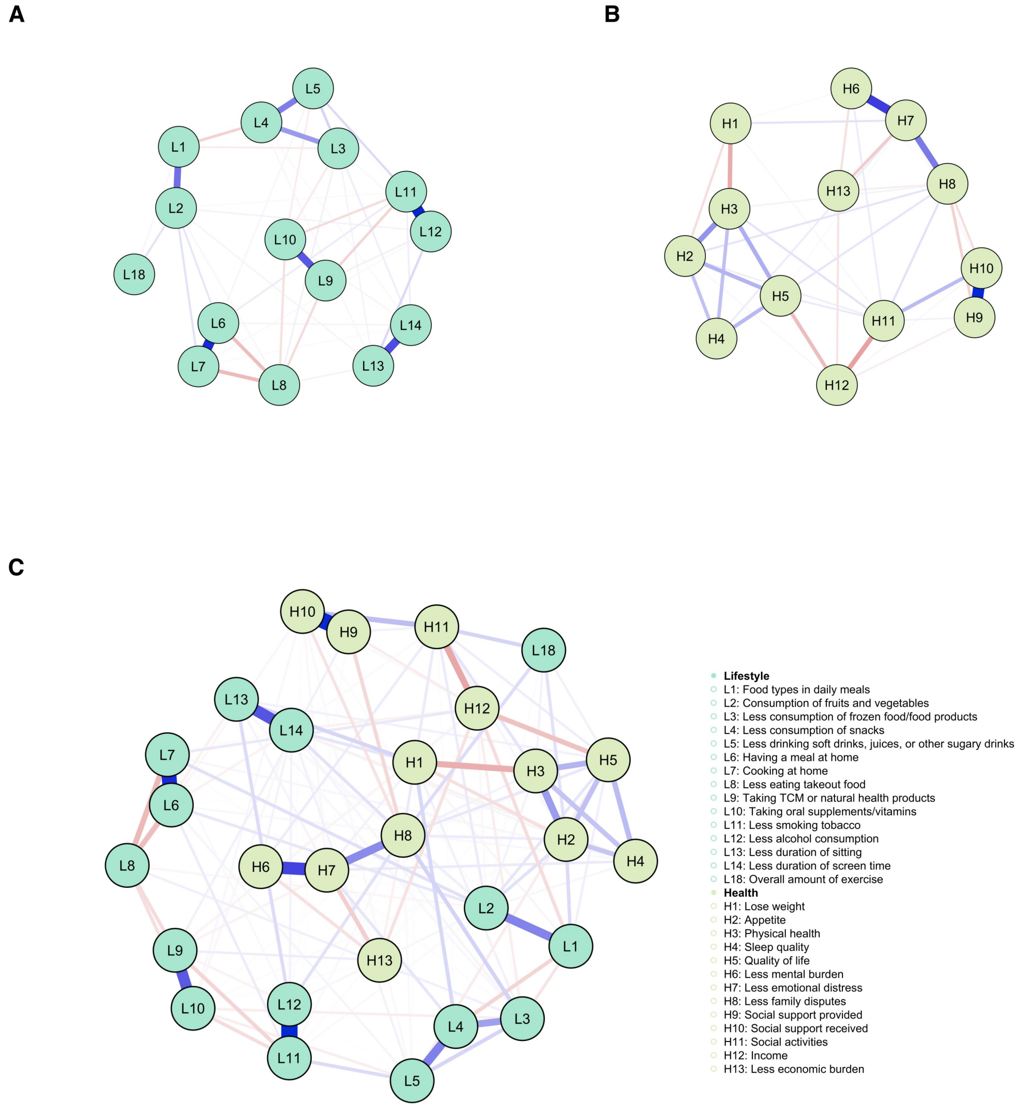

A
B
C
Lifestyle
L1: Food types in daily meals
L2: Consumption of fruits and vegetables
L3: Less consumption of frozen food/food products
L4: Less consumption of snacks
L5: Less drinking soft drinks, juices, or other sugary drinks
L6: Having a meal at home
L7: Cooking at home
L8: Less eating takeout food
L9: Taking TCM or natural health products
L10: Taking oral supplements/vitamins
L11: Less smoking tobacco
L12: Less alcohol consumption
L13: Less duration of sitting
L14: Less duration of screen time
L18: Overall amount of exercise
Health
H1: Lose weight
H2: Appetite
H3: Physical health
H4: Sleep quality
H5: Quality of life
H6: Less mental burden
H7: Less emotional distress
H8: Less family disputes
H9: Social support provided
H10: Social support received
H11: Social activities
H12: Income
H13: Less economic burden

**Figure S4.** Centrality difference test of lifestyle, health outcomes, and bridge networks across 29 countries

## (A). Australia

**Lifestyle network**

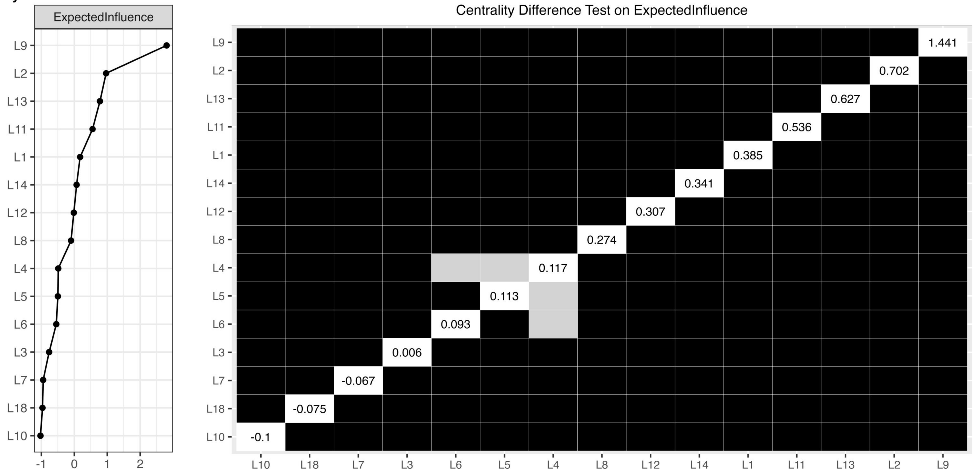


**Health outcome network**

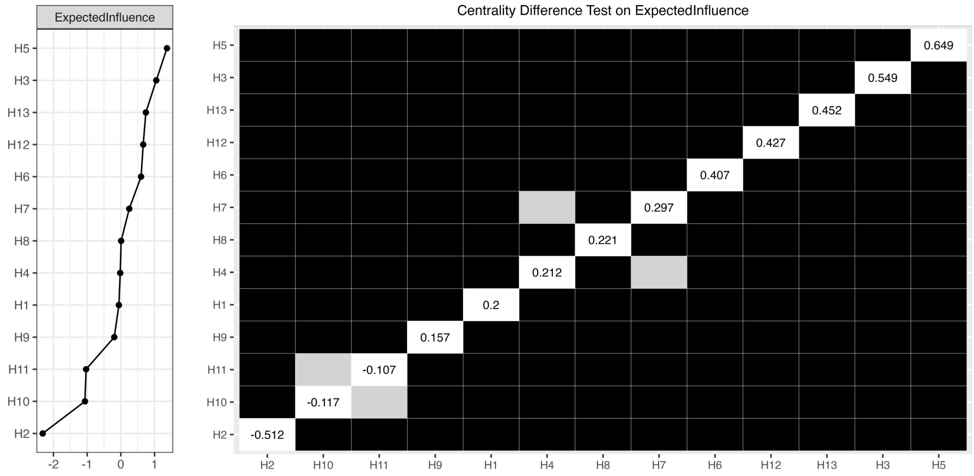


**Bridge network**

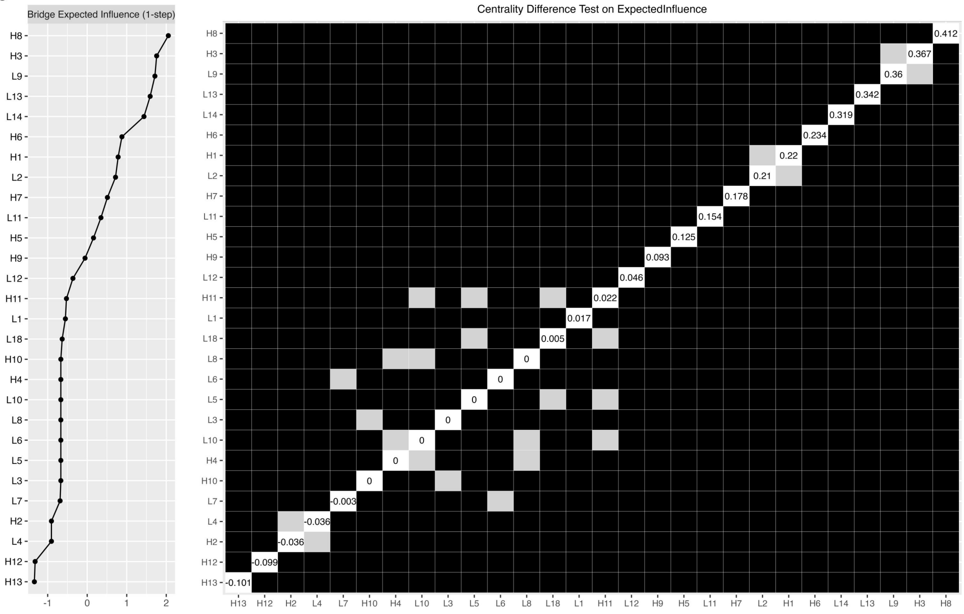

# (B). Brazil

### Lifestyle network

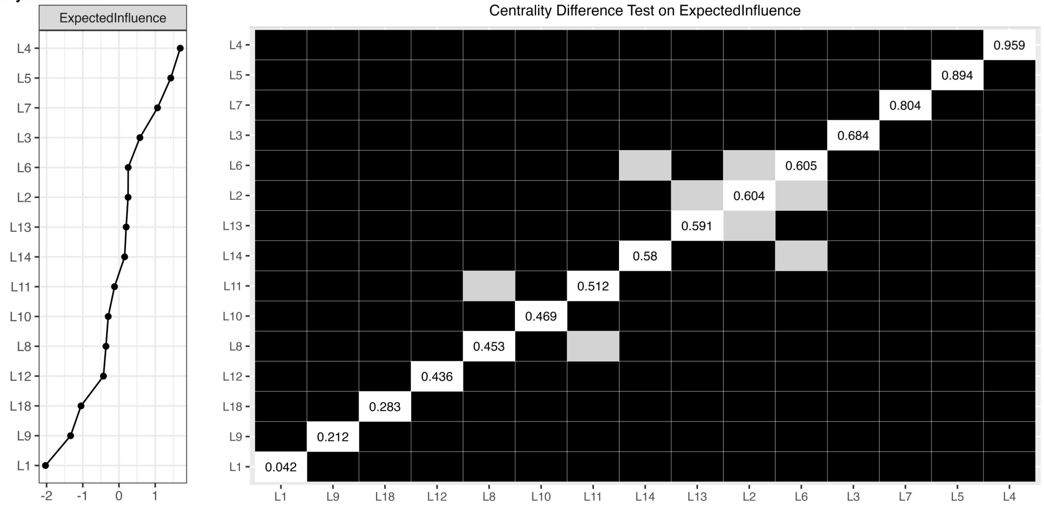


### Health outcome network

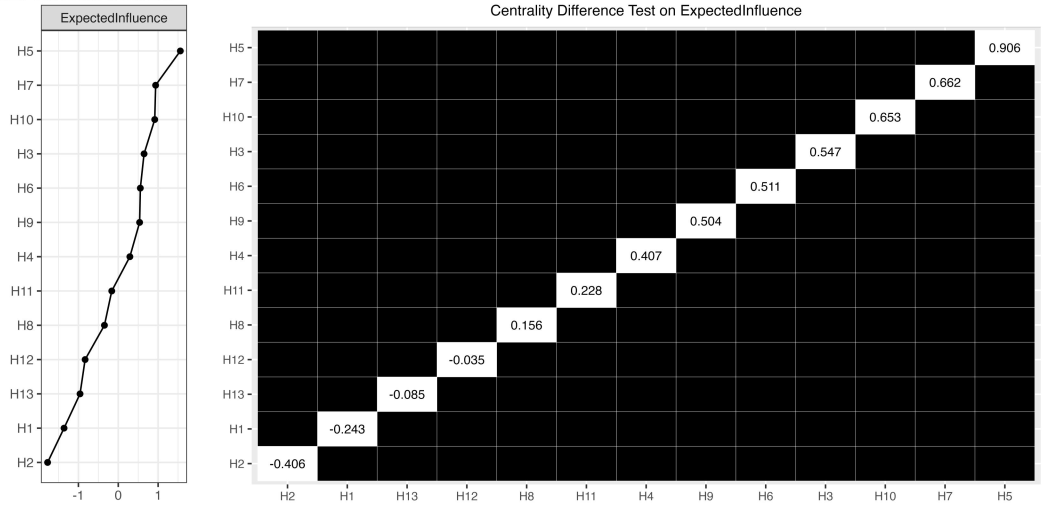


### Bridge network

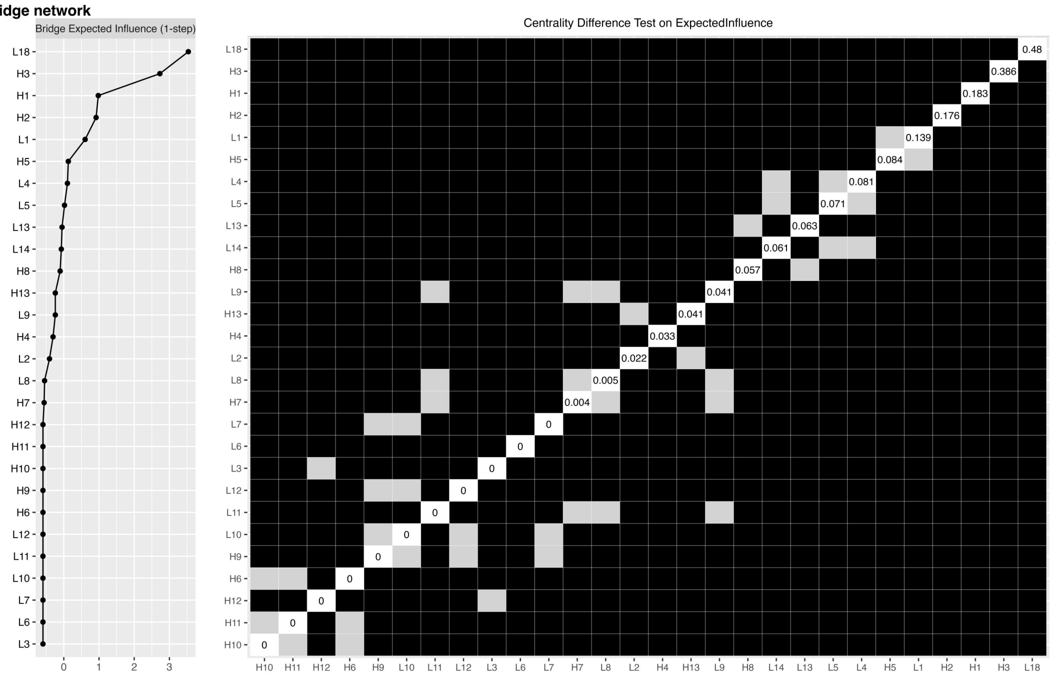

## (C). Burundi

**Lifestyle network**

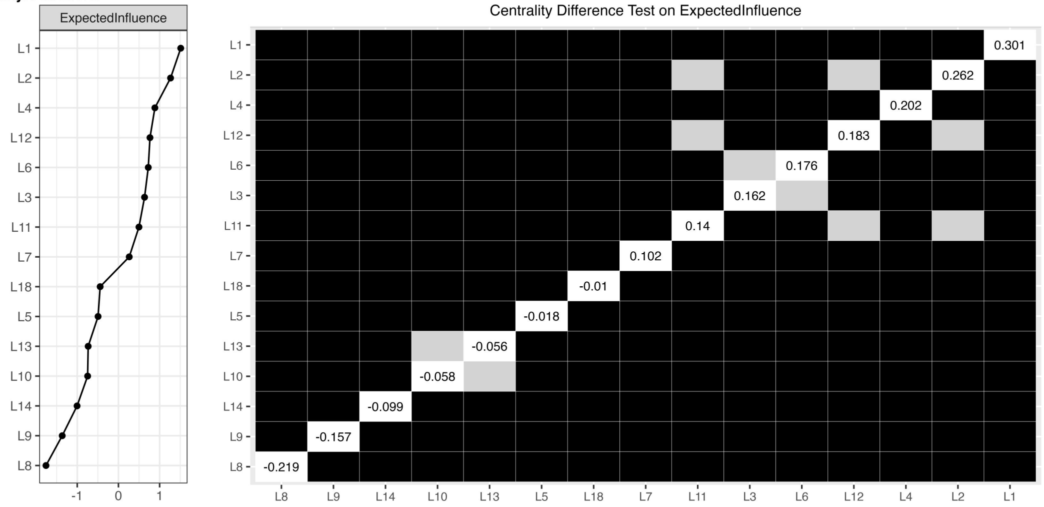


**Health outcome network**

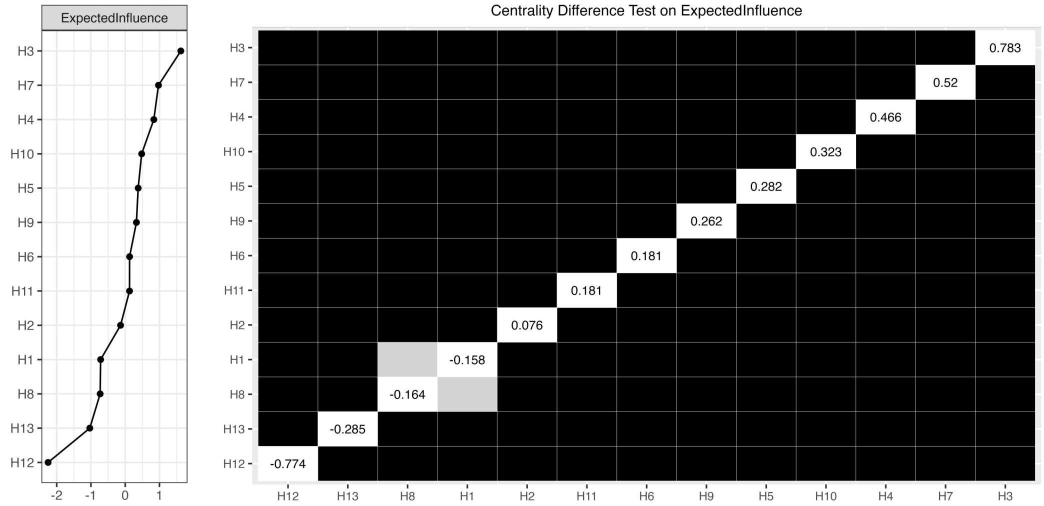


**Bridge network**

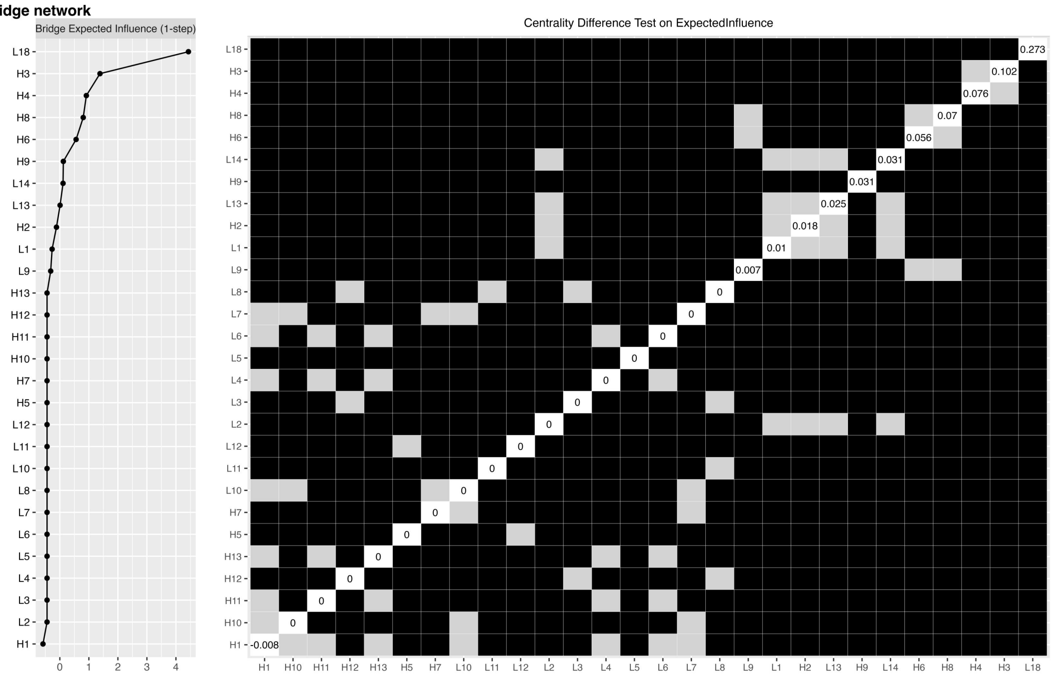

## (D). Canada

### Lifestyle network

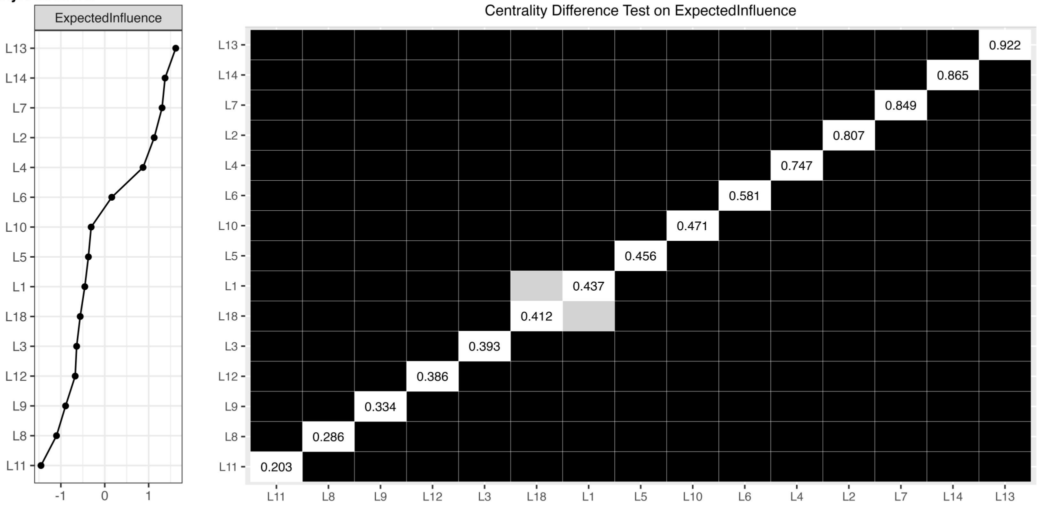


### Health outcome network

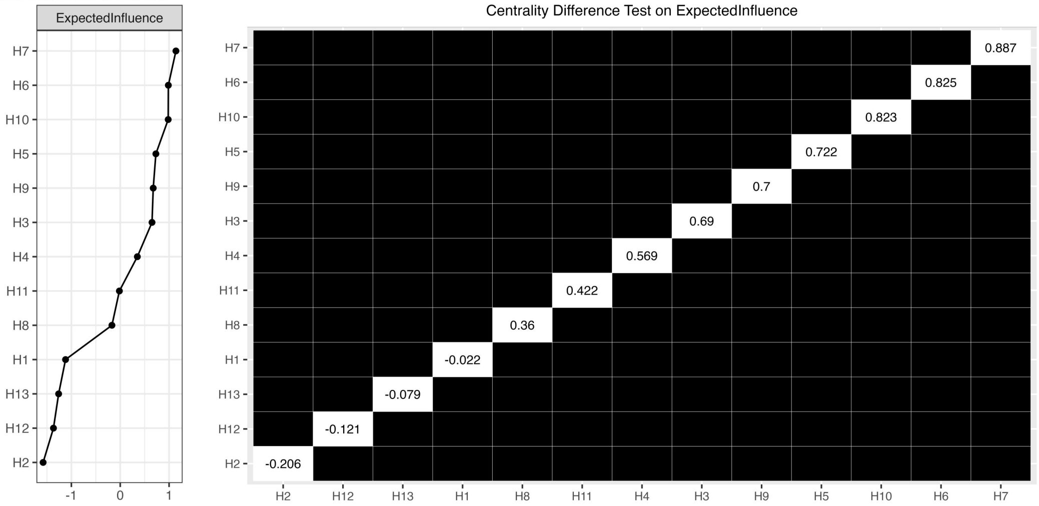


### Bridge network

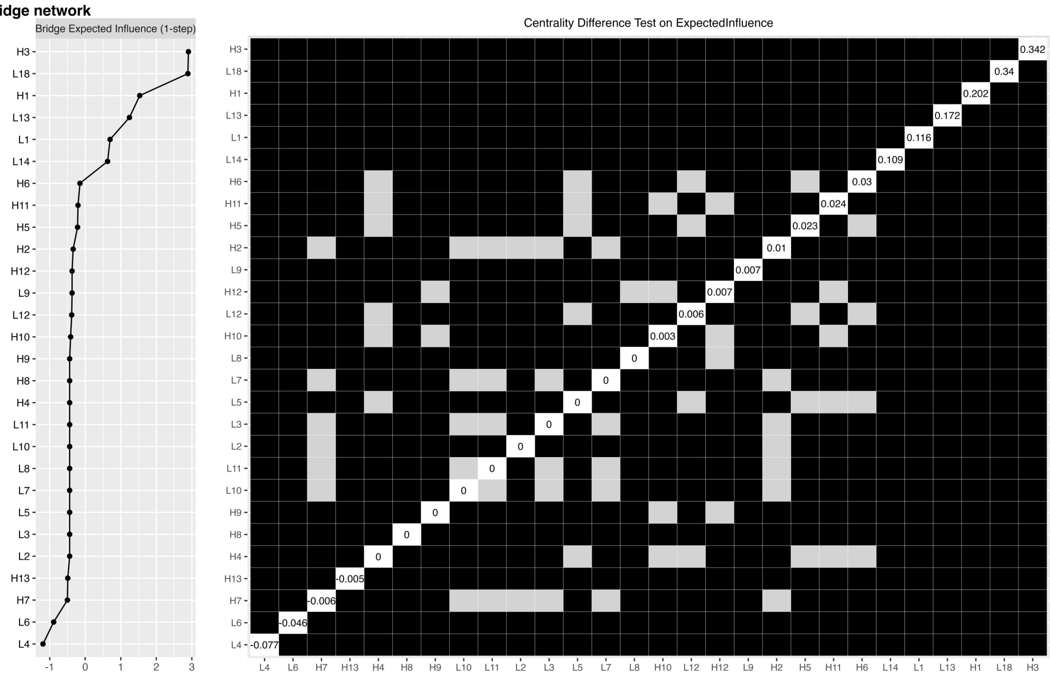

# (E). Chile

**Lifestyle network**

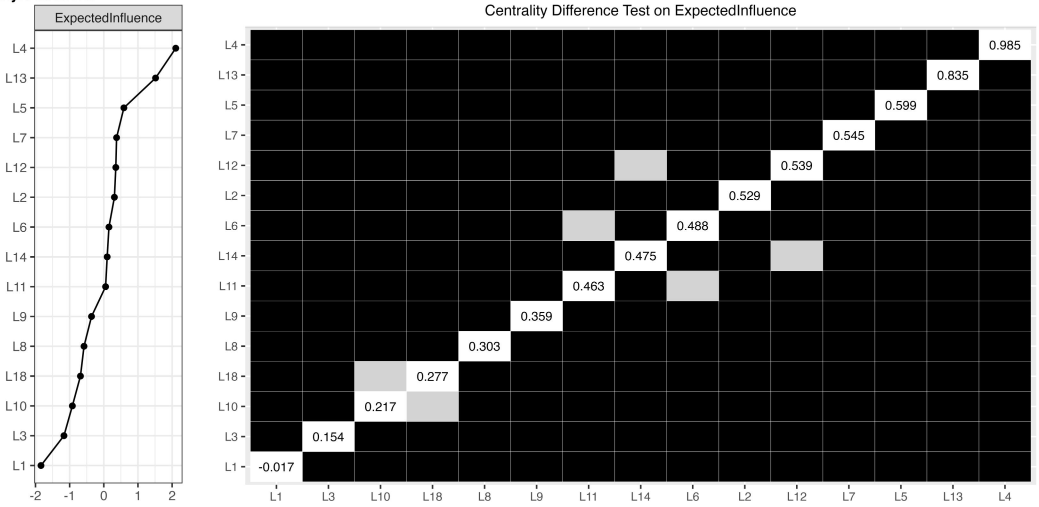


**Health outcome network**

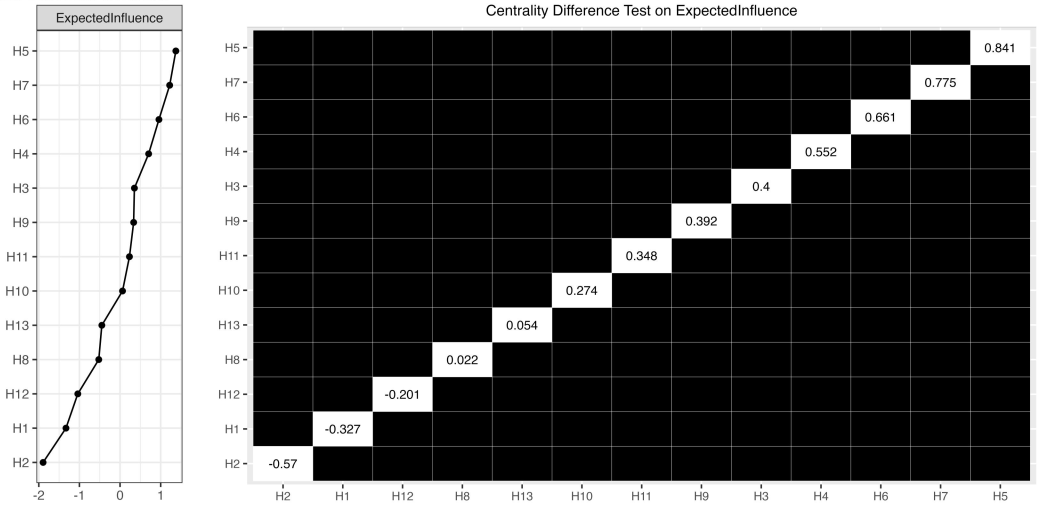


**Bridge network**

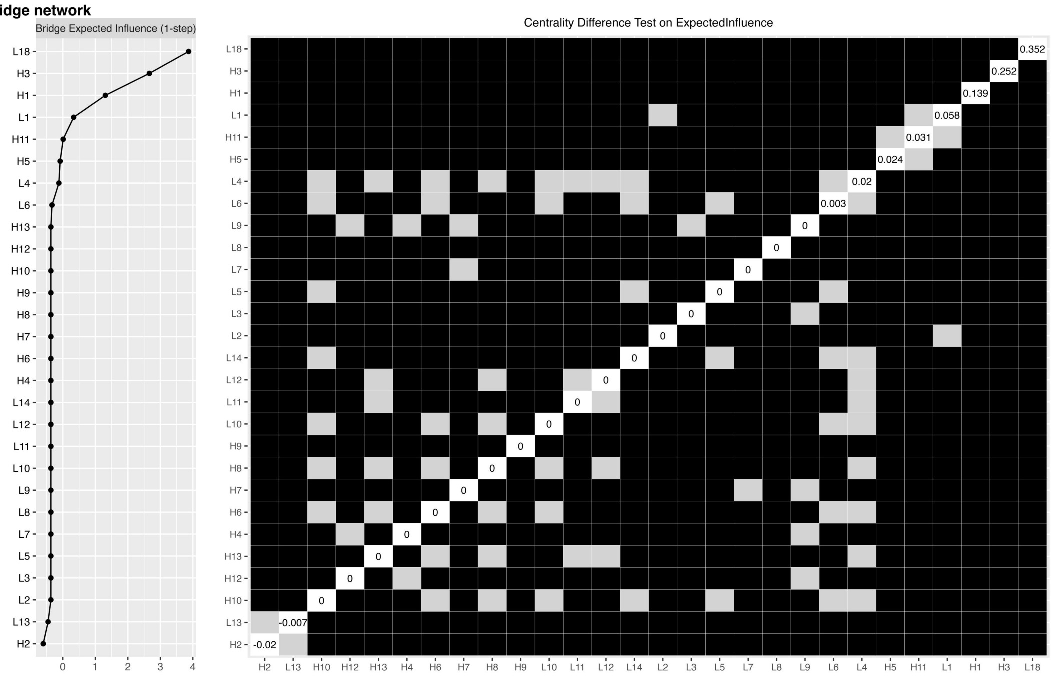

# (F). Egypt

**Lifestyle network**

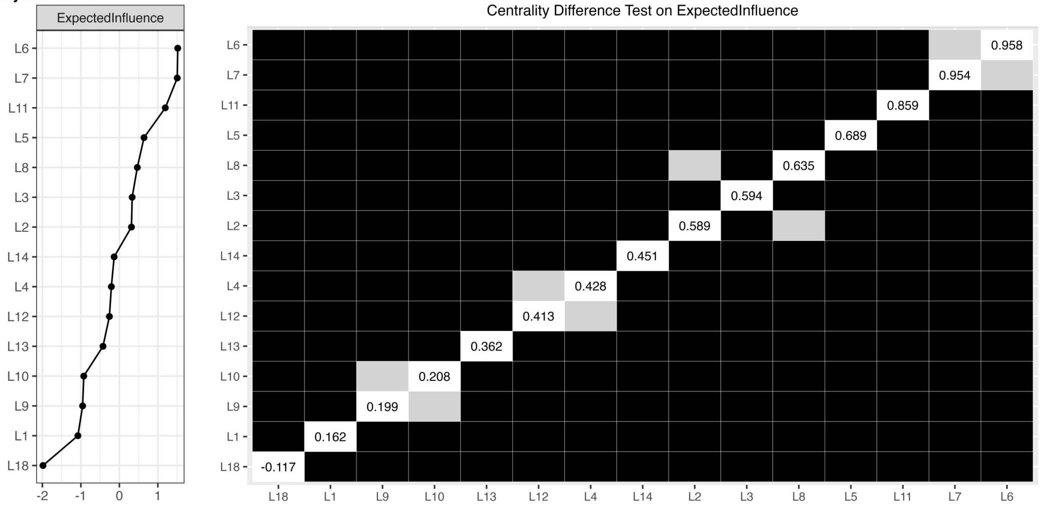


**Health outcome network**

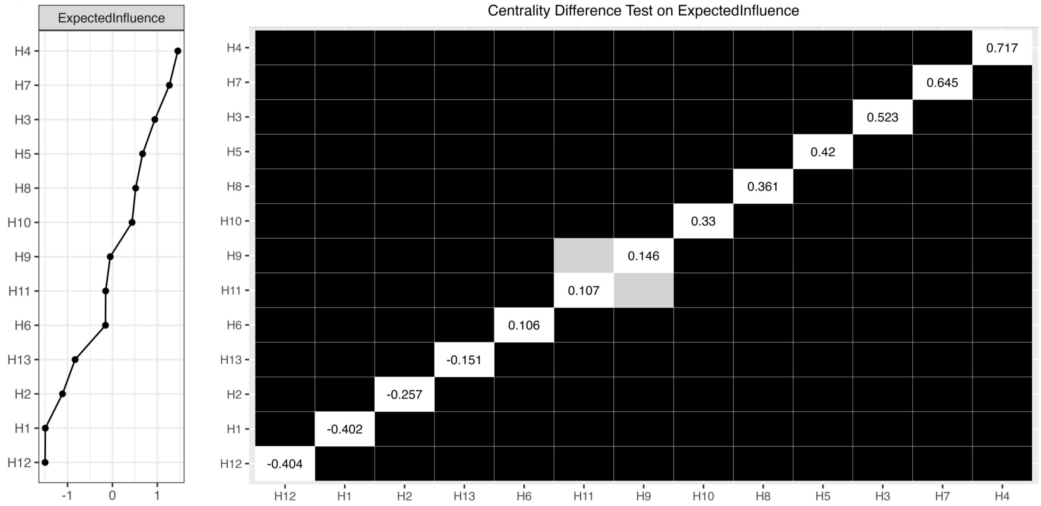


**Bridge network**

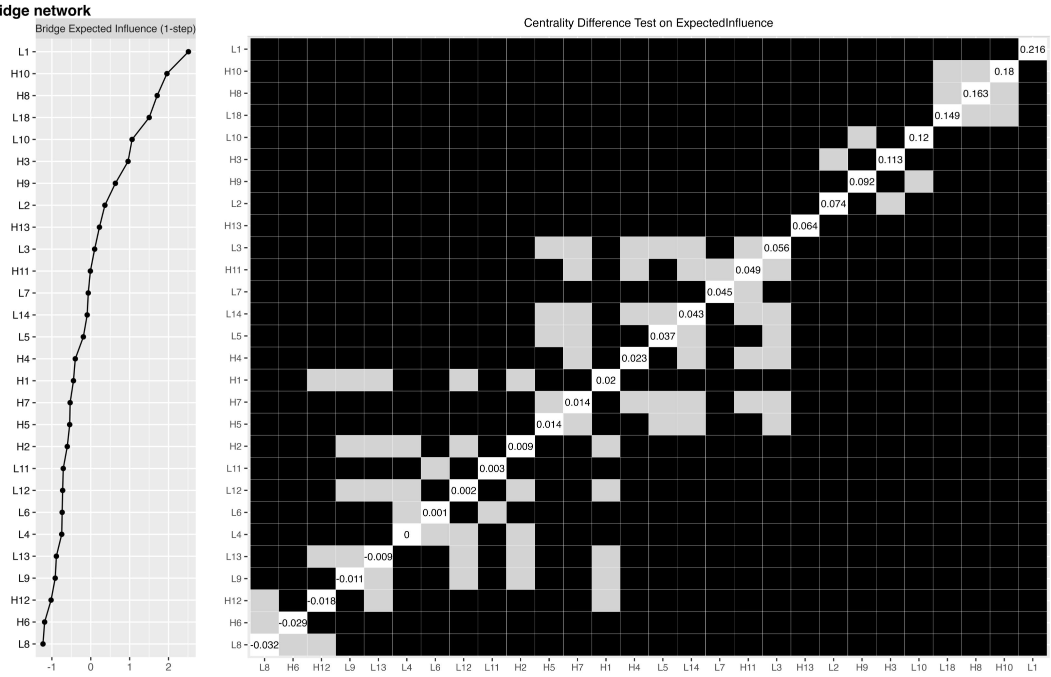

## (G). Guatemala

**Lifestyle network**

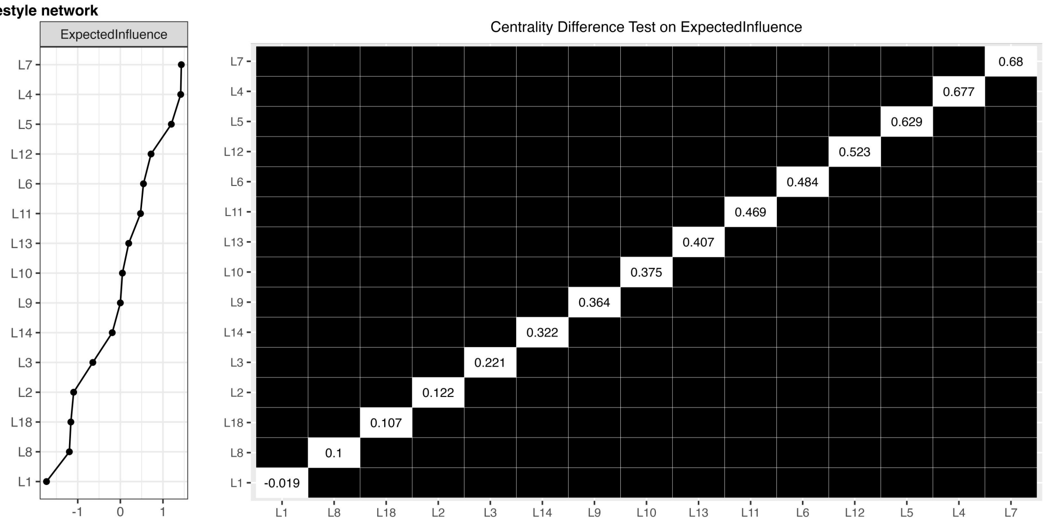


**Health outcome network**

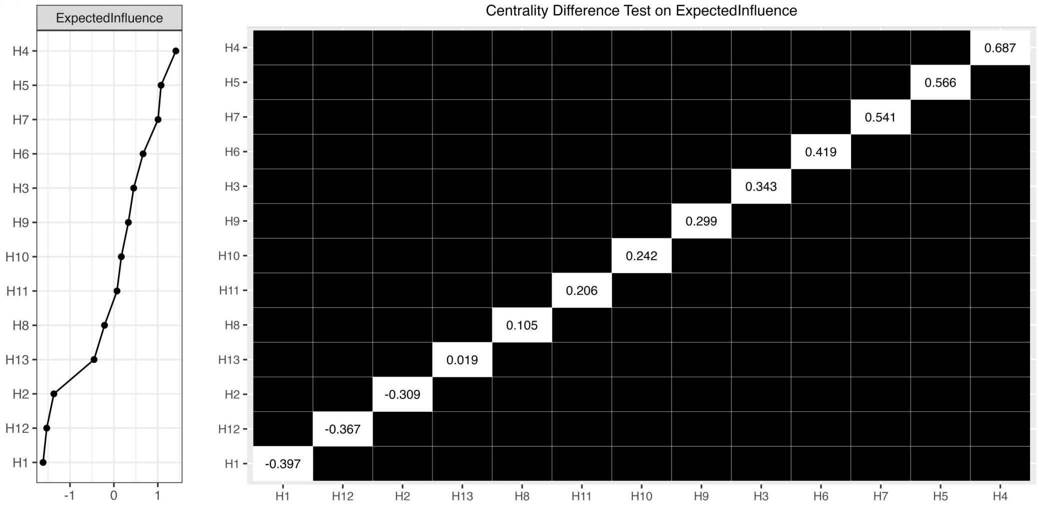


**Bridge network**

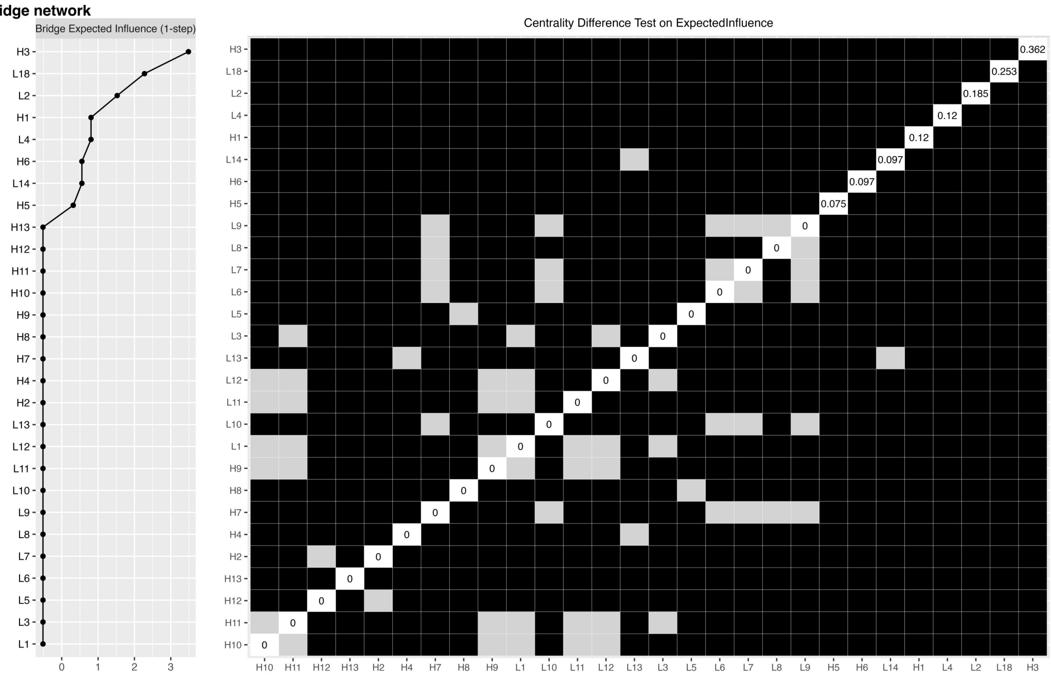

# (H). Hong Kong

**Lifestyle network**

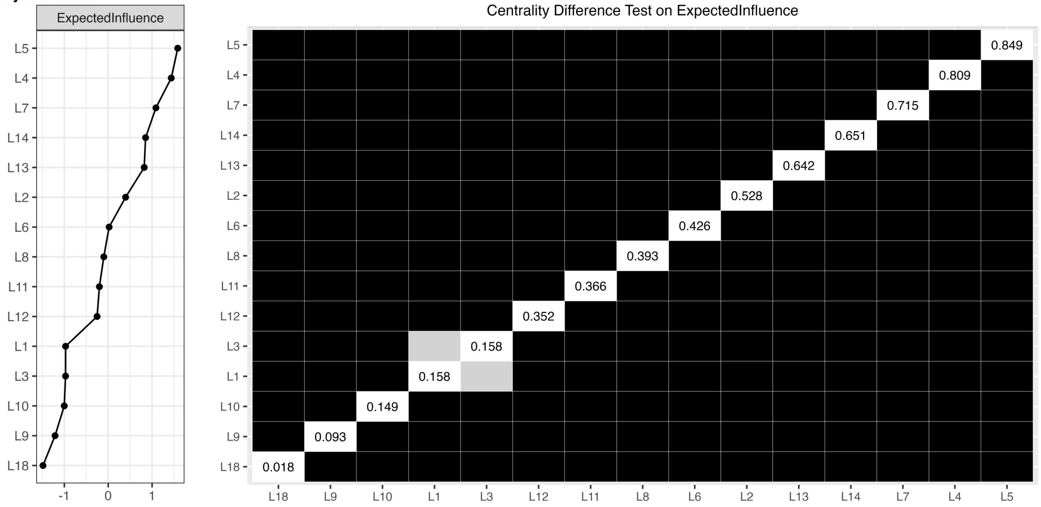


**Health outcome network**

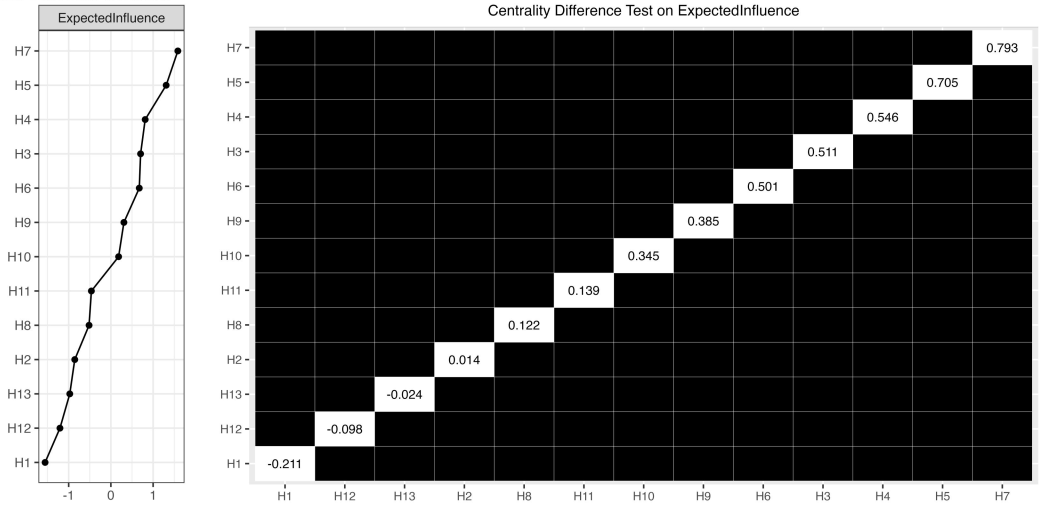


**Bridge network**

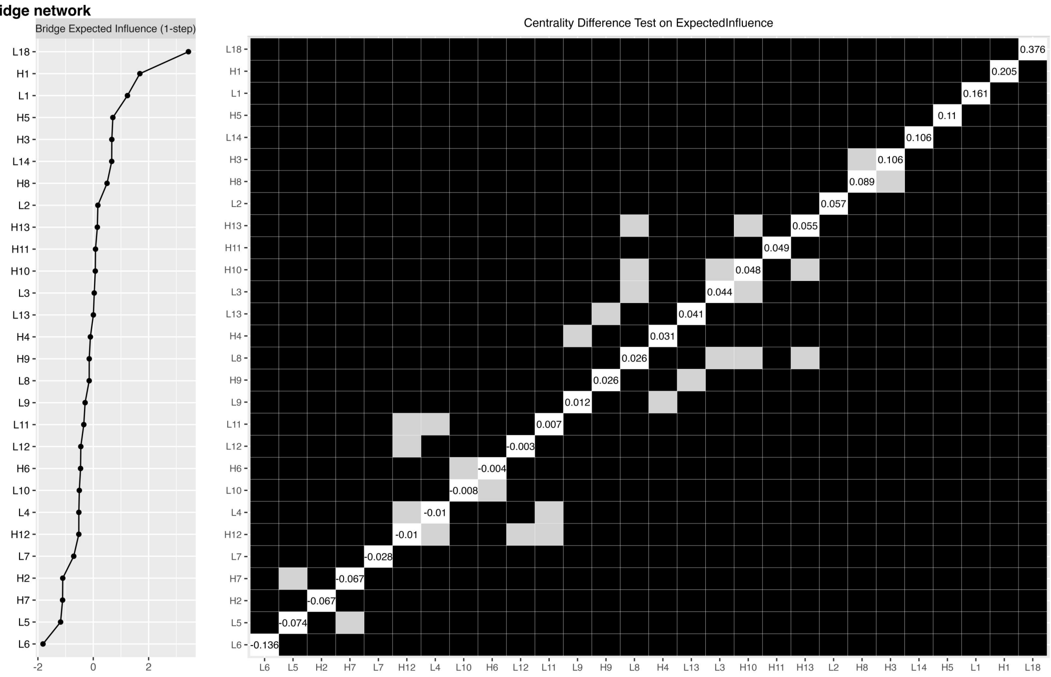

# (I). India

**Lifestyle network**

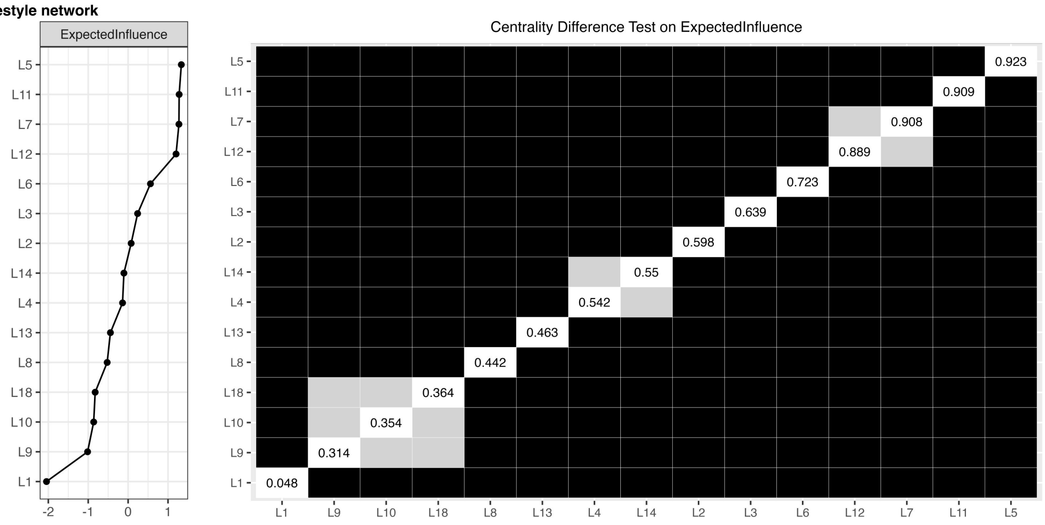


**Health outcome network**

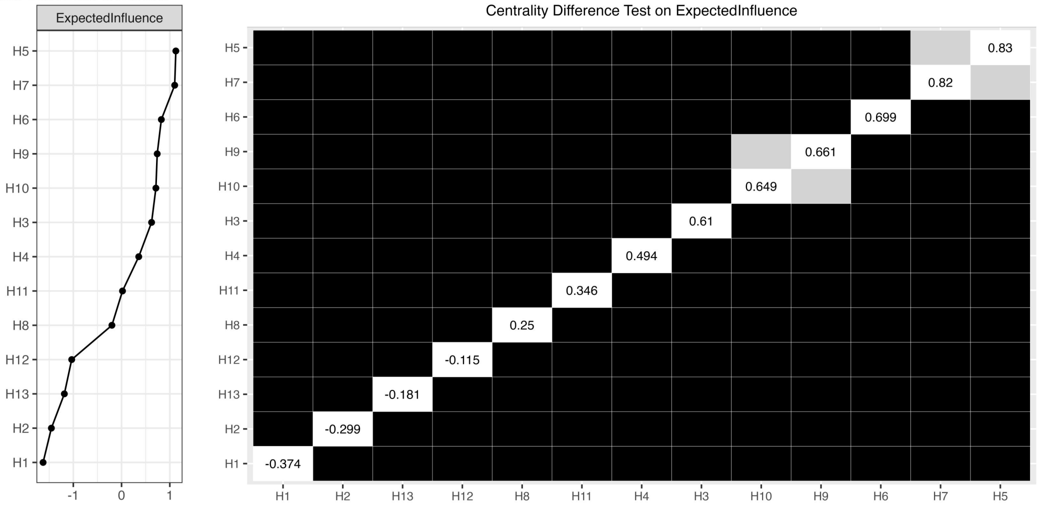


**Bridge network**

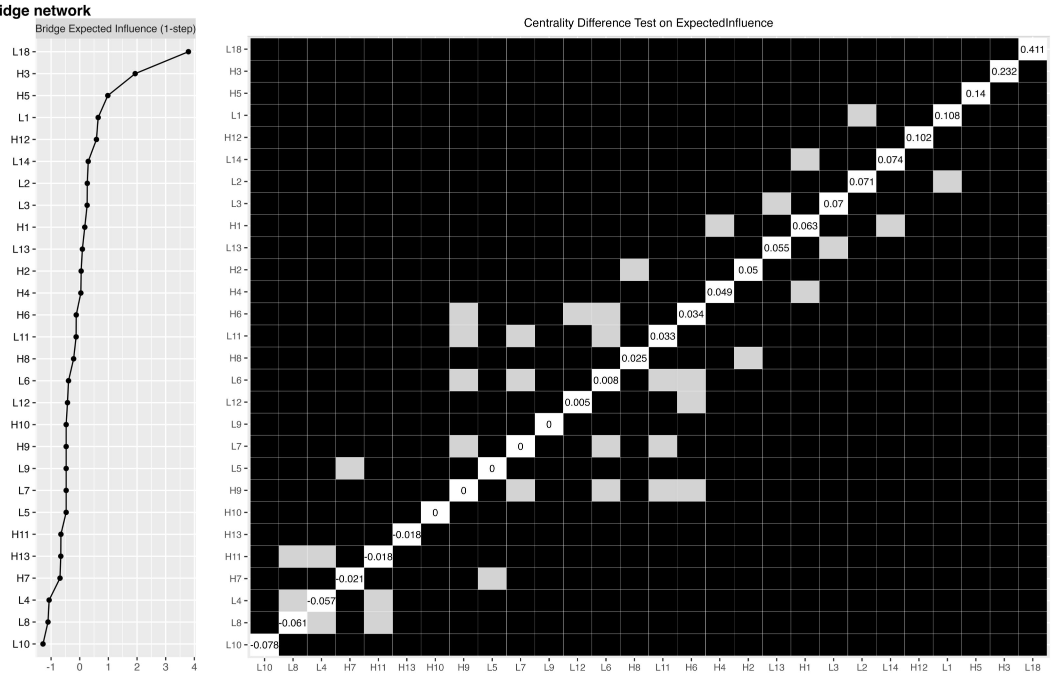

## (J). Indonesia

**Lifestyle network**

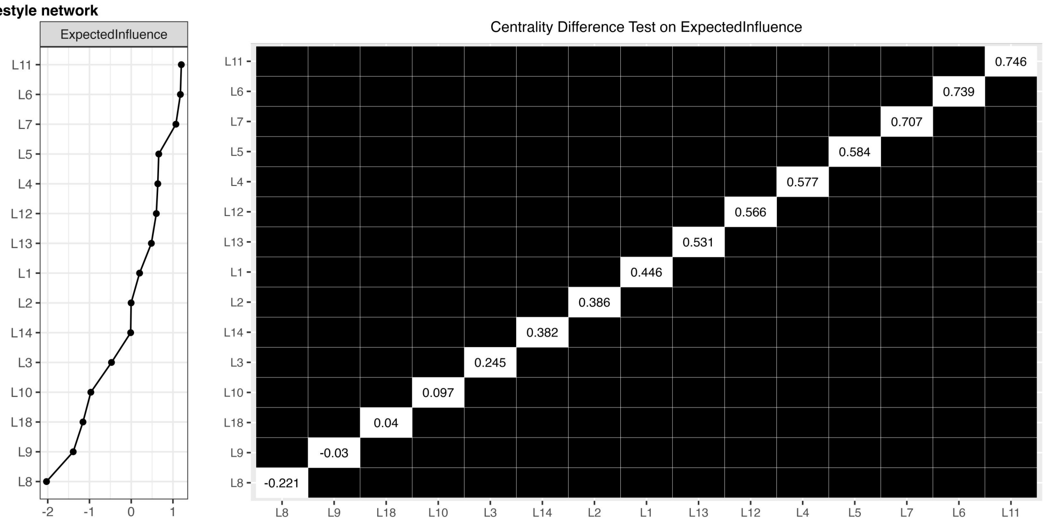


**Health outcome network**

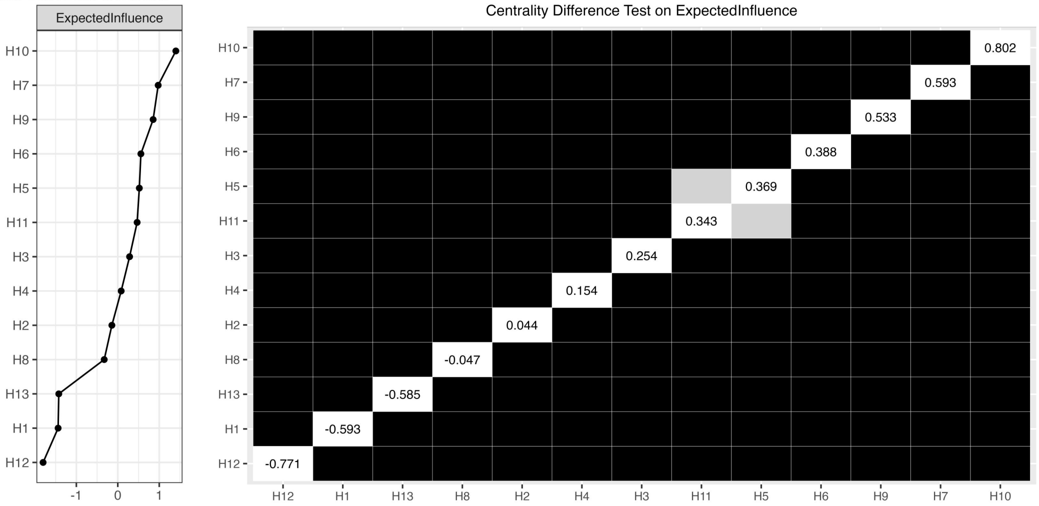


**Bridge network**

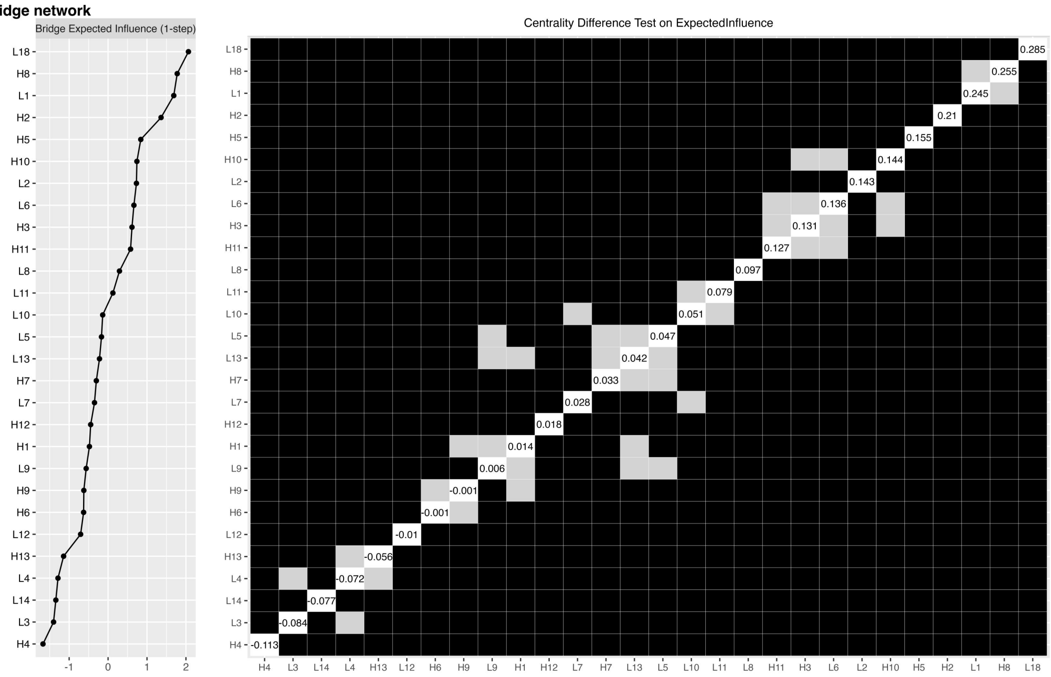

# (K). Italy

**Lifestyle network**

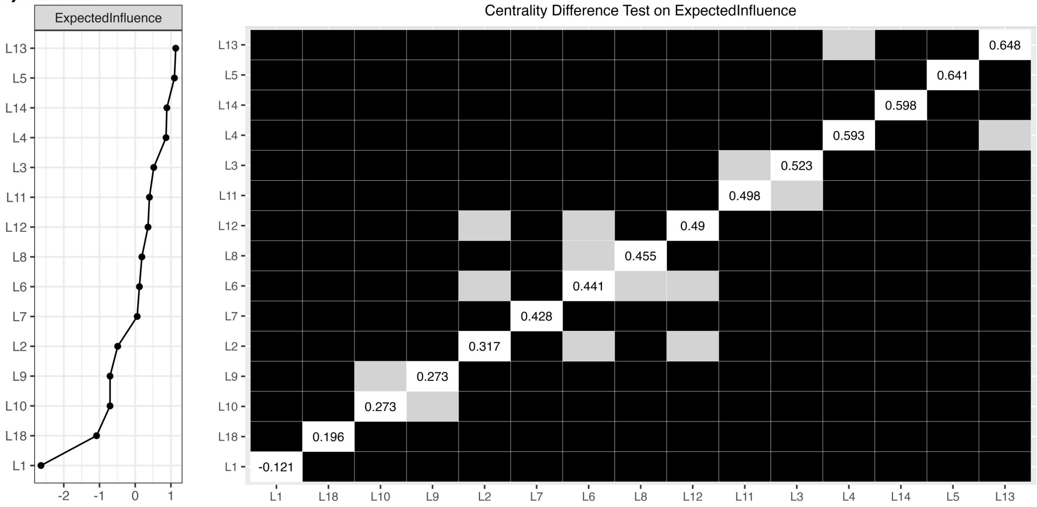


**Health outcome network**

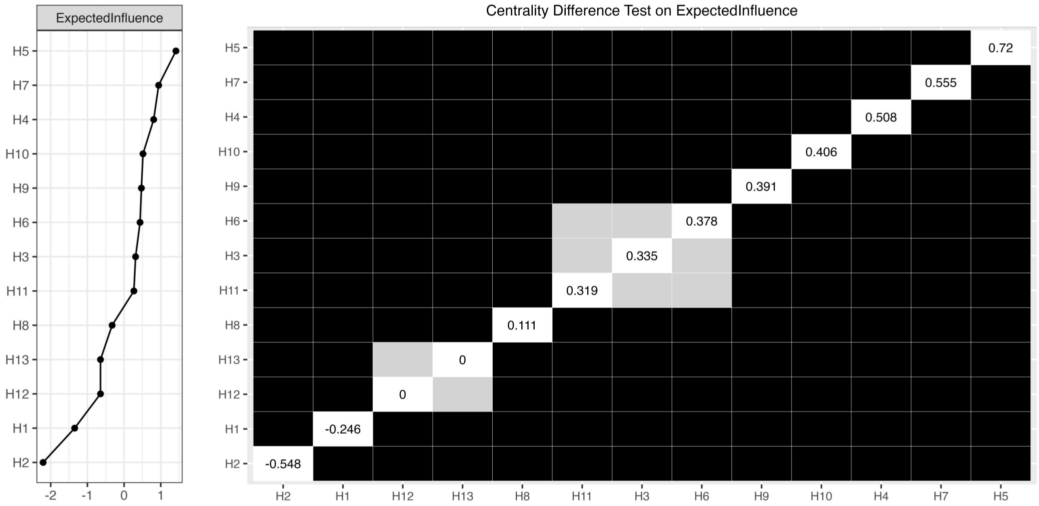


**Bridge network**

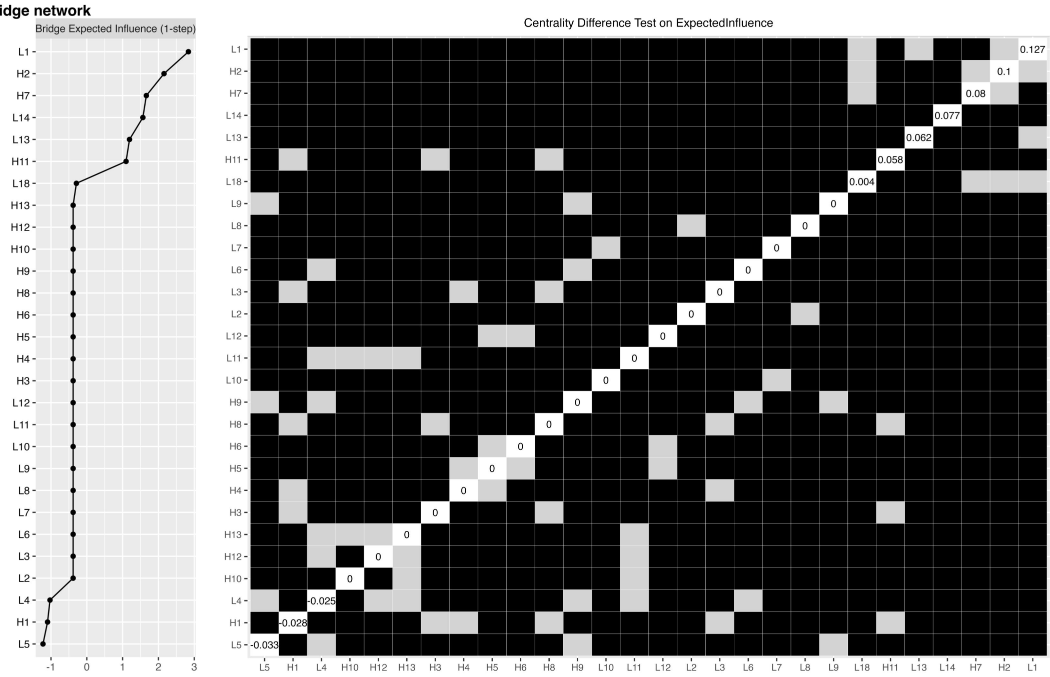

# (L). Lebanon

**Lifestyle network**

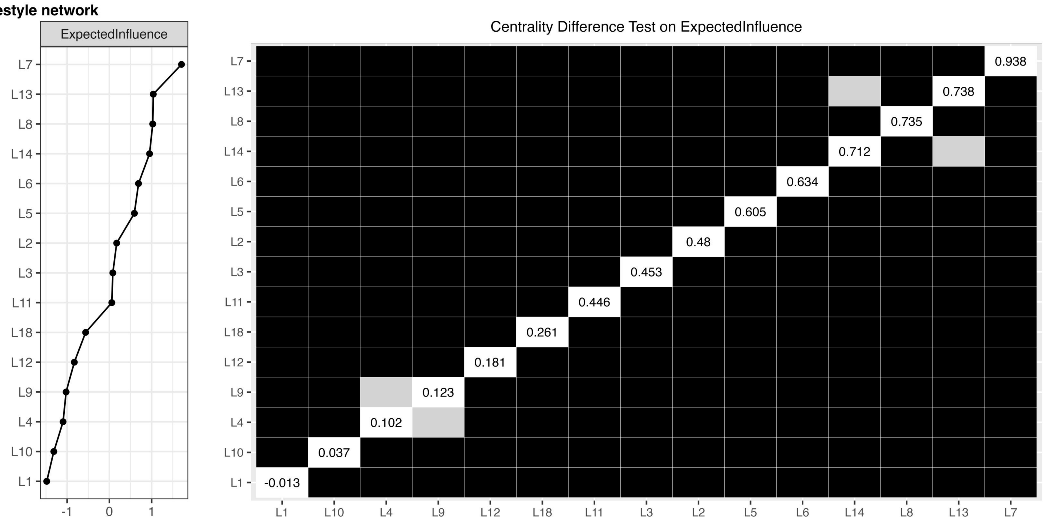


**Health outcome network**

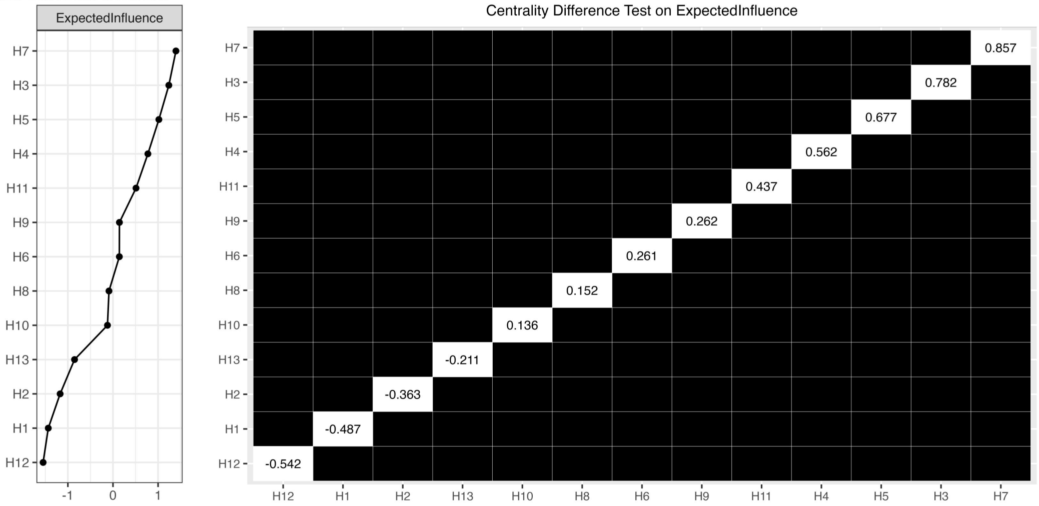


**Bridge network**

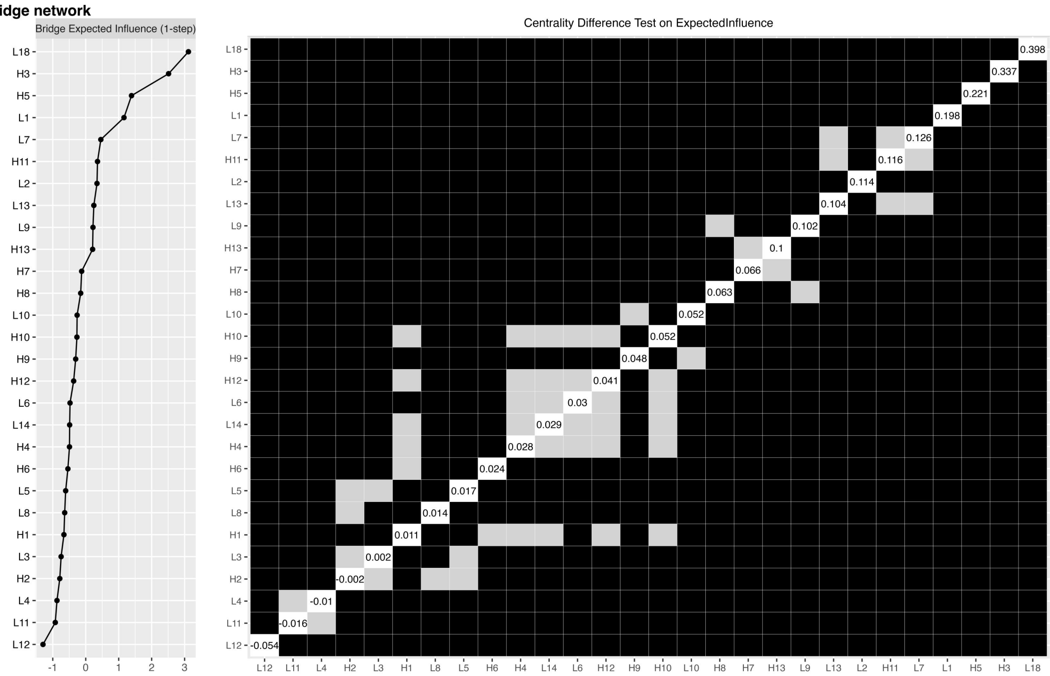

## (M). Libya

**Lifestyle network**

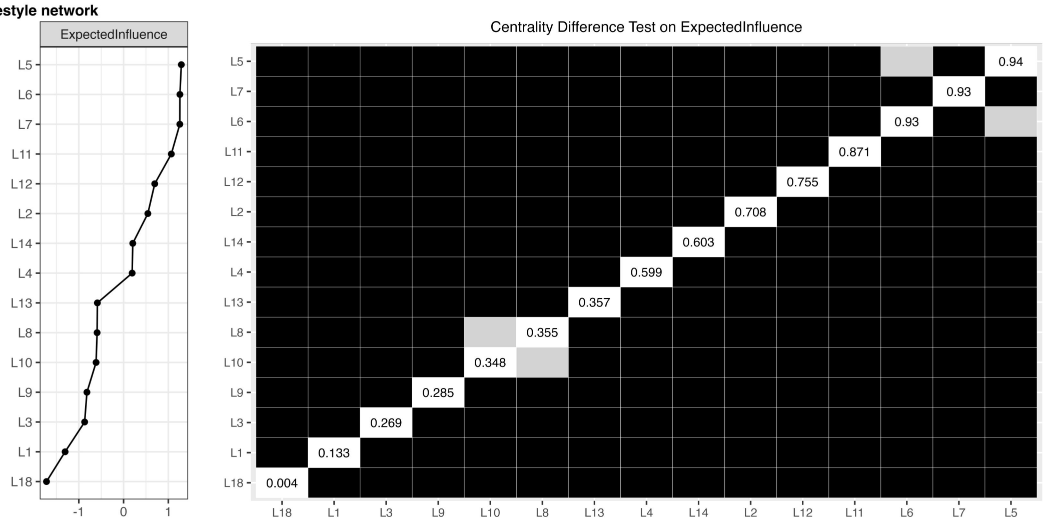


**Health outcome network**

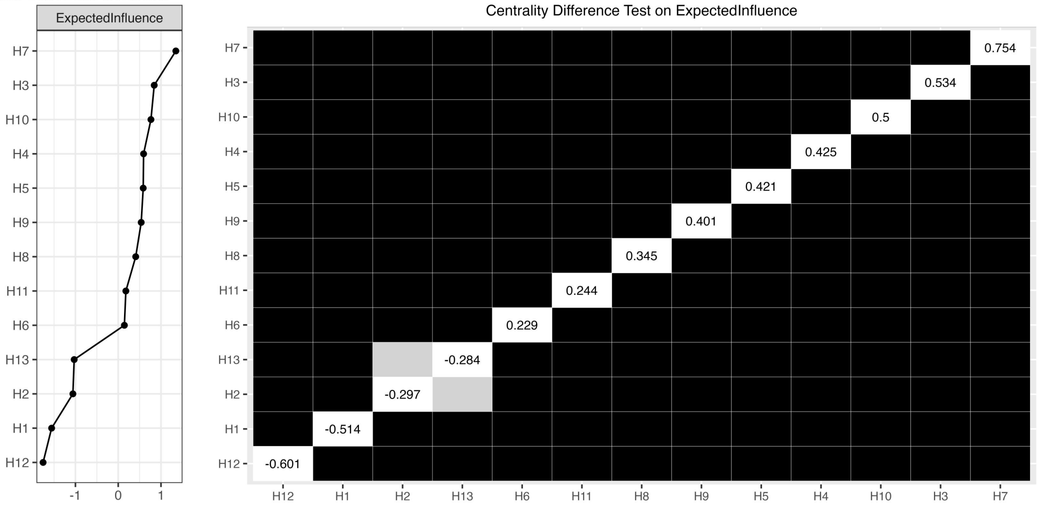


**Bridge network**

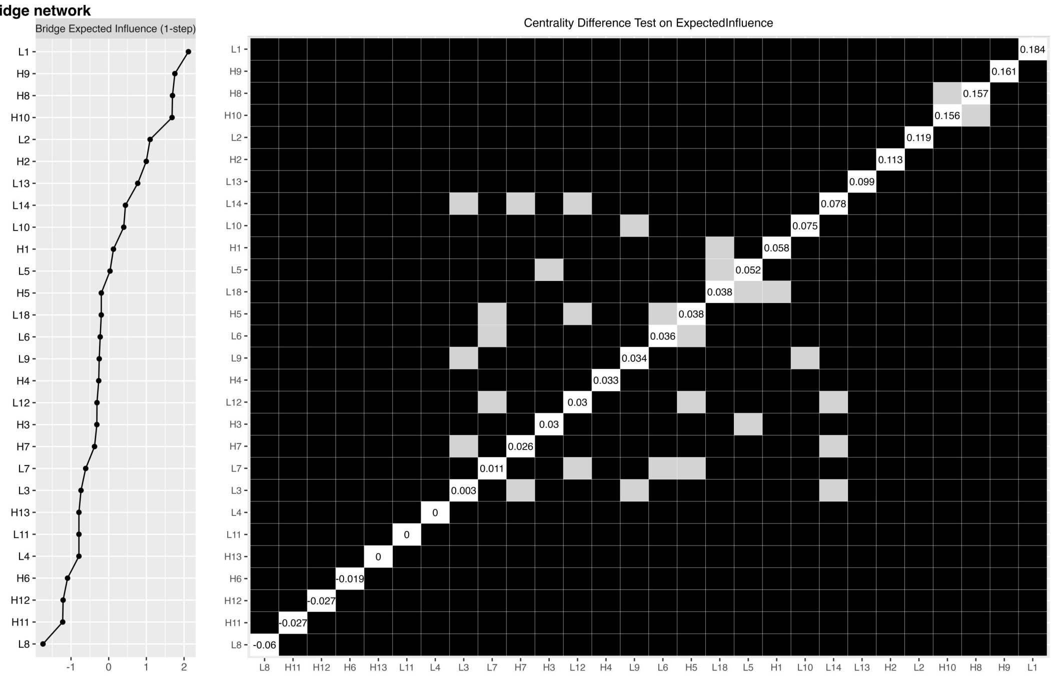

## (N). Macau

**Lifestyle network**

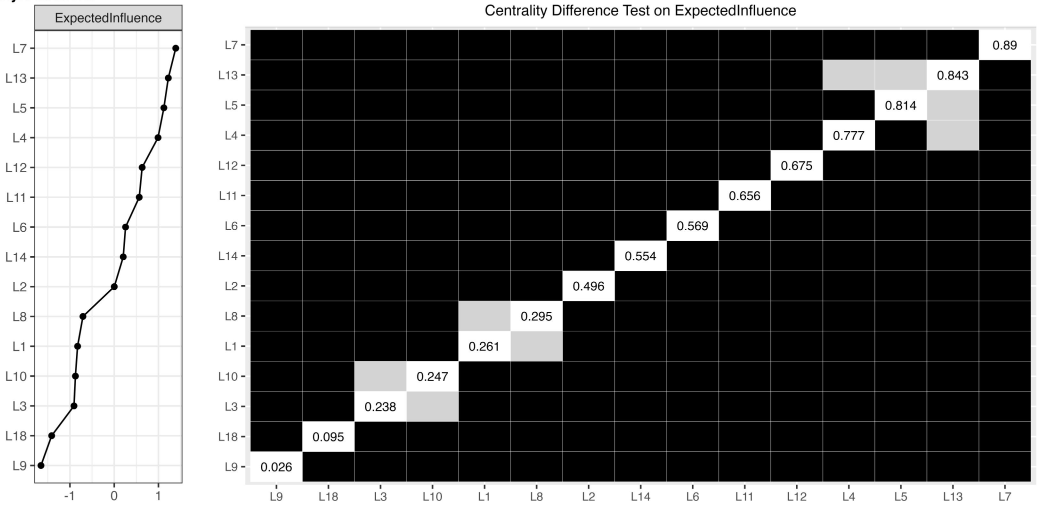


**Health outcome network**

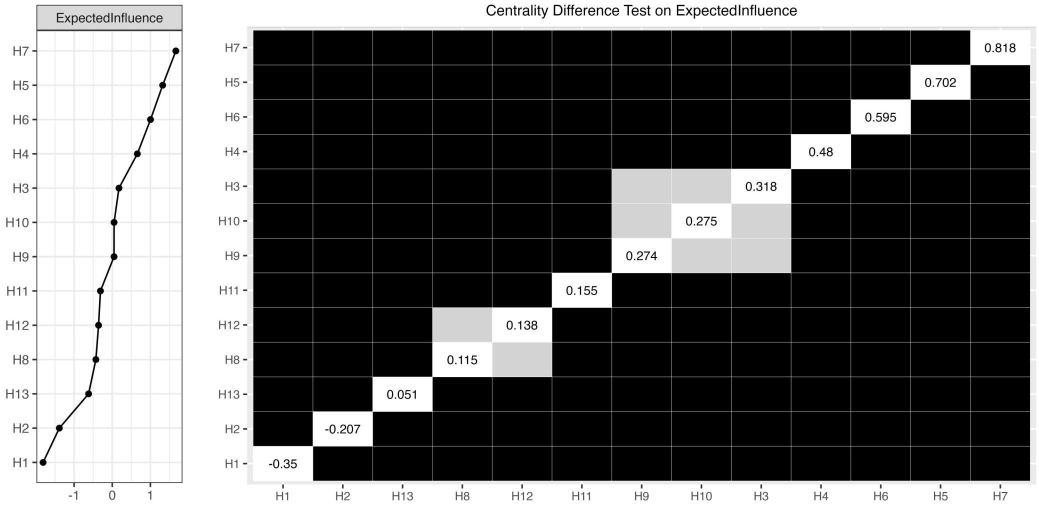

## (O). Mainland China

**Lifestyle network**

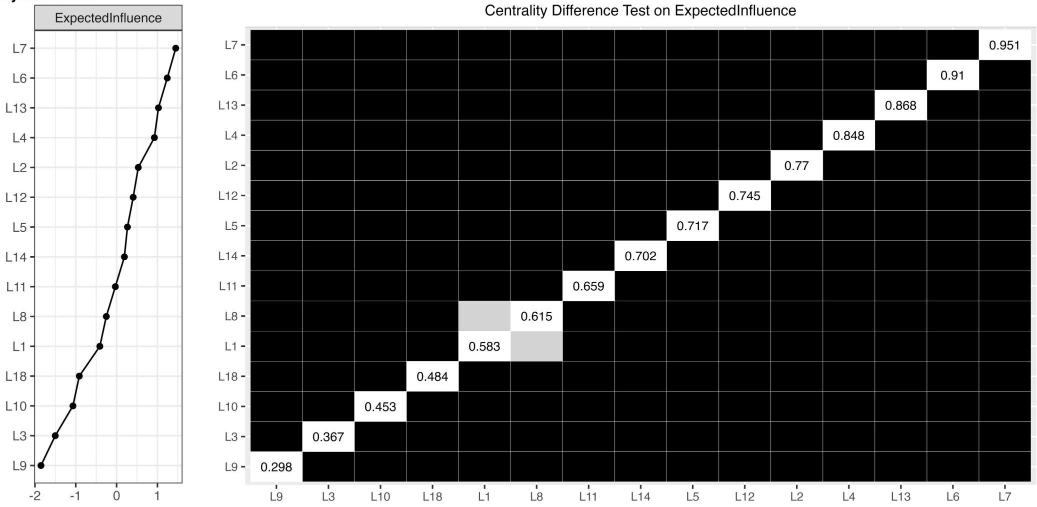


**Health outcome network**

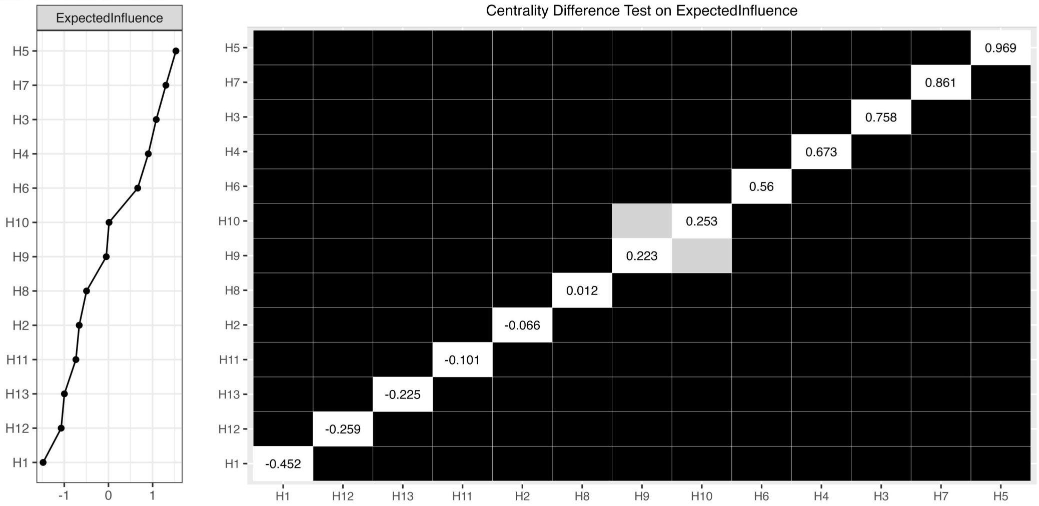


**Bridge network**

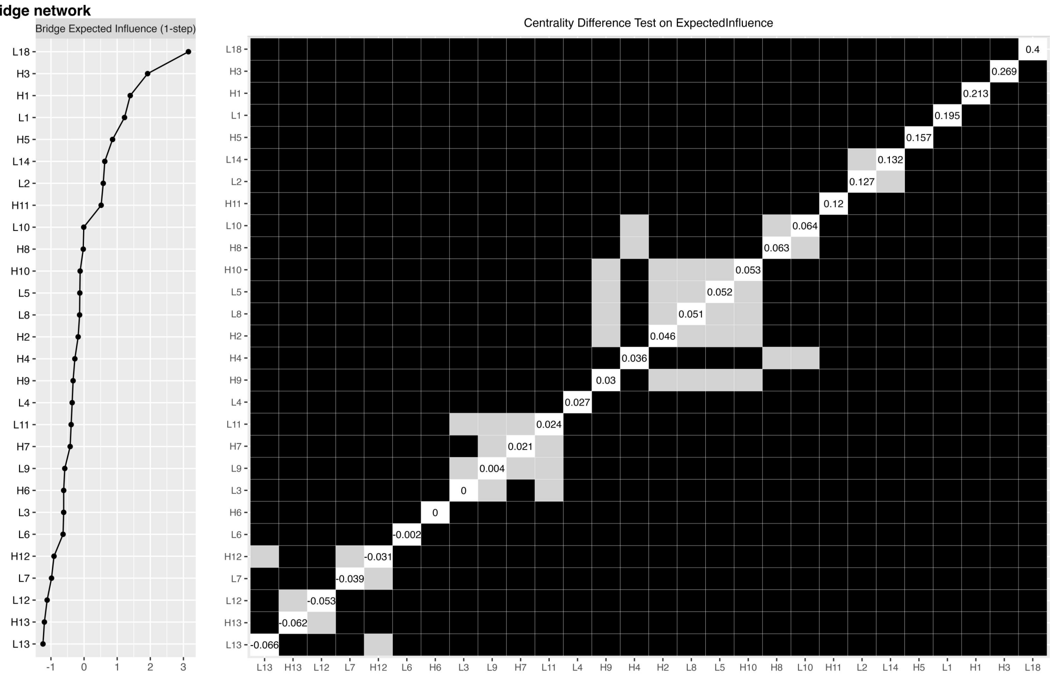

## (P). Malaysia

**Lifestyle network**

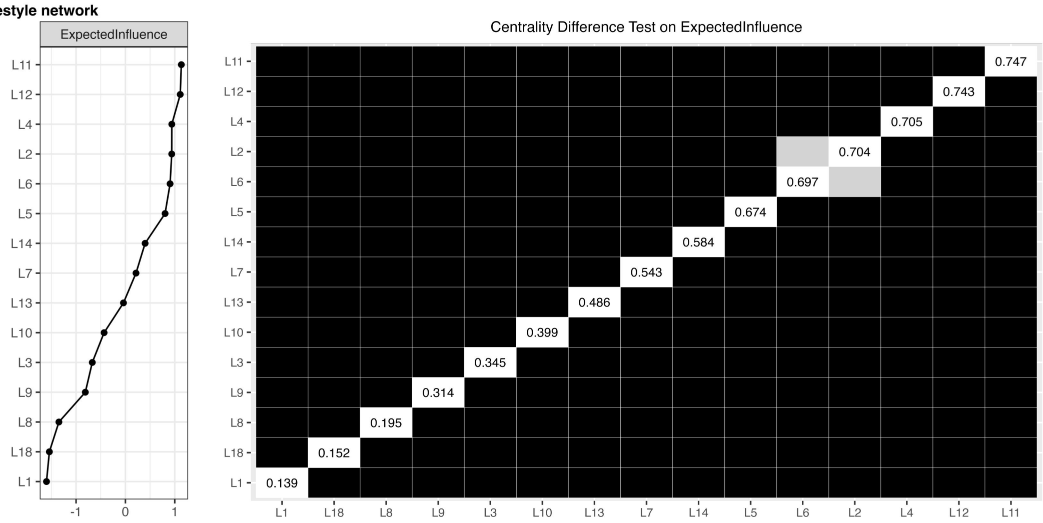


**Health outcome network**

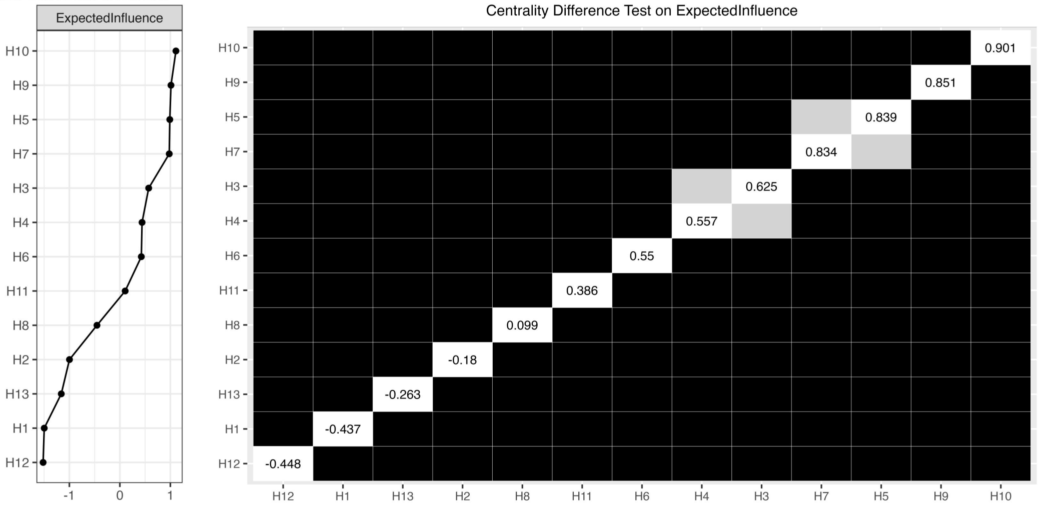


**Bridge network**

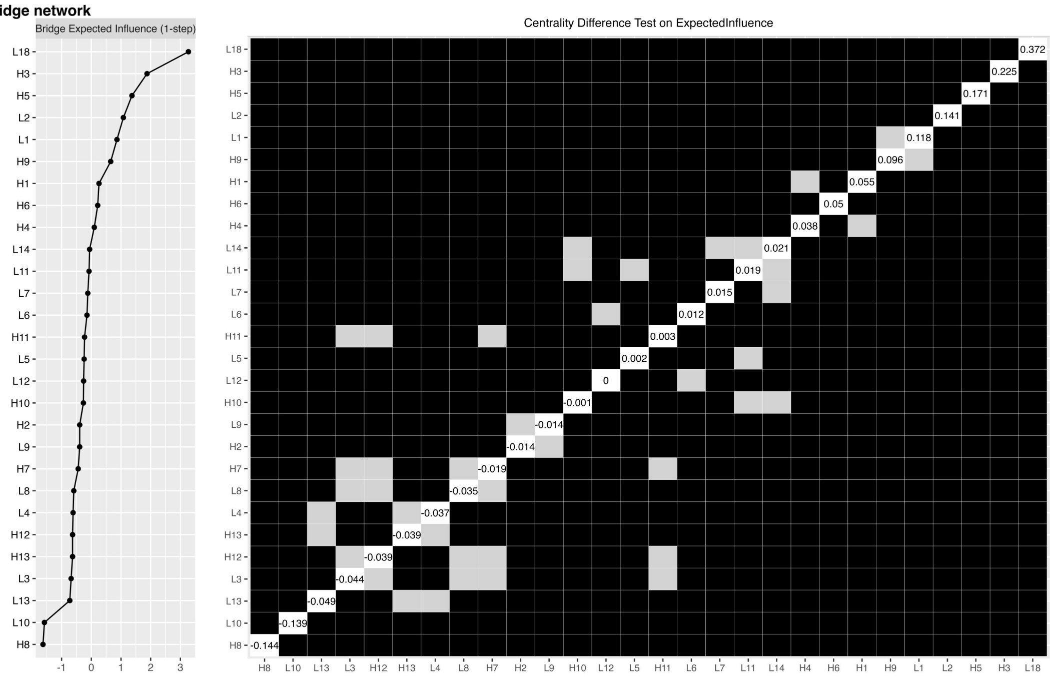

# (Q). Mexico

**Lifestyle network**

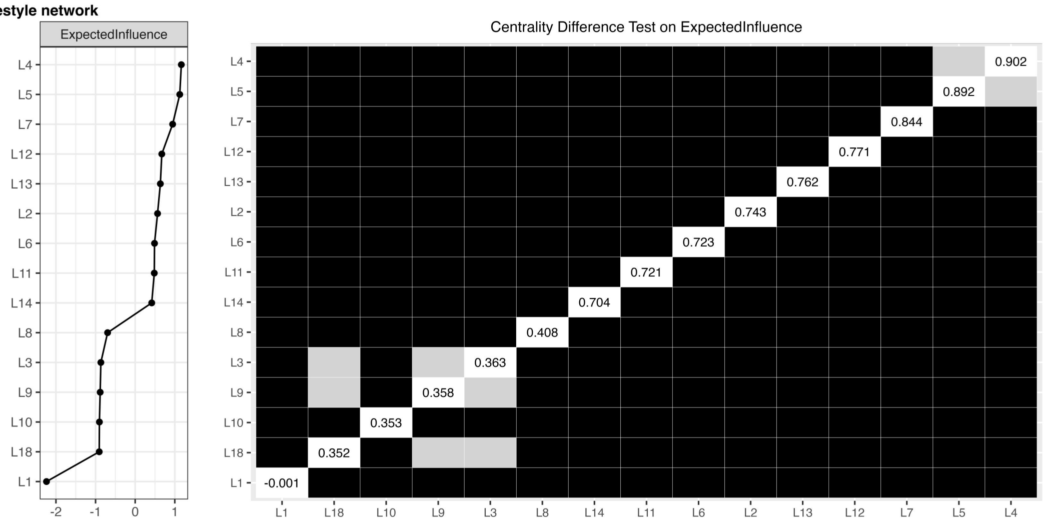


**Health outcome network**

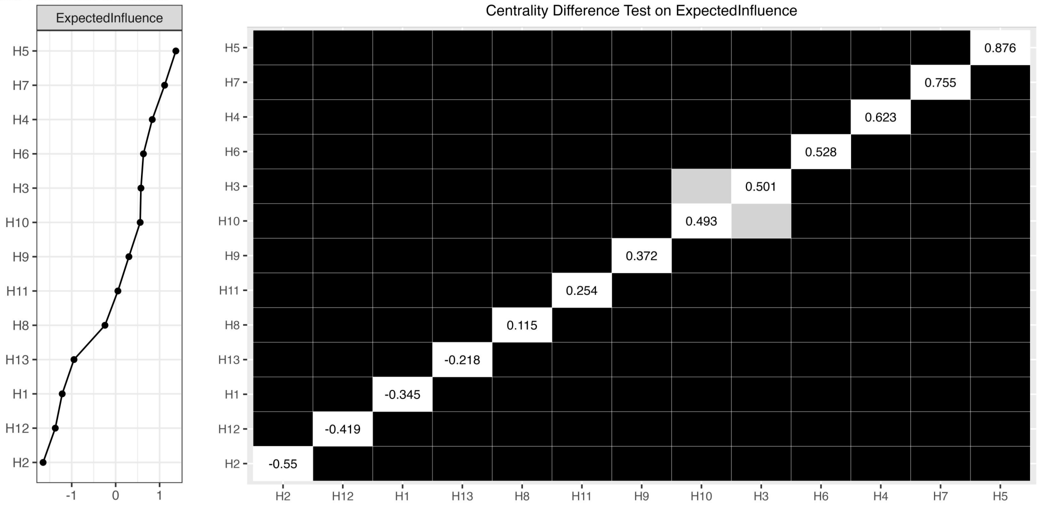


**Bridge network**

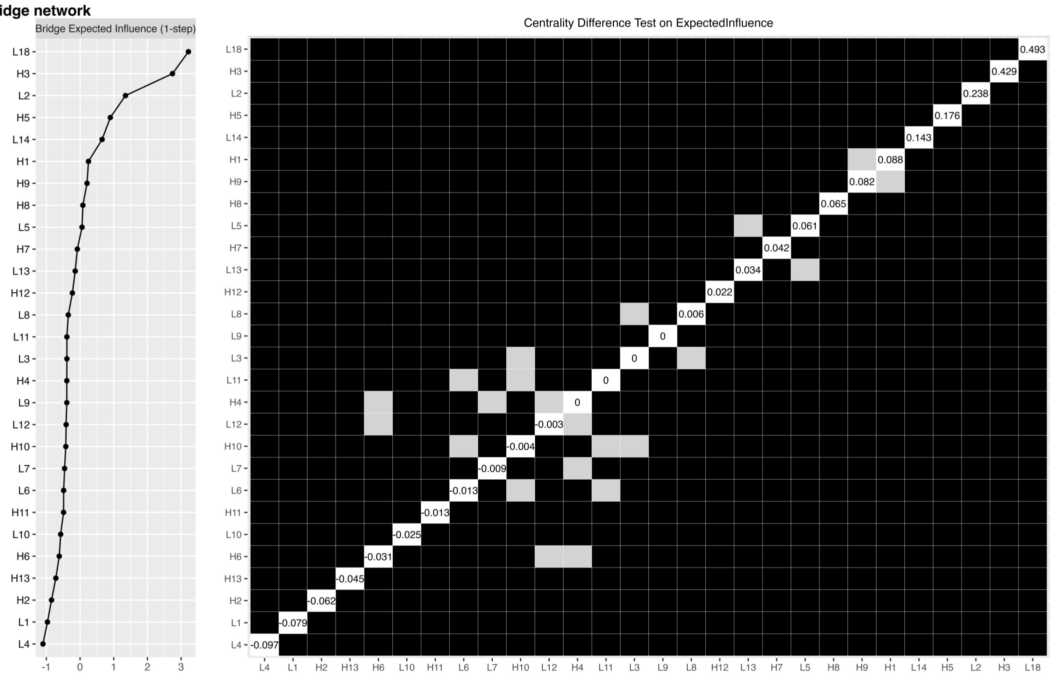

# (R). Nigeria

**Lifestyle network**

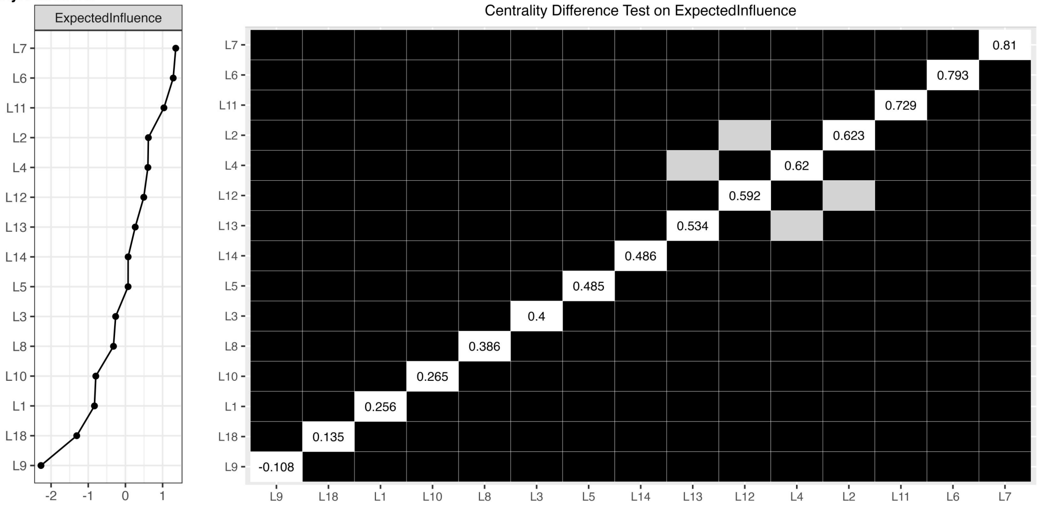


**Health outcome network**

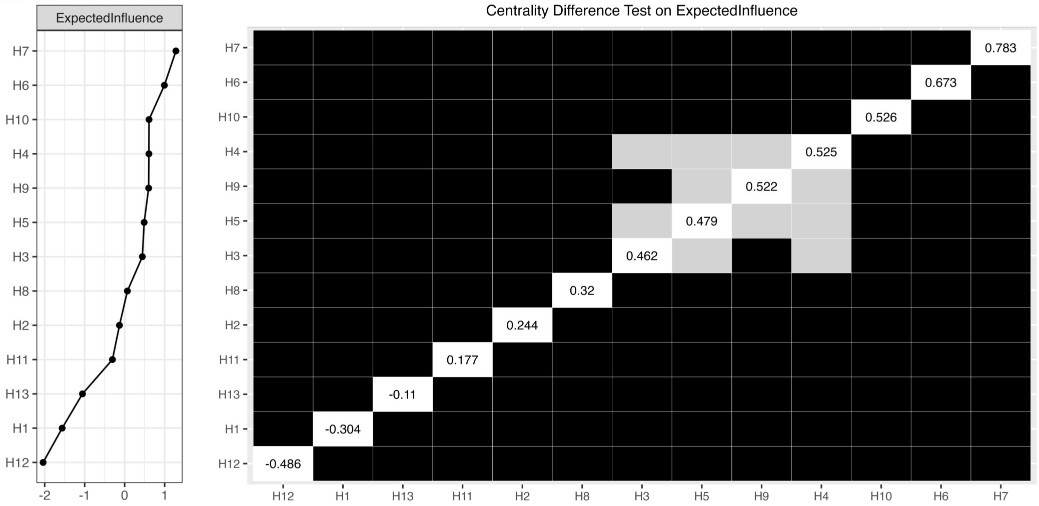


**Bridge network**

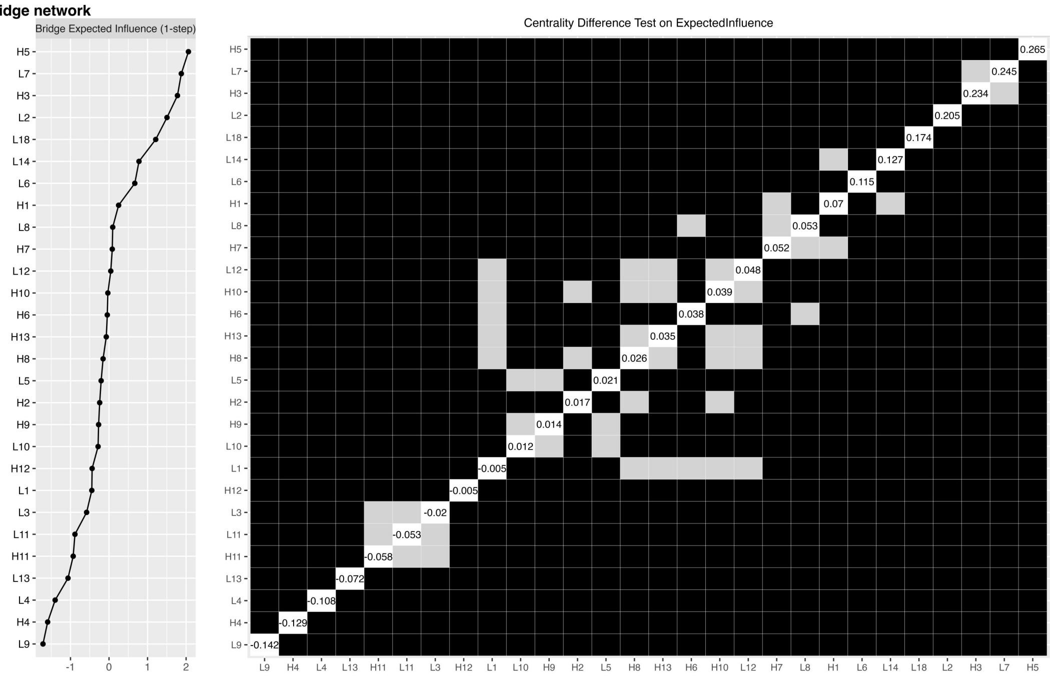

# (S). Philippines

**Lifestyle network**

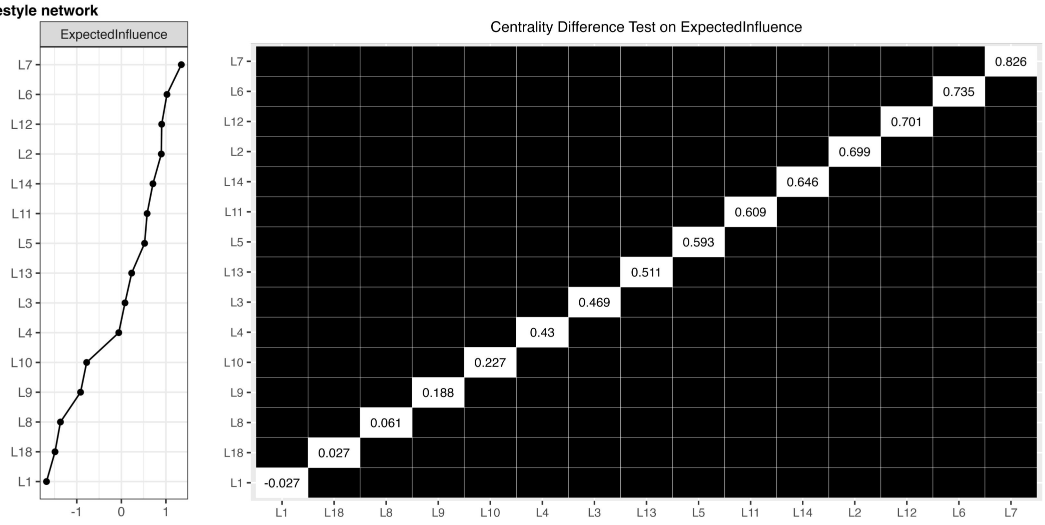


**Health outcome network**

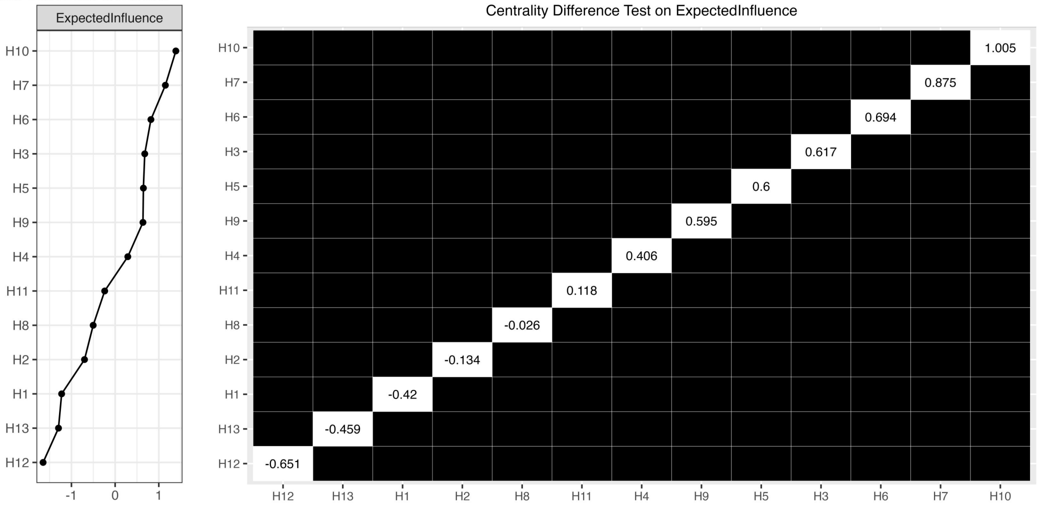


**Bridge network**

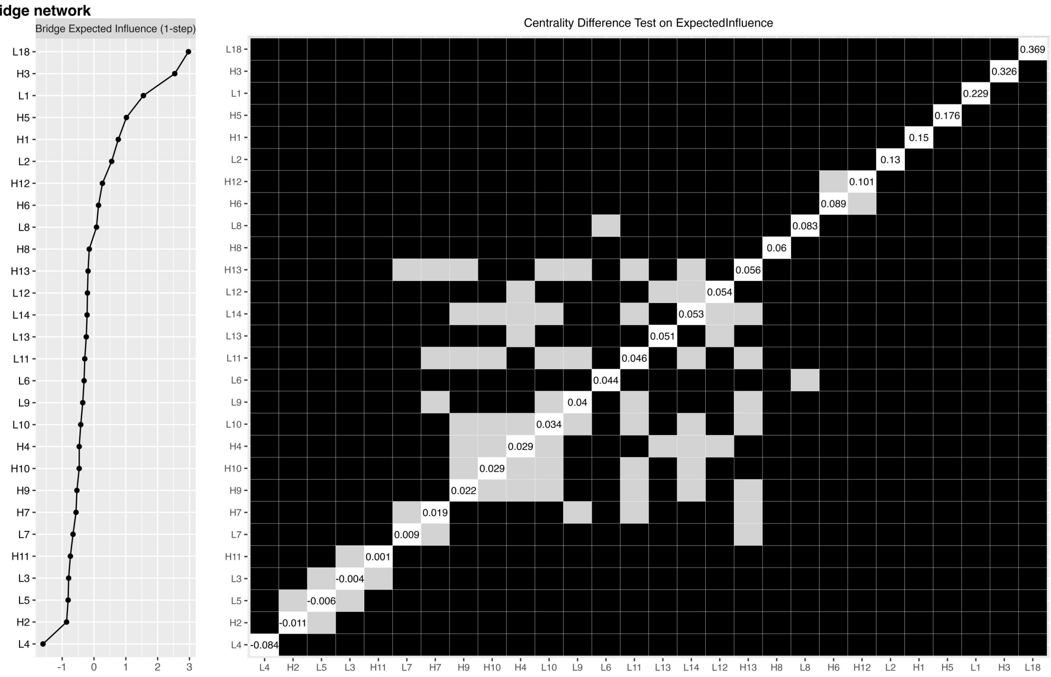

# (T). Republic of Sudan

**Lifestyle network**

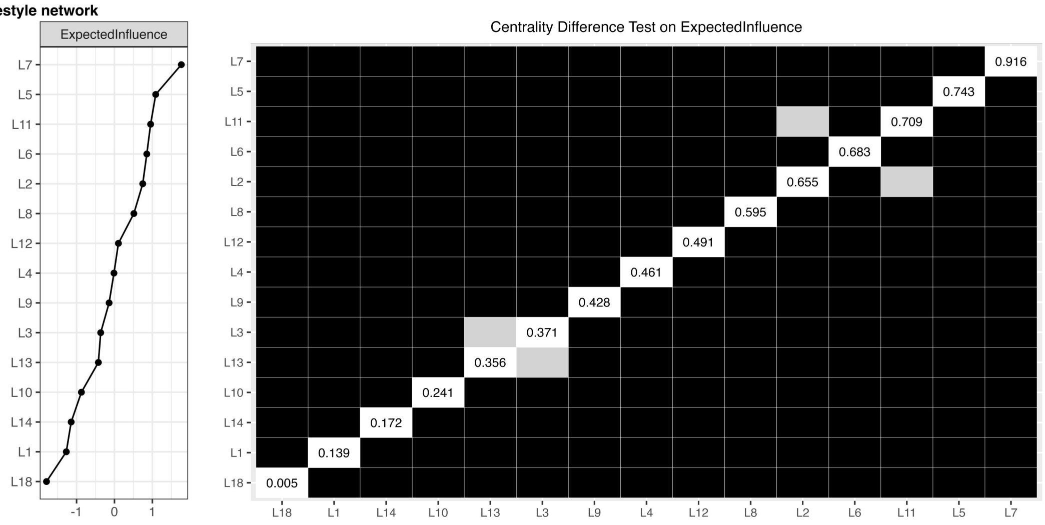


**Health outcome network**

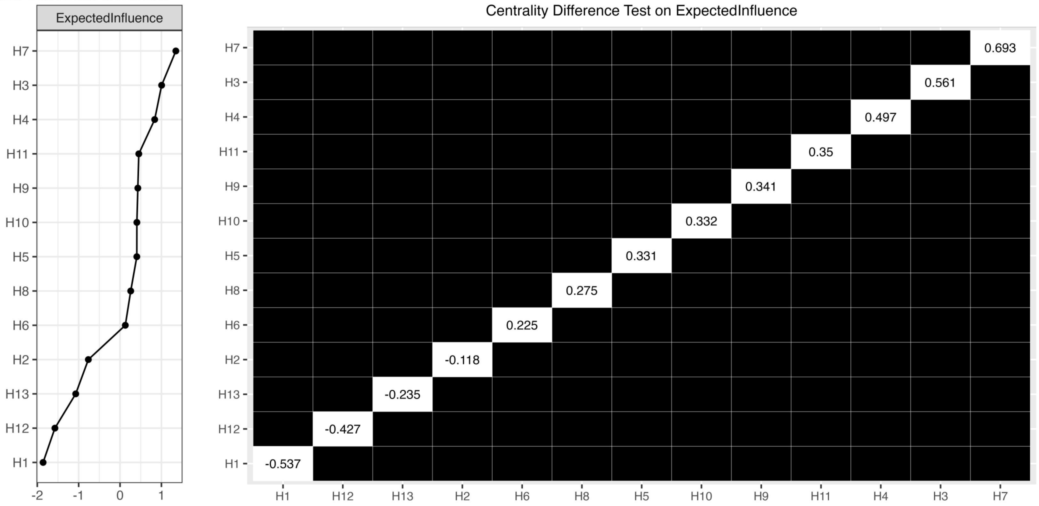


**Bridge network**

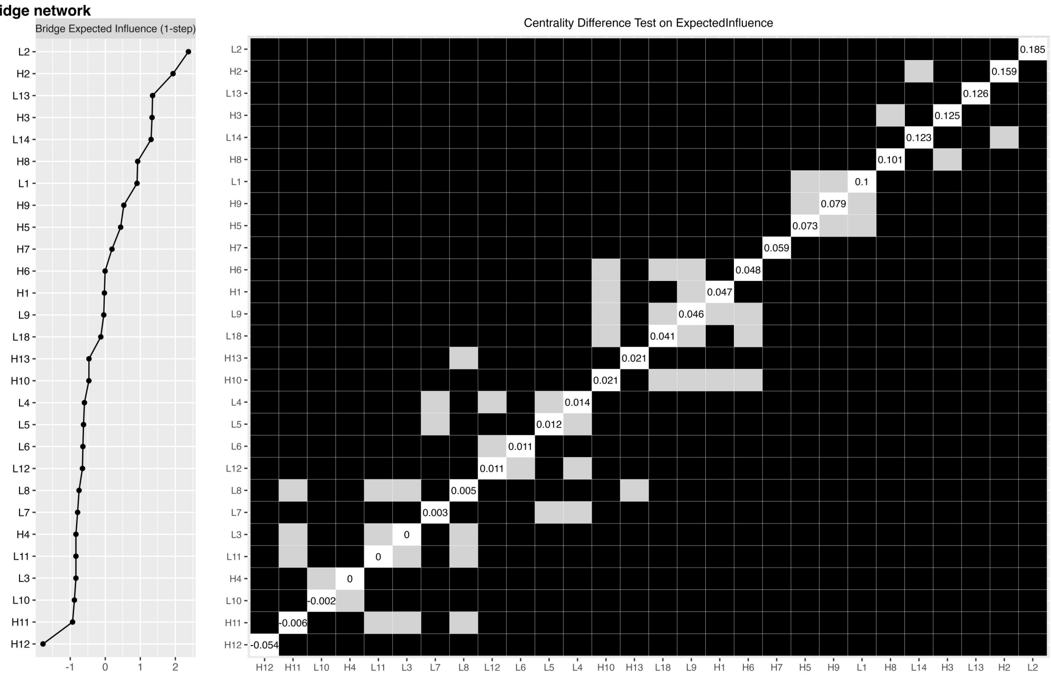

# (U). Rwanda

**Lifestyle network**

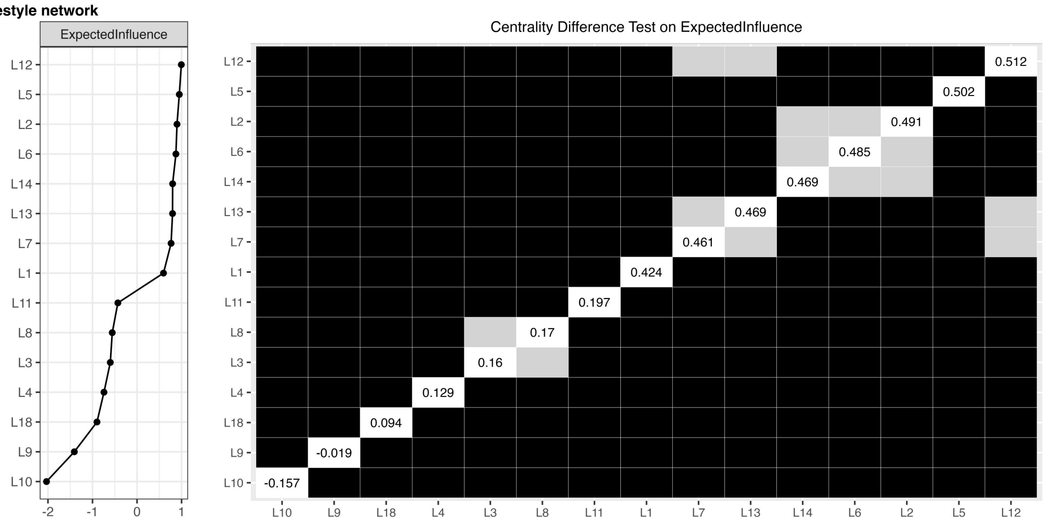


**Health outcome network**

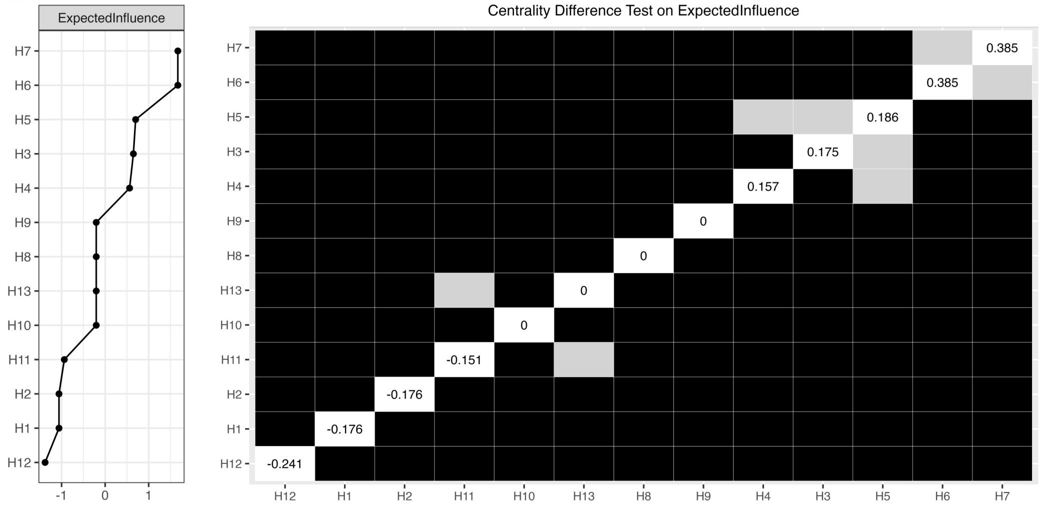


**Bridge network**

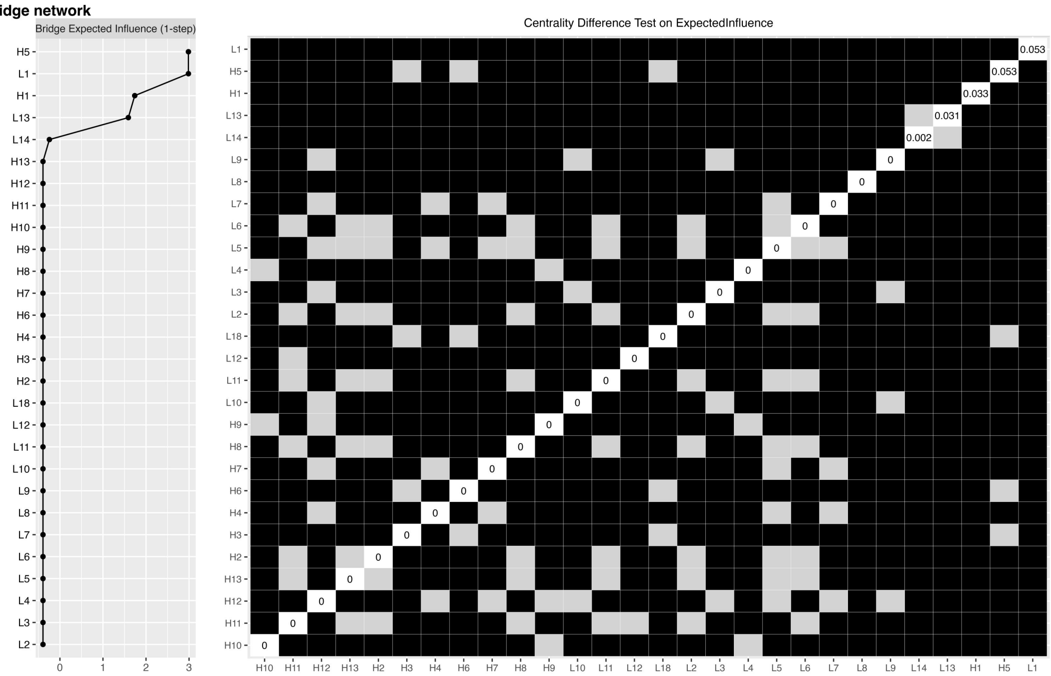

# (V). Saudi Arabia

**Lifestyle network**

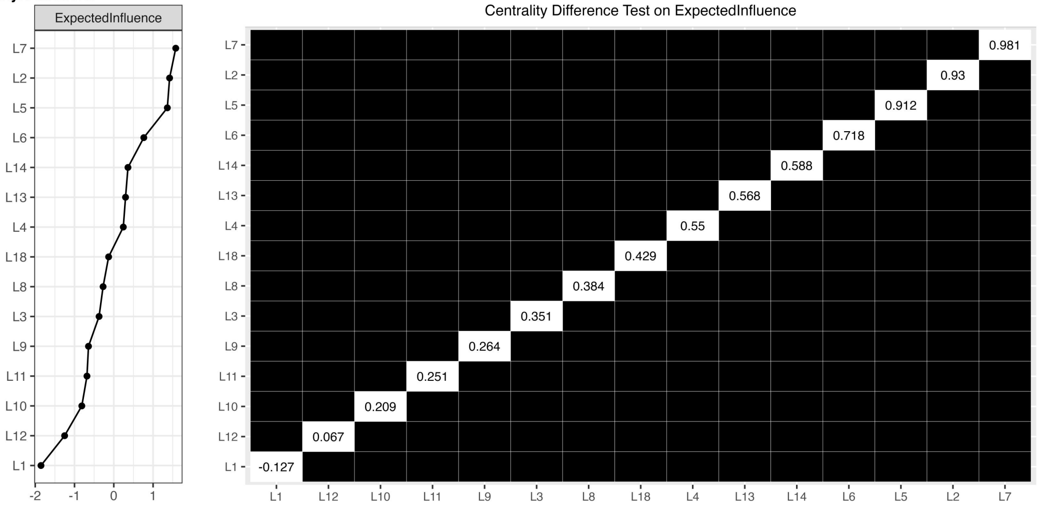


**Health outcome network**

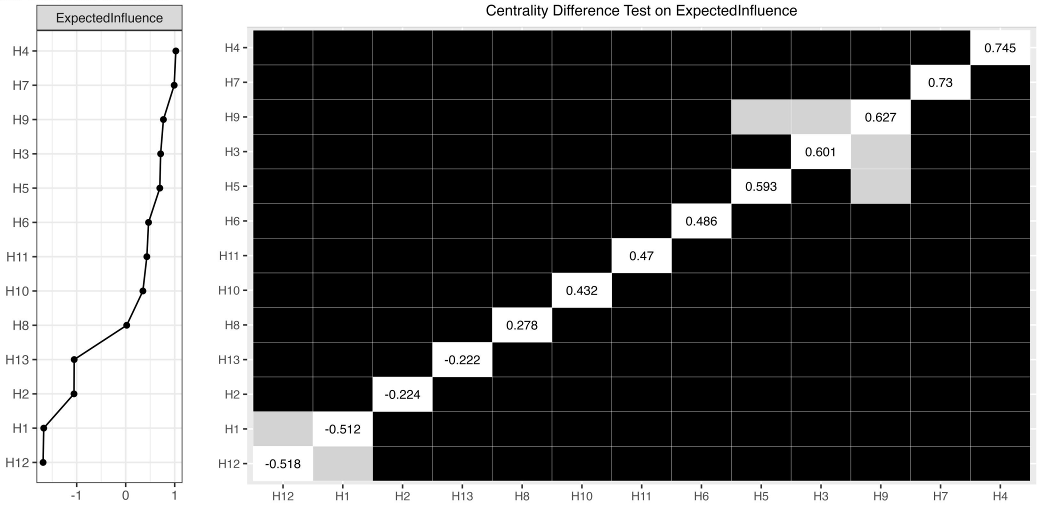


**Bridge network**

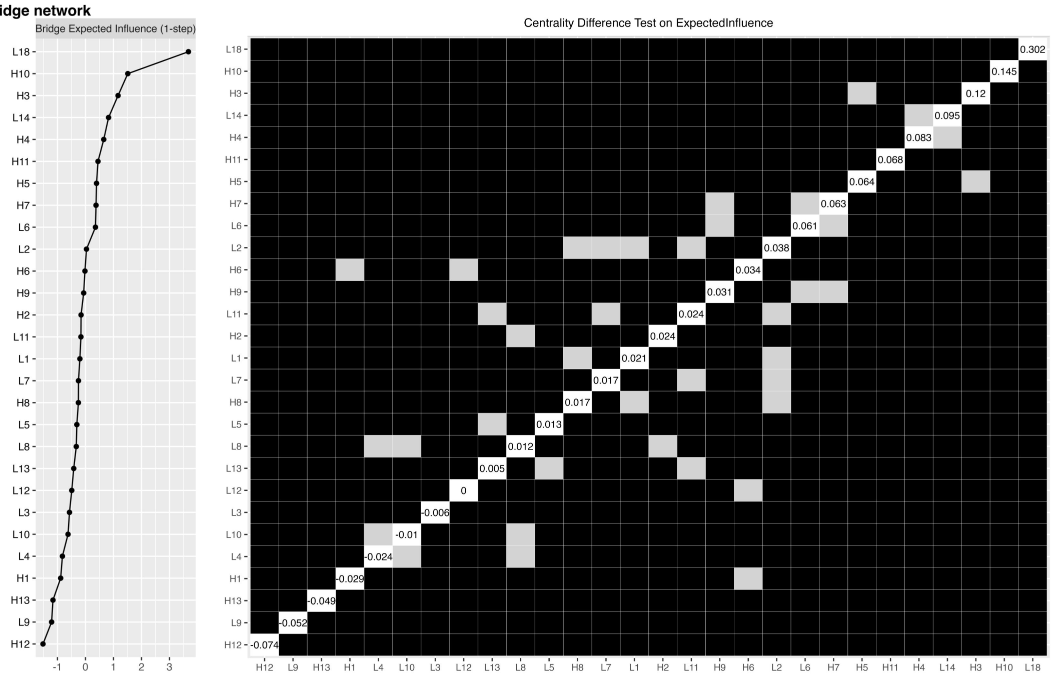

# (W). Singapore

**Lifestyle network**

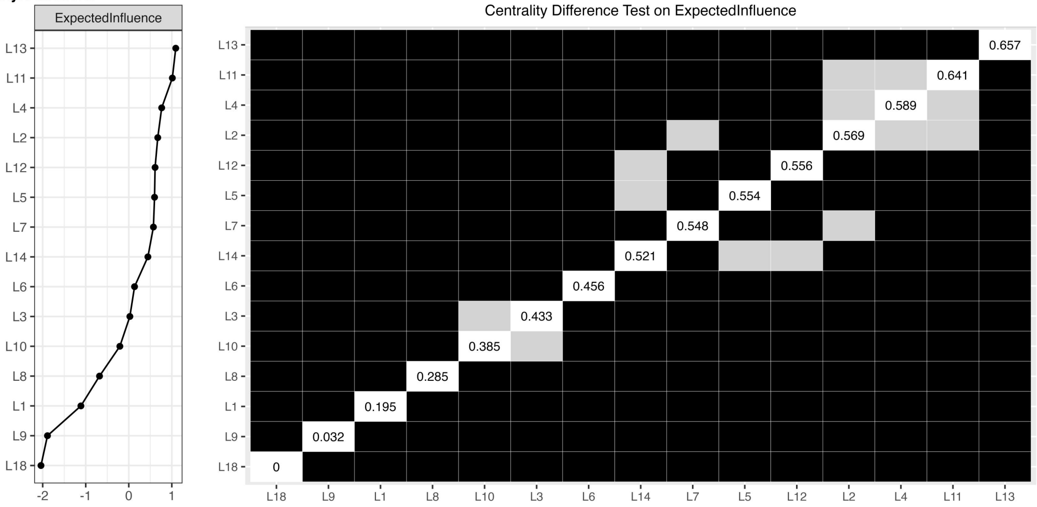


**Health outcome network**

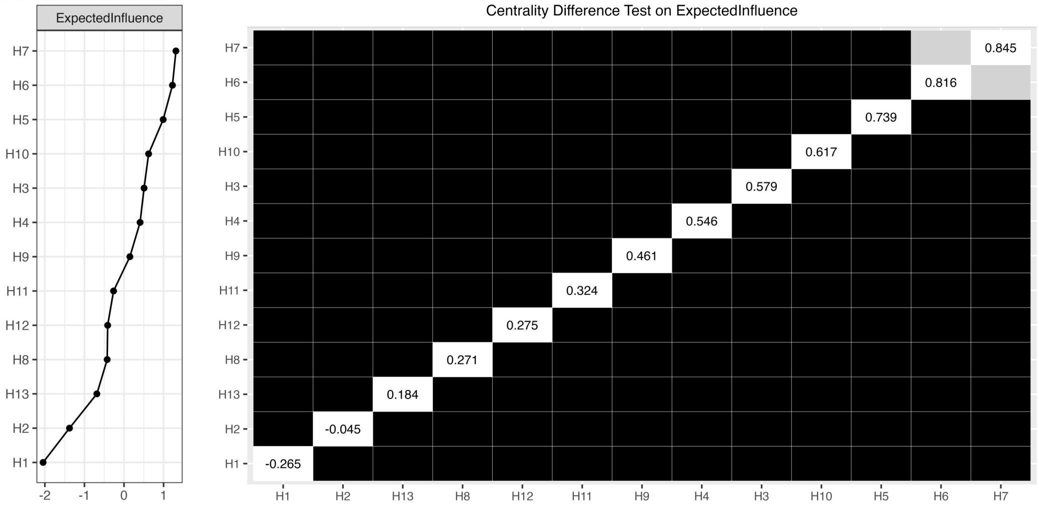


**Bridge network**

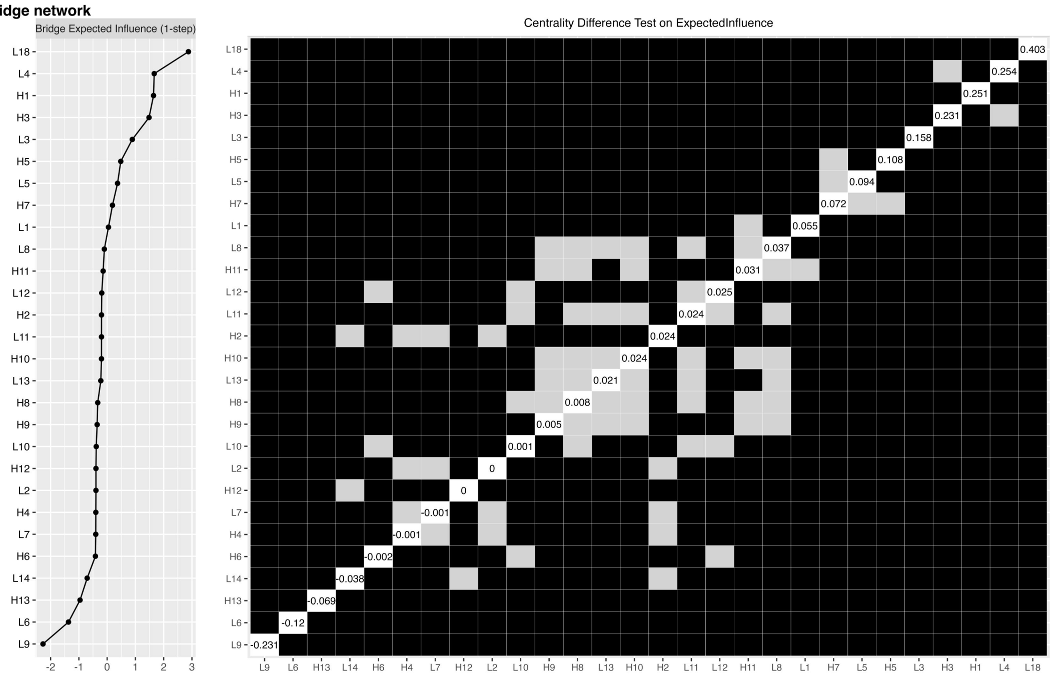

# (X). South Africa

**Lifestyle network**

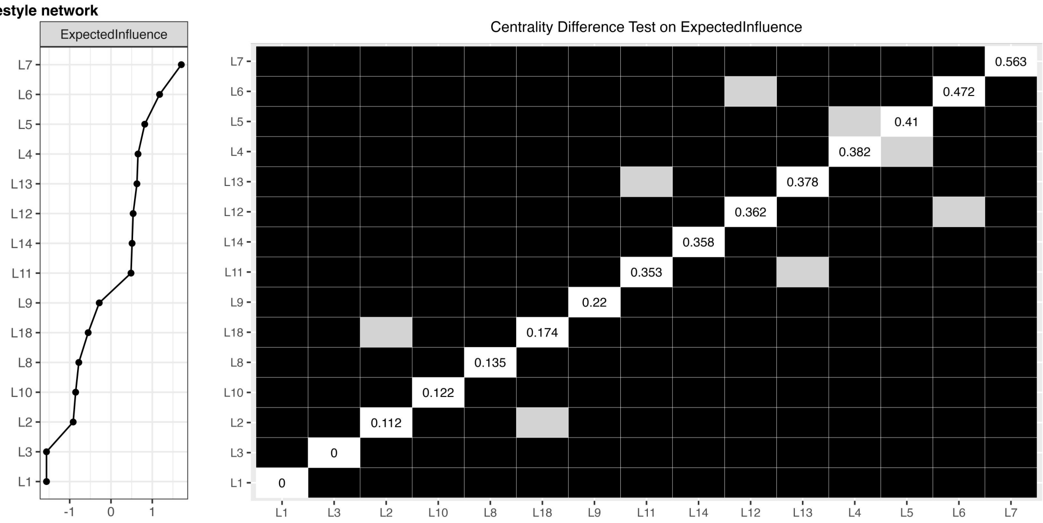


**Health outcome network**

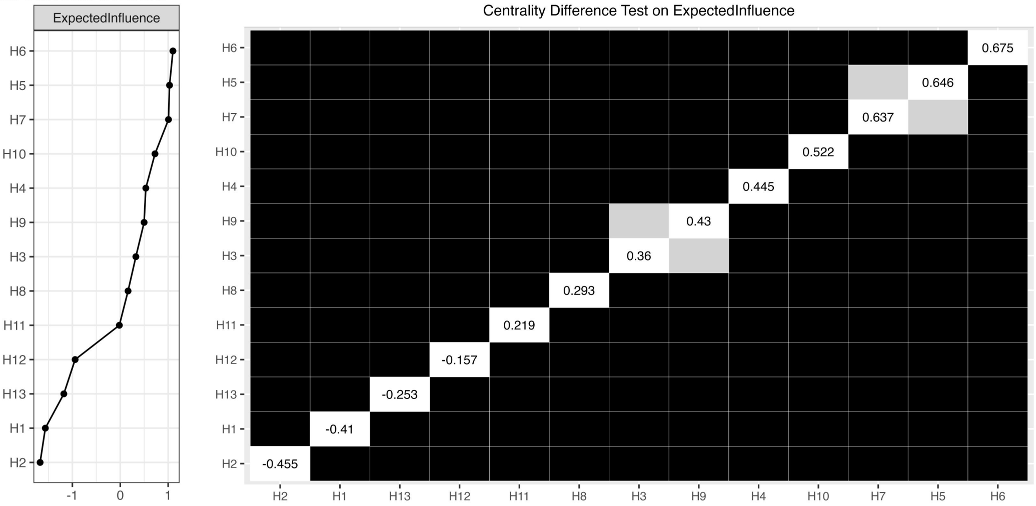


**Bridge network**

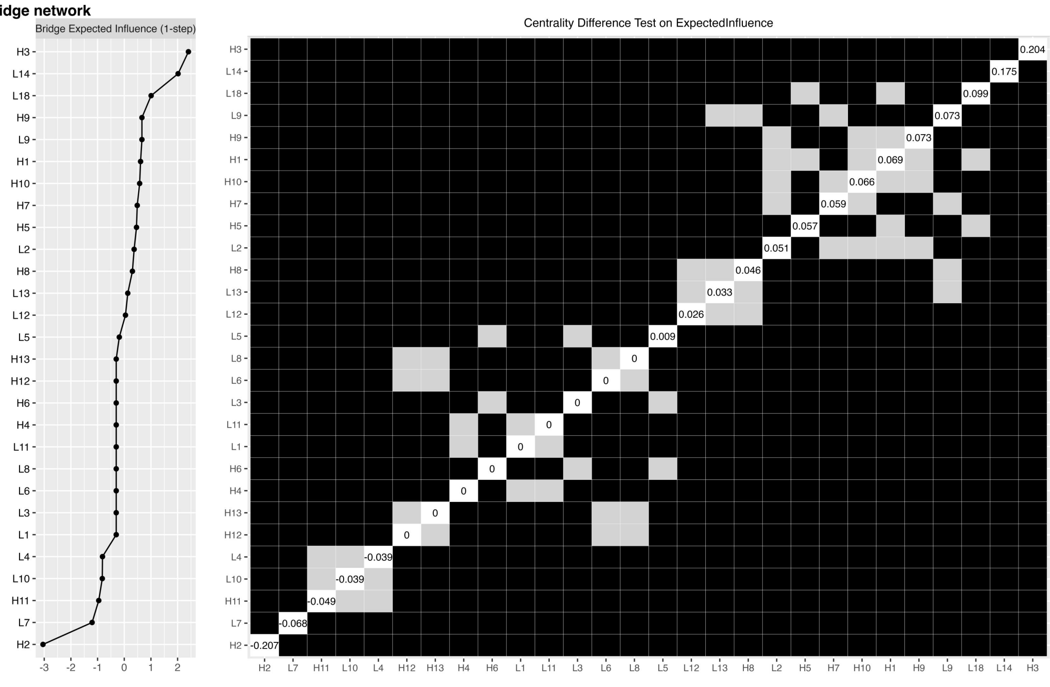

# (Y). South Korea

**Lifestyle network**

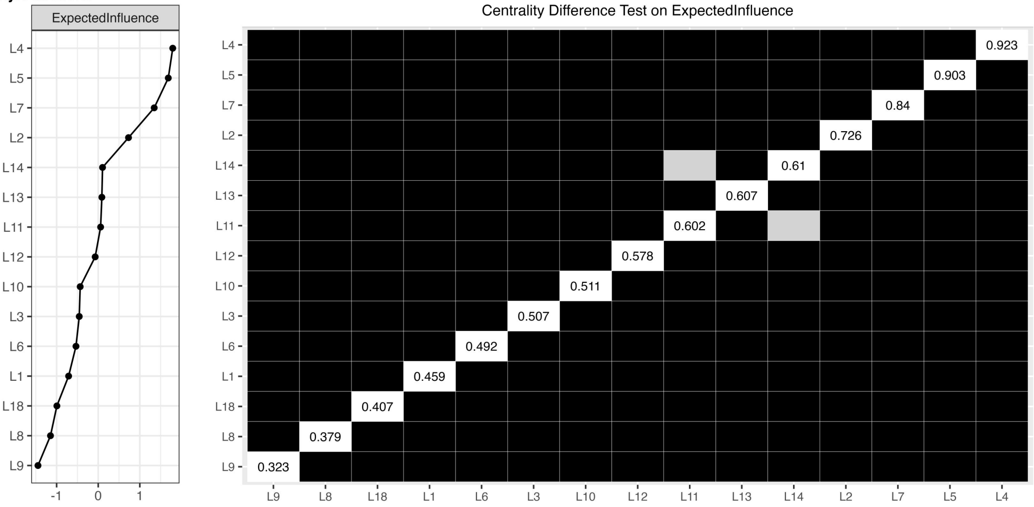


**Health outcome network**

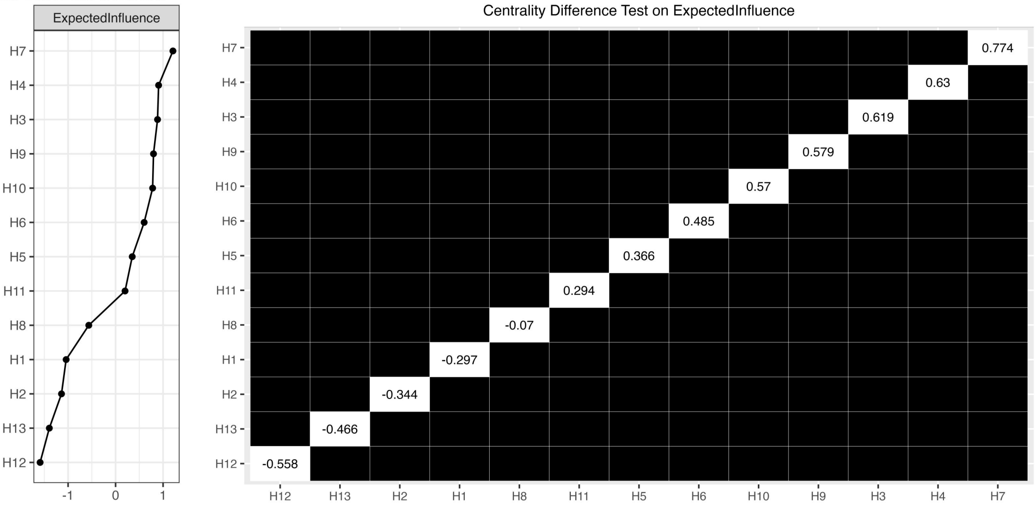


**Bridge network**

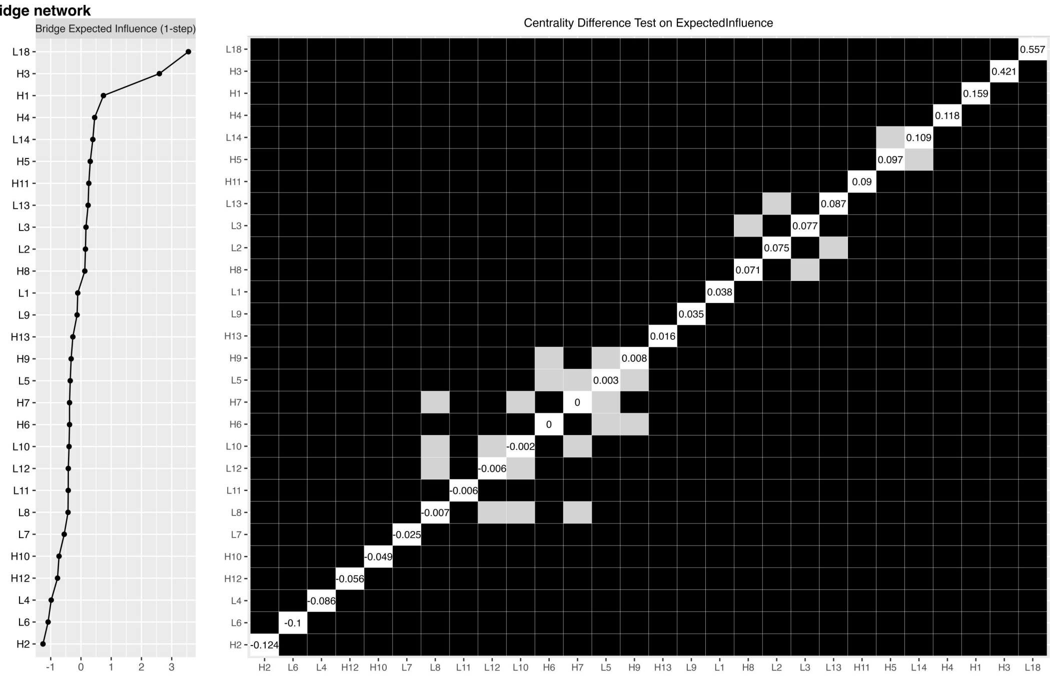

# (Z). Thailand

**Lifestyle network**

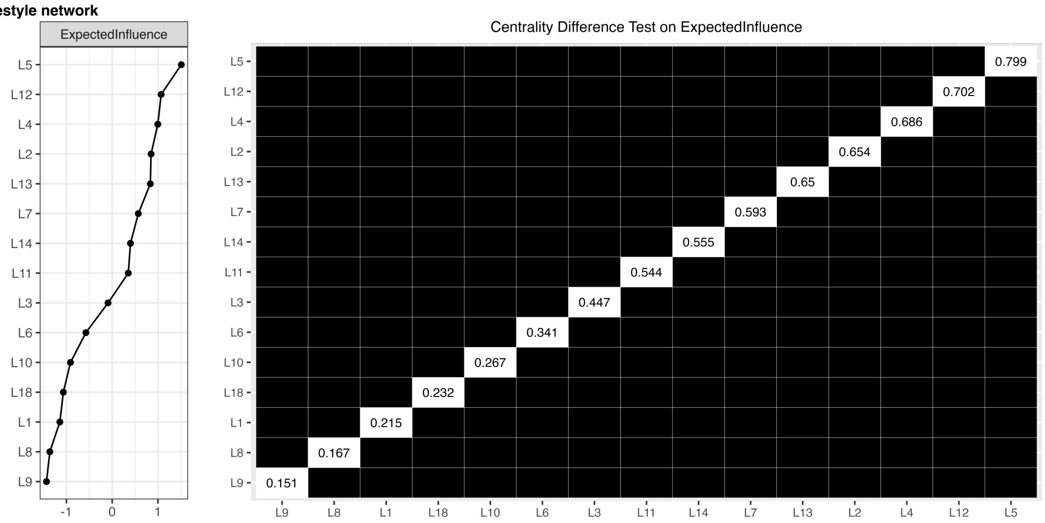


**Health outcome network**

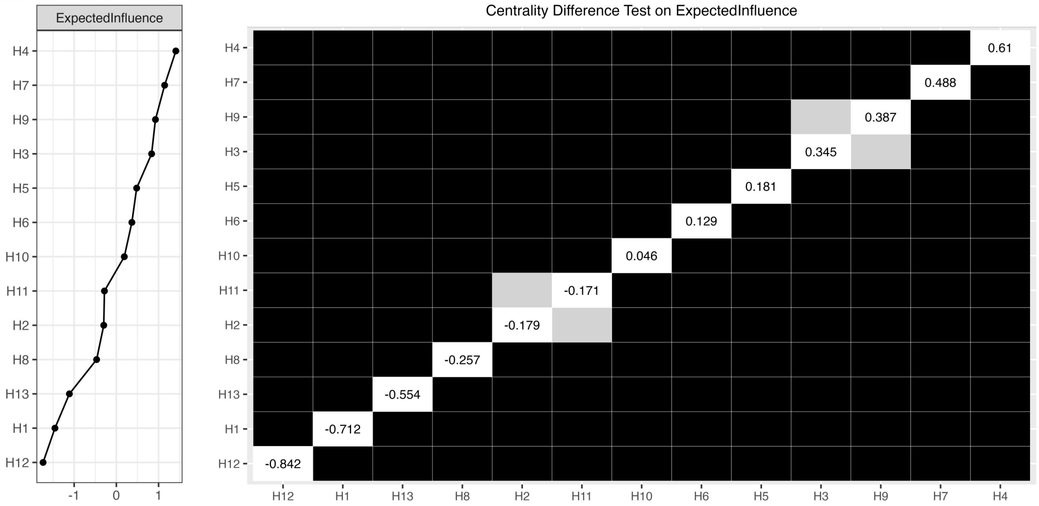


**Bridge network**

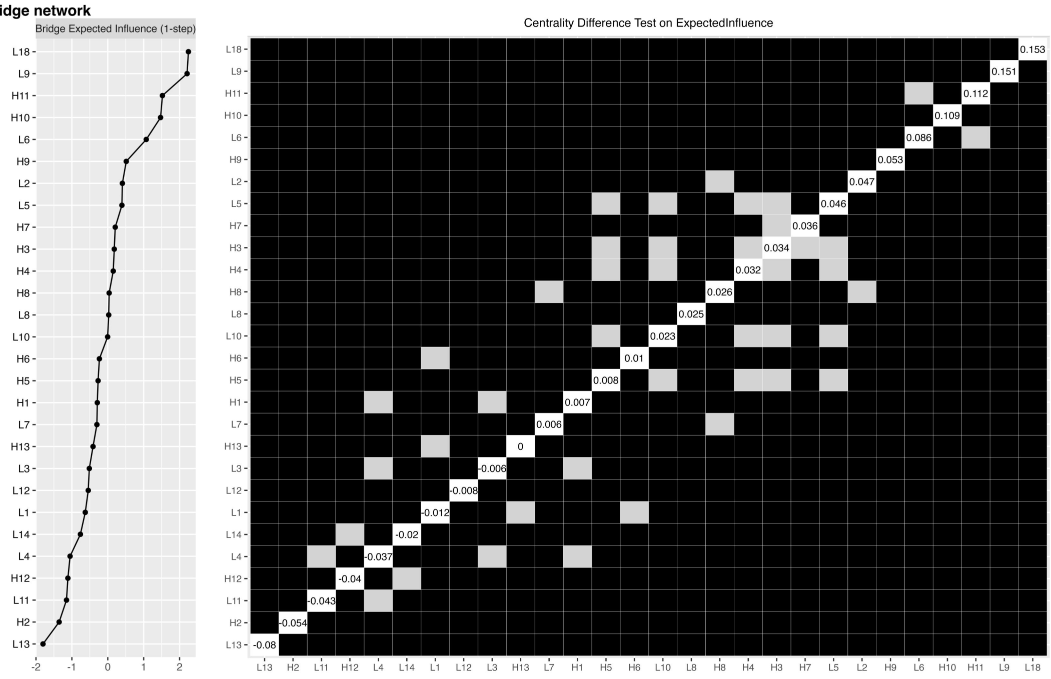

## (AA). United Kingdom

**Lifestyle network**

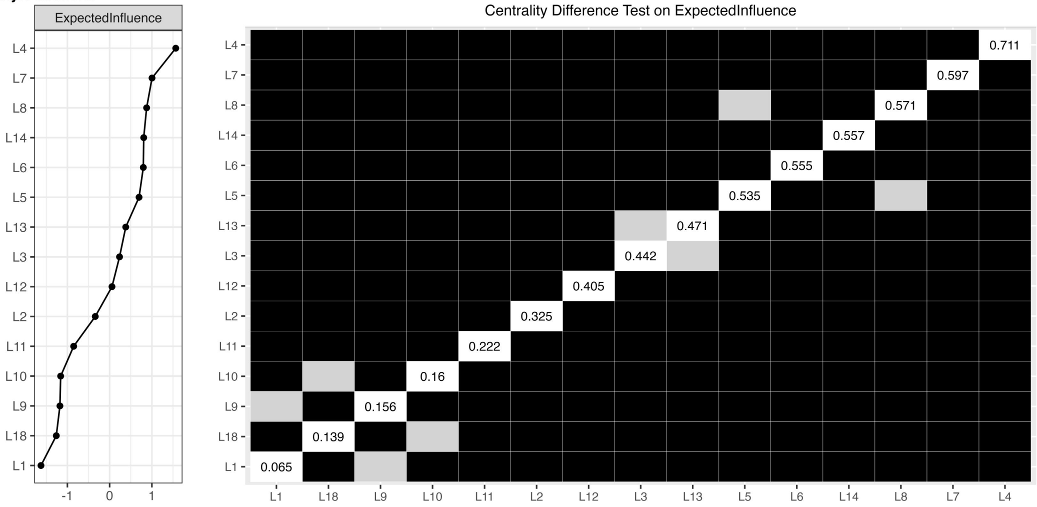


**Health outcome network**

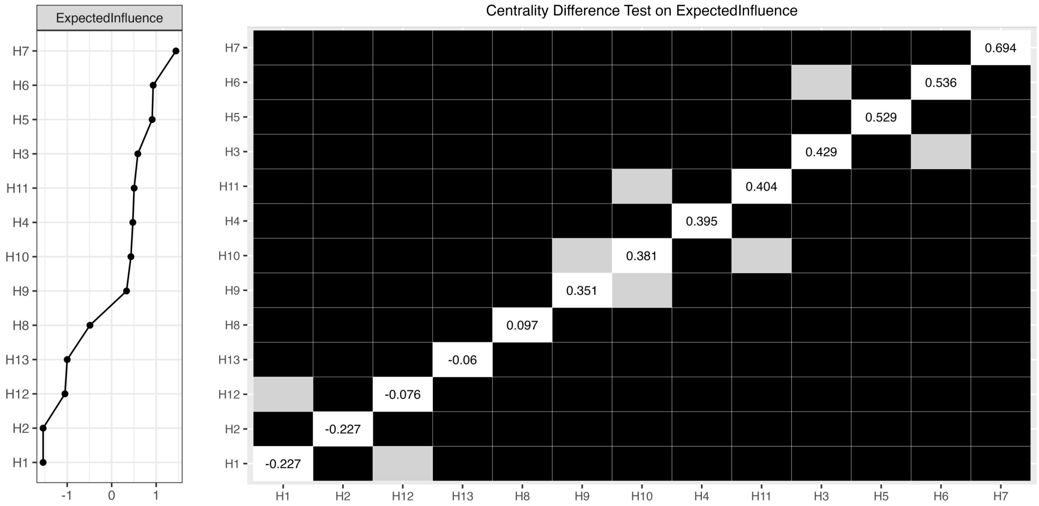


**Bridge network**

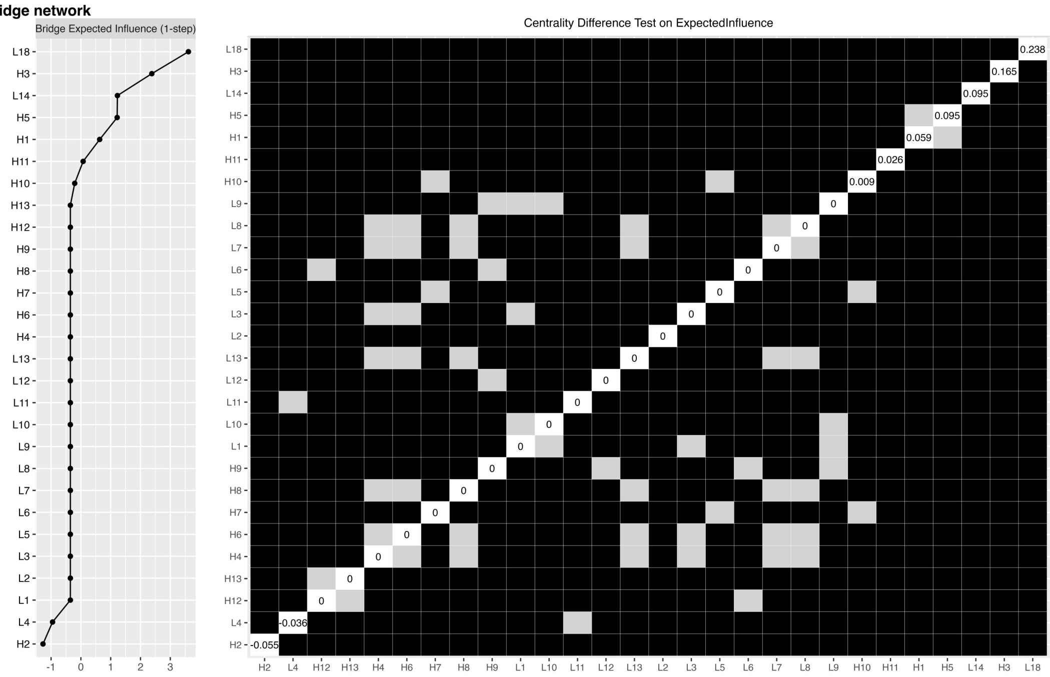

## (AB). United States

**Lifestyle network**

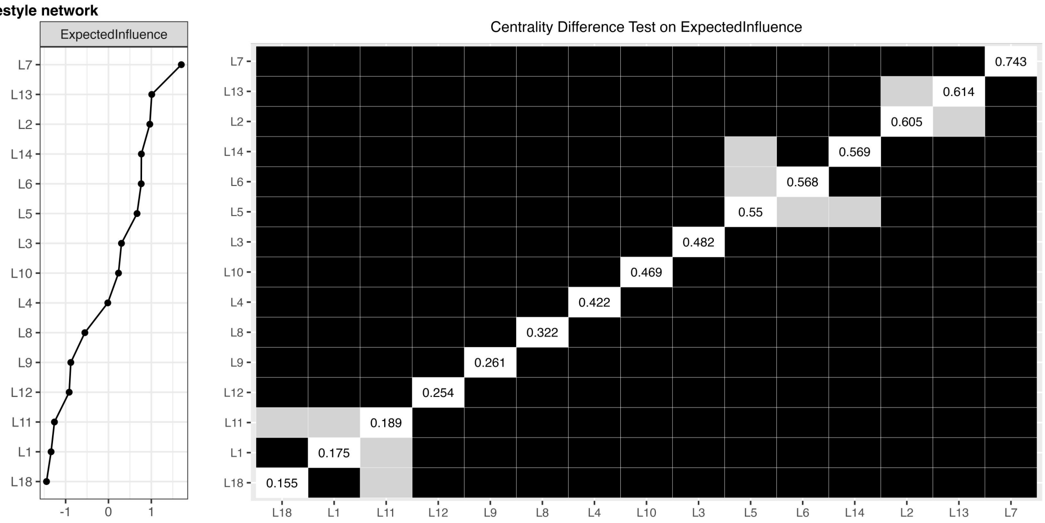


**Health outcome network**

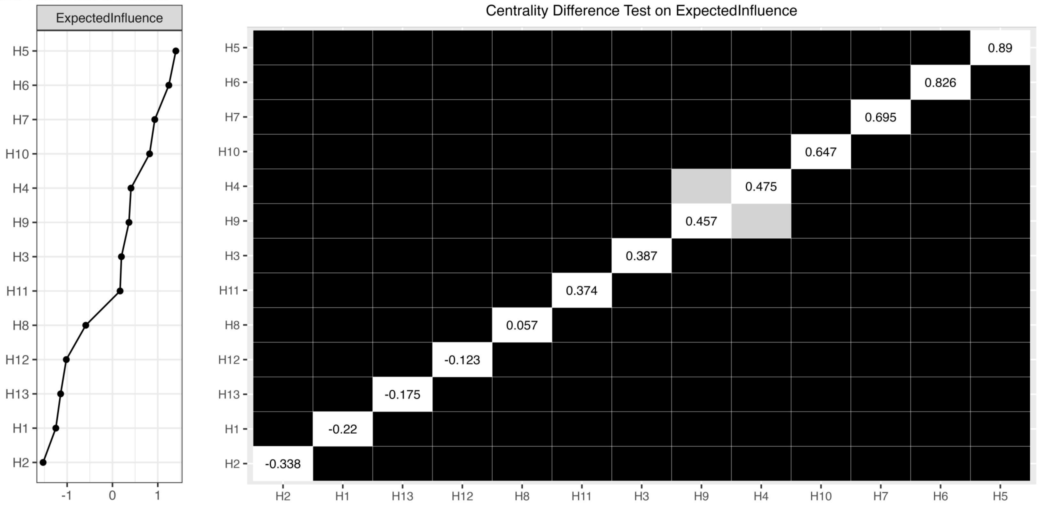


**Bridge network**

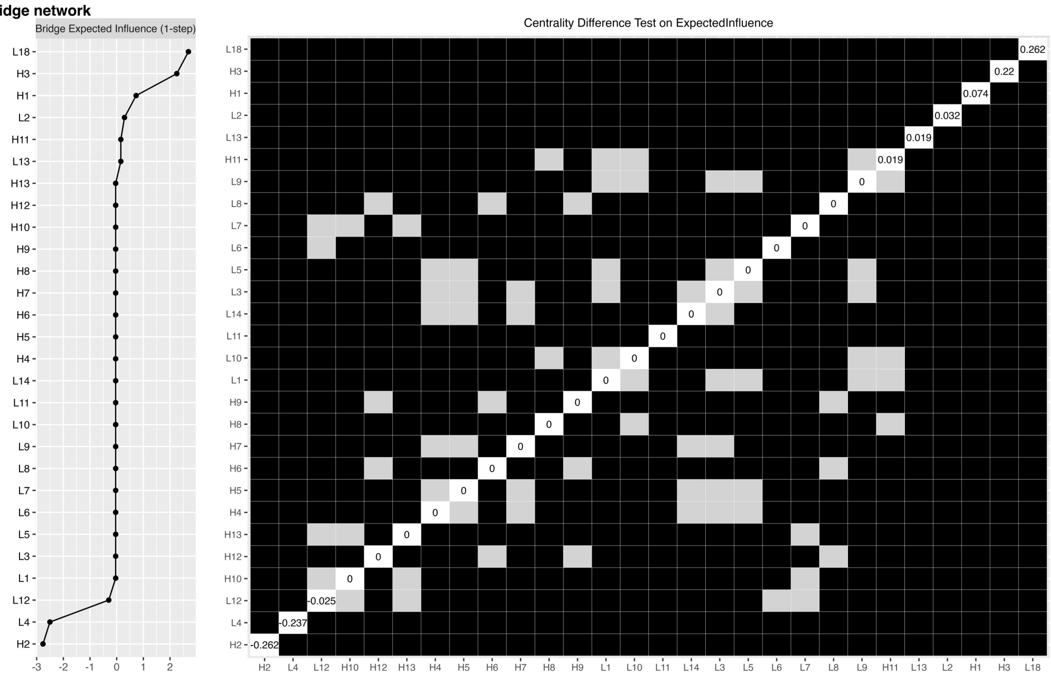

# (AC). Vietnam

**Lifestyle network**

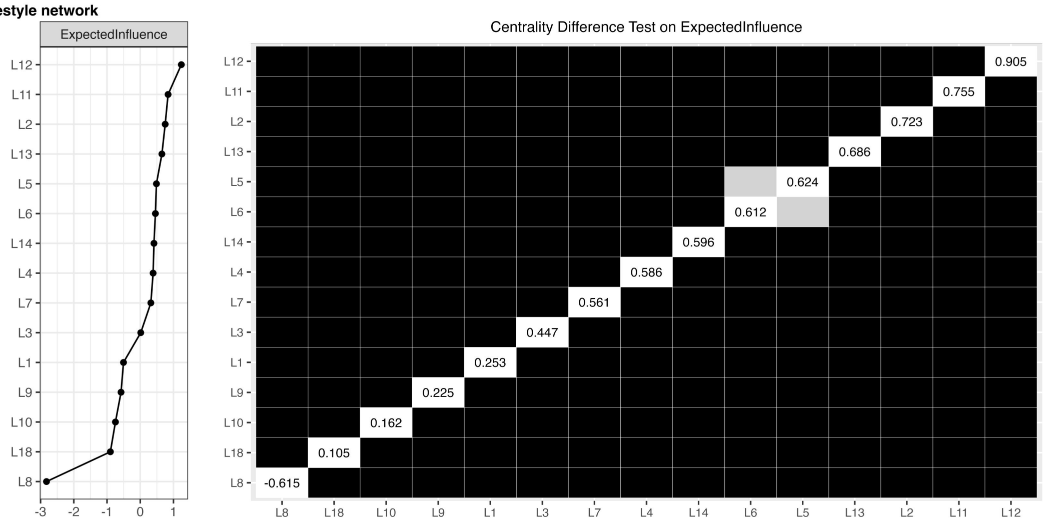


**Health outcome network**

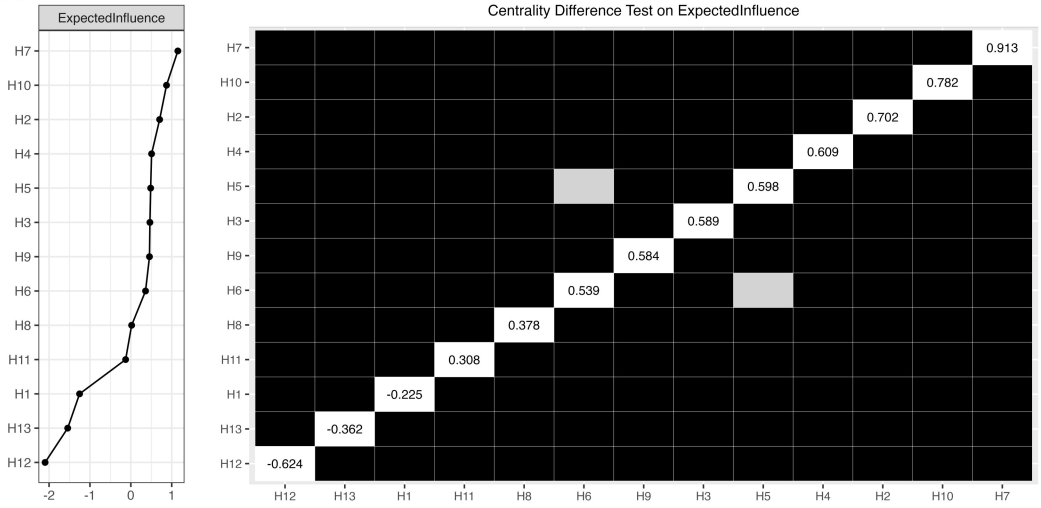


**Bridge network**

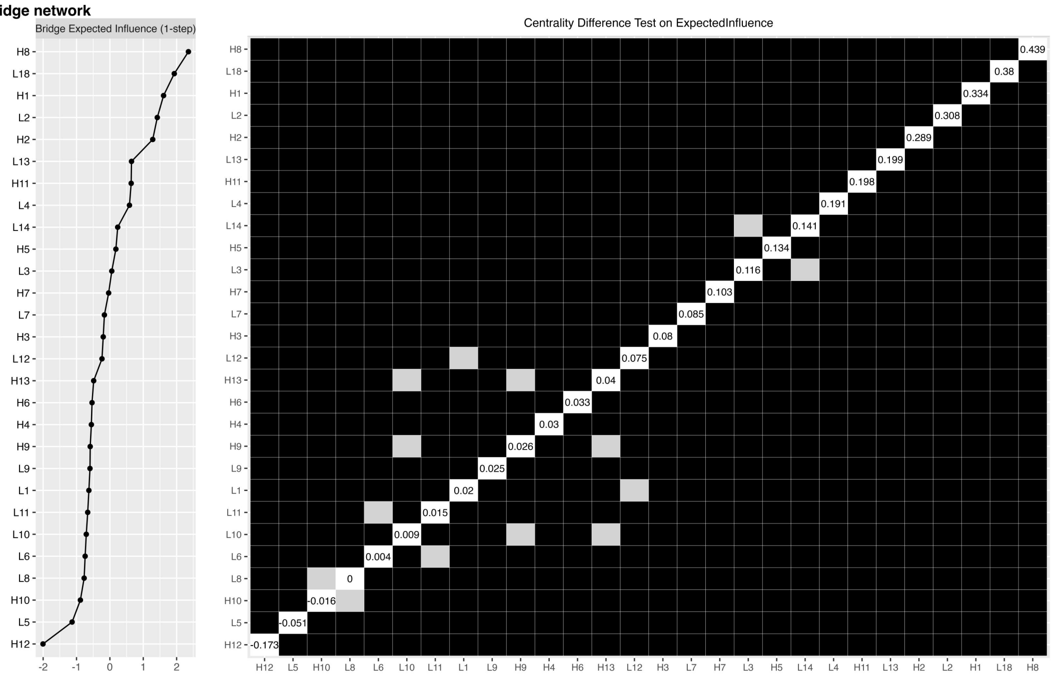

**Figure S5.** Bootstrapped confidence intervals of edge weights for the health outcome network across 29 countries.

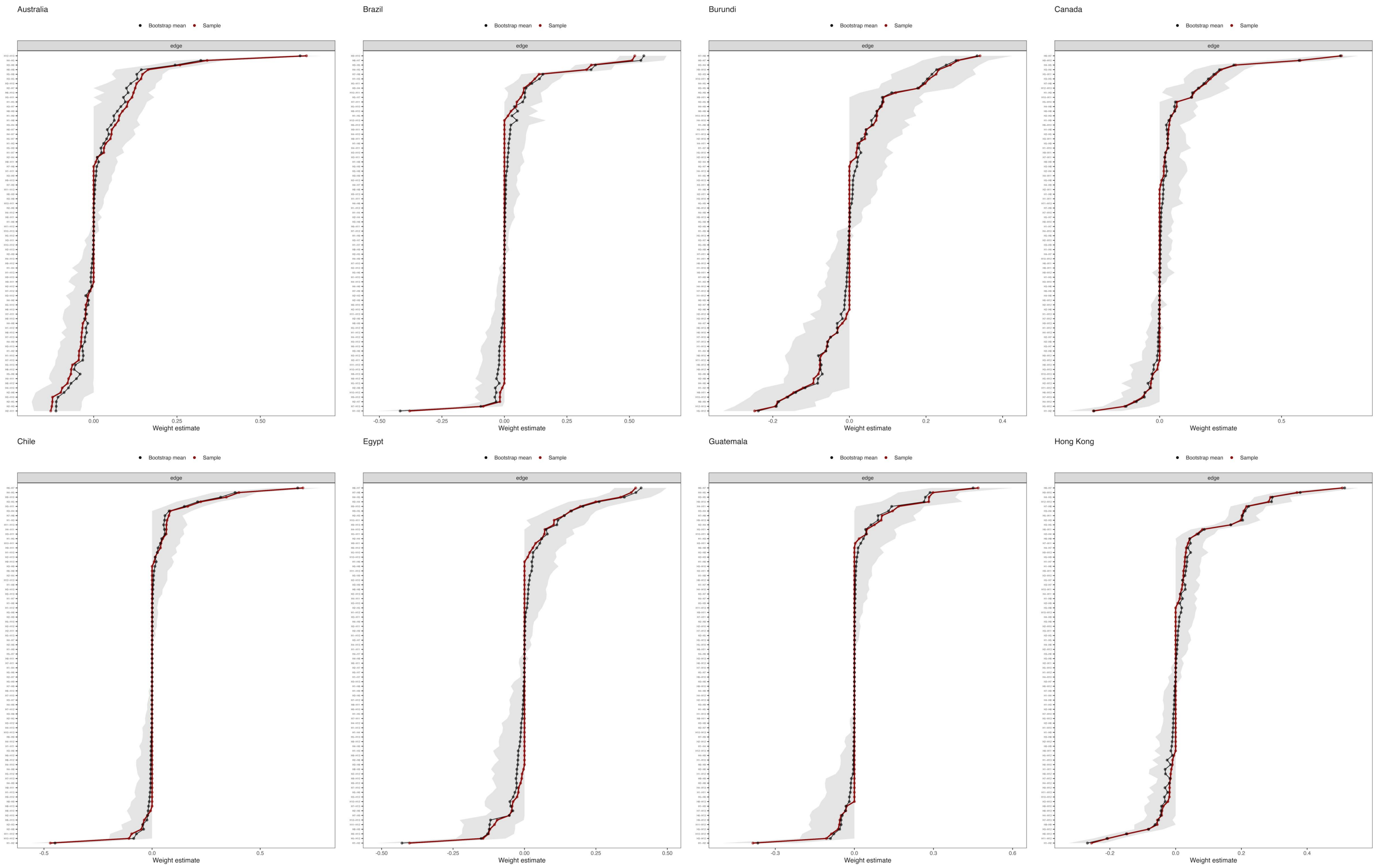

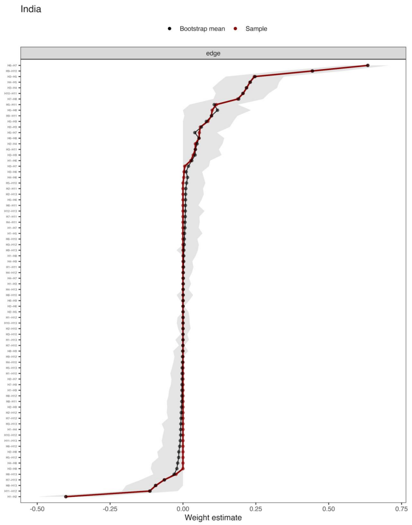
India
Bootstrap mean
Sample
edge
-0.50
-0.25
0.00
0.25
0.50
0.75
Weight estimate

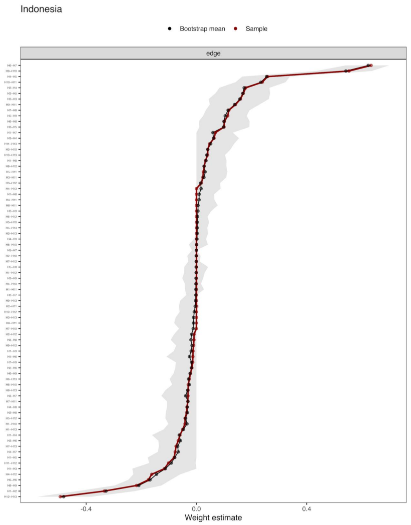
Indonesia
Bootstrap mean
Sample
edge
-0.4
0.0
0.4
Weight estimate

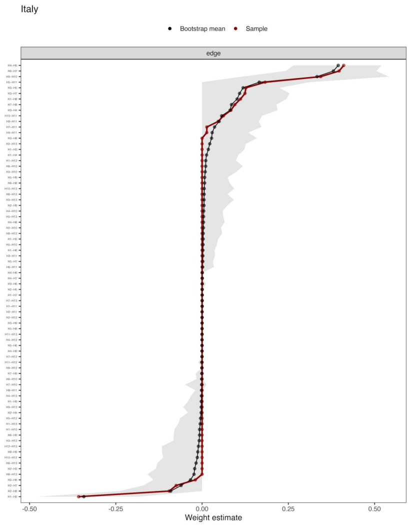
Italy
Bootstrap mean
Sample
edge
-0.50
-0.25
0.00
0.25
0.50
Weight estimate

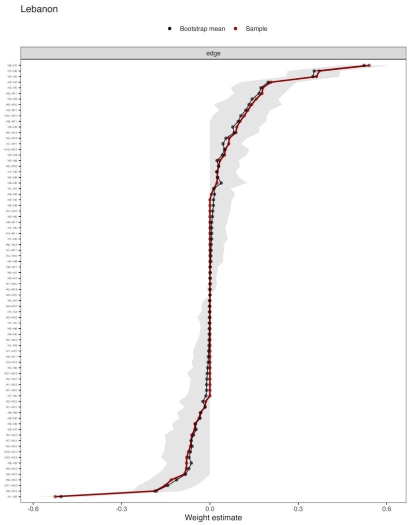
Lebanon
Bootstrap mean
Sample
edge
-0.6
-0.3
0.0
0.3
0.6
Weight estimate

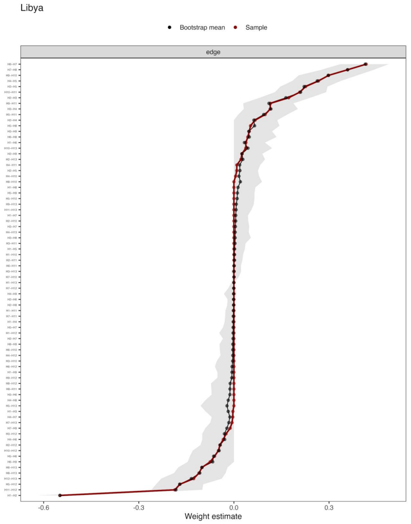
Libya
Bootstrap mean
Sample
edge
-0.6
-0.3
0.0
0.3
Weight estimate

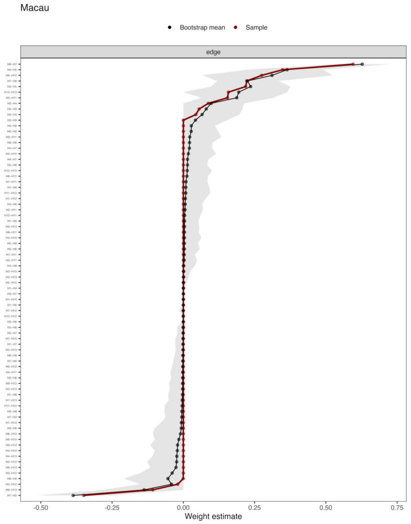
Macau
Bootstrap mean
Sample
edge
-0.50
-0.25
0.00
0.25
0.50
0.75
Weight estimate

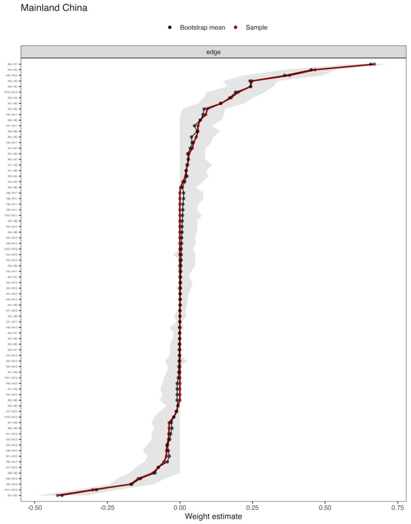
Mainland China
Bootstrap mean
Sample
edge
-0.50
-0.25
0.00
0.25
0.50
0.75
Weight estimate

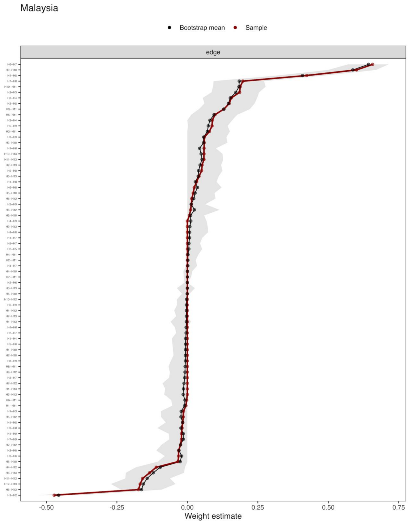
Malaysia
Bootstrap mean
Sample
edge
-0.50
-0.25
0.00
0.25
0.50
0.75
Weight estimate

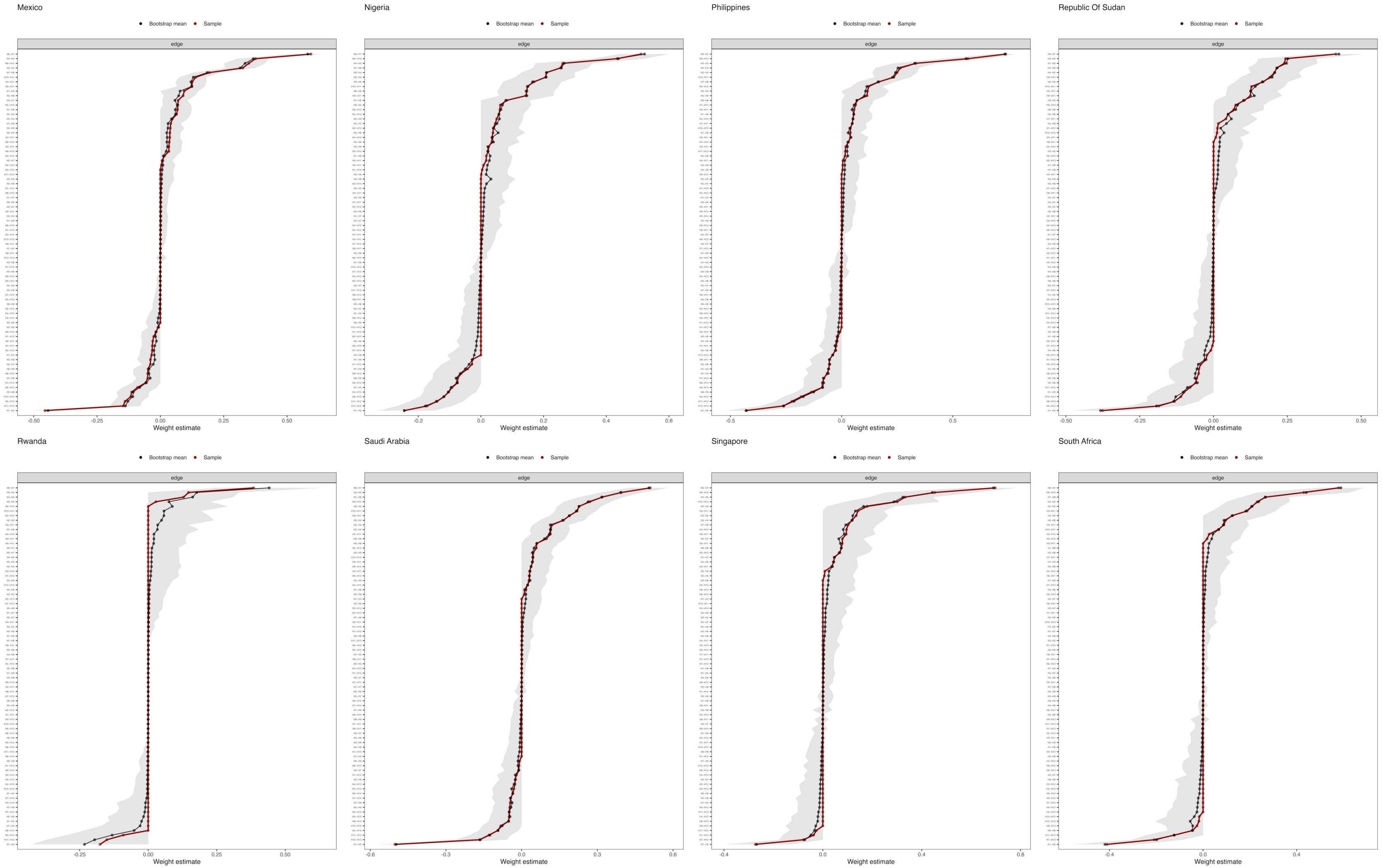
Mexico
Bootstrap mean
Sample
edge
-0.50 -0.25 0.00 0.25 0.50
Weight estimate
Nigeria
Bootstrap mean
Sample
edge
-0.2 0.0 0.2 0.4 0.6
Weight estimate
Philippines
Bootstrap mean
Sample
edge
-0.5 0.0 0.5
Weight estimate
Republic Of Sudan
Bootstrap mean
Sample
edge
-0.50 -0.25 0.00 0.25 0.50
Weight estimate
Rwanda
Bootstrap mean
Sample
edge
-0.25 0.00 0.25 0.50
Weight estimate
Saudi Arabia
Bootstrap mean
Sample
edge
-0.6 -0.3 0.0 0.3 0.6
Weight estimate
Singapore
Bootstrap mean
Sample
edge
-0.4 0.0 0.4 0.8
Weight estimate
South Africa
Bootstrap mean
Sample
edge
-0.4 0.0 0.4
Weight estimate

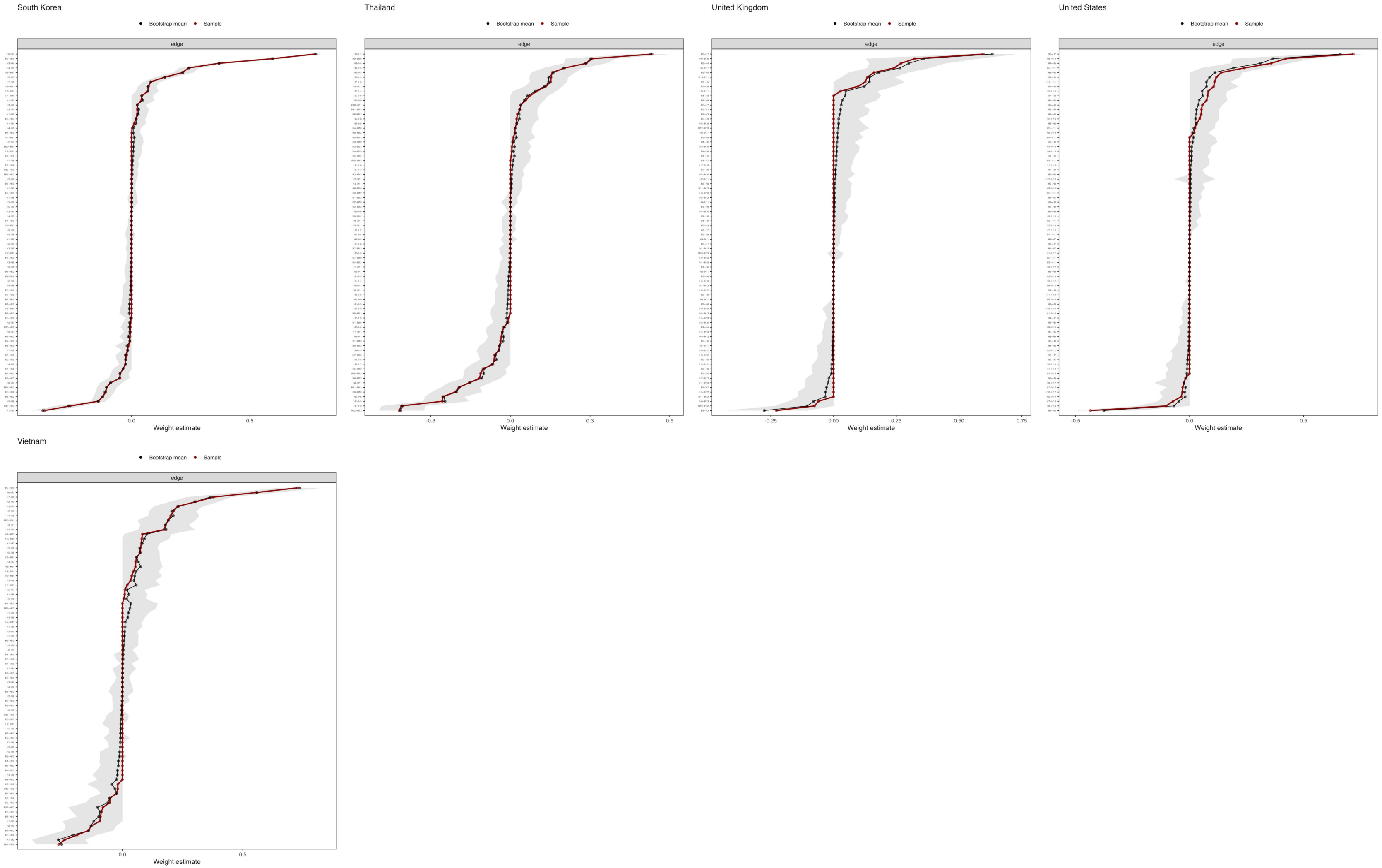

South Korea
Bootstrap mean
Sample
edge
0.0
0.5
Weight estimate
Thailand
Bootstrap mean
Sample
edge
-0.3
0.0
0.3
0.6
Weight estimate
United Kingdom
Bootstrap mean
Sample
edge
-0.25
0.00
0.25
0.50
0.75
Weight estimate
United States
Bootstrap mean
Sample
edge
-0.5
0.0
0.5
Weight estimate
Vietnam
Bootstrap mean
Sample
edge
0.0
0.5
Weight estimate

**Figure S6.** The stability of expected influence centrality index in health outcomes network across 29 countries using case-dropping bootstrap.

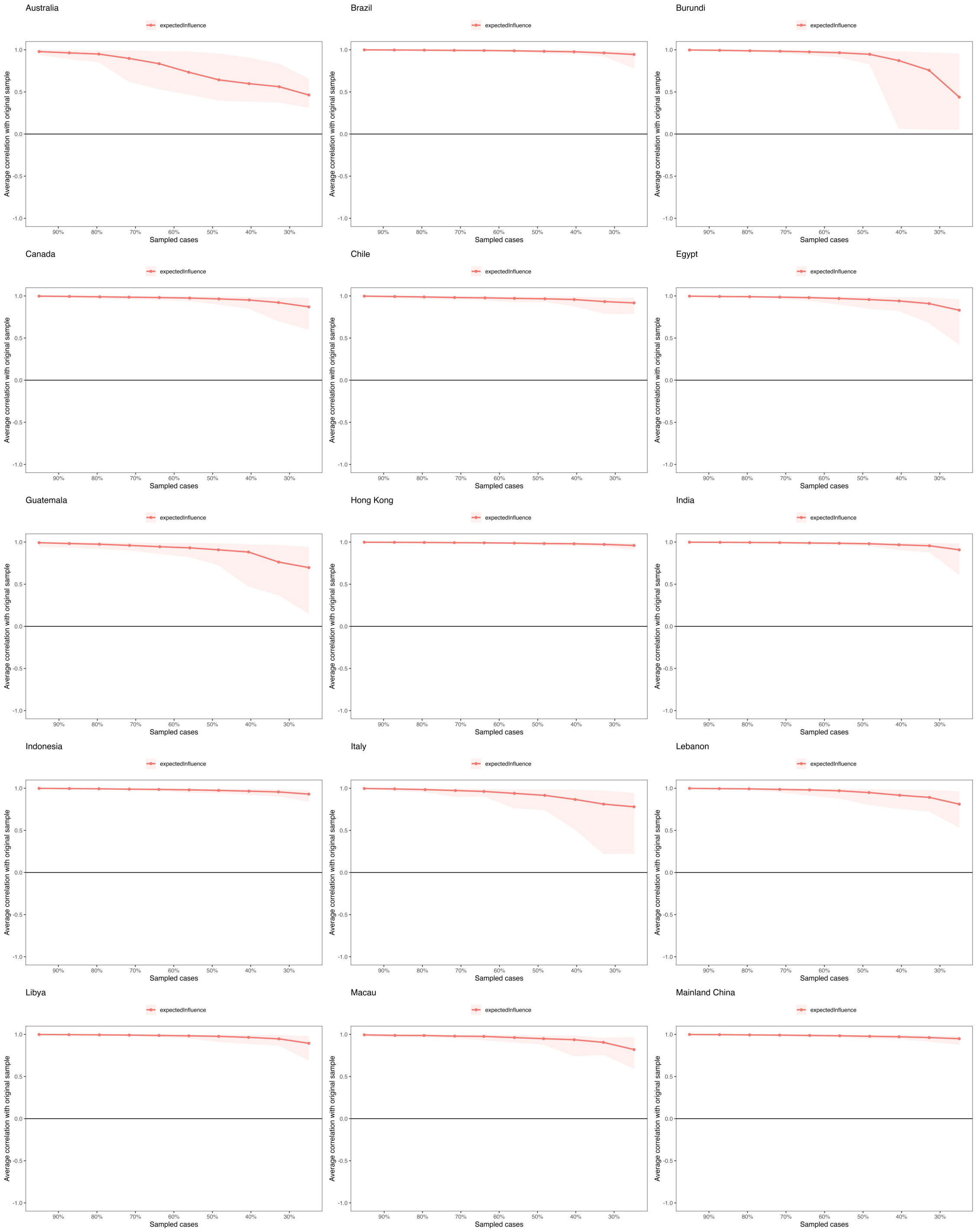

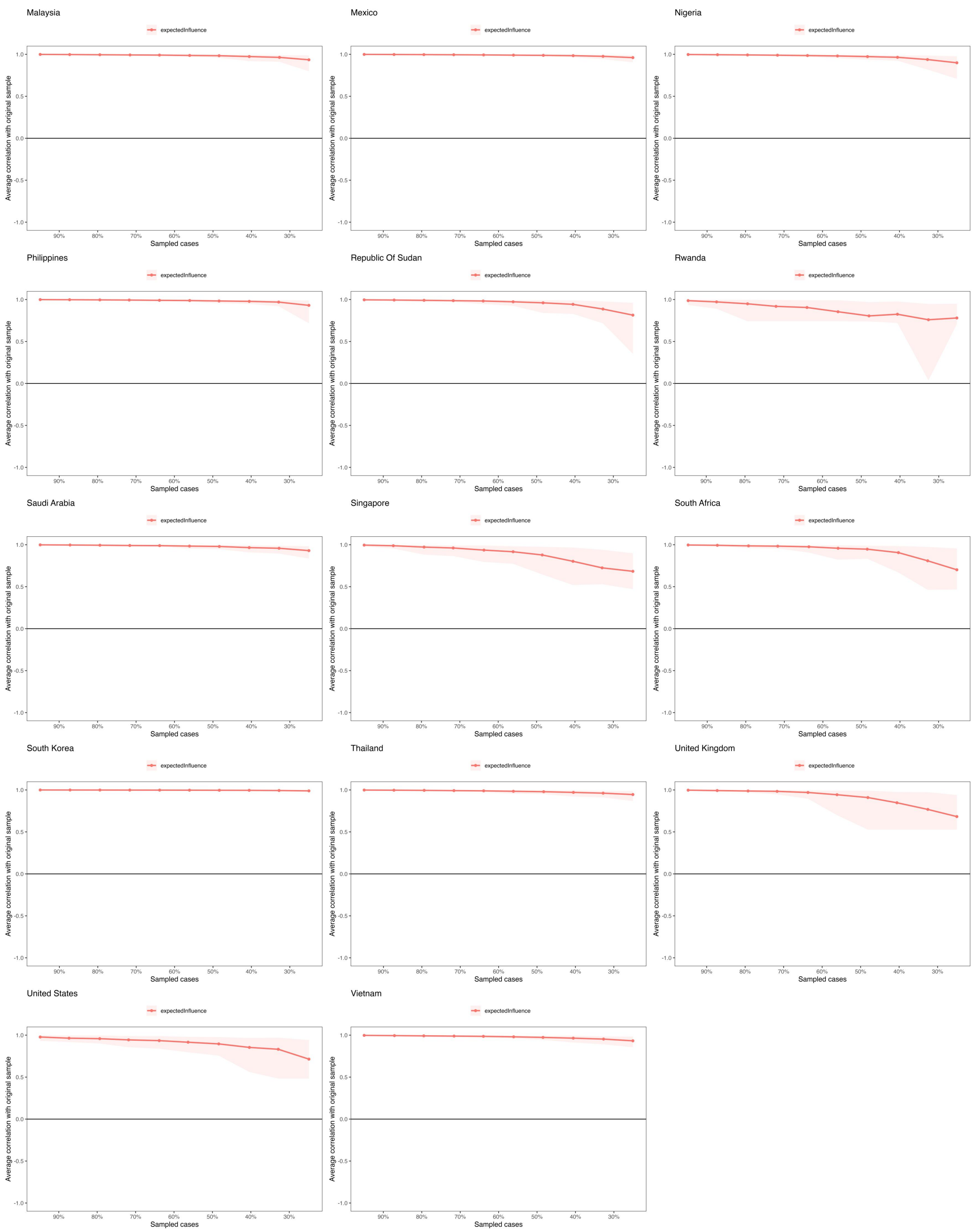

Malaysia
Mexico
Nigeria
Philippines
Republic Of Sudan
Rwanda
Saudi Arabia
Singapore
South Africa
South Korea
Thailand
United Kingdom
United States
Vietnam
expectedInfluence
Average correlation with original sample
1.0
0.5
0.0
-0.5
-1.0
90%
80%
70%
60%
50%
40%
30%
Sampled cases

**Figure S7.** Bootstrapped confidence intervals of edge weights for the bridge network across 29 countries.

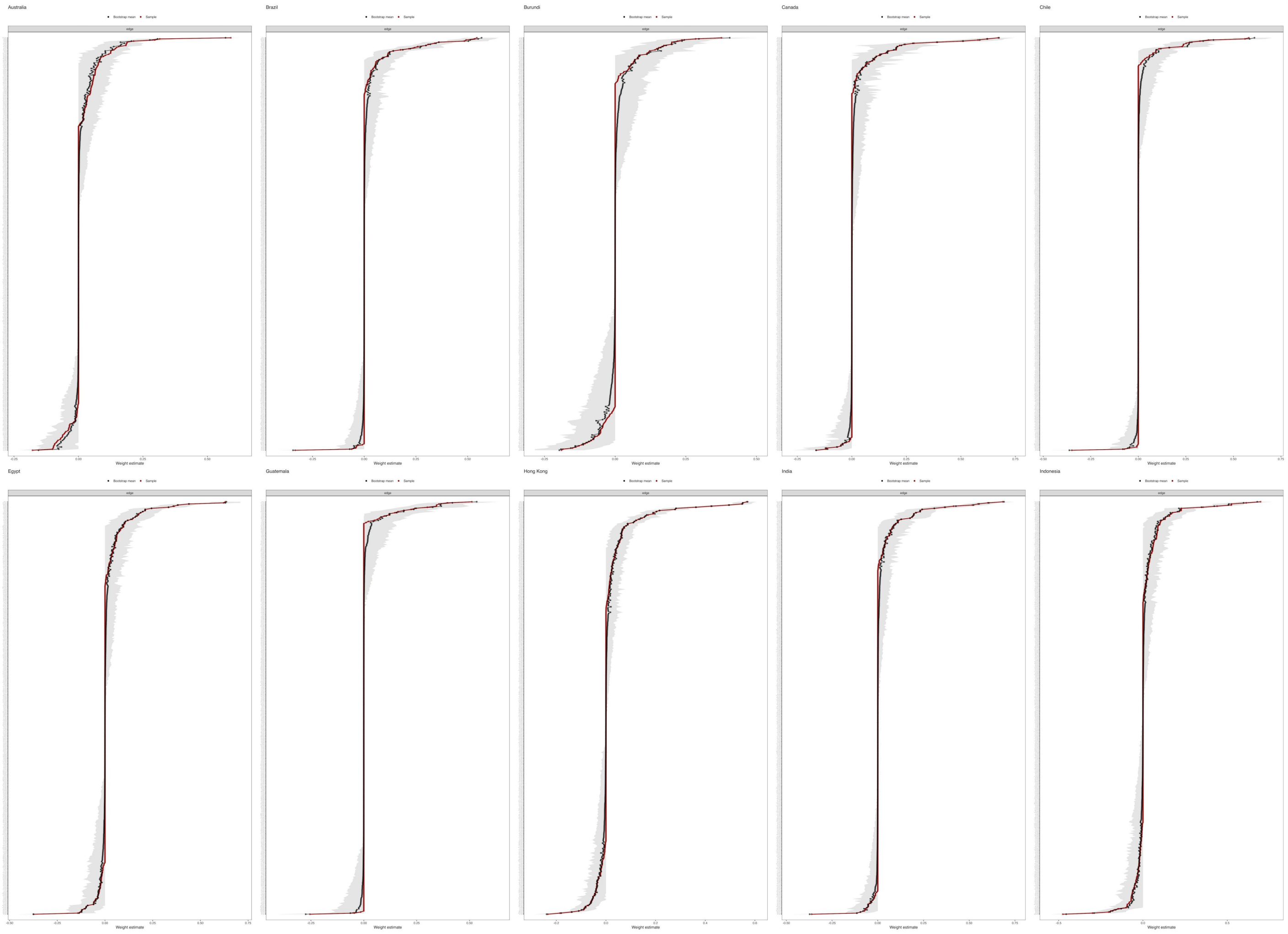

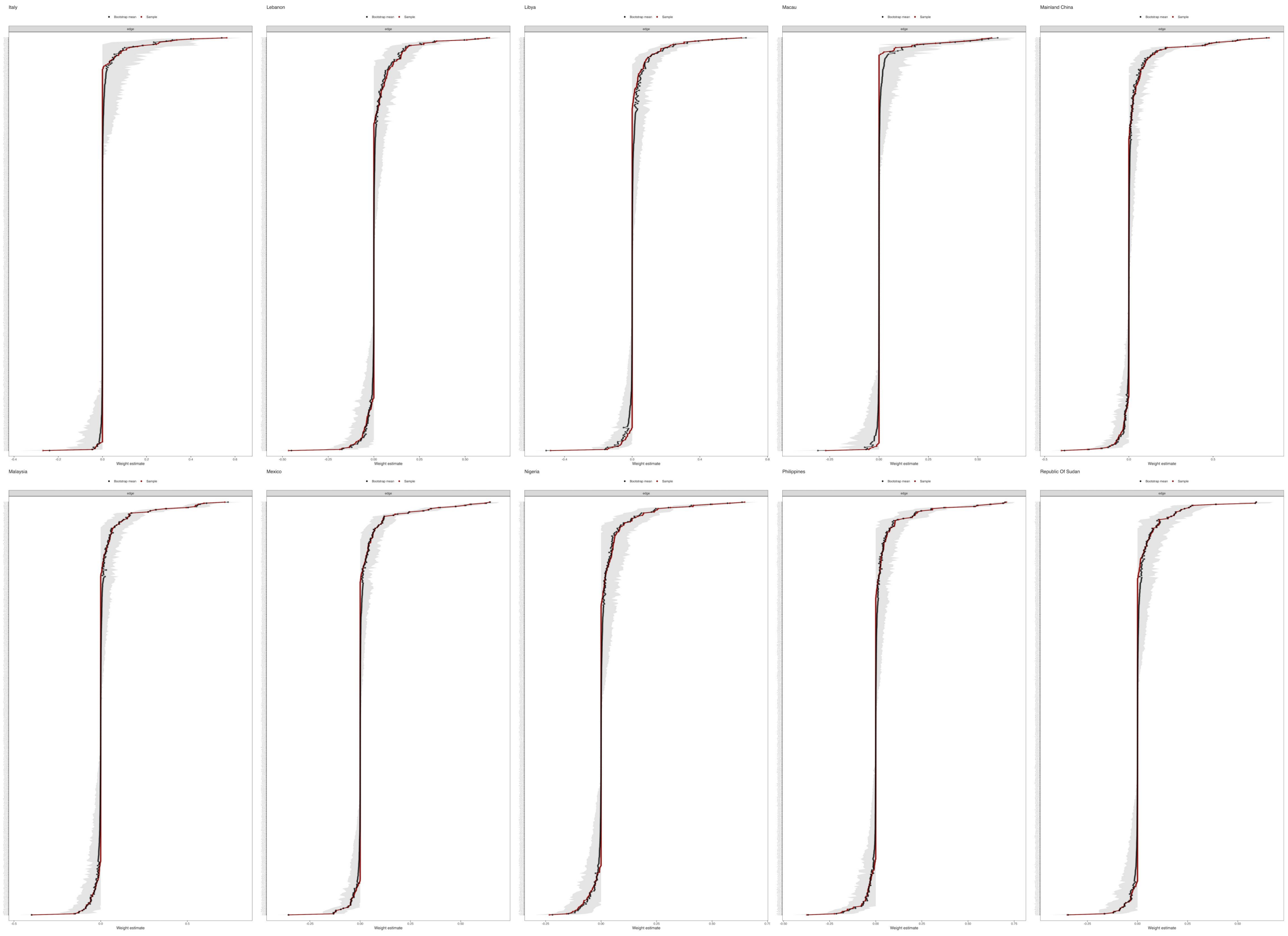
Italy
Lebanon
Libya
Macau
Mainland China
Malaysia
Mexico
Nigeria
Philippines
Republic Of Sudan
Bootstrap mean
Sample
edge
Weight estimate

**Figure S8.** The stability of bridge expected influence centrality index in bridge network across 29 countries using case-dropping bootstrap.

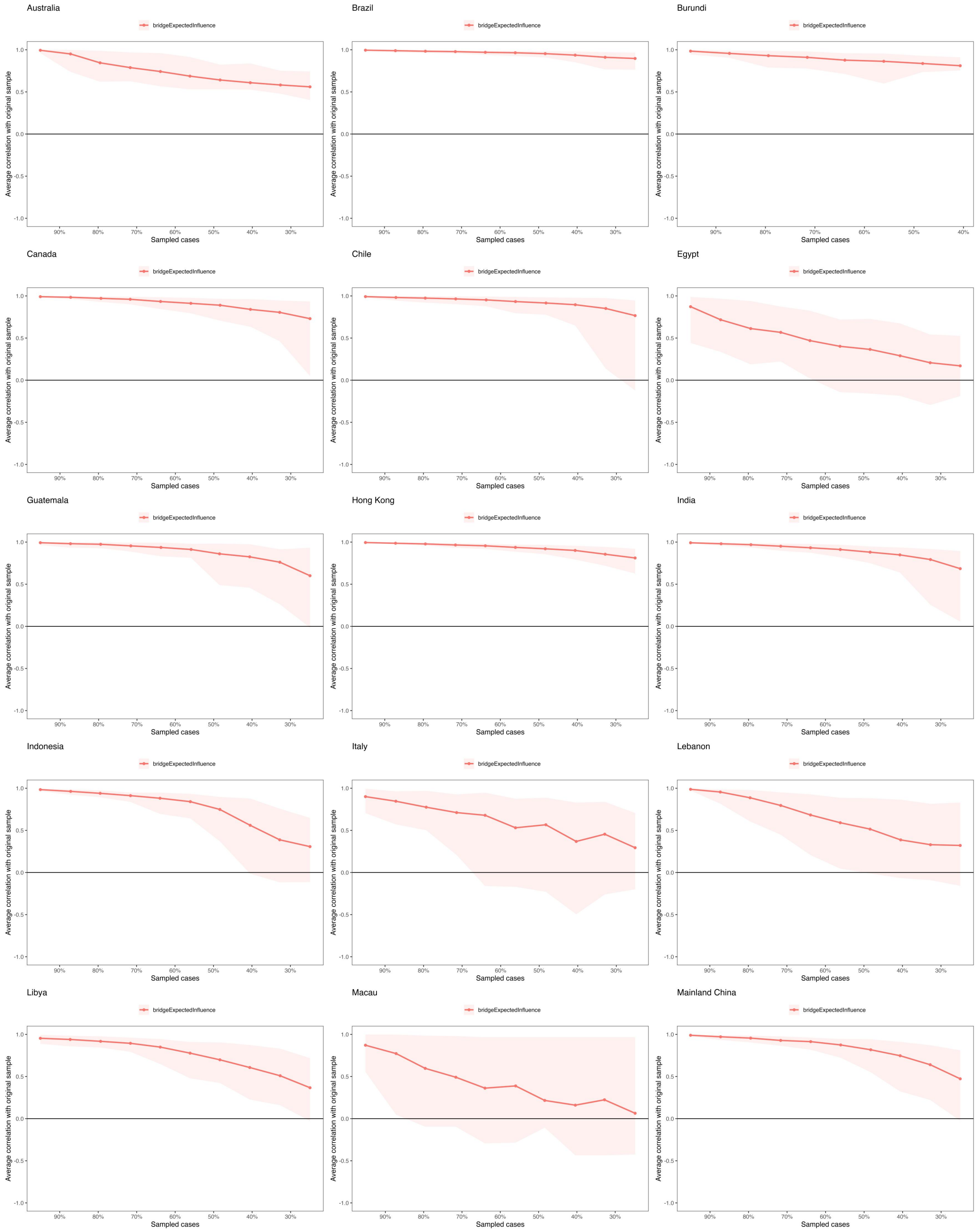

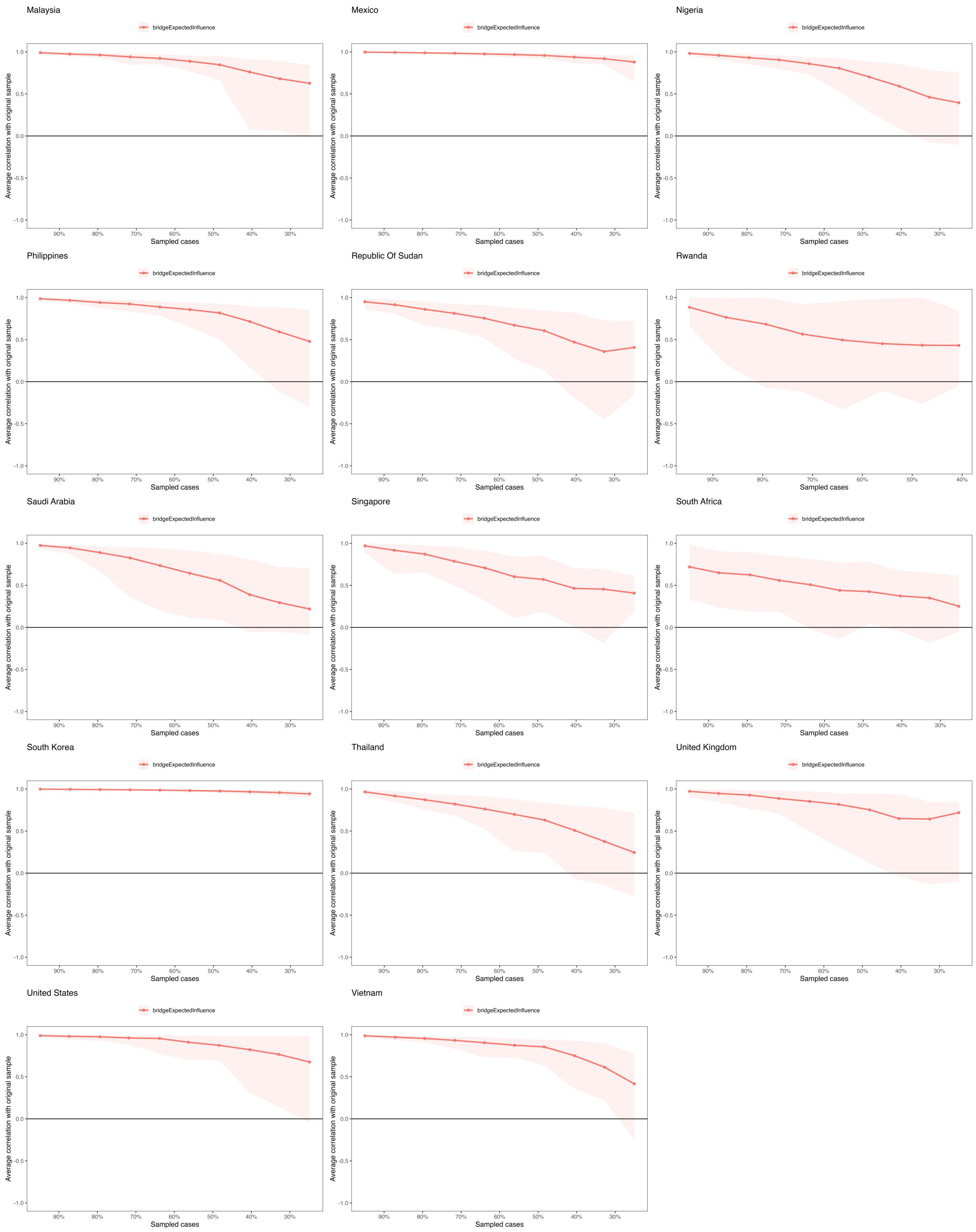
Malaysia
Mexico
Nigeria
Philippines
Republic Of Sudan
Rwanda
Saudi Arabia
Singapore
South Africa
South Korea
Thailand
United Kingdom
United States
Vietnam
bridgeExpectedInfluence
Average correlation with original sample
Sampled cases
90%
80%
70%
60%
50%
40%
30%
1.0
0.5
0.0
-0.5
-1.0
